# Functionalized Terthiophene as an Ambipolar Redox System: Structure, Spectroscopy, and Switchable Proton-Coupled Electron Transfer

**DOI:** 10.1021/jacs.4c16003

**Published:** 2025-01-27

**Authors:** Daniel Käch, Daniel Klose, Lionel Wettstein, Máté J. Bezdek

**Affiliations:** Department of Chemistry and Applied Biosciences, ETH Zürich, Vladimir-Prelog-Weg 1, 8093 Zürich, Switzerland

## Abstract

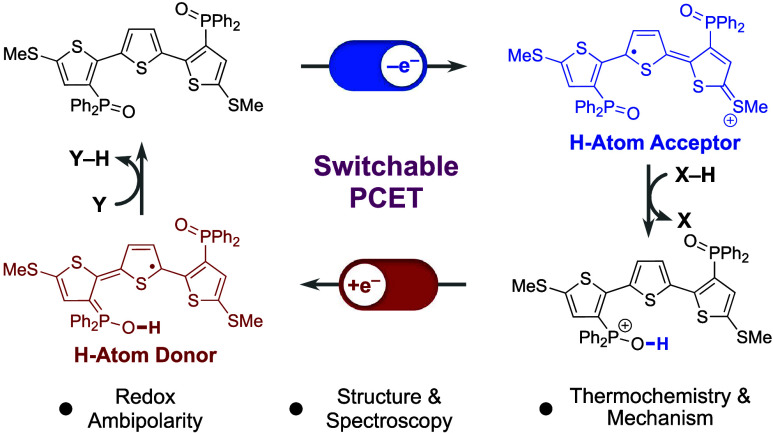

Organic redox systems that can undergo oxidative and reductive (ambipolar) electron transfer are elusive yet attractive for applications across synthetic chemistry and energy science. Specifically, the use of ambipolar redox systems in proton-coupled electron transfer (PCET) reactions is largely unexplored but could enable “switchable” reactivity wherein the uptake and release of hydrogen atoms are controlled using a redox stimulus. Here, we describe the synthesis and characterization of an ambipolar functionalized terthiophene (TTH) bearing methyl thioether and phosphine oxide groups that exhibits switchable PCET reactivity. Electrochemical studies established that the functionalized TTH can be reversibly oxidized and reduced, prompting the synthesis and characterization of cationic and anionic radicals on a single TTH platform. Combined structural, spectroscopic, and computational investigations revealed the influence of the methyl thioether and phosphine oxide moieties on the TTH electronic structure that results in the stabilization of both cationic and anionic radicals. Upon single-electron oxidation, the functionalized TTH serves as a hydrogen atom acceptor and undergoes PCET with 1,4-dihydroquinone to generate a TTH hydroxyphosphonium species. The process was found to be reversible upon single-electron reduction, with functionalized TTH acting as a hydrogen atom donor in a PCET reaction with 2,3-dimethylanthraquinone. The thermochemistry of the O–H bond formed and cleaved in functionalized TTH during the reaction sequence was investigated, revealing that a bond weakening of 30 kcal/mol underpins the switchable PCET reactivity. Overall, these studies provide an electrochemical, structural, spectroscopic, and thermochemical foundation for the use of ambipolarity to control PCET reactions in organic redox systems.

## Introduction

**Scheme 1:**
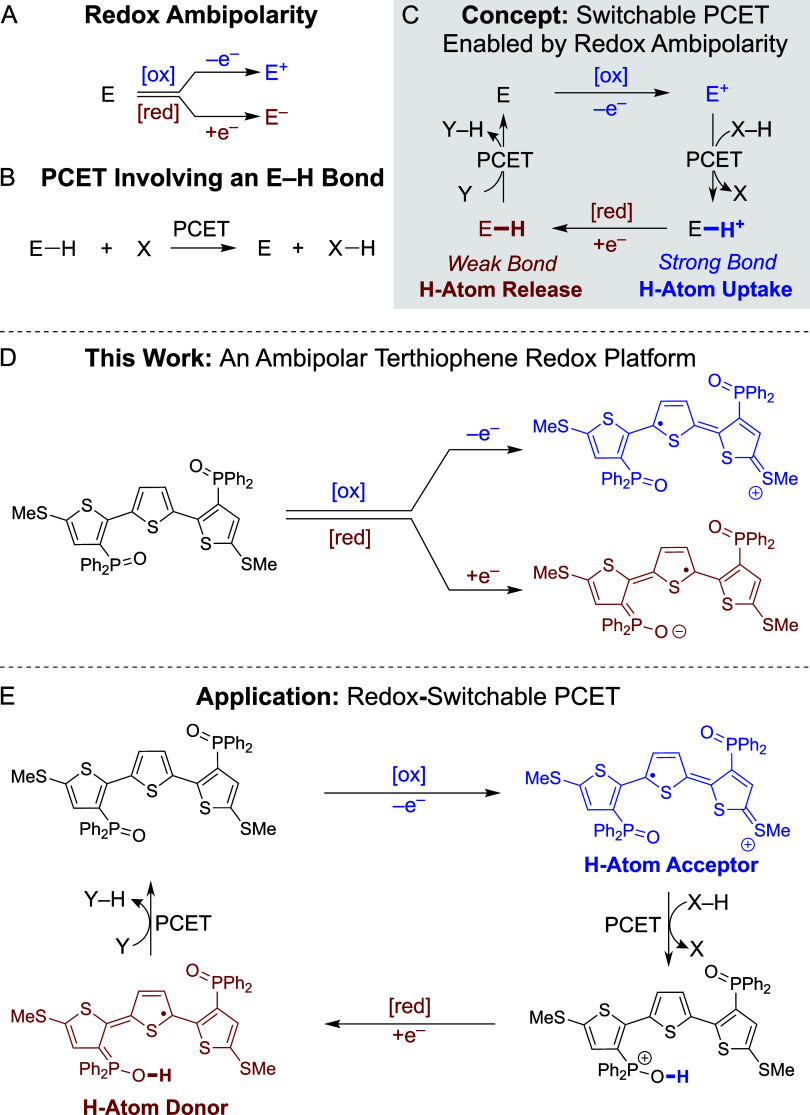
(A) Redox Ambipolarity, (B) PCET of an E–H Bond, (C) Switchable PCET, (D) The Ambipolar Terthiophene Redox System Presented in this Work and its (E) Application

Organic redox systems are attractive for use as components in post-silicon electronic materials, photovoltaics, batteries, and sensors.^1^ In particular, “ambipolar” organic compounds that can undergo both oxidative (anodic) and reductive (cathodic) electron transfer are promising for applications that currently employ separate materials to carry out each type of redox function (Scheme 1A).^2^ For instance, ambipolar organic electrolytes could simultaneously serve as catholytes and anolytes in symmetric redox flow batteries.^3^ Furthermore, single-component ambipolar materials capable of transporting both holes and electrons could streamline microelectronics that currently rely on separate p- and n-type semiconductor channels.^4^ While sustainability and economic considerations motivate a pivot from the use of metals in these settings, the highly reactive nature of organic radicals upon electron transfer hinders their widespread implementation.^5^ It is hence imperative to identify organic structural motifs that exhibit well-defined ambipolar redox chemistry, and to explore new applications for this feature.

An underexplored area for organic ambipolar redox systems is their application in proton-coupled electron transfer (PCET) schemes. Reagents that relay proton and electron equivalents are increasingly being utilized as mediators in redox flow batteries^6^ and dye sensitized solar cells,^7^ in addition to their use in aerobic oxidation,^8^ olefin hydrofunctionalization^9^ and small-molecule activation reactions.^10^ As shown in Scheme 1B, the PCET reactivity between an H atom donor (E–H) and acceptor (X) is dictated by differences in E–H/X–H bond dissociation free energies (BDFEs), which necessitates their matching to effect the desired X–H bond formation.^11^ This thermodynamic requirement typically restricts the variety of PCET reagents that can be used in a particular application. In this context, scaffolds in which E–H BDFEs can be toggled by an external stimulus would provide a convenient handle for controlling PCET reactivity. Because E–H BDFE is a thermodynamic parameter that is directly proportional to redox potential,^12^ ambipolar redox systems are well-suited to provide “switchable” control over PCET reactivity through redox-induced changes in E–H BDFE (Scheme 1C). This strategy would uniquely enable a single reagent to serve as both a hydrogen atom donor and acceptor, depending on the redox stimulus. While BDFE modulation via redox chemistry is documented for transition metal complexes,^13^ analogous processes with organic molecules remain challenging to control.^14^

As part of an ongoing interest in the development of organic redox systems, our research group recently reported that the functionalization of 2,2′:5′,2″-terthiophene (TTH) with phosphine oxides unlocks its well-defined cathodic electron transfer reactivity.^15^ While oligothiophenes such as TTH generally exhibit limited stability upon reduction, the phosphine oxide functionalities uniquely provide electronic and steric stabilization for an otherwise highly reactive TTH radical anion. Consequently, we questioned whether well-defined anodic redox processes could also be accessed in phosphine oxide-functionalized TTH to yield an overall ambipolar redox system. Given the ability of phosphine oxides to serve as proton transfer sites,^16^ pairing this feature with redox ambipolarity at the terthiophene core could be the basis for a redox-switchable PCET concept (Scheme 1C). However, the chemical instability of TTH upon oxidation has so far prevented access to ambipolar reactivity.

In this work, we report an ambipolar TTH featuring methyl thioether and phosphine oxide functionalities that can accept and release H atom equivalents upon application of redox stimuli. An array of characterization methods including single-crystal X-ray diffraction (sc-XRD), electron paramagnetic resonance (EPR) and UV-vis-NIR spectroscopy establish the synergistic role of the functionalities in stabilizing both cationic and anionic TTH-based radicals in an overall ambipolar system (Scheme 1D). Further, we show that redox ambipolarity can be leveraged to control dual PCET reactivity in functionalized TTH. Specifically, functionalized TTH is shown to serve as a hydrogen atom acceptor upon single-electron oxidation, and as a hydrogen atom donor upon reduction (Scheme 1E). As such, the formally endergonic hydrogen atom transfer from 1,4-dihydroquinone to 2,3-dimethylanthraquinone is accomplished using functionalized TTH as a redox-switchable mediator. Finally, the BDFEs of O–H bonds formed and cleaved during the reaction sequence are presented, providing thermochemical basis for a new strategy to control PCET in an organic system.

## Results and Discussion

### Synthesis and Ambipolar Redox Chemistry of **4**

**Scheme 2:**
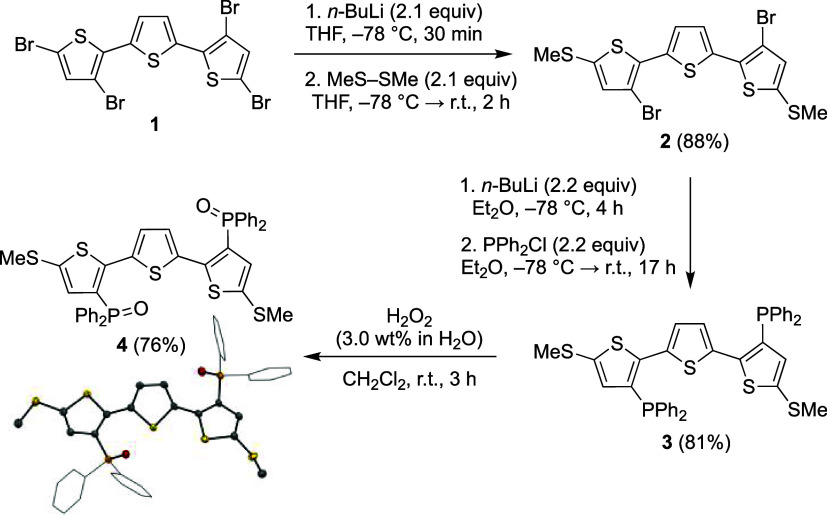
Synthesis and Solid-State Structure of **4** Represented with 50% probability ellipsoids, hydrogen atoms omitted for clarity.

Our studies commenced with the synthesis of a terthiophene bearing phosphine oxide and methyl thioether functionalities. Shown in Scheme 2, installation of a –SMe group was accomplished by lithiation of 3,3″,5,5″-Br_4_-TTH (**1**)^17^ and subsequent treatment with dimethyl disulfide, furnishing 3,3″-Br_2_-5,5″-(SMe)_2_-TTH (**2**) in 88% yield. Product **2** was treated with *n*-BuLi and PPh_2_Cl consecutively to achieve P–C bond formation, affording 3,3″-(PPh_2_)_2_-5,5″-(SMe)_2_-TTH (**3**) in 81% yield. Consistent with reported phosphine-functionalized oligothiophenes,^18^**3** slowly oxidizes in air. To achieve controlled oxidation, **3** was treated with excess H_2_O_2_ enabling isolation of the target molecule 3,3″-(P(O)Ph_2_)_2_-5,5″-(SMe)_2_-TTH (**4**) as a crystalline yellow solid in 76% yield. The solid-state structure of **4** was confirmed by sc-XRD (Scheme 2). The ^1^H and ^31^P{^1^H} NMR spectra of **4** in CDCl_3_ were found to be consistent with a C_2v_ molecular symmetry that is indicative of free rotation about the interthiophene C–C, C–P and C–S bonds.

**Figure 1:**
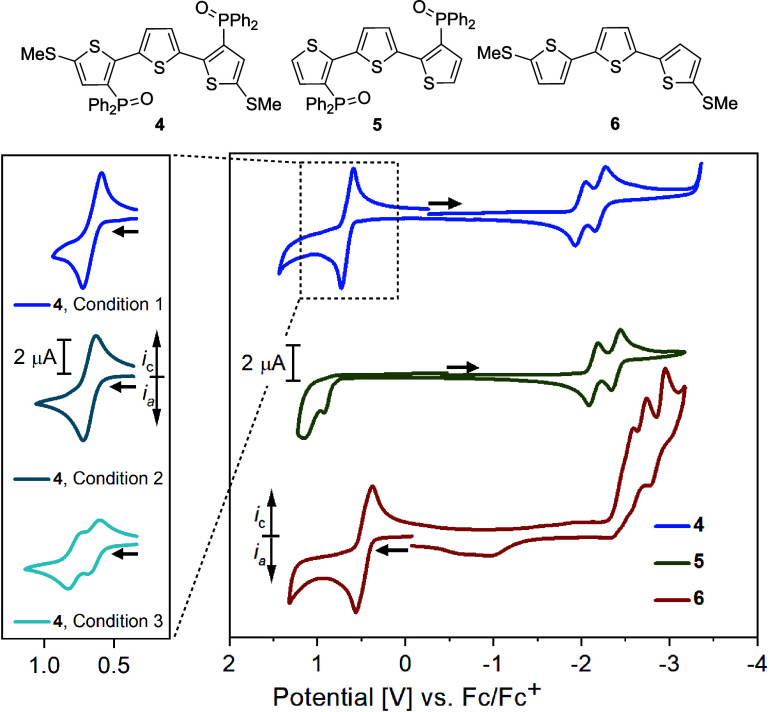
Cyclic voltammograms of **4**, **5**, and **6** (1.0 mM concentration with 0.10 M [(*n*-Bu)_4_N][PF_6_] supporting electrolyte in DME, 100 mV sec^–1^ scan rate, r.t., glassy carbon working electrode). *i*_c_: cathodic current, *i*_a_: anodic current. Inset: cyclic voltammograms of **4** collected under various conditions. Condition 1: 0.10 M [(*n*-Bu)_4_N][PF_6_] supporting electrolyte in DME. Condition 2: 0.10 M [(*n*-Bu)_4_N][PF_6_] supporting electrolyte in DFB. Condition 3: 0.10 M [(*n*-Bu)_4_N][Al(OR^*F*^)_4_] supporting electrolyte in DFB.

The electrochemical behavior of **4** was examined by cyclic voltammetry (CV) to probe the suitability of the installed functionalities to induce redox ambipolarity. The cyclic voltammogram of **4** in DME solution at room temperature exhibits a reversible anodic wave as well as two reversible cathodic waves with half-wave potentials (*E*_1/2_) of +0.66, −1.97, and −2.22 V vs Fc/Fc^+^ (Fc = ferrocene), respectively (Figure 1). Square wave voltammetry revealed that the feature at +0.66 V corresponds to net two-electron oxidation, while successive one-electron reduction events take place at −1.97 and −2.22 V (Figure S16). Intriguingly, the two-electron anodic wave of **4** can be resolved into two successive one-electron oxidations with *E*_1/2_ = +0.79 and +0.64 V vs Fc/Fc^+^ using noncoordinating 1,2-difluorobenzene (DFB) solvent and [(*n*-Bu)_4_N][Al(OR^*F*^)_4_] (R^*F*^ = C(CF_3_)_3_) as the supporting electrolyte (Figure 1 inset and Figure S20).^19^ While the first oxidation at *E*_1/2_ = +0.64 V was found to be fully reversible, the second anodic wave at *E*_1/2_ = +0.79 V exhibits quasi-reversible character (Figure S19). From these electrochemical data, we surmised that access to **4**^**+**^ and **4**^**–**^ should be possible by judicious selection of reaction conditions for the single-electron chemical oxidation and reduction of **4**, respectively.

To probe whether both the phosphine oxide and methyl thioether functionalities are necessary for the observed ambipolar electrochemical behavior of **4**, the cyclic voltammograms of 3,3″-(P(O)Ph_2_)_2_-TTH (**5**)^15^ and 5,5″-(SMe)_2_-TTH (**6**)^20^ were collected in DME solution for comparison (Figure 1). Interestingly, **5** maintains irreversibility in its anodic features but exhibits reversible cathodic waves at −2.14 and −2.40 V. Inversely, **6** exhibits a reversible anodic wave at +0.48 V but irreversible cathodic features. Hence, the phosphine oxide functionalities are responsible for the observed reductive stability of **4** while the presence of the methyl thioether moieties result in its reversible electrochemical oxidation. Therefore, the combination of both functionalities uniquely enables redox ambipolarity in **4**.

**Figure 2:**
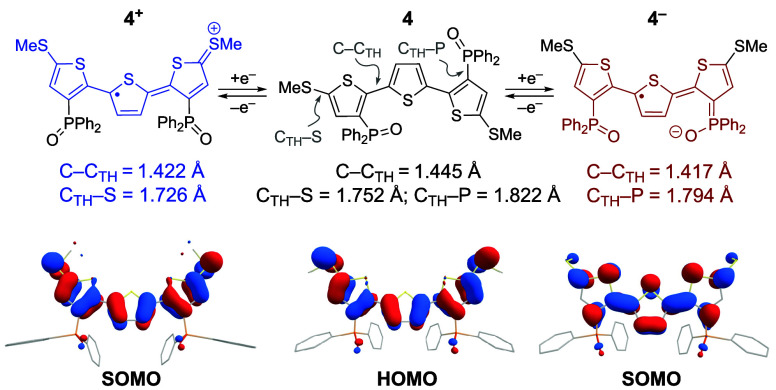
DFT-computed (BP86/def2-TZVP) one-electron oxidation and reduction of **4** to generate **4**^**+**^ and **4**^**–**^, respectively, together with key bond lengths and frontier orbital illustrations.

The observed redox ambipolarity of **4** motivated exploration of the electronic structures of its oxidized and reduced forms. Shown in Figure 2, the frontier orbitals of **4**^**+**^ and **4**^**–**^ were computed by density functional theory (DFT) at the BP86/def2-TZVP level of theory and found to be consistent with the established electronic structures of oxidized (p-type) and reduced (n-type) oligothiophenes, respectively.^21^ Specifically, while the highest occupied molecular orbital (HOMO) of **4** exhibits “benzenoidal” (aromatic) character, removal or addition of an electron results in “quinoidal” singly occupied molecular orbitals (SOMOs) in **4**^**+**^ and **4**^**–**^.^22^ A diagnostic feature of the quinoidal bonding in oligothiophenes is the planarization of the terthiophene backbone with concomitant contraction of interthiophene C–C bonds (C–C_TH_) that was observed in the geometry-optimized structures of both **4**^**+**^ and **4**^**–**^. In addition, an increased C–P bond order was observed for **4**^**–**^ between thiophene and phosphine oxide moieties (C_TH_–P) that signals the role of the Ar_2_P(O) substituents in stabilizing reduced TTH. Finally, the shortening of C–S bonds between thiophene and methyl thioether groups in **4**^**+**^ (C_TH_–S) establishes the stabilizing influence of the SMe moieties upon oxidation of **4**. These results corroborate the unique role of each functionality in providing resonance stabilization for either cationic or anionic terthiophene radicals.

### Synthesis and Structural Characterization of **4**^**–**^ and **4**^**+**^

**Scheme 3:**
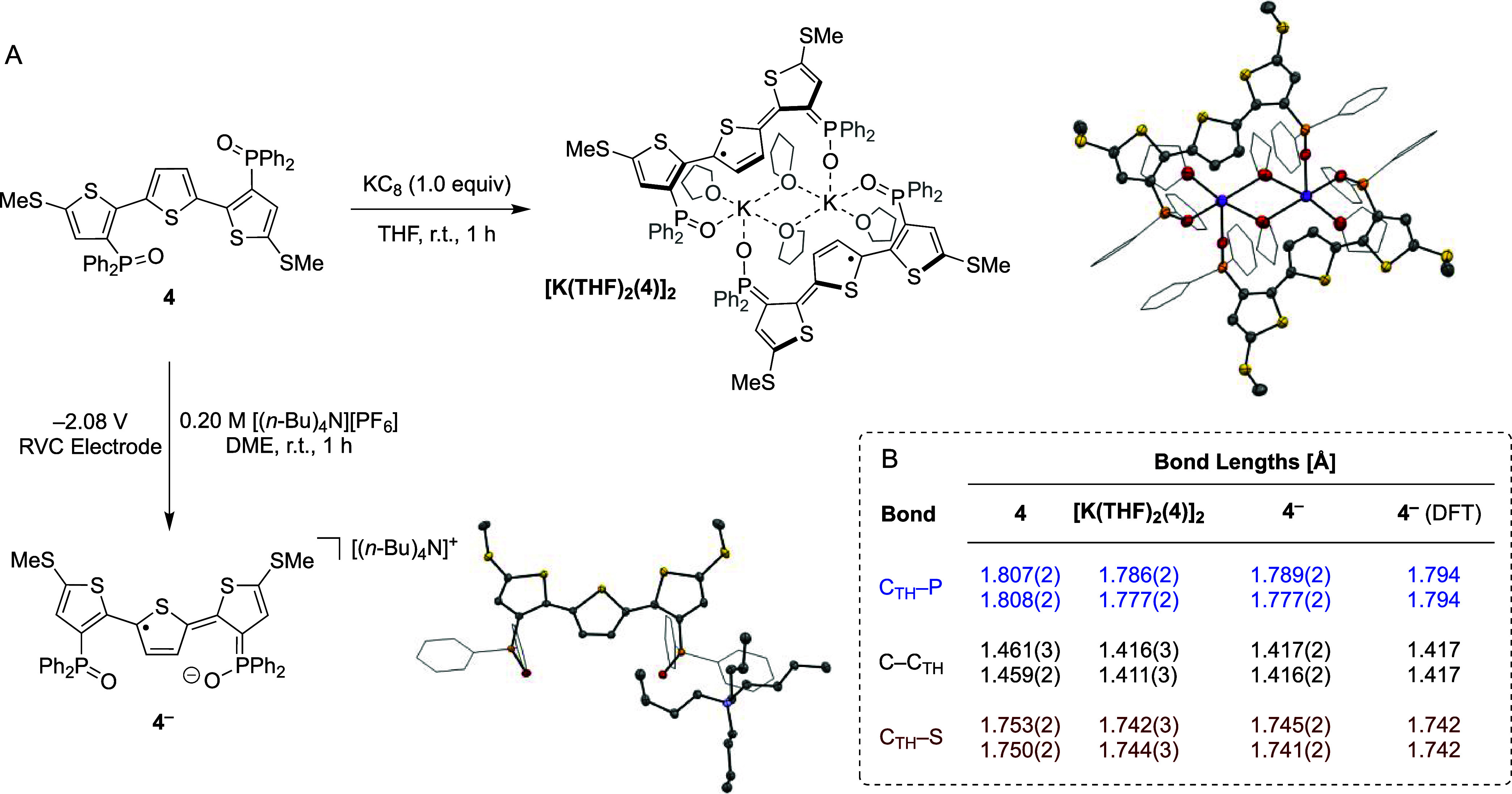
(A) Synthesis and Solid-State Structures of [K(THF)_2_(4)]_2_ and **4**^**–**^ together with (B) Key Bond Lengths Represented with 50% probability ellipsoids (hydrogen atoms omitted, phenyl groups and solvent atoms represented without probability ellipsoids for clarity).

To gain deeper insight into the structural and spectroscopic features of **4**^**–**^ and **4**^**+**^, we pursued their synthesis and isolation. To achieve single-electron reduction, **4** was treated with one equivalent of potassium graphite (KC_8_, Scheme 3A). The reaction was carried out at room temperature for 1 h, during which time a color change from pale yellow to dark blue was observed. The product of the reaction was identified as the potassium complex [K(THF)_2_(**4**)]_2_ isolated in 90% yield. The solid-state structure of the complex was determined by sc-XRD. Shown in Scheme 3A, [K(THF)_2_(**4**)]_2_ exhibits a dimeric structure, wherein each potassium atom is coordinated by two phosphine oxides, in addition to one terminal and two bridging THF solvent molecules. The terthiophene backbones adopt a planar arrangement, which is accompanied by several bond length changes compared to **4**. These include a shortening of the interthiophene C–C bonds (C–C_TH_) in addition to contraction of C–P bonds between thiophene and the phosphine oxide moieties (C_TH_–P) in [K(THF)_2_(**4**)]_2_ (Scheme 3B). As supported by DFT computations (see Figure 2), these structural distortions are consistent with a TTH-based reduction event and concomitant electronic structure shift to a quinoidal bonding scenario with the phosphine oxide moieties providing resonance stabilization. Hence, each functionalized TTH unit in [K(THF)_2_(**4**)]_2_ is best described as a radical anion wherein coordination to potassium gives an overall neutral complex.

To investigate whether the isolation of **4**^**–**^ could be accomplished without potassium intercalation, its synthesis was also pursued by electrochemical means. Accordingly, controlled-potential electrolysis of **4** (5.0 mM in DME) was carried out at −2.08 V (vs Fc/Fc^+^) using a reticulated vitreous carbon (RVC) working electrode in the presence of [(*n*-Bu)_4_N][PF_6_] (0.20 M) supporting electrolyte. After stirring at room temperature for 1 h, the singly reduced analog **4**^**–**^ was isolated as a tetra-*n*-butylammonium salt after recrystallization. Single-crystal X-ray diffraction revealed that diagnostic C–C_TH_ and C_TH_–P bond lengths in the solid-state structure of **4**^**–**^ closely match those determined for [K(THF)_2_(**4**)]_2_ (Scheme 3B). Notably, the DFT-computed bond lengths for **4**^**–**^ are in good agreement with the experimental values determined by sc-XRD. These findings thus provide experimental evidence for diagnostic structural changes that accompany single-electron reduction at TTH.

**Scheme 4:**
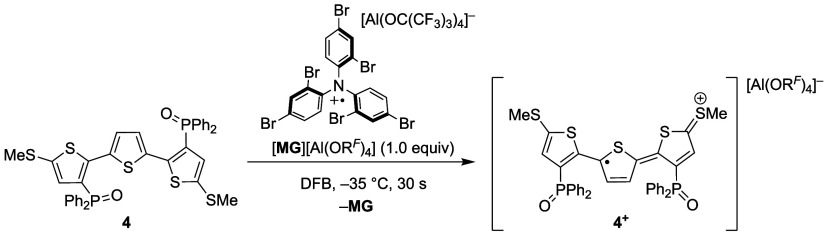
Synthesis of [**4**][Al(OR^*F*^)_4_]

Following our investigation into the reduction of **4**, we turned our attention to its chemical oxidation. To access **4**^**+**^, we looked to utilize the brominated triarylammonium radical cation “Magic Green” as an oxidant ([**MG**]^**+**^ = [N(2,4-Br_2_-C_6_H_3_)_3_]^+^), as it features a sufficiently high oxidation potential.^23^ We chose to pair this cation with [Al(OR^*F*^)_4_]^−^ due to its chemical inertness under highly oxidizing conditions and favorable solubility properties. Further, the electrochemical behavior of **4** implied that the presence of the [Al(OR^*F*^)_4_]^−^ counterion is beneficial for accessing **4**^**+**^ without over-oxidation to **4**^**2+**^ (Figure 1 inset). Accordingly, we synthesized [**MG**][Al(OR^*F*^)_4_] in analogy to procedures reported for related triarylammonium aluminates^24^ and examined its electrochemical properties by cyclic voltammetry (see Supporting Information (SI) for details). These studies revealed a fully reversible anodic wave with *E°* = +1.13 V (vs Fc/Fc^+^) in MeCN (see Figure S23), confirming the suitability of [**MG**]^+^ for the oxidation of **4** (*E°* = +0.68 V in MeCN). Indeed, addition of [**MG**][Al(OR^*F*^)_4_] to **4** at −35 °C resulted in a color change from clear yellow to dark turquoise within seconds. Analysis of the reaction mixture by ^1^H NMR spectroscopy indicated formation of N(2,4-Br_2_-C_6_H_3_)_3_ (**MG**) as the sole diamagnetic product, which was taken as evidence for electron transfer (Scheme 4 and Figure S6). The inertness of the [Al(OC(CF_3_)_3_)_4_]^−^ anion was confirmed by ^19^F and ^27^Al NMR spectroscopy, with its diagnostic signals at −76.0 ppm (^19^F NMR) and −34.6 ppm (^27^Al NMR) remaining unchanged during the course of the reaction. While the limited stability of **4**^**+**^ precluded structural characterization, its extended lifetime at low temperatures (<−35 °C) enabled the acquisition of spectroscopic data. These investigations are described in detail below.

### Spectroscopic Characterization of **4**^**–**^ and **4**^**+**^

**Figure 3:**
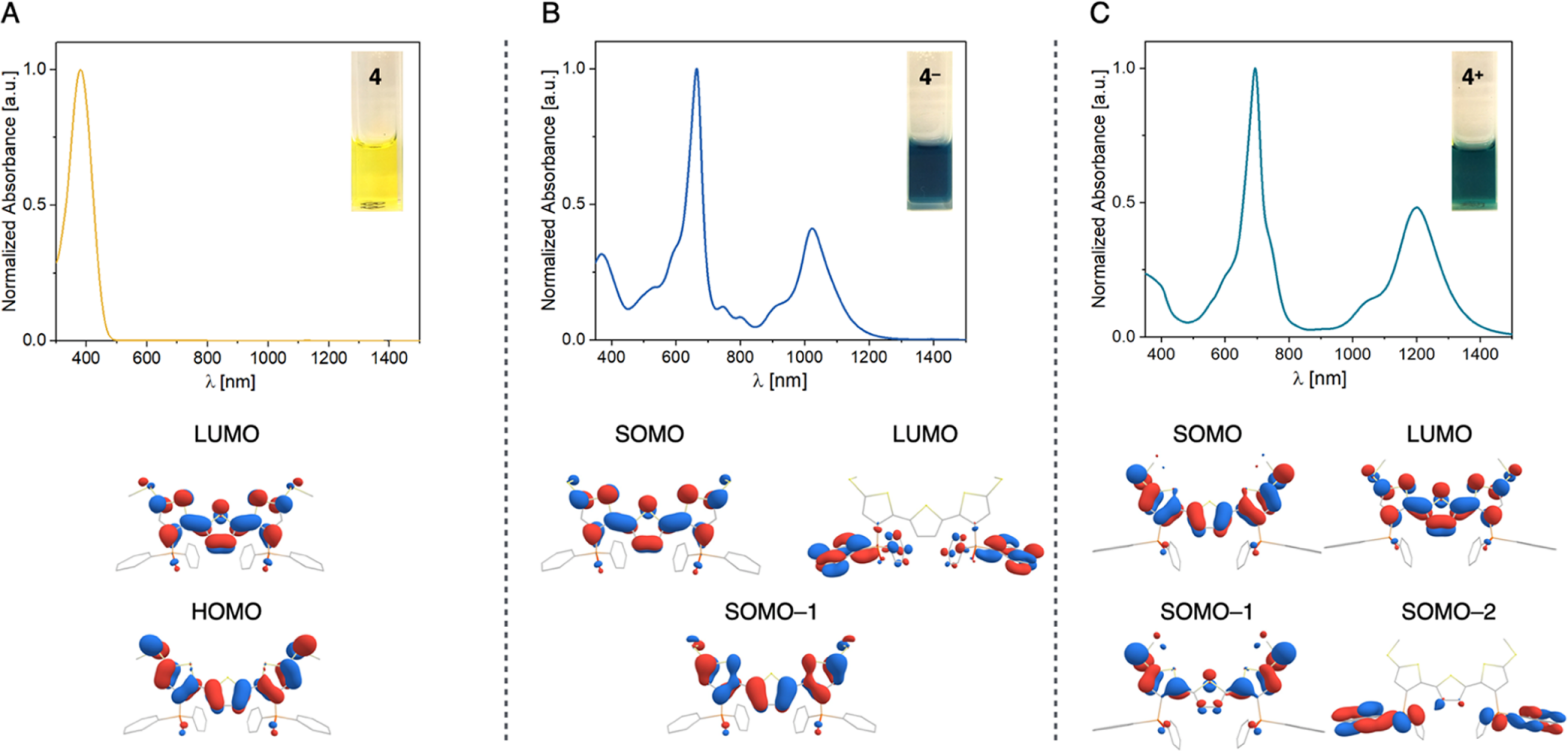
UV-vis-NIR spectra (DFB solution, top) together with sample photographs (inset) and relevant DFT-computed frontier molecular orbitals (bottom) for **4** (A), **4**^**–**^ (B) and **4**^**+**^ (C).

The synthesis of **4**^**–**^ and **4**^**+**^ provided a rare opportunity to study the spectroscopic features of anionic and cationic TTH radicals on a single platform. Accordingly, we examined the optical properties of **4**, **4**^**–**^ and **4**^**+**^ by UV-vis-NIR spectroscopy. Several noticeable differences are present in the electronic absorption spectra of **4**^**–**^ and **4**^**+**^ compared to neutral **4**. As shown in Figure 3A, **4** is clear yellow in DFB solution and exhibits one absorption band with λ_max_ = 381 nm. By contrast, **4**^**–**^ is deep blue and **4**^**+**^ is deep turquoise in color and both display intense absorptions in the visible and NIR regions. In addition to a feature at λ_max_ = 369 nm, **4**^**–**^ shows absorption bands at λ_max_ = 664 and 1023 nm that are each accompanied by vibronic transitions (shoulders) at 600 and 920 nm, respectively (Figure 3B). Similarly, **4**^**+**^ shows strong absorption features at λ_max_ = 695 and 1200 nm with respective vibronic shoulders at 606 and 1055 nm (Figure 3C).

To gain deeper insight into the optical absorption features of **4**, **4**^**–**^ and **4**^**+**^, time-dependent DFT (TD-DFT) computations were carried out [ωB97-D3, def2-TZVP, CPCM(1,2-DFB)]. In the case of **4**, TD-DFT computations predict an electronic transition at 387 nm, assignable to the lowest energy π → π* (HOMO → LUMO) transition of TTH. This result is in close agreement with the experimentally observed λ_max_ of 381 nm for **4** and is consistent with the established electronic absorption properties of oligothiophenes.^25^ For **4**^**–**^ and **4**^**+**^, TD-DFT computations also produce features that are in line with the experimentally observed UV-vis-NIR spectra of the compounds and hence were utilized to assign the nature of the observed transitions (Figures S86–S96). In the case of **4**^**–**^, the feature at λ_max_ = 369 nm is assigned to a SOMO–1 → SOMO transition involving orbitals that are delocalized across the TTH moiety with significant S atom contribution (Figure 3B). On the other hand, computations reveal that the absorptions at λ_max_ = 664 and 1023 nm correspond to SOMO → LUMO+N excitations to orbitals with principally TTH and Ar_2_P(O) π*-character in **4**^**–**^. The diarylphosphine oxide moieties are also implicated in the optical absorption characteristics of **4**^**+**^, wherein the transition at λ_max_ = 695 nm is assigned as the SOMO–2 → SOMO excitation from an orbital with significant Ar_2_P(O) π-character (Figure 3C). DFT computations also revealed that the lowest energy absorption in **4**^**+**^ (λ_max_ = 1200 nm) corresponds to a SOMO–1 → SOMO transition involving orbitals delocalized across TTH and SMe. Taken together, these results establish the electronic interplay of the TTH, SMe and Ar_2_P(O) moieties that give rise to the optical characteristics of **4**, **4**^**–**^ and **4**^**+**^.

**Figure 4:**
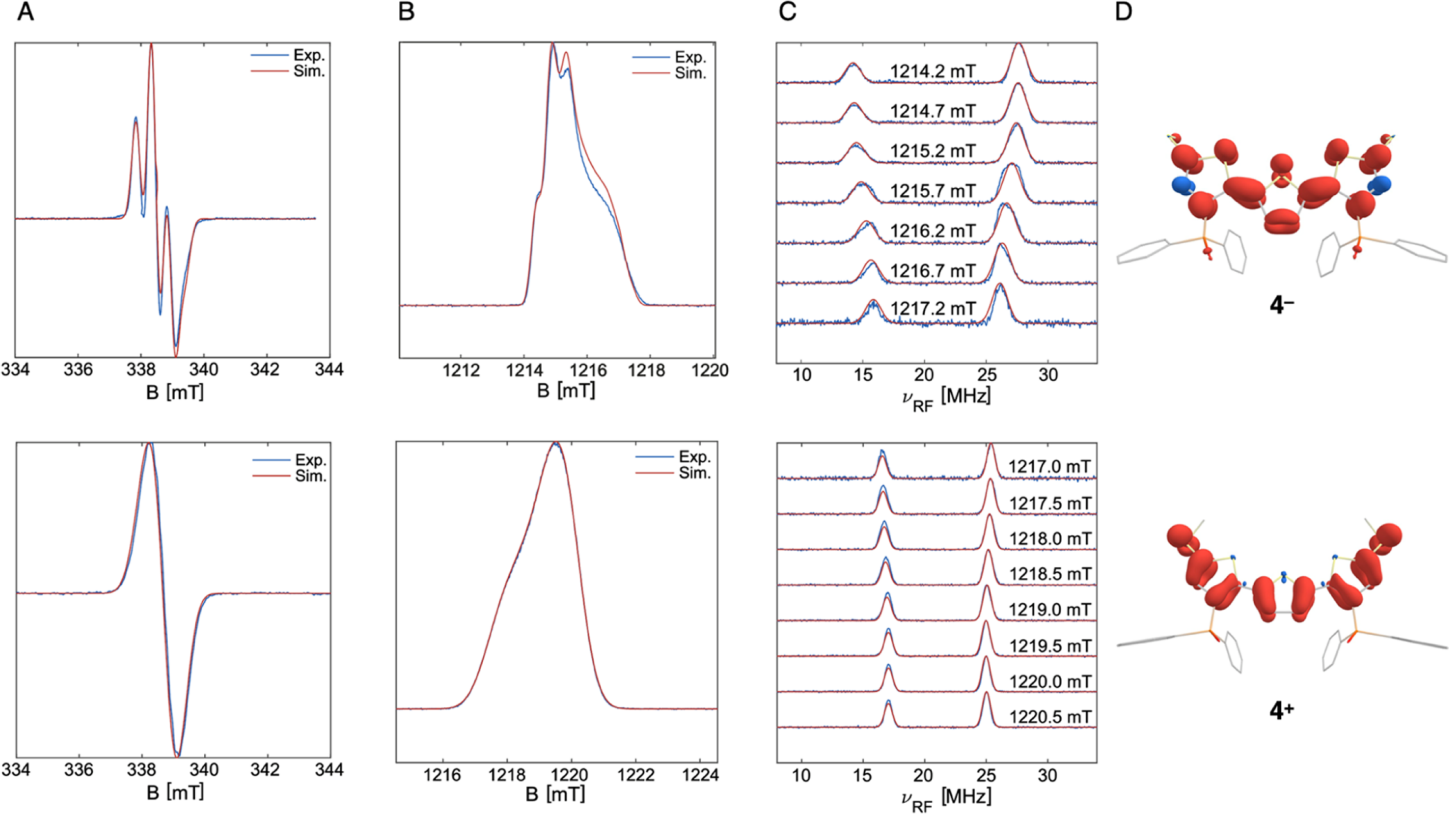
(A) X-band CW EPR spectra for **4**^**–**^ (top) and **4**^**+**^ (bottom) collected at 90 K. (B) Q-band echo-detected EPR spectra for **4**^**–**^ (top) and **4**^**+**^ (bottom) collected at 40 K. (C) Magnetic-field dependent ^31^P ENDOR for **4**^**–**^ (top) and **4**^**+**^ (bottom) collected at 40 K. (D) DFT-computed spin-density plot using contour levels of +0.25% (red), –0.25% (blue) for **4**^**–**^ (top) and **4**^**+**^ (bottom) (see SI for details).

The paramagnetism of **4**^**–**^ and **4**^**+**^ afforded the opportunity to probe the electronic structures of the compounds by magnetic measurements and electron paramagnetic resonance (EPR) spectroscopy. Solution-state magnetic moments of 1.91 ± 0.05 and 1.84 ± 0.04 μ_B_ (Evans method) were measured for **4**^**–**^ and **4**^**+**^, respectively, consistent with an *S* = 1/2 ground state for each. Shown in Figure 4A,B, continuous-wave (CW) EPR spectra at X- and Q-band frequencies were recorded at 90 and 40 K, respectively. These spectra exhibit anisotropic line shapes with hyperfine couplings (see below) and *g*-tensors of *g* = 2.0019 2.0058 2.0058 for **4**^**–**^ (2-Me-THF glass) and *g* = 2.0026 2.0034 2.0072 for **4**^**+**^ [DFB:toluene glass (3:7, *v*:*v*)]. The magnetic moments and observed *g*-values near the free electron value (*g*_e_ = 2.0023) support the formulation of both **4**^**–**^ and **4**^**+**^ as radical species, each with an unpaired electron delocalized across its TTH π-system. The minor *g*-anisotropy observed in each case (*g*_max_– *g*_min_ = 0.0039 for **4**^**–**^; *g*_max_ – *g*_min_ = 0.0046 for **4**^**+**^) suggests that the distribution of conformational isomers is limited for both compounds, likely due to their quinoidal electronic structures that restrict free rotation about the interthiophene C–C bonds. Interestingly, while hyperfine couplings to two equivalent phosphorus atoms are sufficiently large to show a line splitting in the X-band EPR spectrum of **4**^**–**^, a similar coupling was not resolved for **4**^**+**^. Q-band pulsed Davies electron and nuclear double resonance (ENDOR) spectroscopy was thus performed at 40 K to determine the magnitude of the ^31^P coupling constants and revealed a value of A(^31^P) = (9.90 13.50 13.50) MHz for **4**^**–**^ (Figure 4C, top). By contrast, a notably smaller coupling constant of A(^31^P) = (7.99 7.68 8.84) MHz was determined for **4**^**+**^ (Figure 4C, bottom). These data are consistent with the notion that the SOMO of **4**^**–**^ features a significant contribution from the Ph_2_P(O) functionality. To further support this electronic structure assignment, DFT-computed spin density analysis was carried out for **4**^**–**^ and **4**^**+**^. In addition to significant spin density delocalization across the TTH π-system, DFT computations show a contribution from the Ph_2_P(O) moieties without significant spin density on the SMe groups in **4**^**–**^ (Figure 4D, top). By contrast, substantial spin density is localized on the SMe functionalities in **4**^**+**^ with a reduced contribution from Ph_2_P(O) (Figure 4D, bottom). Thus, the combined magnetic, spectroscopic, and computational data underscore the necessity of the appended functionalities in stabilizing both reduced and oxidized TTH to achieve redox ambipolarity.

### Redox-Switchable PCET

**Scheme 5:**
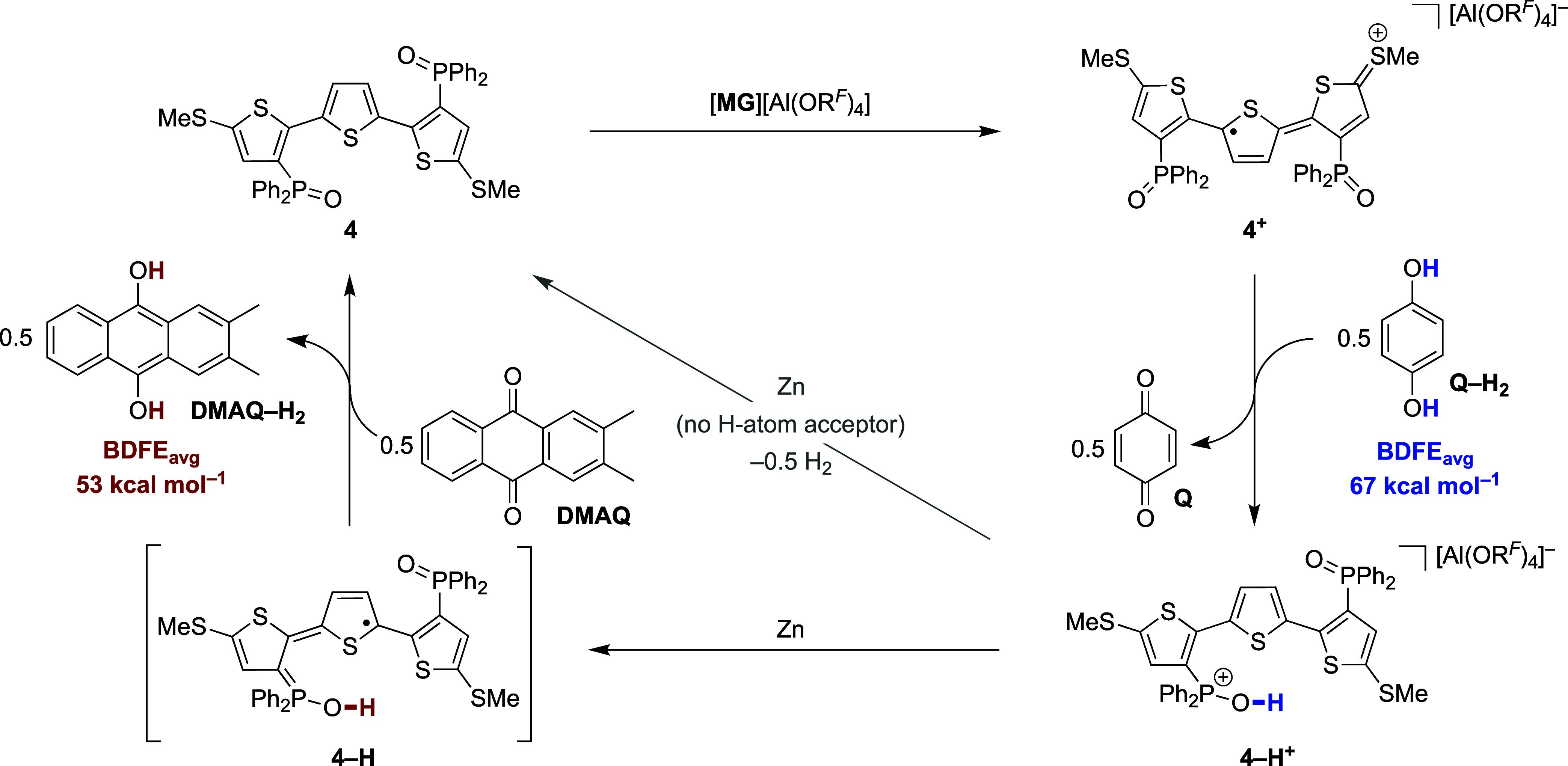
Redox-induced PCET between **4**^**+**^/**4–H** and Q–H_2_/DMAQ

Having established the electronic and structural features underpinning the redox ambipolarity of **4**, its application in a redox-switchable PCET scheme was targeted. To this end, **4**^**+**^ was generated and treated with a hydrogen atom donor to assess its ability to undergo a net PCET reaction. Accordingly, 1,4-dihydroquinone (**Q–H**_**2**_) was added to **4**^**+**^ at −35 °C in DFB solution that resulted in an immediate color change from deep turquoise to clear yellow. Analysis of the reaction mixture by ^1^H NMR spectroscopy revealed the formation of 1,4-benzoquinone (**Q**) together with a diamagnetic product assigned as the hydroxyphosphonium species **4–H**^**+**^ (Scheme 5, right). To confirm this assignment, **4–H**^**+**^ was prepared independently by protonation of **4** with [H(OEt_2_)_2_][BAr_4_^*F*^] (Ar^*F*^ = 3,5-(CF_3_)_2_-C_6_H_3_) (see SI for details). As in the case of **4**, the ^1^H NMR spectrum of **4–H**^**+**^ in CD_3_CN also features the number of resonances consistent with C_2v_ molecular symmetry in solution. However, the signal observed in the ^31^P{^1^H} NMR spectrum of **4–H**^**+**^ at 33.9 ppm is significantly more deshielded than the corresponding signal of **4** (^31^P{^1^H} δ = 18.8 ppm in CD_3_CN), consistent with the presence of an electron-withdrawing hydroxyphosphonium functionality.^16^ Upon addition of excess [H(OEt_2_)_2_][BAr_4_^*F*^] to **4–H**^**+**^, the ^31^P{^1^H} NMR signal shifts further downfield to δ = 45.5 ppm in CD_3_CN, confirming the availability of a second phosphine oxide moiety for protonation (Figure S8). Furthermore, the number of peaks in both the ^1^H and ^31^P{^1^H} NMR spectra of **4–H**^**+**^ remained unchanged upon cooling to −40 °C, suggestive of fast H^+^ exchange between the hydroxyphosphonium and phosphine oxide functionalities on the NMR time scale (Figure S7). Overall, these observations support PCET between **4**^**+**^ and **Q–H**_**2**_ to form **4–H**^**+**^. Given that the thermodynamics of PCET reactions are governed by the strengths of X–H bonds formed and cleaved, **Q–H**_**2**_ defines the lower bound for the O–H BDFE in **4–H**^**+**^ as 67 kcal mol^–1^ (Scheme 5, right).^11^

Noting that the cyclic voltammogram of **4** exhibits two successive one-electron oxidation events (see above), we investigated the possibility of generating dicationic **4**^**2+**^ and its competence in a PCET reaction. In this pursuit, we were cognizant of the fact that the second oxidation wave in the cyclic voltammogram of **4** exhibits quasi-reversible character, suggesting limited stability of a dicationic species (Figure S19). Indeed, treatment of **4** with 2 equiv of [**MG**][Al(OR^*F*^)_4_] oxidant at −35 °C in DFB solution resulted in the formation of an unstable, NMR-silent compound with an electronic absorption spectrum distinct from that observed for **4**^**+**^ (Figure S38). The rapid decomposition of this species precluded its isolation. Similarly, treatment of **4** with 2 equiv of [**MG**][Al(OR^*F*^)_4_] followed by the addition of **Q–H**_**2**_ led to significant TTH degradation as judged by ^1^H NMR spectroscopic analysis of the product mixture (Figure S4). These data point to the severe chemical instability of putative **4**^**2+**^ and further motivated the use of **4**^**+**^ in our desired switchable PCET scheme.

The moderate O–H BDFE in **4–H**^**+**^ (>67 kcal mol^–1^) led us to investigate its single-electron reduction with the goal of inducing O–H bond weakening. According to the Bordwell eq (eq 1), reduced species with negatively shifted *E°* values are expected to feature lower X–H BDFEs and should hence be effective H atom donors.^31^ A sufficiently weak O–H bond in the reduced product **4–H** would then enable its use as a hydrogen atom donor in a switchable PCET scheme (Scheme 1C). To aid our selection of appropriate reductant, the cyclic voltammogram of **4–H**^**+**^ was collected and revealed an irreversible cathodic feature with an inflection potential (*E*_i_)^26^ of −0.61 V. To investigate the nature of the observed electrochemical irreversibility, we treated **4–H**^**+**^ with excess Zn reductant and observed the clean formation of **4** alongside H_2_ gas over the course of 1 h at room temperature (Figure S9). From these results, we surmised that the product of single-electron reduction (**4–H**) likely features O–H BDFEs below the thermodynamic threshold for H_2_ evolution (Δ*G*_f_^°^(H^•^) = 52.0 kcal mol^–1^ in MeCN).^27^ The species **4–H** is thus assumed to be a highly potent H atom donor and we pursued its incorporation into a redox-switchable PCET scheme.

To demonstrate redox-induced PCET with **4–H**, we targeted the reduction of **4–H**^**+**^ in the presence of an appropriate H atom acceptor. To this end, **4–H**^**+**^ was treated with excess Zn powder in the presence of 2,3-dimethylanthraquinone (**DMAQ**). After 1 h of stirring at room temperature, the formation of **4** was observed alongside 2,3-dimethyl-9,10-dihydroxyanthracene (**DMAQ–H**_**2**_) in 88% yield (Scheme 5, left). Control experiments ruled out a reaction pathway initiated either by reduction of **DMAQ** by Zn or protonation of **DMAQ** by **4–H**^**+**^ and instead point to a PCET process from *in situ* generated **4–H** (see SI for details). The observed reactivity with **DMAQ** hence sets the upper bound for the O–H BDFE of **4–H** as 53 kcal mol^–1^.^28^ Importantly, the overall reaction sequence depicted in Scheme 5 represents net hydrogen atom transfer from **Q–H**_**2**_ to **DMAQ** using **4** as the mediator. This reaction is formally endergonic [BDFE_O–H_(**Q–H**_**2**_) > BDFE_O–H_(**DMAQ–H**_**2**_)] and establishes the utility of **4** as a single-component PCET reagent that can take up and release hydrogen atom equivalents upon a redox stimulus. This feature is especially noteworthy given that reductive (X–H bond forming) and oxidative (X–H bond cleaving) PCET typically require separate reagents.^11,29^ While PCET at phosphine oxides has been proposed to operate in catalytic processes,^30^ to our knowledge, **4** represents the first controlled example of net hydrogen atom transfer with this functionality.

**Scheme 6:**
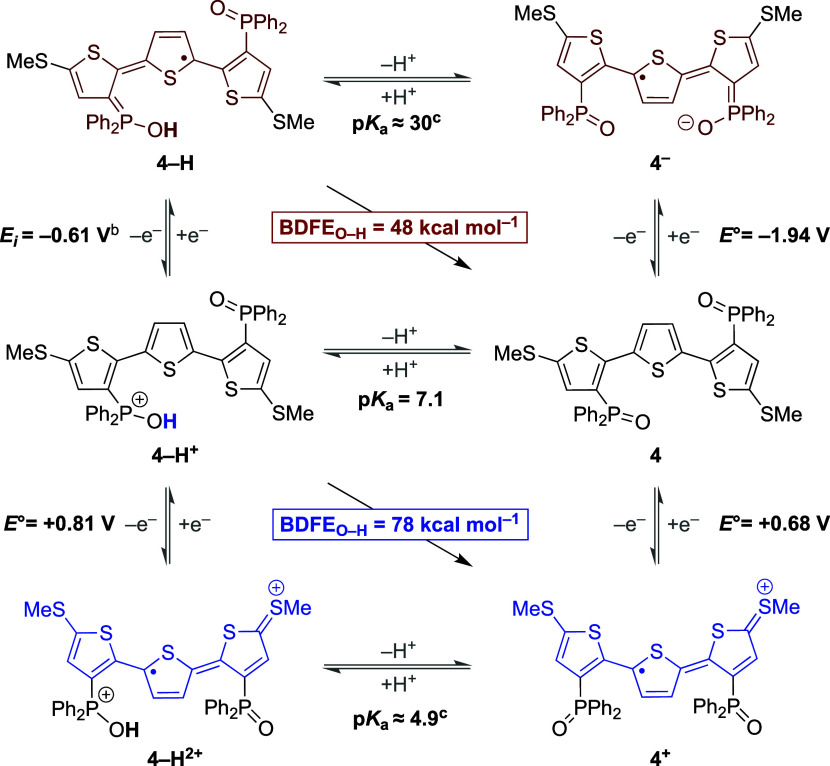
Square Schemes Containing Thermochemical Parameters Contributing to the BDFE_O–H_ in **4–H**^**+**^ and **4–H** All experimental measurements were conducted in MeCN solution. *E°* values are reported as half-wave potentials unless noted otherwise (1.0 mM concentration with 0.10 M [(*n*-Bu)_4_N][PF_6_] supporting electrolyte, 100 mV sec^–1^ scan rate, r.t., glassy carbon working electrode). Inflection potential of an irreversible cathodic wave. Calculated from Bordwell equation using experimentally determined values for BDFE_O–H_ and *E°* (see SI for details).

Having demonstrated the utility of **4** in a redox-switchable PCET scheme, we conducted thermochemical studies to quantify the strength of the O–H bond formed and cleaved during the process. We hence constructed thermochemical square schemes wherein O–H BDFEs of **4–H**^**+**^ and **4–H** are expressed using constituent proton and electron transfer steps (Scheme 6). The Bordwell eq (eq 1)^31^ then allows for the determination of relevant O–H BDFEs by summing experimentally measured p*K*_a_ and redox potential (*E°*) values together with the solvent-specific H^+^/H^•^ reduction potential (*C*_G_).
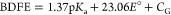
1

Critical for the experimental determination of the O–H BDFEs in both **4–H**^**+**^ and **4–H** is the measurement of the O–H p*K*_a_ in **4–H**^**+**^ (Scheme 6, middle row). This value was determined by ^31^P NMR spectroscopy using PPh_3_ as the reference base (p*K*_a_ = 7.6 in MeCN).^32^ Upon addition of PPh_3_ to **4–H**^**+**^, the equilibrium concentration relative to its conjugate base **4** was measured and yielded a p*K*_a_ of 7.1 in MeCN.^33^ In combination with the **4**^**+**^/**4** redox couple (*E°* = +0.68 V in MeCN), this value allowed determination of the O–H BDFE in **4–H**^**+**^ as 78 kcal mol^–1^. Analogously, the p*K*_a_ of **4–H**^**+**^ together with the experimentally determined value for the **4–H**^**+**^**/4–H** redox couple (*E*_i_ = −0.61 V) yielded an O–H BDFE of ca. 48 kcal mol^–1^ for **4–H**. These thermochemical studies thus establish a remarkable degree of redox-induced bond weakening (30 kcal mol^–1^) in **4–H** compared to **4–H**^**+**^. Our results show that, on the one hand, the relatively strong O–H bond of 78 kcal mol^–1^ in **4–H**^**+**^ drives PCET between **Q–H**_**2**_ and **4**^**+**^. Conversely, the O–H bond in **4–H** is sufficiently weak (BDFE_O–H_ = 48 kcal mol^–1^) to transfer a hydrogen atom equivalent to **DMAQ** or evolve H_2_ in the absence of a suitable reaction partner. It is important to note that during this reaction sequence, the TTH backbone is either formally reduced (**4–H**) or oxidized (**4**^**+**^) while a phosphine oxide functionality accepts a H^+^ equivalent. It is thus the unique interplay of the Ph_2_P(O) and SMe functionalities in simultaneously enabling *both* TTH ambipolarity *and* H^+^ transfer that allows for a controlled, redox-switchable PCET reactivity observed with **4**. Overall, these results illustrate that redox ambipolarity can be used to induce large changes in O–H BDFE, thereby enabling both hydrogen atom uptake and release at a single organic scaffold.

Besides quantifying the O–H BDFEs in **4–H**^**+**^ and **4–H**, the thermodynamic information presented in Scheme 6 can be used to draw mechanistic conclusions about the PCET reactions between **4**^**+**^ and **Q–H**_**2**_, as well as concerning the reaction involving **4–H** and **DMAQ** (Scheme 5). In general, three mechanistic extremes are feasible for PCET reactions: (1) initial proton transfer followed by electron transfer (PT-ET); (2) electron transfer followed by proton transfer (ET-PT) or (3) concerted proton–electron transfer (CPET).^11^ Comparing the relevant p*K*_a_/*E°* values of the reactants can indicate the thermodynamic feasibility of a stepwise pathway and hence provide mechanistic insights. Accordingly, estimation of the p*K*_a_ of **4**^**+**^ (≈ 4.9 in MeCN)^34^ rules out initial proton transfer (PT) from **Q–H**_**2**_ (p*K*_a_ ≈ 32 in MeCN)^35^ based on its endergonic nature (Δ*G°*_PT_ ≈ +37 kcal mol^–1^). On the other hand, experimental measurement of the reduction potential of **4**^**+**^ (*E°* = +0.68 V) indicates that a reaction pathway initiated by electron transfer (ET) from **Q–H**_**2**_ (*E*_i_ = +0.85 V in MeCN) is endergonic but thermodynamically feasible (Δ*G°*_ET_ ≈ +4.2 kcal mol^–1^). Hence, the reaction of **4**^**+**^ and **Q–H**_**2**_ may proceed via stepwise ET-PT, or by a concerted pathway (CPET; see Scheme S1). By stark contrast, both the redox potentials and p*K*_a_ values of **4–H** (p*K*_a_ ≈ 30; *E*_i_ = −0.61 V in MeCN) and **DMAQ** (p*K*_a_ < 0.1;^36^*E°* = −1.38 V in MeCN) are mismatched with Δ*G°*_PT_ ≳ +41 kcal mol^–1^ and Δ*G°*_ET_ ≈ +18 kcal mol^–1^. Based on these results, a CPET mechanism is likely favored in the reaction between **4–H** and **DMAQ** that avoids high-energy intermediates of stepwise ET-PT/PT-ET pathways (Scheme S2). Altogether, these results provide rare mechanistic insight into the PCET reactivity of an organic system that functions as both a hydrogen atom acceptor and donor upon application of a redox stimulus.

## Conclusions

In summary, a redox-ambipolar TTH bearing methyl thioether and phosphine oxide functionalities (**4**) was synthesized and its switchable PCET reactivity was demonstrated. The redox ambipolarity of **4** was established by electrochemical studies, which motivated the preparation of cationic (**4**^**+**^) and anionic (**4**^**–**^) radical species on a single TTH platform. A suite of characterization methods including single-crystal X-ray diffraction, UV-vis-NIR, electron paramagnetic resonance and electron–nuclear double resonance spectroscopy uncovered the unique electronic stabilization imparted by the appended functionalities that is critical for the observed redox ambipolarity. The dual reactivity of **4** as both a hydrogen atom acceptor and donor was demonstrated in a redox-switchable PCET scheme. In particular, a PCET reaction took place between **4** and 1,4-dihydroquinone (**Q–H**_**2**_) upon single-electron oxidation to generate the hydroxyphosphonium species **4–H**^**+**^. The PCET reactivity was reversed upon single-electron reduction, resulting in the transfer of a hydrogen atom equivalent from **4–H** to 2,3-dimethylanthraquinone (**DMAQ**). Electrochemical and p*K*_a_ measurements were carried out to construct a thermochemical square scheme that revealed significant redox-induced O–H bond weakening between **4–H**^**+**^/**4–H** (30 kcal mol^–1^) enabling the observed PCET reactivity. Examination of the relevant *E°* and p*K*_a_ values ruled out a PT-ET pathway in the case of **4**^**+**^ and **Q–H**_**2**_ while pointing to a CPET mechanism for the reaction of **4–H** with **DMAQ**.

Overall, the switchable PCET demonstrated herein offers new design principles for controlling the uptake and release of hydrogen atom equivalents at an ambipolar organic scaffold that may be generally applied across synthetic chemistry and catalysis.^8−10^ More broadly, besides the potential application of functionalized oligothiophenes as ambipolar electrolytes for energy storage,^3^ the radicals **4**^**+**^ and **4**^**–**^ can be viewed as charge carrier models for oxidized (p-type) and reduced (n-type) polythiophene, respectively.^37^ We hence envision that the spectroscopic features of **4**^**+**^ and **4**^**–**^ reported herein may improve the understanding of charge carrier dynamics in oligothiophene-based organic electronics.^38^ By connecting the concepts of redox ambipolarity and PCET, our study opens fundamentally new parameter space for proton and electron management in organic systems.

## Supporting Information Available

Experimental details; characterization data for all new compounds; details of thermochemical studies (p*K*_a_ and BDFE determinations); and computational data (PDF)
