# Supplementary material for: Functionalized Terthiophene as an Ambipolar Redox System: Structure, Spectroscopy, and Switchable Proton-Coupled Electron Transfer
Source: J Am Chem Soc. 2025 Jan 27;147(5):4493–503. doi: 10.1021/jacs.4c16003 (PMC11803731; doi:10.1021/jacs.4c16003)
Supplement: Supplementary file 1 — ja4c16003_si_001.pdf [file ja4c16003_si_001.pdf]

Supporting Information

Functionalized Terthiophene as an Ambipolar Redox System: Structure, Spectroscopy and Switchable Proton-Coupled Electron Transfer

Daniel Käch, Daniel Klose, Lionel Wettstein, and Máté J. Bezdek*

Department of Chemistry and Applied Biosciences

ETH Zürich

Vladimir-Prelog-Weg 1, 8093 Zürich (Switzerland)

*E-mail: mbezdek@ethz.ch

Table of Contents

I. Materials and Methods S2
II. Synthetic Protocols S5
III. Additional Reactions and Associated NMR Spectra S11
IV. pK_a Determinations S20
V. PCET Mechanism S23
VI. Electrochemical Experiments and Characterization S26
VII. Additional Electrochemical Data S38
VIII. Magnetic Moment Determination S40
IX. Photophysical Data S41
X. NMR Spectra S44
XI. EPR Data S63
XII. X-Ray Data S67
XIII. Computational Data S73
XIV. References S93

I. Materials and Methods

General Methods

All air- and moisture-sensitive manipulations were carried out using standard vacuum line Schlenk techniques or in an argon-filled MBraun LabMaster Pro glovebox. Reactions were carried out in oven-dried or flame-dried glassware equipped with a stirbar. Solvents were purified using a Innovative Technology PureSolv purification system and were degassed with purified argon. The water content was determined using a Mettler-Toledo C30 coulometric Karl-Fischer titrator. Solvents were stored over 3 Å or 4 Å molecular sieves. Chemicals were received from Sigma-Aldrich, Fluorochem, abcr, Acros, STREM and were used as received. 3,3'',5,5''-tetrabromo-2,2':5',2''-terthiophene[1], $[H(OEt_2)_2][BAr^F_4]$ (Ar^F = 3,5-$(CF_3)_2C_6H_3$) [2], (2,4-Br_2-Ph)$_3$N and [N(4-Br-C_6H_4)$_3$][$SbCl_6$][3], Li[Al(OC$(CF_3)_3$)$_4$][4], 3,3''-P(O)PPh_2 (**5**)[5], 5,5''-SMe-TTH (**6**)[6] and [(*n*-Bu)$_4$N][Al(OR^F)$_4$][7] were synthesized according to literature procedures. Basic alumina was purchased from MP Biomedicals and dried at 200 °C under high vacuum for 2 days before use. The exact concentration of *n*-BuLi in hexanes was determined by titration according to a literature procedure.[8] Solution NMR measurements were carried out on a Bruker Avance 11.7 T cryoprobe spectrometer. NMR spectra were referenced against residual protonated solvent (^{1}H & ^{13}C), $CFCl_3$ (^{19}F) and H_3PO_4 (^{31}P). Chemical shifts δ are given in ppm and the absolute values of the coupling constants are given in Hertz (Hz). Multiplicities are indicated by s (singlet), d (doublet), t (triplet), vt (virtual triplet), q (quartet), hept (heptet), m (multiplet), br (broad) and combinations thereof.

The X-band CW EPR experiments were performed on a Bruker ElexSys E500 CW EPR spectrometer (Bruker Biospin, Ettlingen, Germany), equipped with a Bruker Super-High-Q resonator and a Helium flow cryostat (ESR-900, Oxford Instruments, Oxfordshire, UK). For experiments at room temperature 20 μl of sample solution with a concentration of 1.0 mM were filled into quartz capillaries with an outer diameter of 1.6 mm in an Ar-filled glovebox. The capillaries were kept sealed in 4.0 mm J-Young quartz tubes and a gentle stream of nitrogen gas was used to maintain a stable temperature. The spectra were recorded with a microwave power of 13 μW to ensure non-saturating conditions, using 2048 points, a lock-in conversion time 81.92 ms, a time constant of 40.96 ms, with a 100 kHz magnetic field modulation of 0.0075 mT. The magnetic field offset was corrected using 2,2-diphenyl-1-picrylhydrazyl (DPPH, Merck, Buchs, Switzerland) as a reference sample.[9] For experiments at cryogenic temperatures 40 μl of 1 mM sample solutions were frozen in liquid N_2 and flame-sealed into quartz tubes with an outer diameter of 3.0 mm. Under cryogenic conditions, CW EPR spectra were recorded with a microwave power of 8 μW to ensure non-saturating conditions, using 1024 points, a lock-in conversion time 81.92 ms, a time constant of 40.96 ms, with a 100 kHz magnetic field modulation of 0.05 mT. The magnetic field offset was corrected using 2,2-diphenyl-1-picrylhydrazyl (DPPH, Merck, Buchs, Switzerland) as a reference sample.[9]

Pulse EPR experiments were performed at Q-band frequencies (ca. 34-34.5 GHz) on a home-built EPR spectrometer[10], equipped with a home-built ENDOR resonator[11] with 3.0 mm sample access, a Helium flow cryostat (CF935, Oxford Instruments, Oxfordshire, UK), a TWT amplifier with a nominal power of 150 W (Applied Systems Engineering Inc., Texas, USA), and a 500 W radiofrequency amplifier (Amplifier Research, Souderton, PA, USA) connected to the ENDOR coils. Echo-detected EPR spectra were recorded with the Hahn echo sequence, $\pi/2$ - τ - π - τ - echo, with a long interpulse delay τ of 1 – 1.4 μs to avoid distortions by nuclear modulations (except for [**MG**][Al(OR^F)$_4$], where τ was set to 600 ns), 16/32 ns rectangular pulses for $\pi/2$ and π, respectively. The echo was integrated with a boxcar integration window of 200 ns. Data were acquired by stepping the magnetic field in 501 points, using 20 shots per

points with a shot repetition time of 20 ms. Davies ENDOR experiments were acquired using the standard pulse sequence[12]. ^{1}H ENDOR spectra were acquired at the magnetic field corresponding to the maximum echo intensity with 241 points (40-64 MHz), using a selective microwave π-pulse of 256 ns and observer pulses of 100 and 200 ns for π/2 and π, respectively, with an interpulse delay of 600 ns. The full echo width was integrated with a boxcar window of 800 ns. The radiofrequency (RF) pulse length was set to 10 μs at 10 dB RF source attenuation to avoid amplifier overtones. The ENDOR spectra were samples in a stochastic manner[13] and with a two-step phase cycle (+x - -x) on the π/2-observer pulse to cancel out receiver offsets. For the ^{31}P Davies ENDOR experiments, spectra were acquired instead with 521 points (8-34 MHz) and 20 μs RF pulse length. The HYSCORE spectrum was recorded using the standard 4-pulse sequence[14] with 256 points, using 16 and 20 ns pulses for π/2 and π, respectively, with time steps of 12 ns in both dimensions, 128 shots per point and a shot repetition time of 0.2 ms.

Data processing and simulations were performed using Matlab (The Mathworks, Natick, USA) with the toolboxes EasySpin 6.0.6[15] and Hyscorean[16].

Single crystals suitable for X-ray diffraction were coated with polyisobutenes in a glovebox, transferred onto a nylon loop and mounted on the goniometer of a Rigaku XtaLab Synergy equipped with a molybdenum X-ray tube (λ = 0.71073 Å) and a copper X-ray tube (λ = 1.5406 Å). The structures were solved using direct methods (SHELXT) in the program OLEX2, then completed by Fourier transformation and finally refined by full-matrix least-squares procedures.[17,18] UV-Vis-NIR spectra were recorded on an Agilent Cary5000. Emission spectra were recorded on a Cary Eclipse Fluorescence Spectrophotometer. FT-IR spectra were collected in the solid state with the ATR technique on a Bruker Alpha inside an MBraun glovebox or a Bruker Tensor II under air. Flash column chromatography was performed on a Biotage Isolera One system with Sfär columns using technical grade solvents. High-resolution mass spectra were provided by the mass spectrometry service facility in the Laboratories of Organic Chemistry at ETH Zürich. The molecular ions $[M]^+$ and $[M+H]^+$ are given in m/z units. NMR spectra are assigned according to the general numbering scheme for the terthiophene (TTH) backbone (Figure S1).

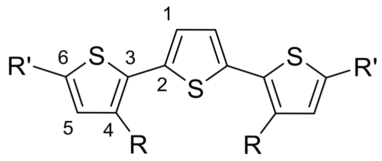

Figure S1. General Numbering Scheme for the TTH backbone.

Electrochemical Experiments and Characterization

General Methods: Purified dimethoxyethane (DME) and electrochemical-grade acetonitrile (MeCN) purchased from Sigma-Aldrich were stored over 3 Å molecular sieves and filtered over a short pad of basic alumina prior to use. $[(n\text{-Bu})_4N][PF_6]$ was purchased from Fluorochem, recrystallized twice from hot ethanol (EtOH) and dried under high vacuum for 48 h at 80 °C. $[(n\text{-Bu})_4N][Al(OR^F)_4]$ was dried under high vacuum for 24 h at 60 °C.

Cyclic Voltammetry: Cyclic Voltammetry (CV) was performed in an argon-filled MBraun UniLab glovebox with a Gamry Interface 1010E potentiostat/galvanostat/ZRA using a three-electrode electrochemical cell. If not stated otherwise, a $Ag/AgNO_3$ (0.010 M in $[(n\text{-Bu})_4N][PF_6]$ in MeCN, BASi), glassy carbon disk (1.6 mm^2, eDAQ) and Pt-wire were used as reference, working and counter electrodes, respectively. The working electrode was polished before each

experiment on a pad using an alox-slurry (0.050 mm) and rinsed sequentially with millipore water, isopropanol (^{i}PrOH), and acetone. Experiments were conducted using 0.10 M [(*n*-Bu)$_4$N][PF$_6$] or [(*n*-Bu)$_4$N][Al(OR^F)$_4$] as supporting electrolyte in DME, MeCN or 1,2-difluorobenzene (DFB). First, a background scan of a blank sample containing electrolyte and solvent was performed to determine the solvent window and ensure a stable potential and the absence of contaminants. Next, the respective compound (1.0 mM) was dissolved in the blank sample and two scans of the full window starting at the open circuit potential (OCP) were recorded. The first scan is reported. CVs were internally referenced against the Fc/Fc$^+$ couple. If necessary, data smoothening using the Savitzky–Golay method was applied. The redox potential of irreversible oxidation and reduction events was determined by taking the inflection point.[19]

Electrosynthesis: Chronoamperometry experiments were performed in a custom-made H-cell in an argon-filled glovebox, adapted from Sanford and coworkers (Figure S2).[20] The cell chambers were separated by a P5-frit (10 mm diameter, porosity 5 from Robu®). We employed a cleaning procedure wherein the cell was sonicated 15 min with EtOH, soaked in water, kept overnight in concentrated nitric acid, soaked in water again, and washed with EtOH. Prior to use, the cell was oven-dried overnight at 180 °C. Reticulated vitreous carbon (RVC) was purchased from Goodfellow (40 pores/cm) and cut into rods of the dimension 0.5 cm x 0.5 cm x 4.0 cm. The RVC rods were connected to a Au wire and positioned ca. 1.5 cm deep in solution. The RVC rods were single use and were dried for 16 h at 80 °C under reduced pressure prior to use. The reference electrode was a Ag/AgNO$_3$ reference containing 0.010 M AgNO$_3$ in 0.10 M [(*n*-Bu)$_4$N][PF$_6$] in MeCN or DME.

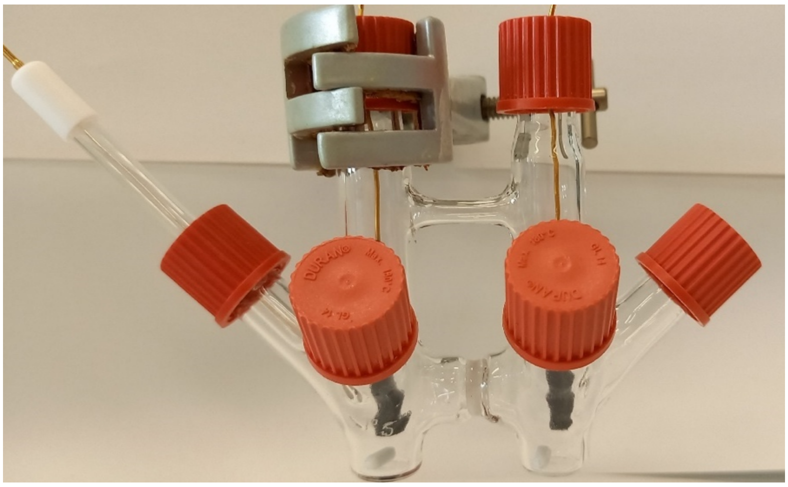

Figure S2. Picture of the assembled H-cell.

II. Synthetic Protocols

Synthesis of **2**

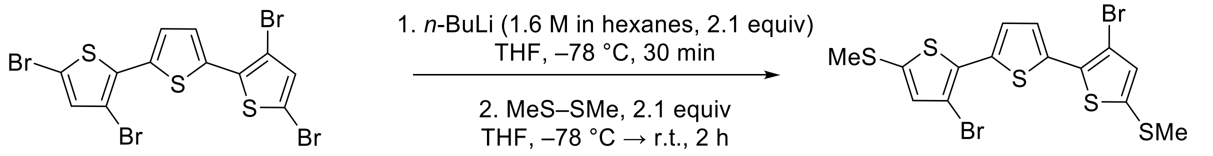

1 **2**

A 250 mL oven-dried two-neck round-bottom flask was charged with a magnetic stir bar and **1** (2.5 g, 4.4 mmol, 1.0 equiv). After flushing the flask headspace with argon, the yellow solid was dissolved in dry THF (75 mL). The resulting dark-yellow solution was cooled to –78 °C using a dry ice/acetone cooling bath. Then, *n*-BuLi (5.8 mL, 1.6 M in hexanes, 2.1 equiv) was added dropwise. After stirring for 30 minutes at –78 °C, dimethyl disulfide (0.83 mL, 9.3 mmol, 2.1 equiv) was added dropwise. After stirring for 30 minutes at –78 °C, the reaction mixture was warmed to room temperature and stirred overnight. After drying *in vacuo*, CH_2Cl_2 (50 mL) was added and washed with brine (3 x 30 mL). After extraction of the aqueous phase with CH_2Cl_2, the organic phases were combined, dried over $MgSO_4$, filtered, and dried *in vacuo*. Flash column chromatography using *n*-hexane:EtOAc (90:10, v:v) was followed by recrystallization from *n*-hexane to obtain the product as a yellow solid (1.95 g, 3.9 mmol, 88%).

^{1}H NMR (500 MHz, $CDCl_3$, 298 K): δ 7.31 (s, 2H, ***H1***), 6.95 (s, 2H, ***H5***), 2.53 (s, 6H, SC***H***$_3$).

^{13}C{^{1}H} NMR (126 MHz, $CDCl_3$, 298 K): δ 137.4 (***C6***), 134.7 (***C3***), 134.1 (***C5***), 133.3 (***C2***), 126.7 (***C1***), 107.4 (***C4***), 21.4 (S***C***H$_3$).

IR [cm^{-1}]: ṽ = 3086, 3060, 2983, 2913, 2845, 2818, 1751, 1650, 1579, 1540, 1511, 1482, 1431, 1412, 1328, 1313, 1302, 1274, 1130, 1083, 995, 979, 963, 879, 853, 825, 783.

HRMS: calc. [M]$^+$ $C_{14}H_{10}Br_2S_5$ 495.7747; found 495.7747.

UV-VIS-NIR: λ_{max} (MeCN): 381 nm (ε = 2.9 × 10^4 M^{-1} cm^{-1}).

Synthesis of **3**

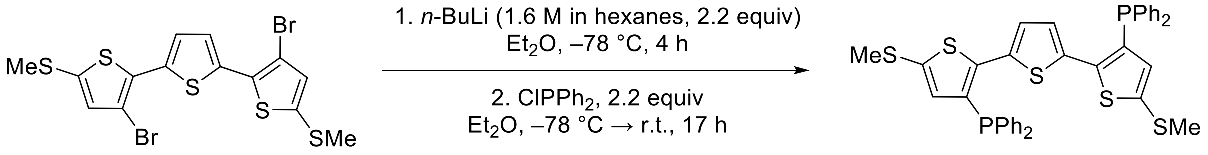

2 **3**

A 250 mL oven-dried two-neck round-bottom flask was charged with a magnetic stir bar and **2** (1.85 g, 3.7 mmol, 1.0 equiv). After flushing the flask headspace with argon, the yellow solid was dissolved in dry Et_2O (85 mL) and the resulting yellow solution was cooled to –78 °C using a dry ice/acetone cooling bath. Then, *n*-BuLi (5.1 mL, 1.6 M in hexanes, 2.2 equiv) was added dropwise. The reaction mixture was stirred for 4 hours at –78 °C. Afterwards, chlorodiphenylphosphine (1.47 mL, 8.2 mmol, 2.2 equiv) was added. After stirring for 1 hour at –78 °C, the reaction mixture was warmed to room temperature and stirred overnight. The resulting yellow suspension was quenched with H_2O (30 mL). Vacuum filtration afforded the product as a yellow solid (2.13 g, 3.0 mmol, 81%).

^{1}H NMR (500 MHz, $CDCl_3$, 298 K): δ 7.35 – 7.29 (m, 20H, PC$_6$***H***$_5$), 7.00 (s, 2H, ***H1***), 6.48 (d, 2H, $^3J_{PH}$ = 0.8 Hz, ***H5***), 2.44 (s, 6H, SC***H***$_3$).

$^{31}P\{^1H\}$ NMR (202 MHz, $CDCl_3$, 298 K): δ –24.4.

$^{13}C\{^1H\}$ NMR (126 MHz, $CDCl_3$, 298 K): δ 144.1 (d, $^2J_{CP}$ = 33.9 Hz, ***C3***), 137.4 (d, $^1J_{CP}$ = 10.4 Hz, P***C***$_6$H$_5$), 136.7 (d, $^1J_{CP}$ = 5.9 Hz, ***C4***),136.5 (***C6***), 136.0 (d, $^2J_{CP}$ = 1.9 Hz, ***C5***), 133.6 (d, $^3J_{CP}$ = 21.8 Hz, ***C2***), 133.5 (d, $^2J_{CP}$ = 19.5 Hz, P***C***$_6$H$_5$), 128.7 (P***C***$_6$H$_5$), 128.8 (d, $^3J_{CP}$ = 6.8 Hz, P***C***$_6$H$_5$), 127.5 ($^4J_{CP}$ = 6.1 Hz, ***C1***), 21.6 (S***C***H$_3$).

IR [cm^{-1}]: $\tilde{v}$ = 3066, 2996, 2920, 1583, 1568, 1471, 1431, 1412, 1390, 1308, 1274, 1231, 1201, 1182, 1137, 1091, 1067, 1025, 997, 965, 923, 878, 856, 829, 797, 745, 694.

HRMS: calc. $[M+H]^+$ $C_{38}H_{31}P_2S_5$ 709.0499; found 709.0492.

UV-VIS-NIR: λ_{max} (MeCN): 393 nm (ε = 1.8 × 10^4 M^{-1} cm^{-1}).

Synthesis of **4**

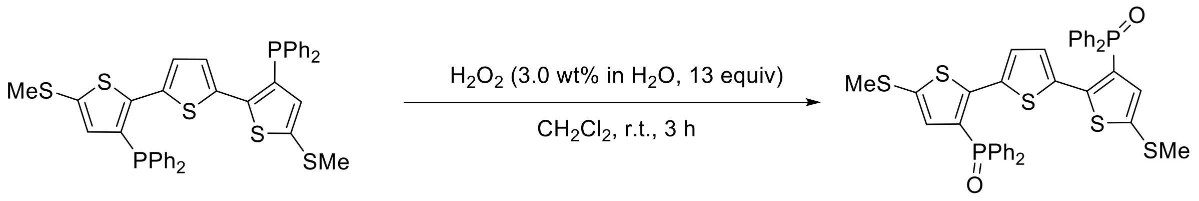

3 **4**

Under air, a 250 mL round-bottom flask was charged with a magnetic stir bar and **3** (2.08 g, 2.9 mmol, 1.0 equiv) dissolved in CH_2Cl_2 (85 mL). To the stirring solution, hydrogen peroxide (3.0 wt% in H_2O, 42.5 mL, 37.6 mmol, 13 equiv) was added. The biphasic solution was then stirred for 3 h at room temperature. Deionized water (3 x 50 mL) was added, and the phases were separated. The aqueous phase was extracted with CH_2Cl_2 (2 x 30 mL). The combined organic phases were dried over $MgSO_4$, filtered and dried *in vacuo*. Recrystallization by slow diffusion of *n*-hexane into a concentrated THF solution at –20 °C afforded the product as a yellow solid (1.65 g, 2.2 mmol, 76%). Single crystals suitable for X-ray diffraction were obtained from a concentrated MeCN solution of **4** at room temperature.

^1H NMR (500 MHz, $CDCl_3$, 298 K): δ 7.61 (m, 8H, P(O)C$_6$***H***$_5$), 7.46 (m, 4H, P(O)C$_6$***H***$_5$), 7.37 (m, 8H, P(O)C$_6$***H***$_5$), 7.00 (s, 2H, ***H1***), 6.62 (d, 2H, $^3J_{PH}$ = 4.7 Hz, ***H5***), 2.45 (s, 6H, SC***H***$_3$).

$^{31}P\{^1H\}$ NMR (202 MHz, $CDCl_3$, 298 K): δ 21.2.

$^{13}C\{^1H\}$ NMR (126 MHz, $CDCl_3$, 298 K): δ 146.4 (d, $^2J_{CP}$ = 10 Hz, ***C3***), 137.9 (d, $^3J_{CP}$ = 16.6 Hz, ***C6***), 136.0 (d, $^2J_{CP}$ = 15.9 Hz, ***C5***), 135.4 (d, $^3J_{CP}$ = 3.1 Hz, ***C2***), 132.3 (d, $^1J_{CP}$ = 107.5 Hz, P(O)***C***$_6$H$_5$), 132.1 (d, $^4J_{CP}$ = 2.8 Hz, P(O)***C***$_6$H$_5$), 131.7 (d, $^3J_{CP}$ = 10.0 Hz, P(O)***C***$_6$H$_5$), 130.6 (***C1***), 130.5 (d, $^1J_{CP}$ = 102.6 Hz, ***C4***), 128.7 (d, $^2J_{CP}$ = 12.6 Hz, P(O)***C***$_6$H$_5$), 21.8 (S***C***H$_3$).

IR [cm^{-1}]: $\tilde{v}$ = 3085, 3051, 2971, 2917, 2850, 1589, 1533, 1476, 1435, 1405, 1313, 1234, 1187, 1147, 1116, 1101, 1063, 1027, 997, 972, 923, 902, 886, 852, 754, 721, 691.

HRMS: calc. $[M+H]^+$ $C_{38}H_{31}O_2P_2S_5$ 741.0397; found 741.0393.

UV-VIS-NIR: λ_{max} (DME): 385 nm (ε = 2.2 × 10^4 M^{-1} cm^{-1}), λ_{max} (MeCN): 381 nm (ε = 1.8 × 10^4 M^{-1} cm^{-1}), λ_{max} (DFB): 381 nm (ε = 2.0 × 10^4 M^{-1} cm^{-1}).

Synthesis of **[K(THF)$_2$(4)]$_2$**

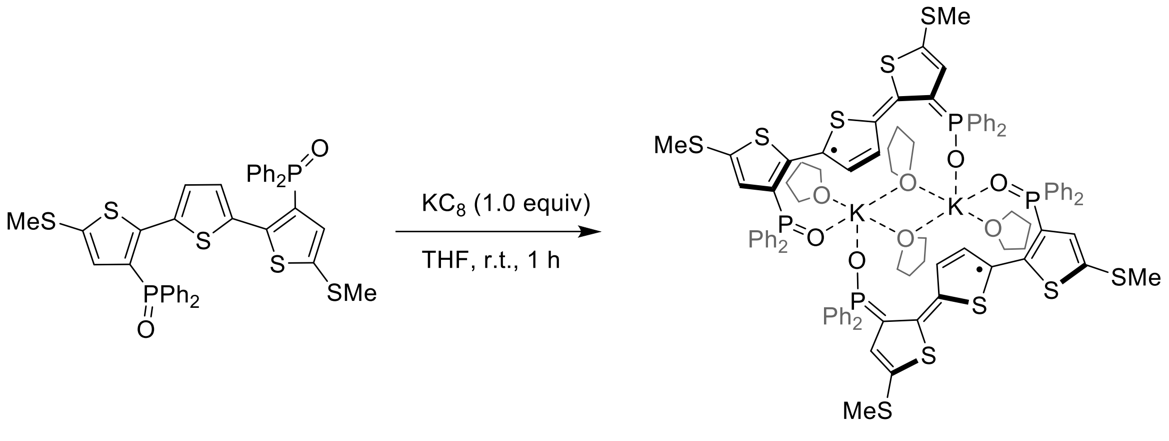

4 **[K(THF)$_2$(4)]$_2$**

In an argon-filled glovebox, a 5 mL scintillation vial was charged with a magnetic stir bar, **4** (50.0 mg, 0.068 mmol, 1.0 equiv) and KC_8 (9.1 mg, 0.068 mmol, 1.0 equiv). Then, THF (5 mL) was added, immediately giving a dark-blue suspension. After stirring for 1 hour at room temperature, the dark-blue suspension was filtered and the filtrate was dried *in vacuo* to afford the product as a dark-blue crystalline solid (57.0 mg, 0.061 mmol, 90%). Single crystals suitable for X-ray diffraction were grown from a concentrated THF solution of **[K(THF)$_2$(4)]$_2$** at –34 °C.

EPR (2-Me-THF, 90 K): g = 2.0017 2.0056 2.0056, A(^{31}P) = (10.10 13.83 13.83) MHz.

IR [cm^{-1}]: $\tilde{\nu}$ = 3049, 2956, 2912, 2851, 1588, 1572, 1464, 1434, 1361, 1302, 1243, 1218, 1168, 1116, 1095, 1065, 1026, 978, 958, 908, 834, 818, 746, 719, 692.

HRMS: calc. [M]$^+$ $C_{38}H_{31}KO_2P_2S_5$ 778.9956; found 778.9955.

UV-VIS-NIR: λ_{max} (DME): 378 nm ($\varepsilon = 1.4 \times 10^4$ M^{-1} cm^{-1}), 661 nm ($\varepsilon = 2.9 \times 10^4$ M^{-1} cm^{-1}), 1024 nm ($\varepsilon = 1.0 \times 10^4$ M^{-1} cm^{-1}), 1142 nm ($\varepsilon = 0.6 \times 10^4$ M^{-1} cm^{-1}).

Electrosynthesis of **4$^-$**

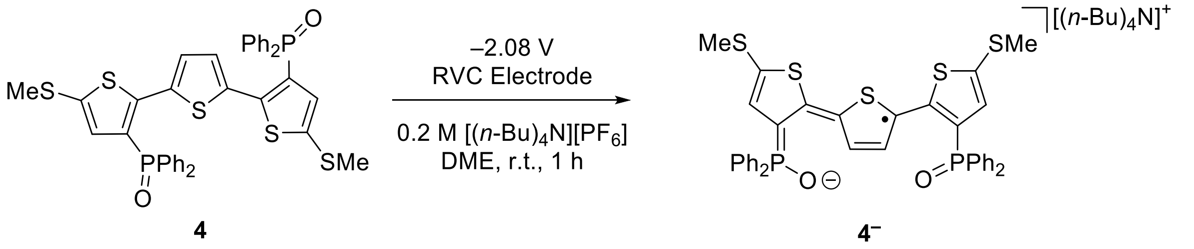

4 **4$^-$**

Two scintillation vials were each charged with [(n-Bu)$_4$N][PF$_6$] (504 mg, 1.3 mmol). Then, into each scintillation vial, DME (6.5 mL) was added after filtration through a short pad of alumina. Upon complete dissolution of [(n-Bu)$_4$N][PF$_6$], **4** (24.1 mg, 0.033 mmol) was added to one of the solutions. The solution containing only [(n-Bu)$_4$N][PF$_6$] in DME was then transferred into the counter side of an H-cell (see above) while the DME solution containing **4** and [(n-Bu)$_4$N][PF$_6$] was added into the working side. Two RVC electrode rods of the dimension 0.5 cm x 0.5 cm x 4.0 cm fixed by Au wires were then inserted into each chamber and placed 1.5 cm deep into the solution. The reaction mixture was electrolyzed at a constant potential of –2.08 V vs Fc/Fc$^+$ under stirring at 500 rpm and stopped after 1 hour. Single crystals suitable

for X-ray diffraction were obtained by slow diffusion of *n*-hexane into a DME solution at room temperature.

EPR (2-Me-THF, 90 K): *g* = 2.0019 2.0058 2.0058, A(^{31}P) = (9.90 13.50 13.50) MHz.

UV-VIS-NIR: λ_{max} (DFB): 369 nm, 664 nm, 1023 nm.

Synthesis of [**MG**][Al(OR^F)$_4$]

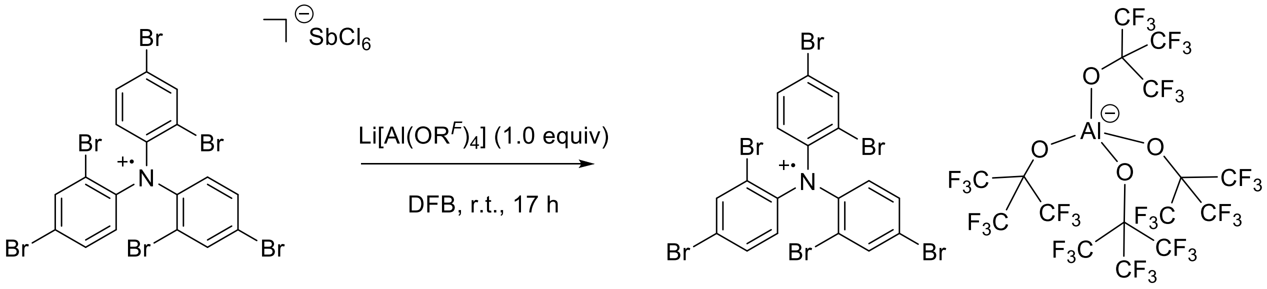

A procedure was adapted from the literature describing the synthesis of the "Magic Blue" analogue [N(4-Br-C_6H_4)$_3$][Al(OR^F)$_4$].[21]

In an argon-filled glovebox, a 5 mL scintillation vial was charged with a magnetic stir bar, [**MG**][$SbCl_6$] (49.2 mg, 0.047 mmol, 1.0 equiv) and Li[Al(OR^F)$_4$] (45.5 mg, 0.047 mmol, 1.0 equiv). Then, DFB (2 mL) was added giving a dark-green solution. After stirring for 17 hours at room temperature, the formation of a white precipitate was observed. The suspension was then filtered and the dark-green filtrate was dried *in vacuo* to afford the product as a dark-green solid (74.3 mg, 0.044 mmol, 94%).

EPR (toluene, 90 K): g = 2.0009 2.0145 2.0190, A(^{14}N) ca. 20 MHz (distributed).

^{19}F{^{1}H} NMR (471 MHz, CD_3CN, 298 K): δ -76.0.

^{27}Al NMR (130 MHz, CD_3CN, 298 K): δ -34.5.

IR[cm^{-1}]: 6121, 3089, 1542, 1523, 1453, 1442, 1378, 1352, 1296, 1264, 1238, 1209, 1166, 1144, 1068, 1041, 968, 918, 881, 832, 815, 783, 740, 725, 691.

HRMS: Positive mode: calc. [M]$^+$ $C_{18}H_9Br_6N$ 712.5830; found 712.5848. Negative mode: calc. [M]$^-$ $C_{16}AlF_{36}O_4$ 966.9043; found 966.9041.

UV-VIS-NIR: λ_{max} (MeCN): 290 nm (ε = 1.3 × 10^4 M^{-1} cm^{-1}), 371 nm (ε = 1.7 × 10^4 M^{-1} cm^{-1}), 859 nm (ε = 1.1 × 10^4 M^{-1} cm^{-1}).

Generation of 4⁺

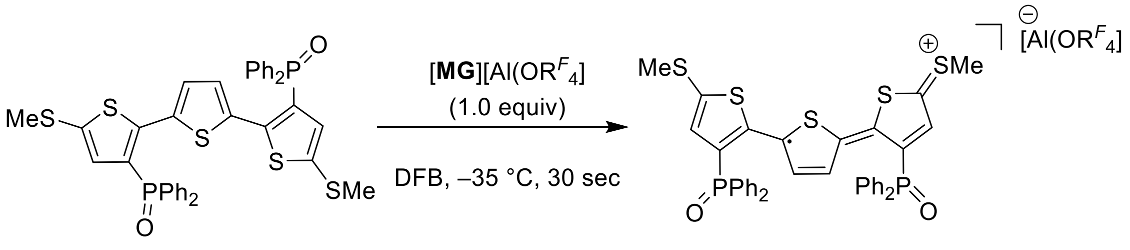

In an argon-filled glovebox, a 5 mL scintillation vial was charged with a magnetic stir bar, **4** (5.0 mg, 0.007 mmol, 1.0 equiv) and [**MG**][Al(OR^F)$_4$] (11.4 mg, 0.007 mmol, 1.0 equiv). Then, cold DFB (0.5 mL) was added at –35 °C, immediately giving a dark-turquoise suspension. After manually swirling the vial for 30 seconds, a dark-turquoise solution is obtained.

^{19}F{^{1}H} NMR (471 MHz, CD_3CN, 298 K): δ -76.0.

^{27}Al NMR (130 MHz, CD_3CN, 298 K): δ -34.6.

EPR (DFB:toluene 3:7, 90 K): g = 2.0026 2.0034 2.0072, A(^{31}P) = (7.99 7.68 8.84) MHz

UV-VIS-NIR: λ_{max} (DFB): 370 nm, 695 nm, 1200 nm.

Independent Synthesis of 4–H⁺

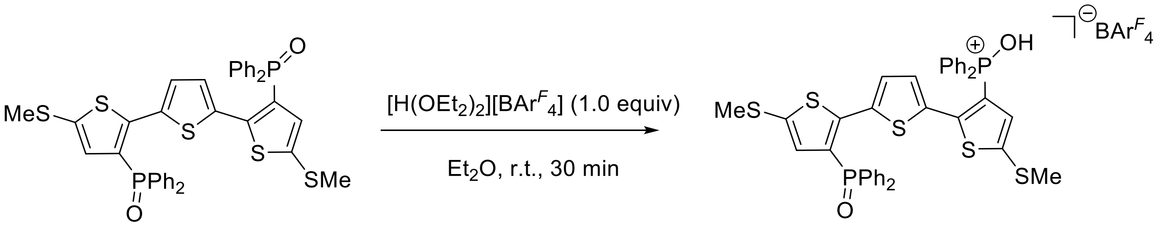

In an argon-filled glovebox, a 5 mL scintillation vial was charged with a magnetic stir bar, **4** (20.0 mg, 0.027 mmol, 1.0 equiv) and [H(OEt_2)$_2$][BAr$^F{}_4$] (27.3 mg, 0.027 mmol, 1.0 equiv). Then, Et_2O (0.5 mL) was added to give a yellow suspension. After stirring for 30 minutes at room temperature, the dark-yellow solution was dried *in vacuo* to afford the product as a yellow solid (41.4 mg, 0.026 mmol, 96%).

^{1}H NMR (500 MHz, CD_3CN, 298 K): δ 7.71-7.69 (m, 12H, B[(3,5-(CF_3)$_2$)C_6***H***$_3$]$_4$, overlap with P(O)C_6***H***$_5$), 7.66 (s, 4H, B[(3,5-(CF_3)$_2$)C_6***H***$_3$]$_4$), 7.55-7.50 (m, 16H, P(O)C_6***H***$_5$), 6.71 (s, 2H, ***H1***), 6.60 (d, 2H, $^3J_{PH}$ = 4.9 Hz, ***H5***), 2.45 (s, 6H, SC***H***$_3$).

^{19}F{^{1}H} NMR (471 MHz, CD_3CN, 298 K): δ -63.3.

^{31}P{^{1}H} NMR (202 MHz, CD_3CN, 298 K): δ 33.9.

^{11}B NMR (160 MHz, CD_3CN, 298 K): δ -6.7.

^{13}C{^{1}H} NMR (126 MHz, CD_3CN, 298 K): δ 162.6 (q, $^2J_{CF}$ = 49.8 Hz, B[(3,5-(CF_3)$_2$)***C***$_6$H$_3$]$_4$), 144.8 (d, $^2J_{CP}$ = 10.4 Hz, ***C3***), 142.8 (d, $^3J_{CP}$ = 18.6 Hz, ***C6***), 135.9 (d, $^3J_{CP}$ = 3.3 Hz, ***C2***), 135.7 (B[(3,5-(CF_3)$_2$)***C***$_6$H$_3$]$_4$), 135.3 (d, $^4J_{CP}$ = 3.1 Hz, P(O)***C***$_6$H$_5$), 134.4 (d, $^2J_{CP}$ = 18.7 Hz, ***C5***), 132.7 (d, $^3J_{CP}$ = 11.5 Hz, P(O)***C***$_6$H$_5$), 131.4 (***C1***), 130.5 (d, $^2J_{CP}$ = 13.5 Hz, P(O)***C***$_6$H$_5$), 129.9 (qq, J_{CF} = 2.8, 31.7 Hz, B[(3,5-(CF_3)$_2$)***C***$_6$H$_3$]$_4$), 127.1 (d, $^1J_{CP}$ = 111.9 Hz, P(O)***C***$_6$H$_5$), 125.5 (q, $^1J_{CF}$ =

271.7 Hz, B[(3,5-(***C***F$_3$)$_2$)C$_6$H$_3$]$_4$), 125.3 (d, $^1J_{CP}$ = 108.5 Hz, ***C4***), 118.7 (hept, $^3J_{CF}$ = 4.1 Hz, B[(3,5-(CF$_3$)$_2$)***C***$_6$H$_3$]$_4$), 21.0 (S***C***H$_3$).

IR [cm^{-1}]: ṽ = 3066, 2930, 1610, 1592, 1479, 1439, 1418, 1353, 1272, 1114, 1030, 1002, 929, 884, 838, 746, 730, 712, 690.

HRMS: Positive mode: calc. [M]$^+$ $C_{38}H_{31}O_2P_2S_5$ 741.0397; found 741.0392. Negative mode: calc. [M]$^-$ $C_{32}H_{12}BF_{24}$ 863.0654; found 863.0651.

UV-VIS-NIR: λ_{max} (MeCN): 370 nm (ε = 1.2 × 10^4 M^{-1} cm^{-1}).

Generation of **4–H$_2^{2+}$**

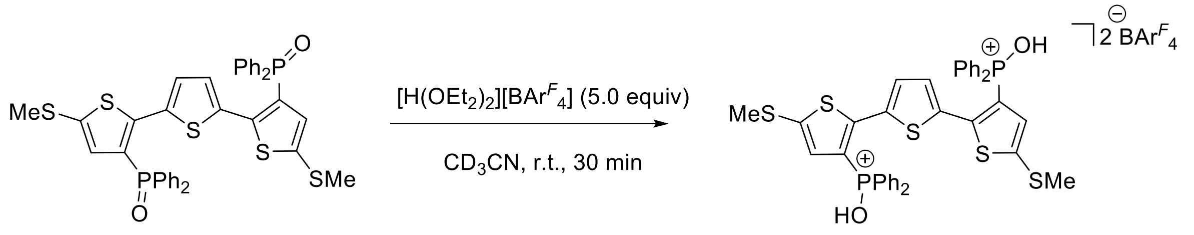

In an argon-filled glovebox, a 5 mL scintillation vial was charged with a magnetic stir bar, **4** (10.0 mg, 0.014 mmol, 1.0 equiv) and [H(OEt$_2$)$_2$][BAr$^F{}_4$] (68.3 mg, 0.068 mmol, 5.0 equiv). Then, CD$_3$CN (0.5 mL) was added to give a yellow suspension. After stirring for 30 minutes at room temperature, the clear yellow-green solution was analyzed spectroscopically.

^{1}H NMR (500 MHz, CD$_3$CN, 298 K): δ 7.81-7.77 (m, 4H, P(O)C$_6$***H***$_5$), 7.72 (s, 16H, B(C$_6$***H***$_3$C$_2$F$_6$)$_4$, overlapping with 3 equiv of [H(OEt$_2$)$_2$][BAr$^F{}_4$]), 7.67 (s, 8H, B(C$_6$***H***$_3$C$_2$F$_6$)$_4$, overlapping with 3 equiv of [H(OEt$_2$)$_2$][BAr$^F{}_4$]), 7.66-7.58 (m, 16H, P(O)C$_6$***H***$_5$), 7.01 (d, 2H, $^3J_{PH}$ = 4.9 Hz, ***H5***), 6.61 (s, 2H, ***H1***), 2.55 (s, 6H, SC***H***$_3$).

^{19}F{^{1}H} NMR (471 MHz, CD$_3$CN, 298 K): δ -63.3.

^{31}P{^{1}H} NMR (202 MHz, CD$_3$CN, 298 K): δ 45.5.

^{11}B NMR (160 MHz, CD$_3$CN, 298 K): δ -6.6.

^{13}C{^{1}H} NMR (126 MHz, CD$_3$CN, 298 K): δ 162.6 (q, $^2J_{CF}$ = 49.8 Hz, B[(3,5-(CF$_3$)$_2$)***C***$_6$H$_3$]$_4$), 148.8 (d, $^2J_{CP}$ = 13.1 Hz, ***C3***), 144.7 (d, $^3J_{CP}$ = 19.1 Hz, ***C6***), 136.7 (d, $^4J_{CP}$ = 3.1 Hz, P(O)***C***$_6$H$_5$), 135.7 (B[(3,5-(CF$_3$)$_2$)***C***$_6$H$_3$]$_4$), 135.3 (d, $^3J_{CP}$ = 3.3 Hz, ***C2***), 133.5 (d, $^2J_{CP}$ = 12.4 Hz, ***C5***), 133.4 (d, $^3J_{CP}$ = 17.2 Hz, P(O)***C***$_6$H$_5$), 132.3 (***C1***), 130.9 (d, $^2J_{CP}$ = 14.0 Hz, P(O)***C***$_6$H$_5$), 130.0 (qq, J_{CF} = 2.8, 31.7 Hz, B[(3,5-(CF$_3$)$_2$)***C***$_6$H$_3$]$_4$), 125.5 (q, $^1J_{CF}$ = 271.7 Hz, B[(3,5-(***C***F$_3$)$_2$)C$_6$H$_3$]$_4$), 123.3 (d, $^1J_{CP}$ = 111.4 Hz, P(O)***C***$_6$H$_5$), 121.6 (d, $^1J_{CP}$ = 111.8 Hz, ***C4***), 118.7 (hept, $^3J_{CF}$ = 3.9 Hz, B[(3,5-(CF$_3$)$_2$)***C***$_6$H$_3$]$_4$), 20.8 (S***C***H$_3$).

III. Additional Reactions and Associated NMR Spectra

PCET between **4⁺** and **Q–H₂**

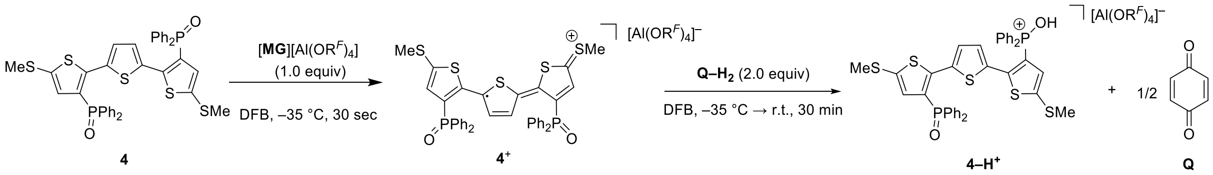

In an argon-filled glovebox, a 5 mL scintillation vial was charged with a magnetic stirbar, **4** (5.0 mg, 6.7 μmol, 1.0 equiv) and [**MG**][Al(OR^F)$_4$] (11.4 mg, 6.7 μmol, 1.0 equiv). Then, cold DFB (0.5 mL) was filtered through a short pad of alumina and added to the vial at –35 °C, immediately giving a dark-turquoise suspension. After manually swirling the vial for 30 seconds, a clear dark-turquoise solution was obtained. Then, 1,4-dihydroquinone (**Q–H₂**) (1.5 mg, 13.5 μmol, 2.0 equiv) was added. The reaction mixture was then stirred for 30 minutes at room temperature. The resulting pale yellow suspension was dried *in vacuo* and re-dissolved in CD_3CN, filtered and transferred into a NMR tube for analysis. A reaction yield of 71% was determined using a ferrocene internal standard by ^{1}H NMR spectroscopy.

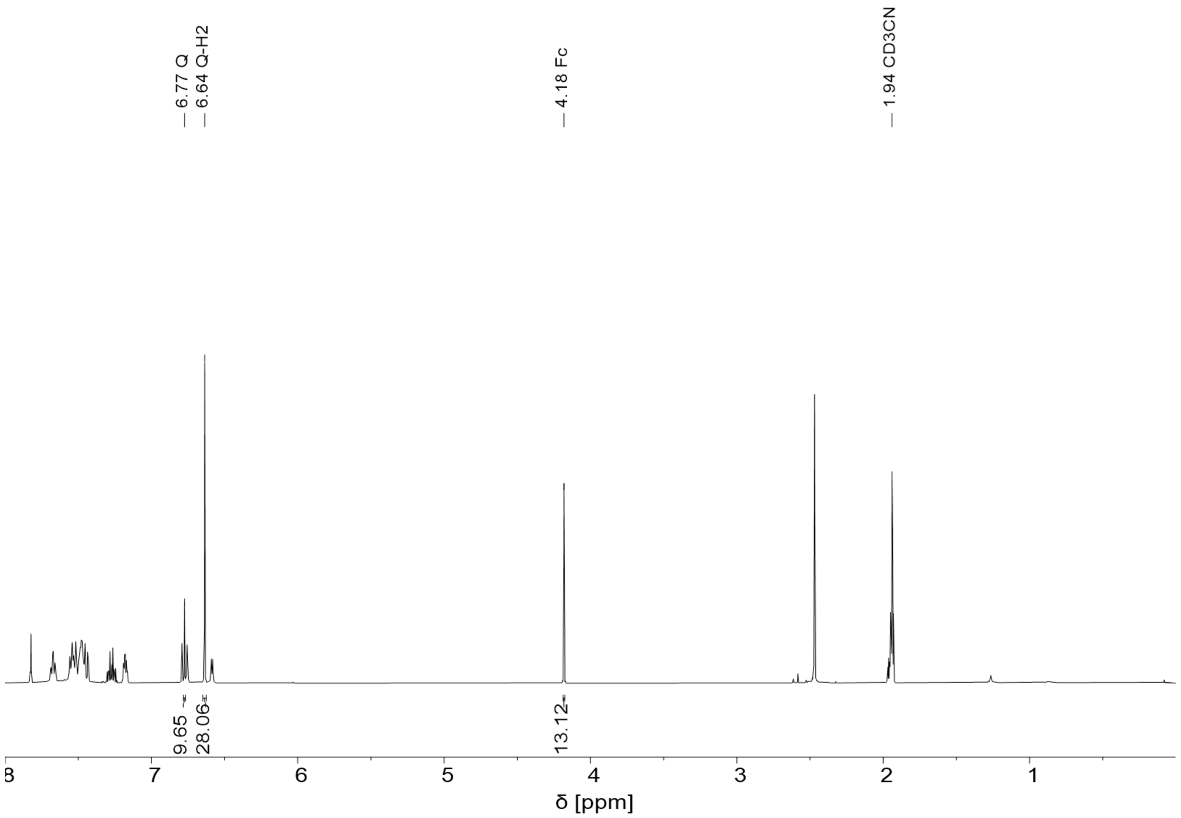

Figure S3. ^{1}H NMR (500 MHz, CD_3CN, 298 K) spectrum of PCET between **4⁺** and **Q–H₂**. **Q** = Benzoquinone, **Q–H₂** = 1,4-dihydroquinone, Fc = ferrocene.

Attempted PCET between 4^{2+} and **Q–H₂**

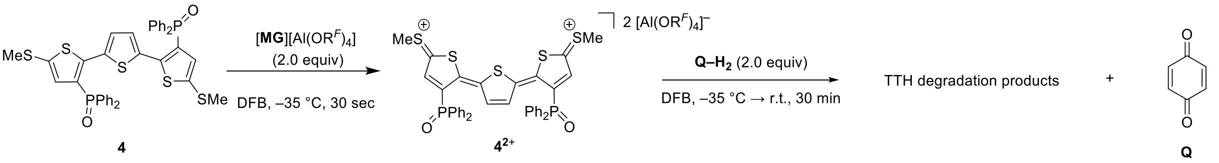

In an argon-filled glovebox, a 5 mL scintillation vial was charged with a magnetic stirbar, **4** (5.0 mg, 6.7 μmol, 1.0 equiv) and [**MG**][Al(OR^F)$_4$] (22.8 mg, 13.5 μmol, 2.0 equiv). Then, cold DFB (0.5 mL) was filtered through a short pad of alumina and added to the vial at –35 °C, immediately giving a dark-turquoise suspension. After manually swirling the vial for 30 seconds, a clear dark-turquoise solution was obtained. Then, 1,4-dihydroquinone (**Q–H$_2$**) (1.5 mg, 13.5 μmol, 2.0 equiv) was added. The reaction mixture was then stirred for 30 minutes at room temperature. The resulting pale yellow suspension was dried *in vacuo* and re-dissolved in CD_3CN, filtered and transferred into a NMR tube for analysis.

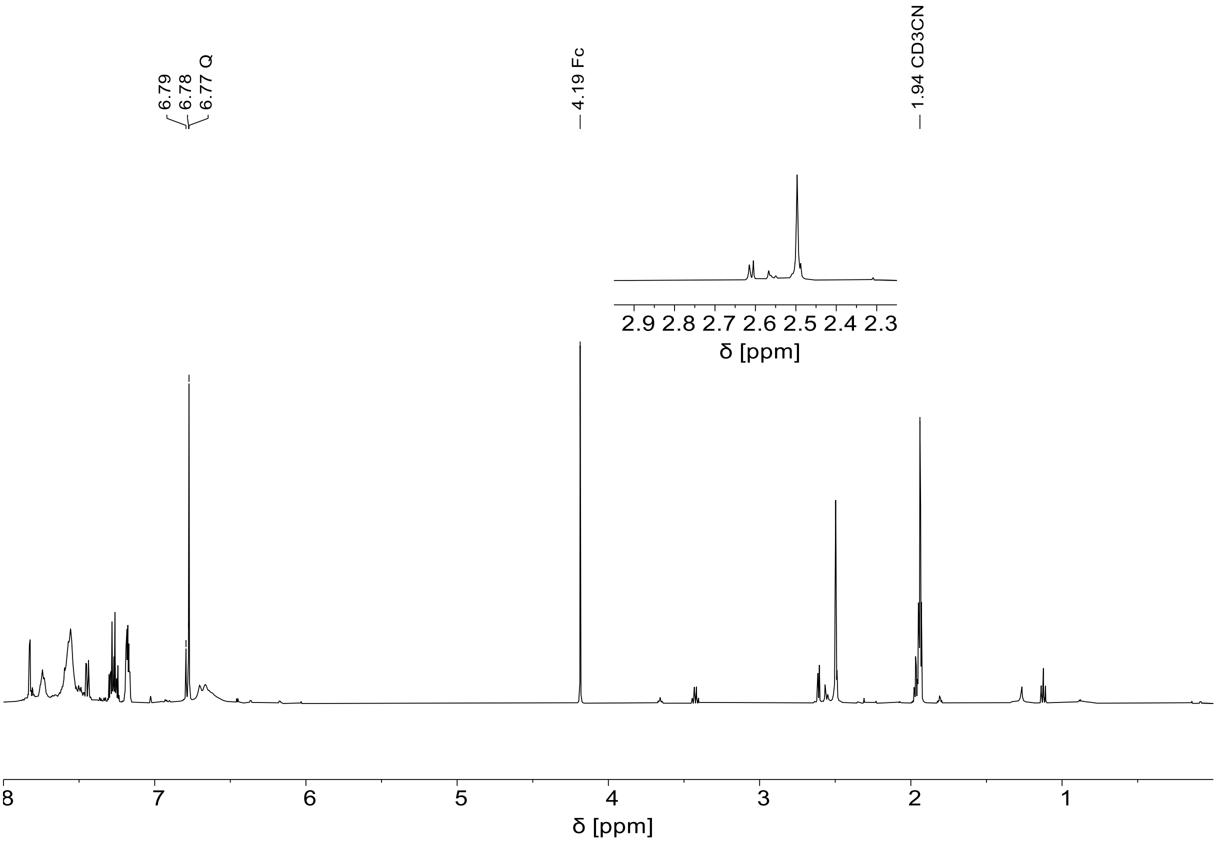

Figure S4. ^1H NMR (500 MHz, CD_3CN, 298 K) spectrum of the attempted PCET between 4^{2+} and **Q–H$_2$**. **Q** = Benzoquinone, Fc = ferrocene. Inset shows C–S bond cleavage products.

The NMR shows a complex mixture with broadened signals and partial C–S bond cleavage of the methyl thioether in **4**, visible in the region between 3 and 2 ppm. No reliable determination of the reaction yield could be performed due to signals overlapping with 1,4-benzoquinone (**Q**).

PCET between **4–H** and **DMAQ**

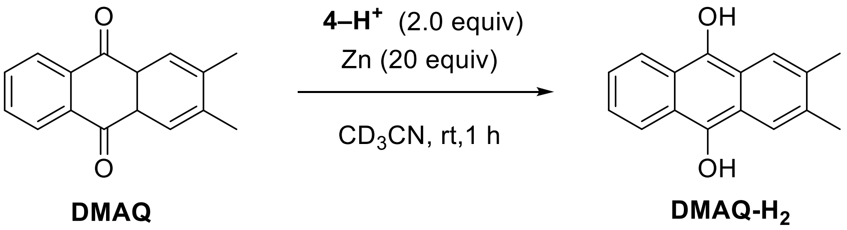

In an argon-filled glovebox, a 5 mL scintillation vial was charged with a magnetic stirbar, **4** (10 mg, 13.5 μmol, 1.0 equiv) and $[H(OEt_2)_2][BAr^F_4]$ (Ar^F = 3,5-$(CF_3)_2C_6H_3$) (13.7 mg, 13.5 μmol, 1.0 equiv). Then, CD_3CN (0.5 mL) was filtered through a short pad of alumina and added to the vial to generate **4–H⁺** *in situ*. The resulting dark-yellow reaction mixture was stirred until complete dissolution was achieved, after which time 2,3-dimethyl anthraquinone (1.6 mg, 6.75 μmol, 0.5 equiv) was added, followed by zinc (8.8 mg, 135 μmol, 10 equiv). The reaction mixture was then stirred for 1 hour at room temperature. The resulting pale yellow solution was filtered and transferred into a NMR tube. A reaction yield of 88% was determined using a ferrocene internal standard by ^{1}H NMR spectroscopy.

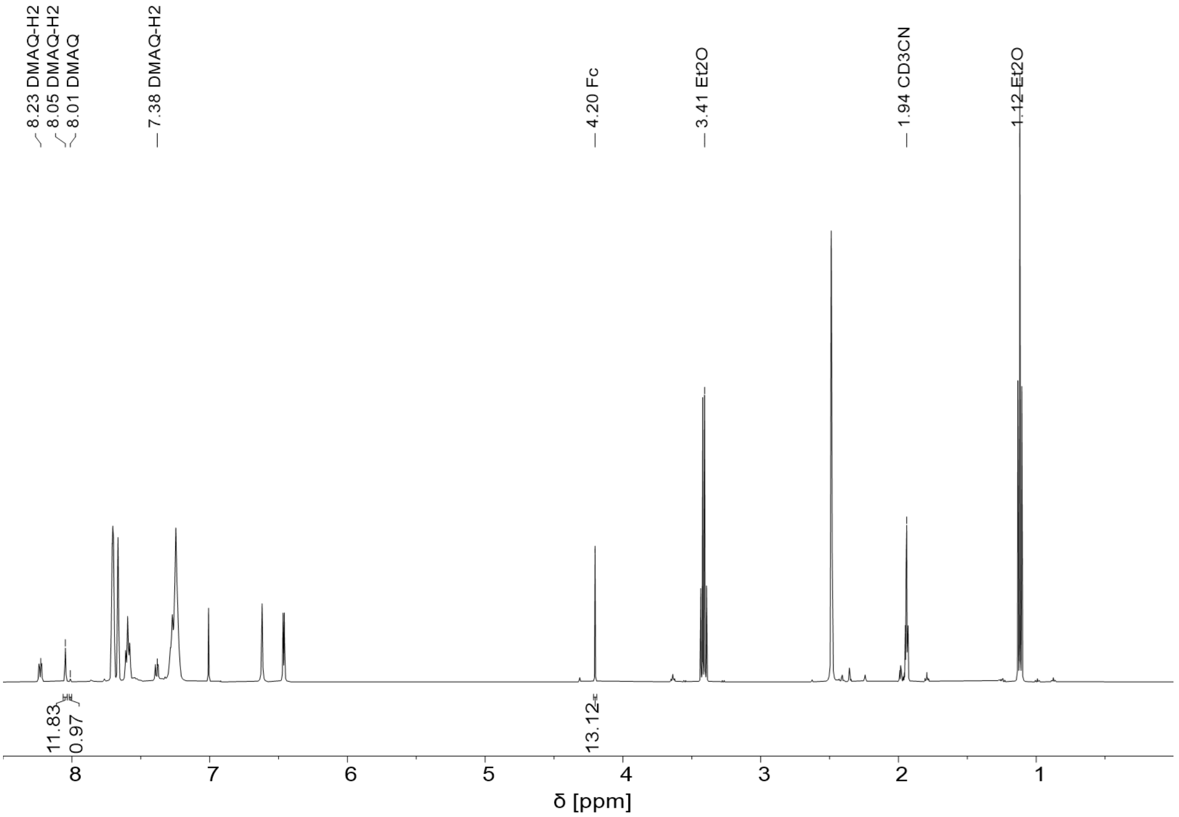

Figure S5. ^{1}H NMR (500 MHz, CD_3CN, 298 K) spectrum of the reaction of **DMAQ** with **4–H⁺**. **DMAQ–H₂** = 2,3-dimethyl-9,10-dihydroxyanthracene, Fc = ferrocene.

Generation of **4⁺**

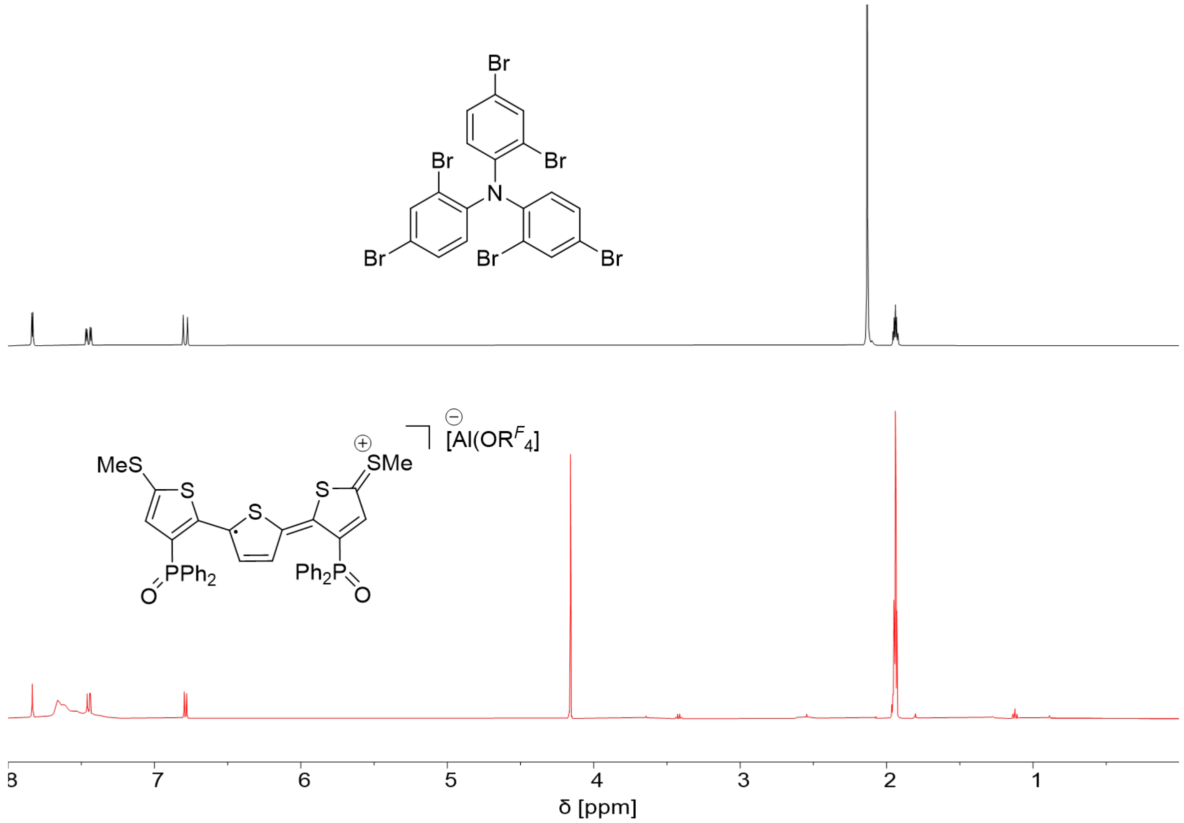

Figure S6. ^{1}H NMR (500 MHz, CD_3CN, 298 K) spectrum comparison of (2,4-Br_2-Ph)$_3$N (top, black) and *in-situ* generated **4⁺** with a Fc capillary (bottom, red) in CD_3CN showing formation of (2,4-Br_2-Ph)$_3$N, supporting electron transfer from **4** to [**MG**][Al(OR^F)$_4$].

4–H⁺ Variable Temperature NMR Data

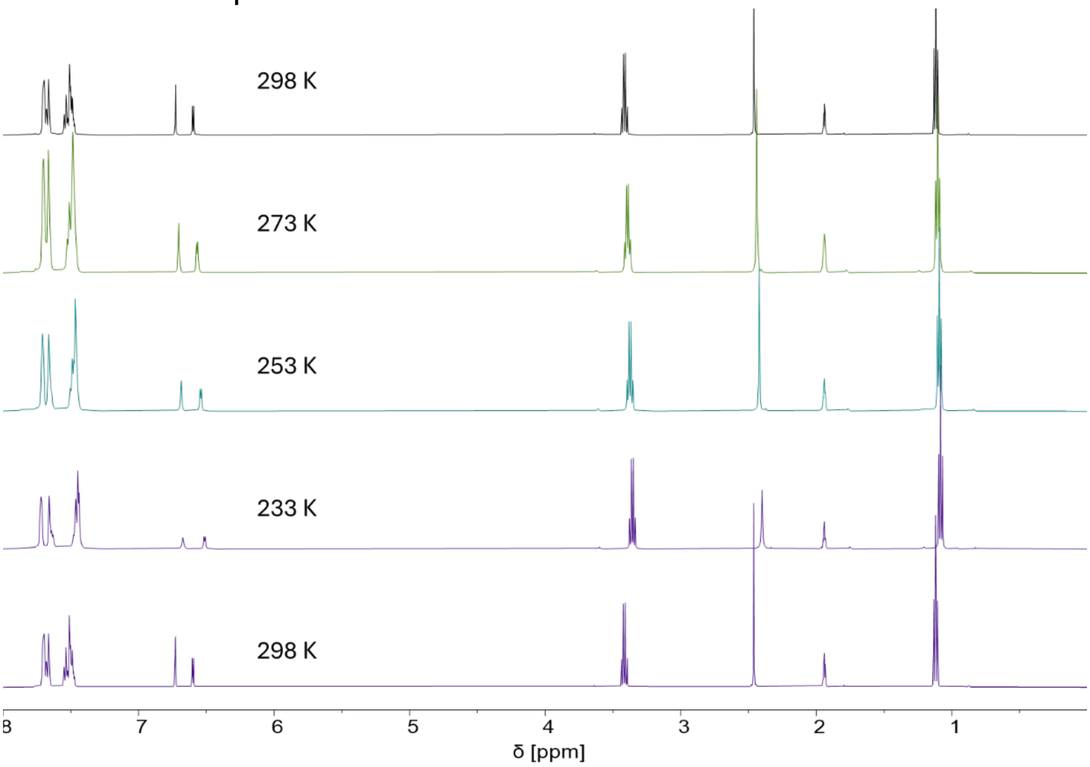

Figure S7. Variable temperature ^{1}H NMR (500 MHz, CD_3CN, 298 K) spectra of **4–H⁺** at 298 K, 273 K, 253 K and 233 K. After cooling to 233 K, the temperature was increased back to 298 K and an additional spectrum was recorded to rule out decomposition during the experiment.

Addition of $[H(OEt_2)_2][BAr^F{}_4]$ to **4**

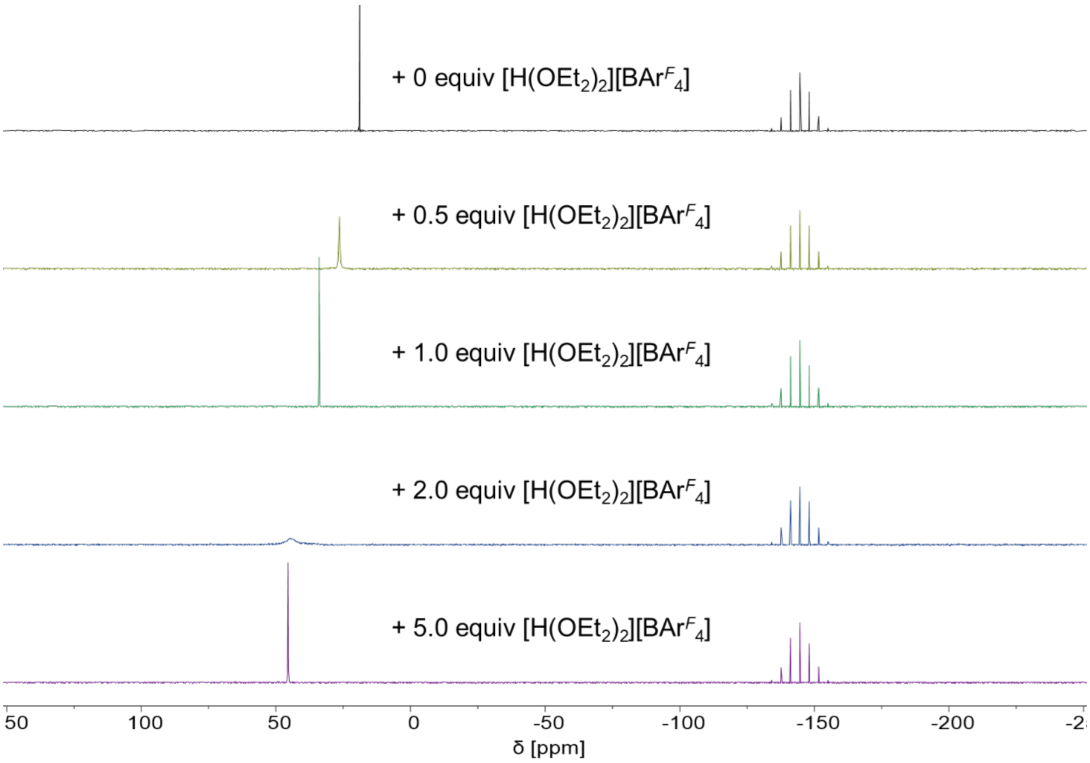

Figure S8. $^{31}P\{^{1}H\}$ NMR spectra (202 MHz, CD_3CN, 298 K) of **4** in CD_3CN upon addition of increasing amounts of $[H(OEt_2)_2][BAr^F{}_4]$. A capillary of $LiPF_6$ in CD_3CN was used as reference.

Hydrogen Evolution from **4–H**

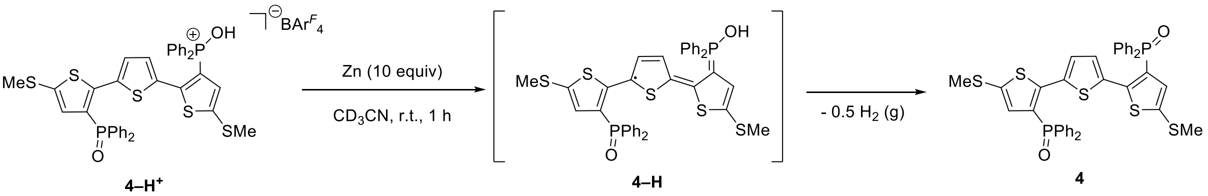

The calculated BDFE of **4–H** (48 kcal mol^{-1}) indicates that this species may be thermodynamically unstable with respect to H_2 evolution ($\Delta G°_f(H•)$ = 52 kcal/mol in MeCN).[22] The single-electron reduction of **4–H⁺** was hence performed in the absence of a hydrogen atom acceptor with the goal of observing H_2 evolution:

In an argon-filled glovebox, a 5 mL scintillation vial was charged with a magnetic stirbar, **4** (10 mg, 13.5 μmol, 1.0 equiv) and [H(OEt$_2$)$_2$][BAr$^F_4$] (Ar^F = 3,5-(CF$_3$)$_2$C$_6$H$_3$) (13.7 mg, 13.5 μmol, 1.0 equiv). Then, CD_3CN (0.5 mL) was filtered through a short pad of alumina and added to the vial to generate **4–H⁺** *in situ*. The resulting mixture was stirred until complete dissolution was achieved, after which time the resulting dark-yellow solution was transferred into a J.Young NMR tube. Then, zinc (8.8 mg, 135 μmol, 10 equiv) was added and the NMR tube was immediately sealed. Upon addition of zinc, the formation of gas bubbles was observed. The J.Young NMR tube was then rotated end-over-end for 1 h, after which time the ^{1}H NMR of the reaction mixture was recorded, revealing H_2 formation.

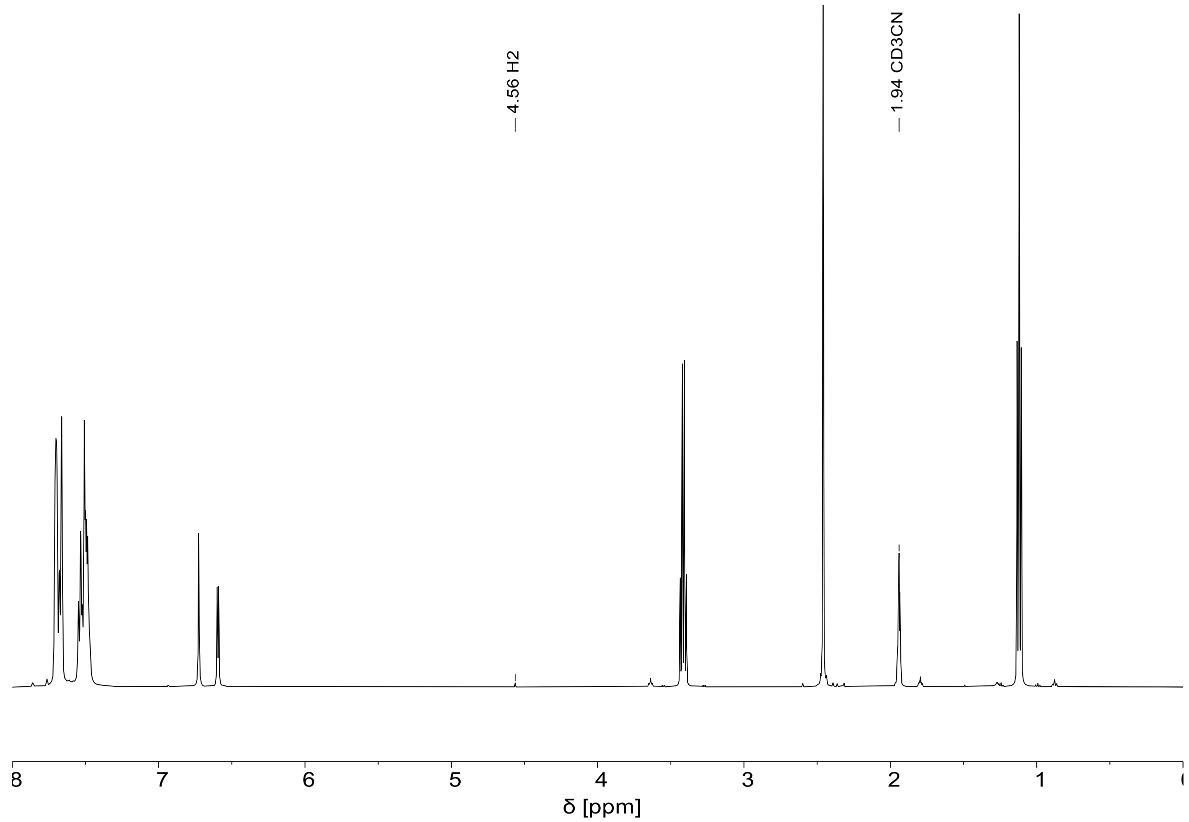

Figure S9. ^{1}H NMR (500 MHz, CD_3CN, 298 K) spectrum of a mixture of **4–H⁺** and 10 equiv of Zn after reaction at room temperature for 1 h. Formation of gas bubbles can be observed and the characterstic signal of H_2 in CD_3CN can be detected at 4.56 ppm.

Control Experiment to Rule Out Reduction of **DMAQ** by Zinc

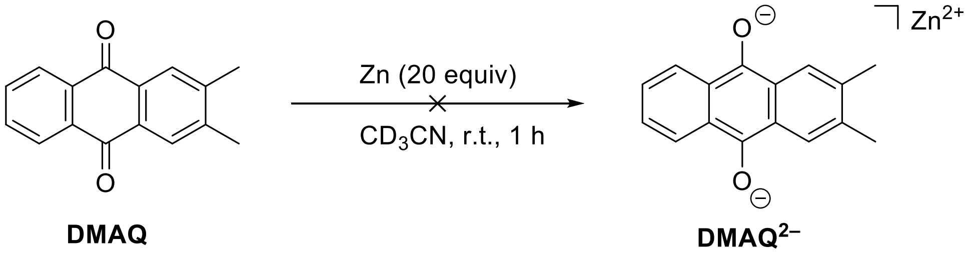

To rule out the possibility of direct **DMAQ** reduction by zinc during the generation of **4–H**, the following control experiment was performed:

In an argon-filled glovebox, a 5 mL scintillation vial was charged with a magnetic stirbar, **DMAQ** (1.6 mg, 6.75 μmol, 0.5 equiv) and zinc (8.8 mg, 135 μmol, 20.0 equiv). After addition of CD_3CN (0.5 mL) the reaction mixture was stirred for 1 h at r.t. Then, the reaction mixture was transferred into a J.Young NMR tube and a ^1H NMR spectrum was recorded. During the course of the reaction, no change was observed in the ^1H NMR spectrum of the reaction mixture and no color change could be detected by eye. These observations were taken to rule out the direct reduction of **DMAQ** by zinc during the *in situ* generation of **4–H**.

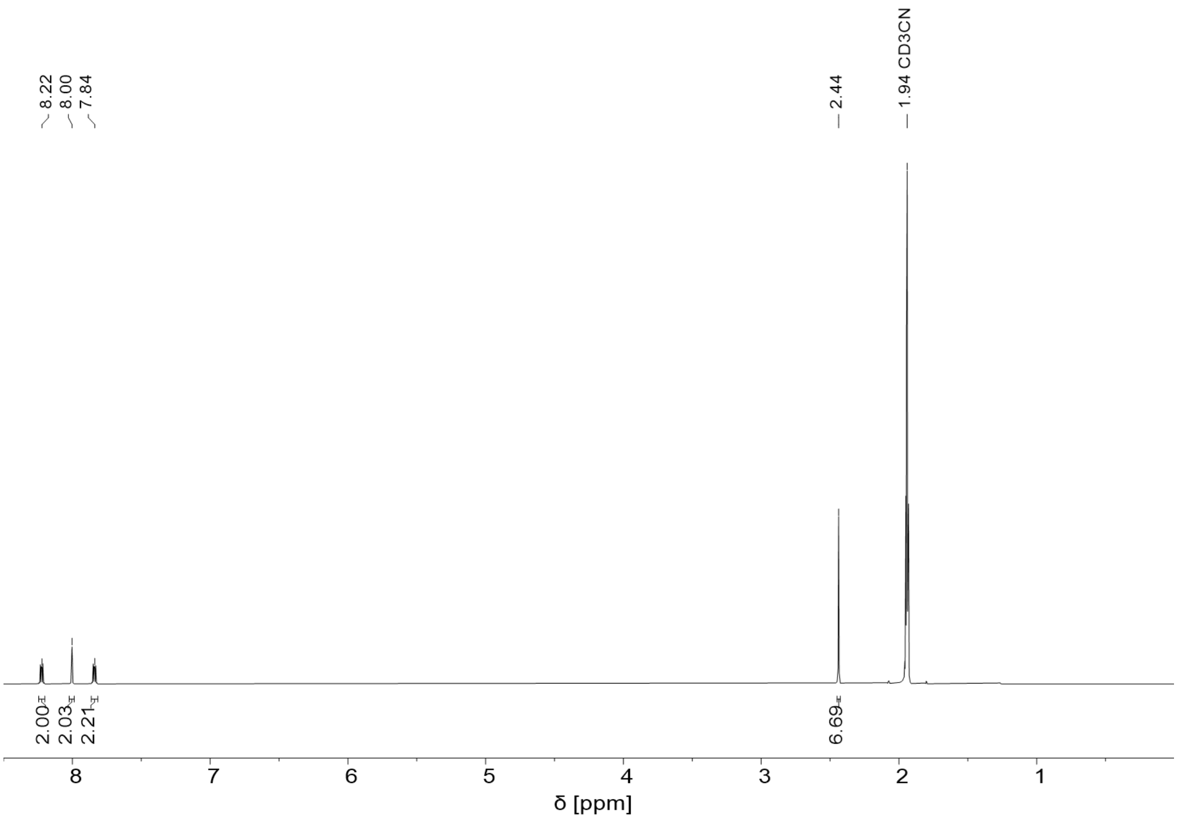

Figure S10. ^1H NMR (500 MHz, CD_3CN, 298 K) spectrum of a mixture of **DMAQ** and Zn (20 equiv) after stirring for 1 h in a CD_3CN solution.

Control Experiment to Rule Out Protonation of **DMAQ** by **4–H⁺**

To rule out the possibility of direct **DMAQ** protonation by **4–H⁺** during the generation of **4–H**, the following control experiment was performed:

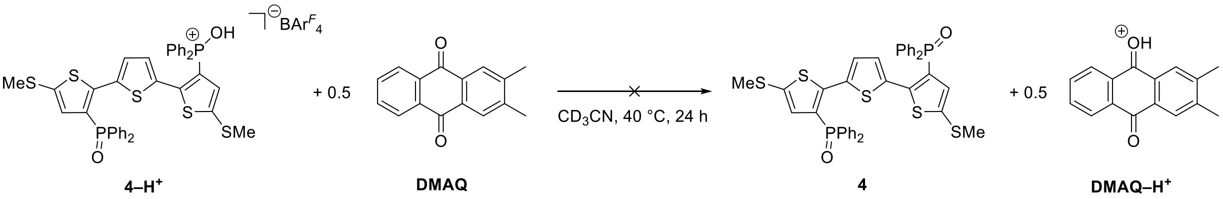

In an argon-filled glovebox, a 5 mL scintillation vial was charged with a magnetic stirbar, **4** (10 mg, 13.5 µmol, 1.0 equiv) and [H(OEt$_2$)$_2$][BAr$^F_4$] (Ar^F = 3,5-(CF$_3$)$_2$C$_6$H$_3$) (13.7 mg, 13.5 µmol, 1.0 equiv). Then, CD$_3$CN (0.5 mL) was filtered through a short pad of alumina and added to the vial to generate **4–H⁺** *in situ*. The resulting dark-yellow reaction mixture was stirred until complete dissolution was achieved, after which time **DMAQ** (1.6 mg, 6.75 µmol, 0.5 equiv) was added. After stirring for 1 h at r.t., the dark-yellow solution was transferred to a J.Young NMR tube. During the course of the reaction, no change was observed in the ^{1}H NMR spectrum of the reaction mixture. To rule out slow kinetics of proton transfer, the J.Young NMR tube was heated at 40 °C for 24 h, after which time no change in the ^{1}H NMR spectrum could be observed. These observations were taken to rule out the direct proton transfer from **4–H⁺** to **DMAQ** during the *in situ* generation of **4–H**.

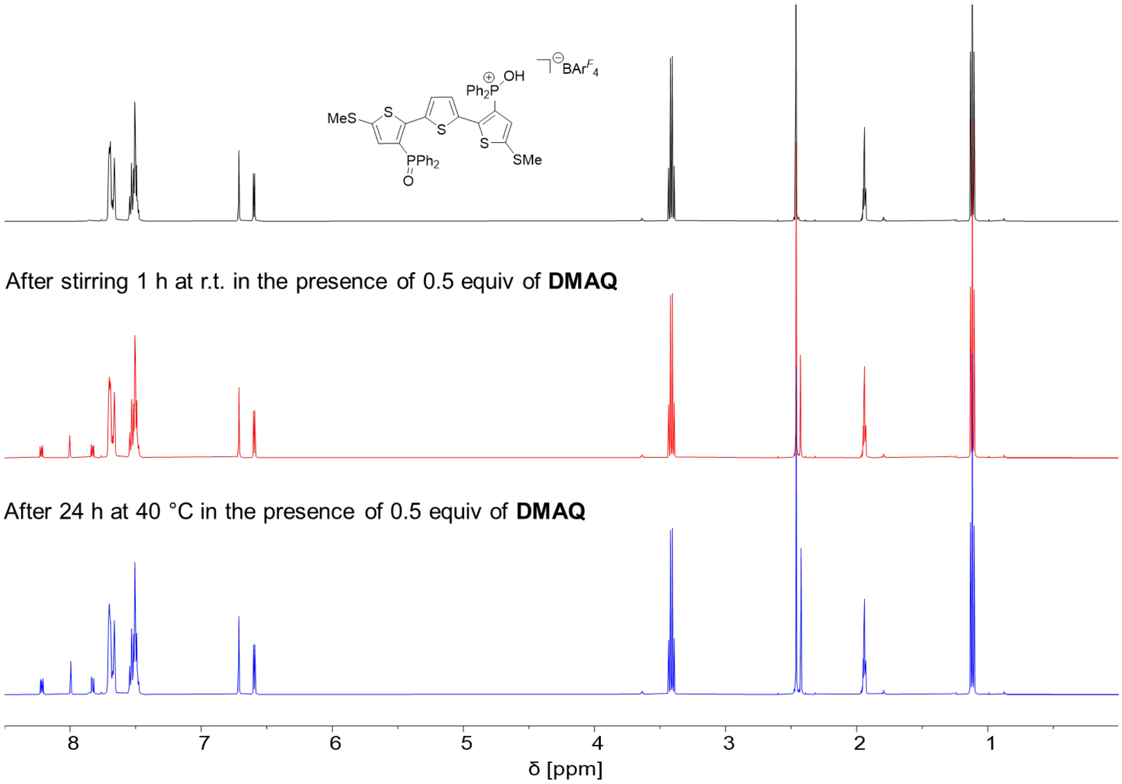

Figure S11. ^{1}H NMR (500 MHz, CD$_3$CN, 298 K) spectrum comparison of **4–H⁺** (top, black), after stirring 1 h at r.t. in the presence of 0.5 equiv **DMAQ** (middle, red) and after 24 h at 40 °C in the presence of **DMAQ** (bottom, blue). No evidence for proton transfer from **4–H⁺** to **DMAQ** can be observed.

Protonation of **DMAQ** using $[H(OEt_2)_2][BAr^F_4]$

In an attempt to estimate the pK_a of **DMAQ** in MeCN, $[H(OEt_2)_2][BAr^F_4]$ was used as a reference acid.

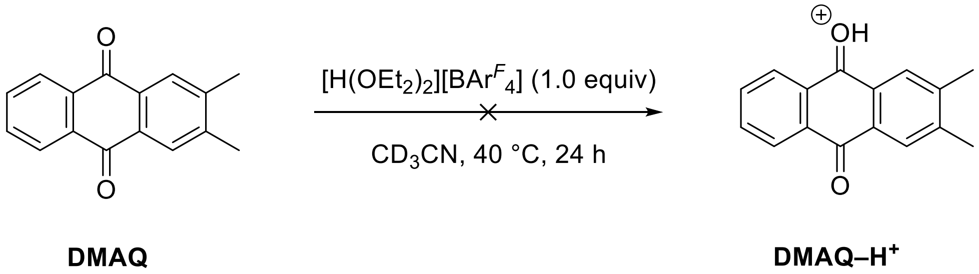

In an argon-filled glovebox, a 5 mL scintillation vial was charged with a magnetic stirbar, **DMAQ** (3.2 mg, 13.5 µmol, 1.0 equiv) and $[H(OEt_2)_2][BAr^F_4]$ (13.7 mg, 13.5 µmol, 1.0 equiv). After addition of CD_3CN, the reaction mixture was stirred for 1 h at r.t. Then, the reaction mixture was transferred into a J.Young NMR tube and a ^1H NMR spectrum was recorded. During the course of the reaction, no change was observed in the ^1H NMR spectrum of the reaction mixture. To rule out slow kinetics of proton transfer, the J.Young NMR tube was heated at 40 °C for 24 h, after which time no evidence for proton transfer was observed, only partial degradation of $[H(OEt_2)_2][BAr^F_4]$. These observations establish that the pK_a of **DMAQ** is lower than that of $[H(OEt_2)_2][BAr^F_4]$.

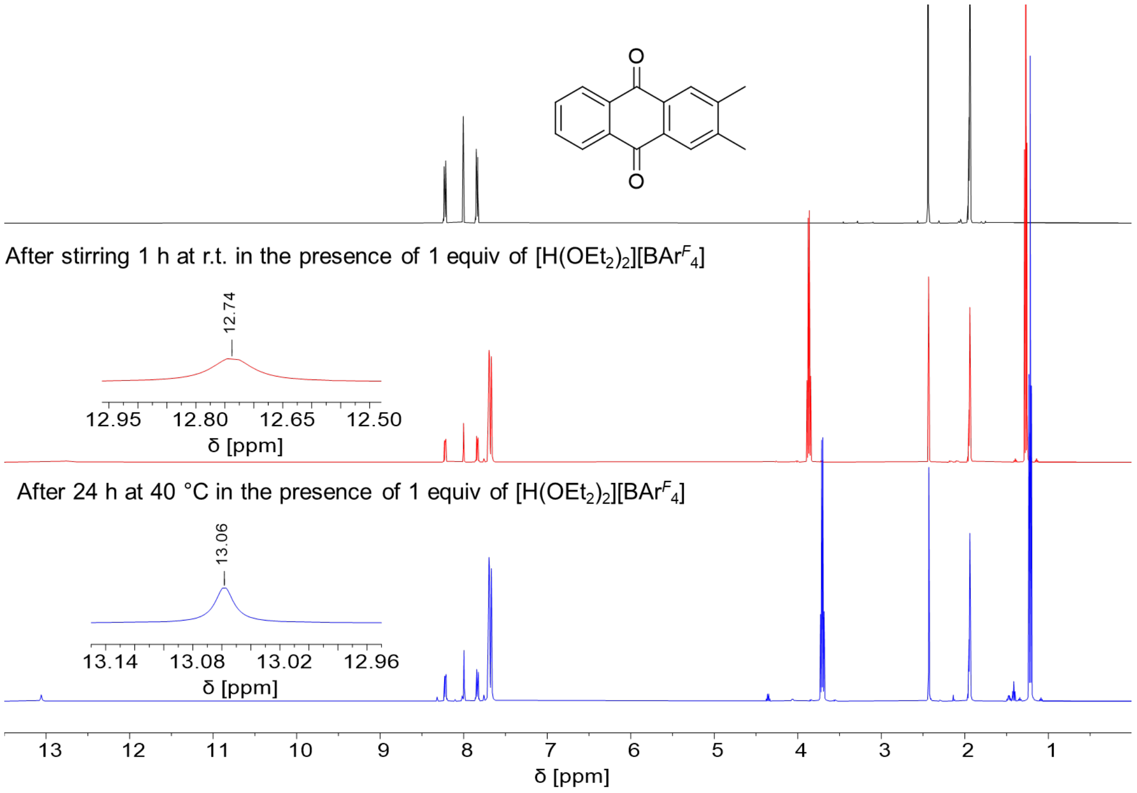

Figure S12. ^1H NMR (500 MHz, CD_3CN, 298 K) spectrum comparison of **DMAQ** (top, black), a 1:1 mixture of **DMAQ**:$[H(OEt_2)_2][BAr^F_4]$ ($Ar^F = 3,5\text{-}(CF_3)_2C_6H_3$) after stirring for 1 h at r.t (middle, red) and after 24 h at 40 °C (bottom, blue). No protonation of **DMAQ** could be identified after 1 h at r.t. After 24 h at 40 °C partial degradation of $[H(OEt_2)_2][BAr^F_4]$ was detected whilst no protonation of **DMAQ** is observed.

IV. pK_a Determinations

For the reaction:

$$AH^+ \overset{K_{eq}}{\rightleftharpoons} A + H^+ \qquad (S1)$$

the pK_{AH} can be written as:

$$pK_{AH} = -log\frac{[A][H^+]}{[AH^+]} \qquad (S2)$$

Thus, if we consider an equilibrium between acid (AH^+) and base (B):

$$AH^+ + B \overset{K_{eq}}{\rightleftharpoons} A + BH^+ \qquad (S3)$$

we can write:

$$\Delta pKa = pK_{AH}(A) - pK_{AH}(B) = -log\frac{[A][H^+]}{[AH^+]} + log\frac{[B][H^+]}{[BH^+]} = log\frac{[AH^+][B]}{[A][BH^+]} \qquad (S4)$$

We assume that:

$$[A] = [BH]^+ \qquad (S5)$$

We determine the degree of protonation (x_A) of A using the experimentally observed NMR chemical shift of the equilibrium mixture (δ_{obs}) alongside the chemical shifts of fully protonated AH^+ (δ^{AH+}) and fully de-protonated A (δ^A):

$$x_A = \frac{\delta_{obs} - \delta^A}{\delta^{AH+} - \delta^A} \qquad (S6)$$

Thus, we can calculate:

$$[AH^+] = [A]_{tot} * x_A \qquad (S7)$$

Using the following equations:

$$[A]_{tot} = [A] + [AH^+] \qquad (S8)$$

$$[A] = [A]_{tot} - [AH^+] \qquad (S9)$$

$$[B]_{tot} = [B] + [BH]^+ \qquad (S10)$$

we can calculate the concentration of every species in equation (S4) and determine the ΔpK_a between acid (AH^+) and reference base (B).

pK_a Determination by NMR

The general procedure below was adapted from the literature:[23]

In an argon-filled glovebox, a 5 mL scintillation vial was charged with a magnetic stirbar and 6.75 µmol of acid (AH^+). Then, CD_3CN (0.5 mL) was filtered through a short pad of alumina and added to the vial to dissolve the acid under stirring. The resulting solution was then transferred into a J.Young NMR tube. After recording ^1H and ^{31}P NMR spectra, 6.75 µmol of the reference base (B) from a stock solution was added and ^1H and ^{31}P NMR spectra were recorded. Each reported pK_a value is the average of three independent trials. No evidence for ion pairing was observed.[24]

In Table S1 the obtained pK_a values are shown.

Table S1. Overview of the pK_a values determined by NMR titration.

Acid (AH^+)	Base (B)	pK_{AH} (B, MeCN)[25]	ΔpK_a	pK_a (AH^+, MeCN)
**4–H+**	Ph_3P	7.6	-0.5 ± 0.1	7.1 ± 0.1
$[H(OEt_2)_2][BAr^F_4]$	Ph_3N	1.3	-1.2 ± 0.1	0.1 ± 0.1

NMR Spectra

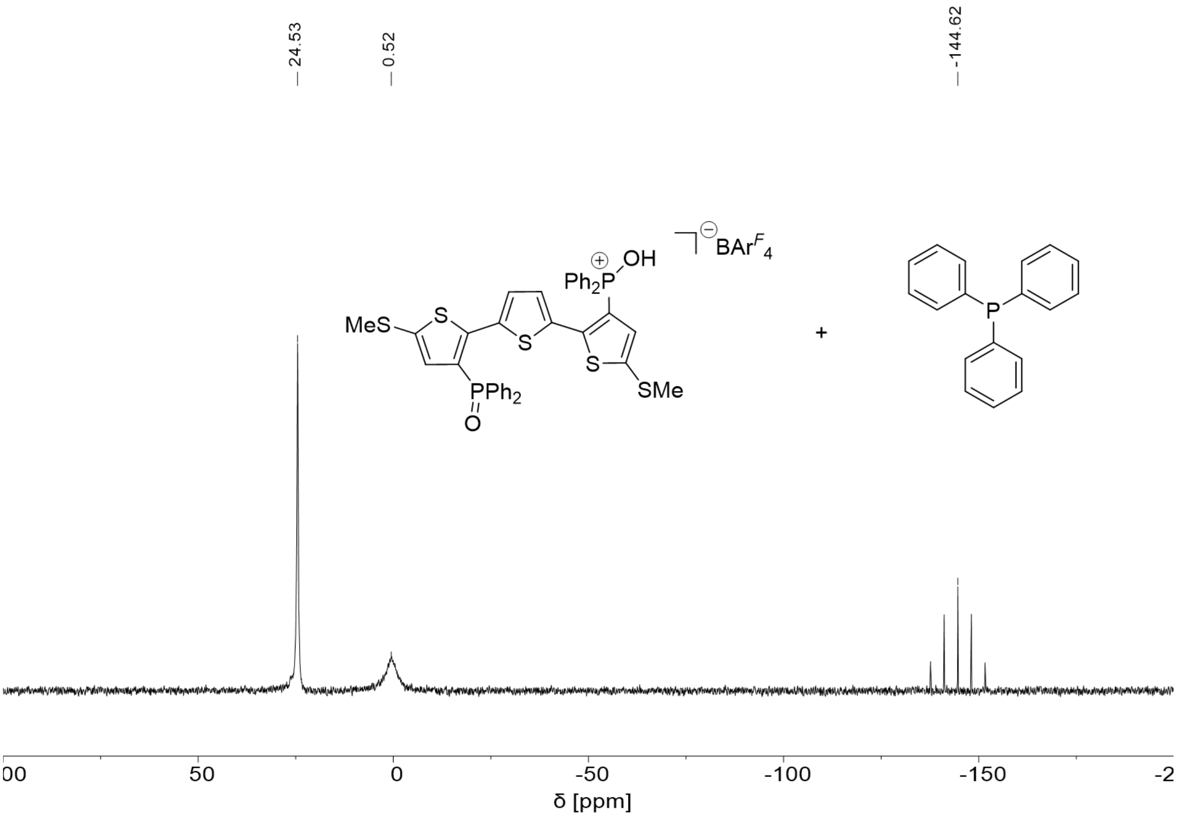

Figure S13. $^{31}P\{^1H\}$ (202 MHz, CD_3CN, 298 K) NMR spectrum of a 1:1 mixture of **4–H+** and Ph_3P. A $Li[PF_6]$ capillary in CD_3CN was used as internal standard.

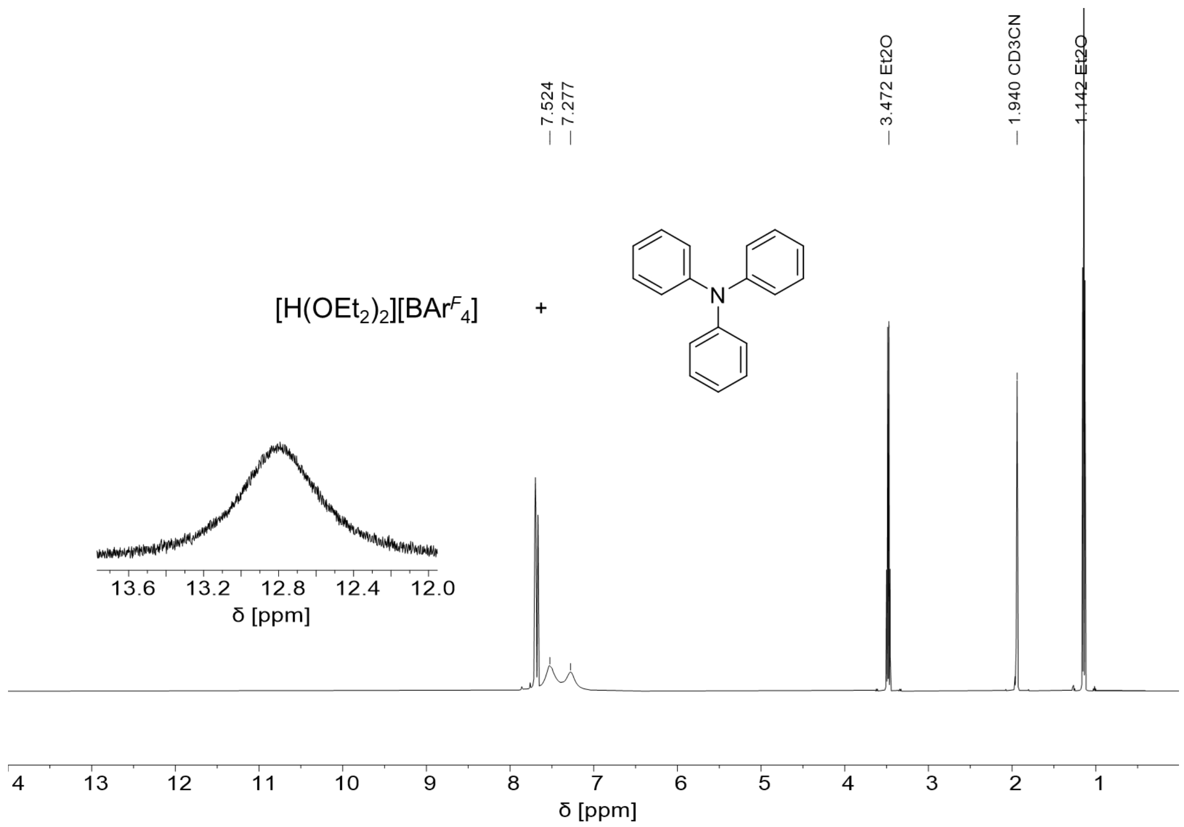

Figure S14. ^{1}H (500 MHz, CD_3CN, 298 K) NMR spectrum of a 1:1 mixture of $[H(OEt_2)_2][BAr^F_4]$ and Ph_3N.

V. PCET Mechanism

PCET between 4^+ and **Q–H$_2$**

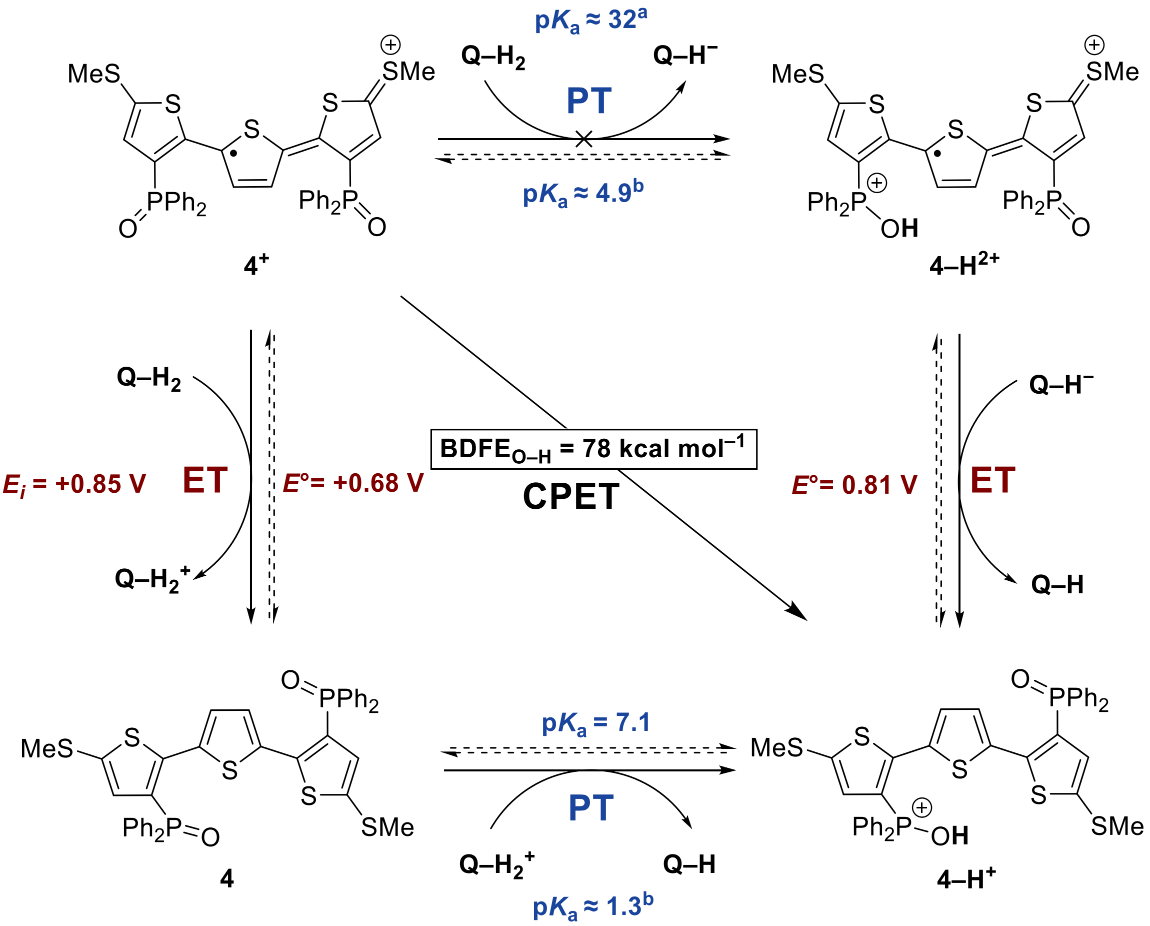

Scheme S1. Square scheme showing possible mechanisms for the PCET reaction between **4**$^+$ and **Q–H$_2$**. All experimental measurements were conducted in MeCN solution. $E°$ values are reported as half-wave potentials and E_i refers to the inflection point of an irreversible electrochemical feature (1.0 mM concentration with 0.10 M [(n-Bu)$_4$N][PF$_6$] supporting electrolyte, 100 mV sec^{-1} scan rate, r.t., glassy carbon working electrode). [a]: Converted value in MeCN following literature procedures[26] using pK_a (DMSO) = 19.8[27]. [b]: Calculated from Bordwell equation using experimentally determined values for BDFE$_{O\text{-}H}$ and $E°$.

Electron Transfer-Proton Transfer (ET-PT): The thermodynamic feasibility of electron transfer between **Q–H$_2$** and **4**$^+$ was evaluated using electrochemical data. The open-circuit potential of **4**$^+$ was determined to be E_{OC} = 0.67 V vs. Fc/Fc$^+$, consistent with the half-wave potential of the **4**/**4**$^+$ redox couple ($E°$ = +0.68 V vs. Fc/Fc$^+$). The oxidation potential of **Q–H$_2$** was estimated to be $E_i°$ = +0.85 V vs Fc/Fc$^+$ using the inflection point of the anodic wave in its cyclic voltammogram. These results yield $\Delta E°$ = 0.18 V, and hence $\Delta G_{ET} \approx$ +4 kcal mol^{-1}. The subsequent proton transfer between **4** and **Q–H$_2$$^+$** is expected to be thermodynamically favorable based on the experimentally determined pK_a of **4** (pK_a = 7.1 in MeCN) and the pK_A of **Q–H$_2$$^+$** (p$K_A$ ≈ 1.3 in MeCN calculated by the Bordwell equation using a DFT-computed BDFE$_{O\text{-}H}$ of 74 kcal mol^{-1} for **Q–H$_2$** [see DFT section below below] and $E_i°$ = +0.85 V vs Fc/Fc$^+$ for the **Q–H$_2$$^+$/Q–H$_2$** redox couple). Therefore, $\Delta G_{PT} \approx -8$ kcal mol^{-1}, rendering an ET-PT mechanism thermodynamically feasible.

Proton Transfer-Electron Transfer (PT-ET): The thermodynamic feasibility of proton transfer between **Q–H$_2$** and **4**$^+$ was evaluated using information about their respective pK_a values. The

experimentally determined pK_a value of **Q–H$_2$** is available in DMSO solvent (pK_a [DMSO] = 19.8).[27] According to literature procedures, a pK_a value in DMSO of an OH-acid can be converted to a pK_a value in MeCN using the following equation[26]:

$$pK_a(DMSO) = pK_a(MeCN, OH) * 0.87(0.02) - nNO2 * 1.0(0.1) + nCF3 * 0.37(0.09) - 8.0(0.6) \quad \text{(S11)}$$

This yields pK_a(**Q–H$_2$**, MeCN) ≈ 32. The pK_a of **4$^+$** was estimated to be ≈ 4.9 (MeCN) by the Bordwell equation using an experimentally-determined BDFE$_{O-H}$ of 78 kcal mol^{-1} for **4–H$^+$** and $E°$ = +0.81 V vs Fc/Fc$^+$ for the **4–H^{2+}/4–H$^+$** redox couple. Therefore, ΔG_{PT} ≈ + 37 kcal mol^{-1}, rules out the thermodynamic feasibility of an PT–ET pathway.

Therefore, the PCET reaction between **Q–H$_2$** and **4$^+$** likely follows either a ET-PT or a concerted proton-electron transfer (CPET) pathway.

PCET between **4–H** and **DMAQ**

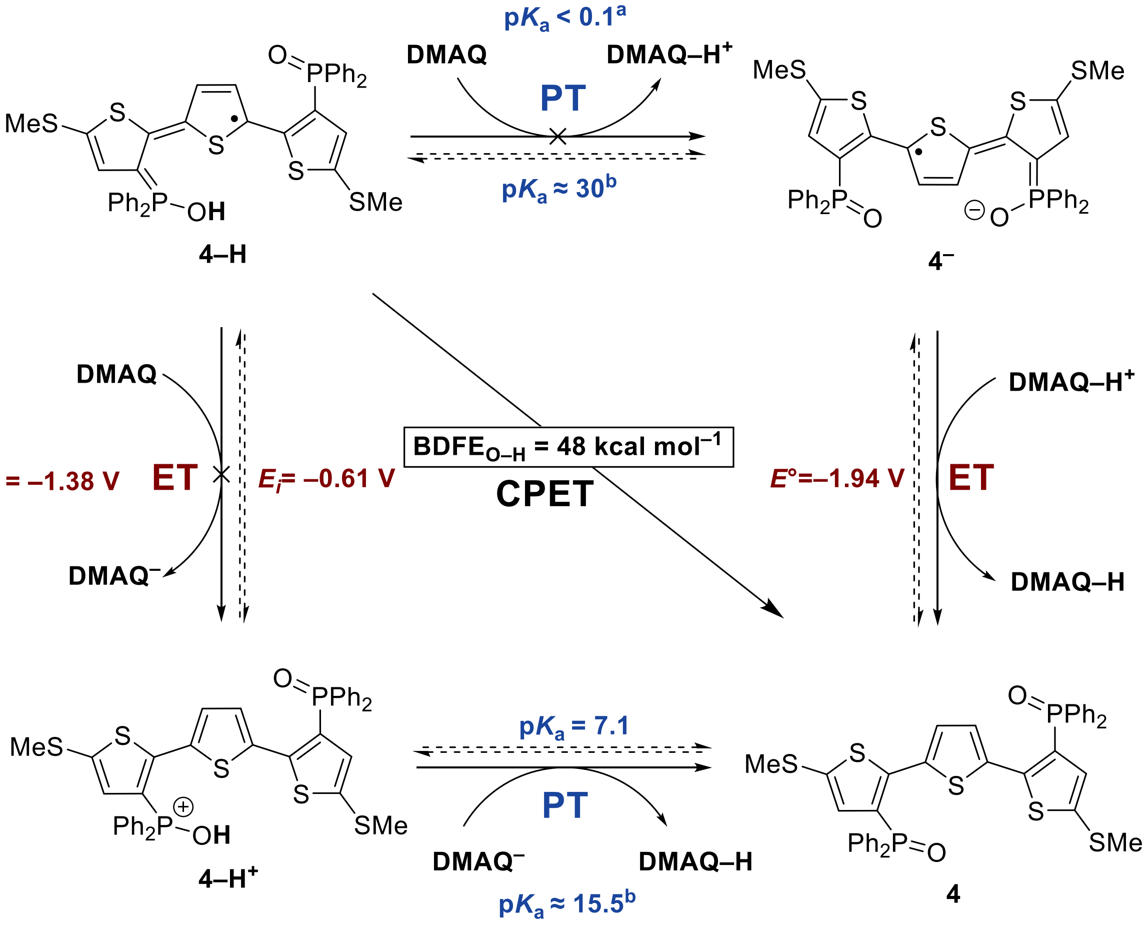

Scheme S2. Square scheme showing possible mechanisms for the PCET reaction between **4–H** and **DMAQ**. All experimental measurements were conducted in MeCN solution. $E°$ values are reported as half-wave potentials and E_i refers to the inflection point of an irreversible electrochemical feature (1.0 mM concentration with 0.10 M [(n-Bu)$_4$N][PF$_6$] supporting electrolyte, 100 mV sec^{-1} scan rate, r.t., glassy carbon working electrode). [a]: see chapter III: Additional Reactions and Associated NMR Spectra and chapter IV: pK_a determination. [b]: Calculated from Bordwell equation using experimentally determined values for BDFE$_{O-H}$ and $E°$.

Electron Transfer-Proton Transfer (ET-PT): The thermodynamic feasibility of electron transfer between **DMAQ** and **4–H** was evaluated using electrochemical data. The cyclic

voltammogram of **DMAQ** features a reversible cathodic wave with E^0 = –1.38 V. On the other hand, the cyclic voltammogram of **4–H⁺** exhibits an irreversible cathodic wave with E_i = –0.61 V. We thus estimate $\Delta E° \approx 0.77$ V, and hence $\Delta G_{ET} \approx +18$ kcal mol^{-1}, ruling out the thermodynamic feasibility of an ET–PT pathway between **DMAQ** and **4–H**.

Proton Transfer-Electron Transfer (PT-ET): The thermodynamic feasibility of proton transfer between **DMAQ** and **4–H** was evaluated using estimation of their respective pK_a values. The pK_a of **4** was estimated to be p$K_a \approx 30$ (MeCN) by the Bordwell equation using an experimentally-determined BDFE$_{O–H}$ of 48 kcal mol^{-1} for **4–H** and $E°$ = –1.94 V vs Fc/Fc$^+$ for the **4**/**4**$^-$ redox couple. To estimate an upper bound for its pK_a, **DMAQ** was treated with 1 equivalent of $[H(OEt_2)_2][BAr^F_4]$ (Ar^F = 3,5-$(CF_3)_2C_6H_3$) at r.t. in CD_3CN and stirred for 1 h. Then, a ^{1}H spectrum was recorded to reveal that no protonation of **DMAQ** has occurred and intact $[H(OEt_2)_2][BAr^F_4]$ (Ar^F = 3,5-$(CF_3)_2C_6H_3$) could be detected. After heating to 40 °C for 24 h another ^{1}H spectrum was recorded. No protonation of **DMAQ** was detected (See chapter III: Additional Reactions and Associated NMR Spectra). This was taken as evidence that the pK_a of **DMAQ** < 0.1 (MeCN, see pK_a determination of $[H(OEt_2)_2][BAr^F_4]$ in chapter IV). Therefore, $\Delta G°_{PT} \gtrsim 41$ kcal mol^{-1} for a PT reaction between **DMAQ** and **4–H**, ruling out the thermodynamic feasibility of an PT–ET pathway.

Consequently, the PCET reaction between **4–H** and **DMAQ** likely follows a concerted proton-electron transfer (CPET) mechanism.

Thermochemistry

The ΔG values were calculated according to Hess' law using the Bordwell equation[28]:

$$BDFE = 23.06E° + 1.37pK_a + C_G \qquad \text{(S12)}$$

Using equation S12, the **BDFE$_{O–H}$** values were calculated using the parameters summarized in Table S2.

Table S2. Overview of the thermochemical data reported.

**Overall Expression**	**pK_a** (MeCN)	**$E°$** (V vs Fc/Fc$^+$, MeCN)	**C_G (MeCN)**[29]	**BDFE$_{O–H}$** (MeCN)
**4–H → 4**	7.1 (**4–H⁺**)	–0.61 (E_i[**4–H⁺**/**4–H**])	52.6	48.3
**4–H → 4**	30 (**4–H**)[a]	–1.94 ($E°$[**4**/**4⁻**])	52.6	48.3
**4–H⁺ → 4⁺**	7.1 (**4–H⁺**)	+0.68 ($E°$[**4⁺**/**4**])	52.6	78.0
**4–H⁺ → 4⁺**	4.9 (**4–H²⁺**)[b]	+0.81 ($E°$[**4–H²⁺**/**4–H⁺**])	52.6	78.0

[a]: Calculated using Eq. S12 using the experimentally determined value of BDFE = 48.3 kcal mol^{-1} and $E°$ = –1.94 V with C_G = 52.6.
[b]: Calculated using Eq. S12 using the experimentally determined value of BDFE = 78.0 kcal mol^{-1} and $E°$ = +0.81 V with C_G = 52.6.

VI. Electrochemical Experiments and Characterization

Compound **4** in MeCN:

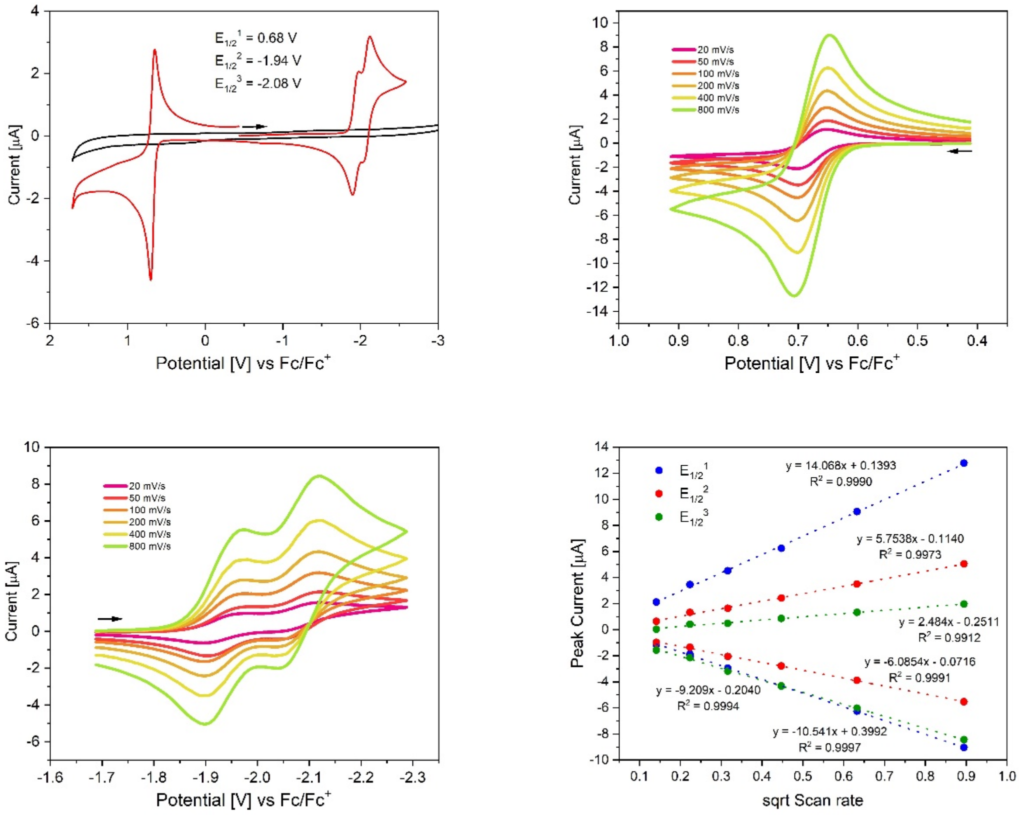

Figure S15. Cyclic voltammogram (top left), and scan rate study (top right, bottom) of **4** (1.0 mM) with 0.10 M [(*n*-Bu)$_4$N][PF$_6$] as supporting electrolyte in MeCN. Referenced against Fc/Fc$^+$. The full scan (red) was recorded at 100 mV/s and is shown against the background (black) of 0.10 M [(*n*-Bu)$_4$N][PF$_6$] in MeCN.

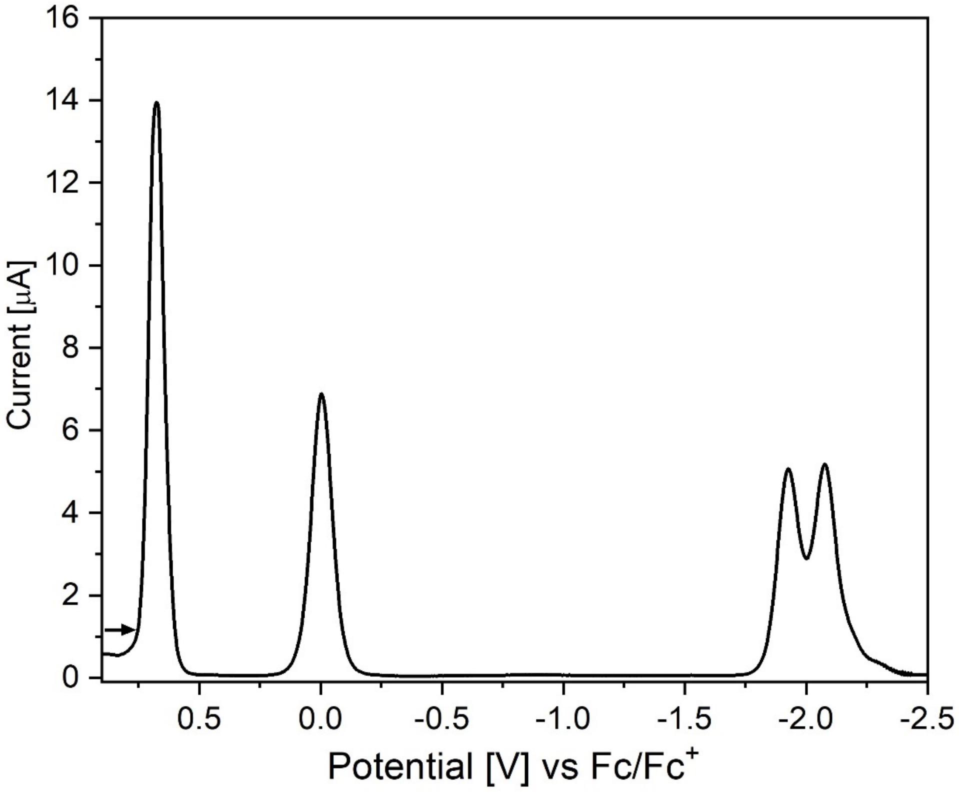

Figure S16. Square-wave voltammetry of **4** (1.0 mM) in MeCN in the presence of Ferrocene (1.0 mM).

Compound **4** in DME:

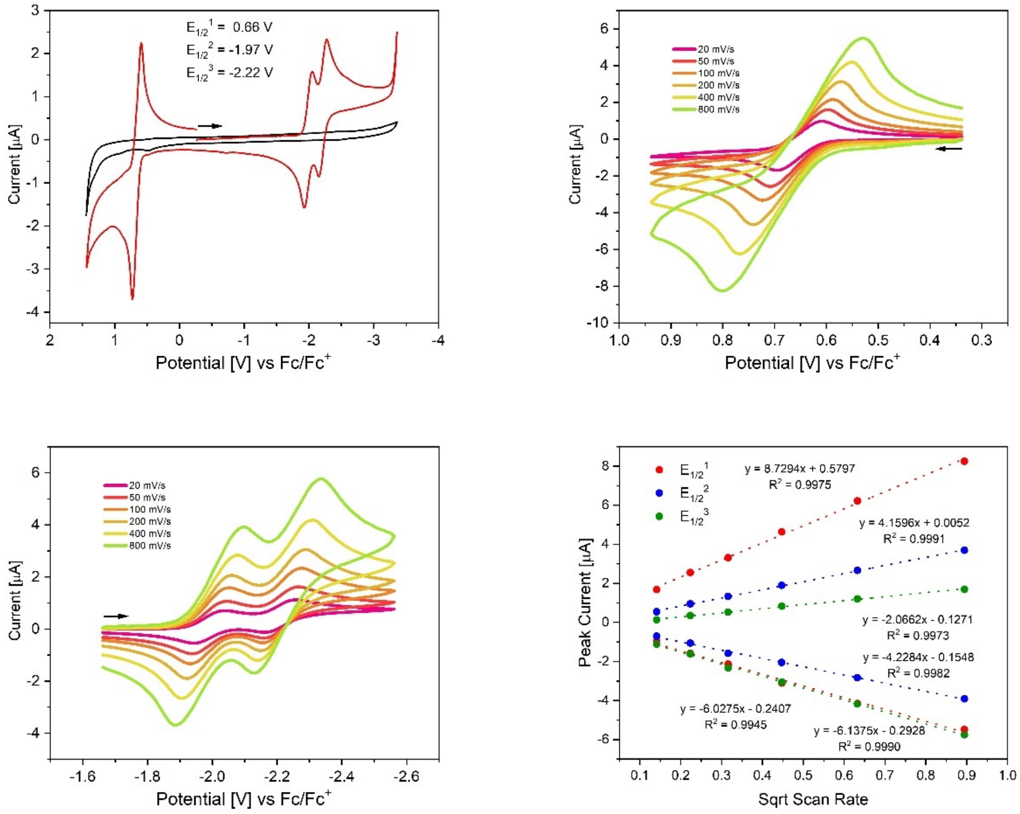

Figure S17. Cyclic voltammogram (top left), and scan rate study (top right, bottom) of **4** (1.0 mM) with 0.10 M [(*n*-Bu)$_4$N][PF$_6$] as supporting electrolyte in DME. Referenced against Fc/Fc$^+$. The full scan (red) was recorded at 100 mV/s and is shown against the background (black) of 0.10 M [(*n*-Bu)$_4$N][PF$_6$] in DME.

Compound **4** in DFB:

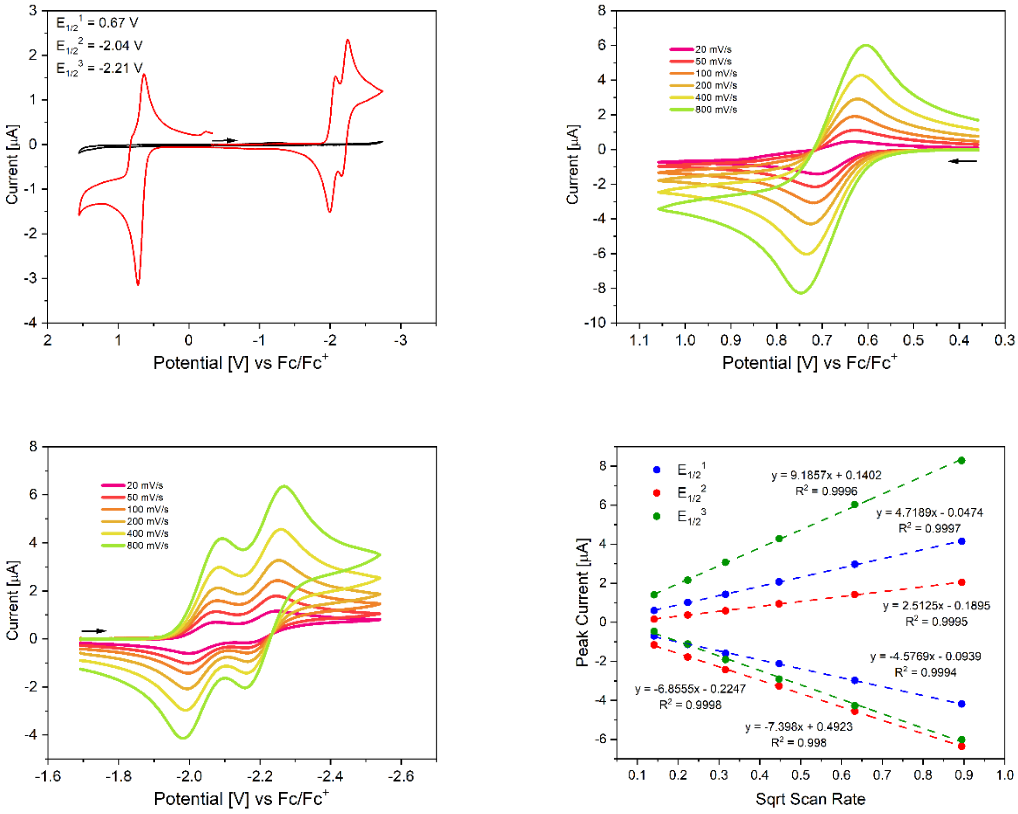

Figure S18. Cyclic voltammogram (top left), and scan rate study (top right, bottom) of **4** (1.0 mM) with 0.10 M [(*n*-Bu)$_4$N][PF$_6$] as supporting electrolyte in DFB. Referenced against Fc/Fc$^+$. The full scan (red) was recorded at 100 mV/s and is shown against the background (black) of 0.10 M [(*n*-Bu)$_4$N][PF$_6$] in DFB.

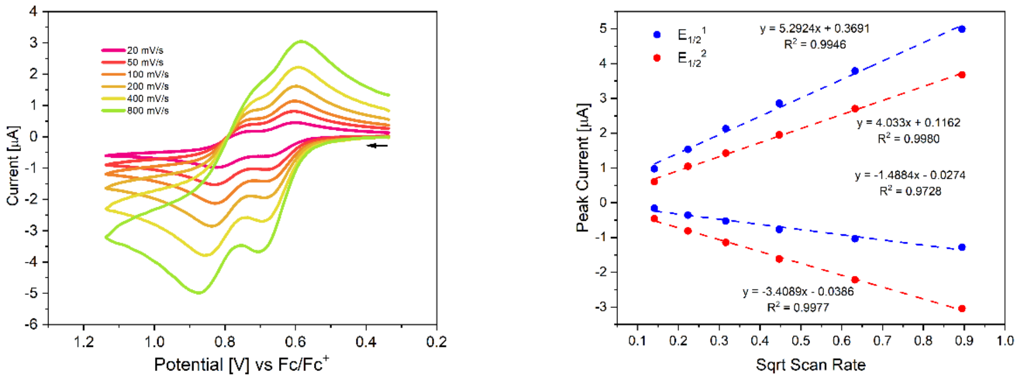

Figure S19. Scan rate study of **4** (1.0 mM) with 0.10 M [(*n*-Bu)$_4$N][Al(OR^F)$_4$] as supporting electrolyte in DFB. Referenced against Fc/Fc$^+$. At higher scan rates the second oxidation shows quasi-reversible character. The redox potentials were determined to be $E_{1/2}^1$ = 0.79 V and $E_{1/2}^2$ = 0.64 V vs Fc/Fc$^+$.

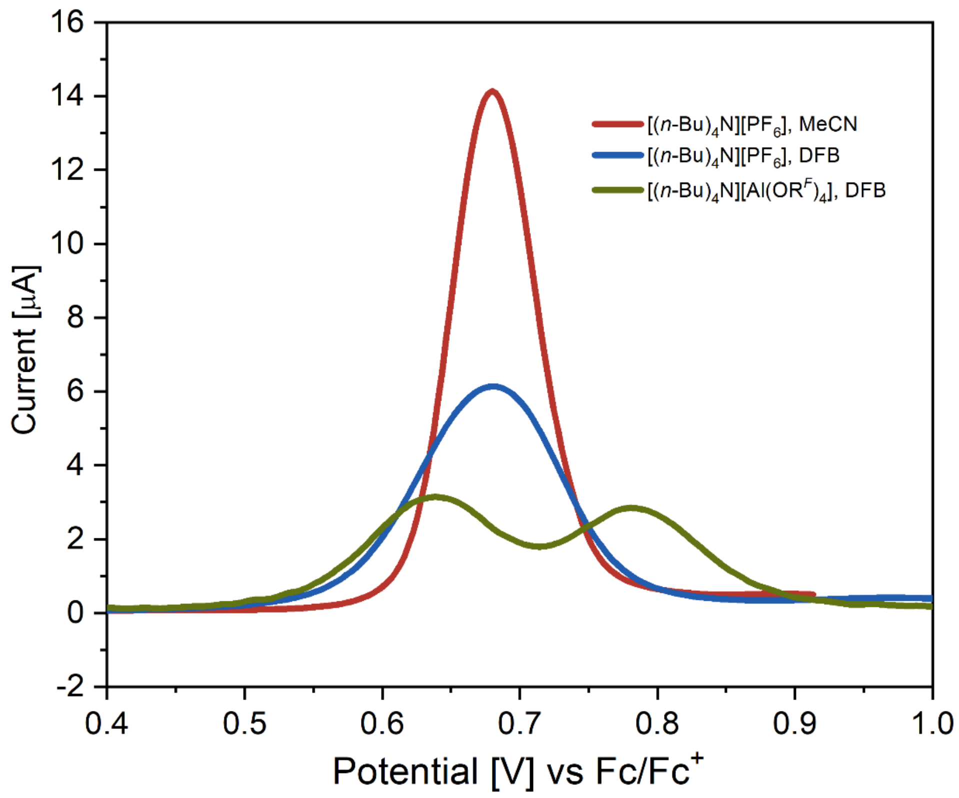

Figure S20. Square-wave voltammetry comparison of the oxidative feature of **4** (1.0 mM) under various conditions. The data was collected in MeCN solvent using 0.1 M [(*n*-Bu)$_4$N][PF$_6$] supporting electrolyte (red trace), in DFB solvent using 0.1 M [(*n*-Bu)$_4$N][PF$_6$] supporting electrolyte (blue trace) and in DFB solvent using 0.1 M [(*n*-Bu)$_4$N][Al(OR^F)$_4$] supporting electrolyte (green trace). The separation of the two-electron feature into successive 1-electron events is clearly visible upon collecing the electrochemical data in non-coordinating DFB solvent using the weakly coordinating [(*n*-Bu)$_4$N][Al(OR^F)$_4$] supporting electrolyte.

Compound **[K(THF)$_2$(4)]$_2$** in DME:

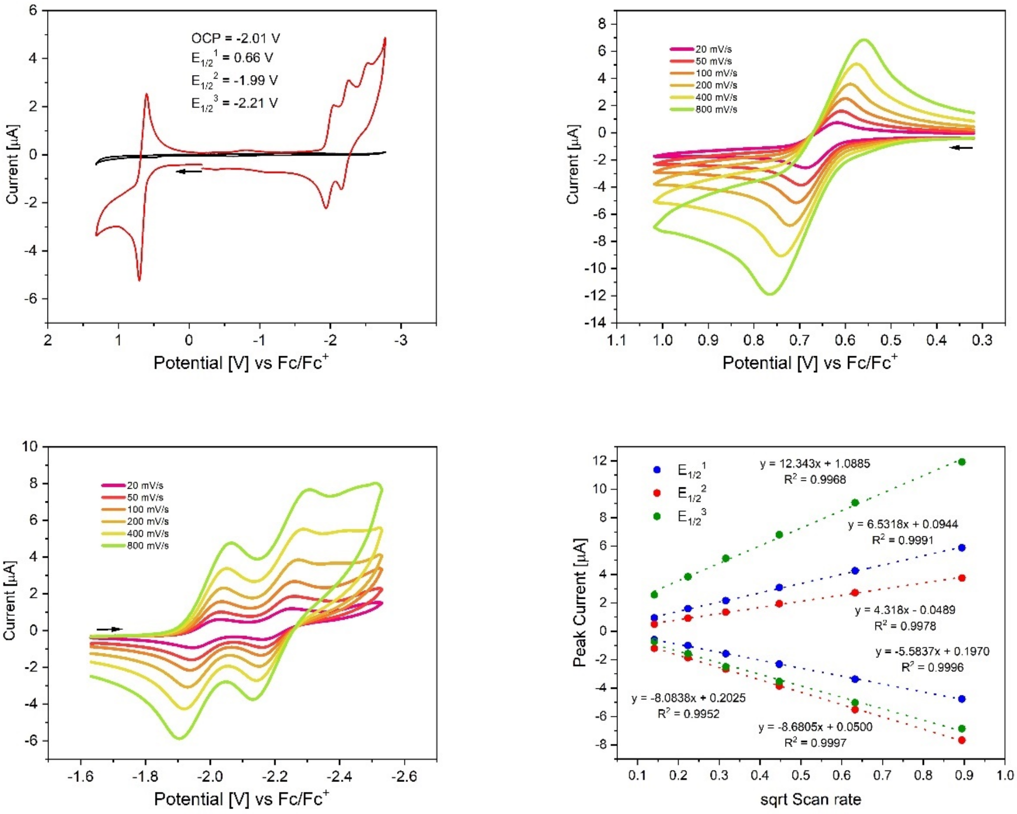

Figure S21. Cyclic voltammogram (top left), and scan rate study (top right, bottom) of **[K(THF)$_2$(4)]$_2$** (1.0 mM) with 0.10 M [(*n*-Bu)$_4$N][PF$_6$] as supporting electrolyte in DME. Referenced against Fc/Fc$^+$. The full scan (red) was recorded at 100 mV/s and is shown against the background (black) of 0.10 M [(*n*-Bu)$_4$N][PF$_6$] in DME.

Compound **4**$^{–}$ in DME:

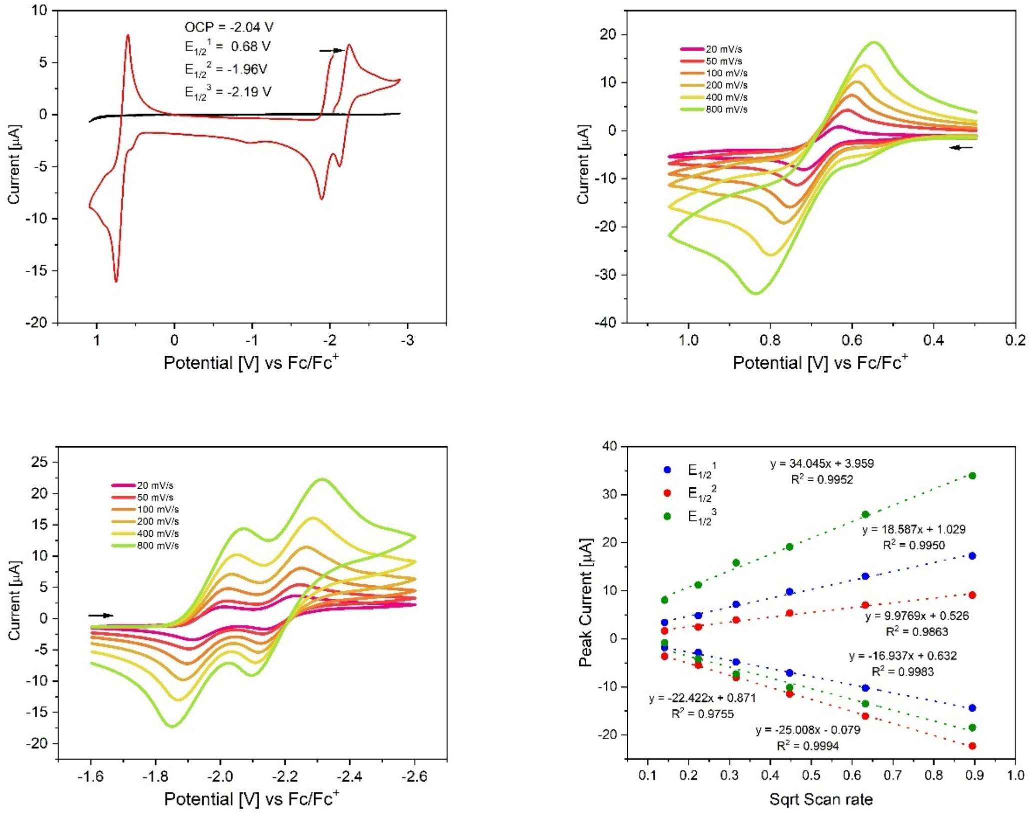

Figure S22. Cyclic voltammogram (top left), and scan rate study (top right, bottom) of **4**$^{–}$ (5.0 mM) with 0.20 M [(n-Bu)$_4$N][PF$_6$] as supporting electrolyte in DME. Referenced against Fc/Fc$^+$. The full scan (red) was recorded at 100 mV/s and is shown against the background (black) of 0.10 M [(n-Bu)$_4$N][PF$_6$] in DME.

Compound [**MG**][Al(OR^F)$_4$] in MeCN:

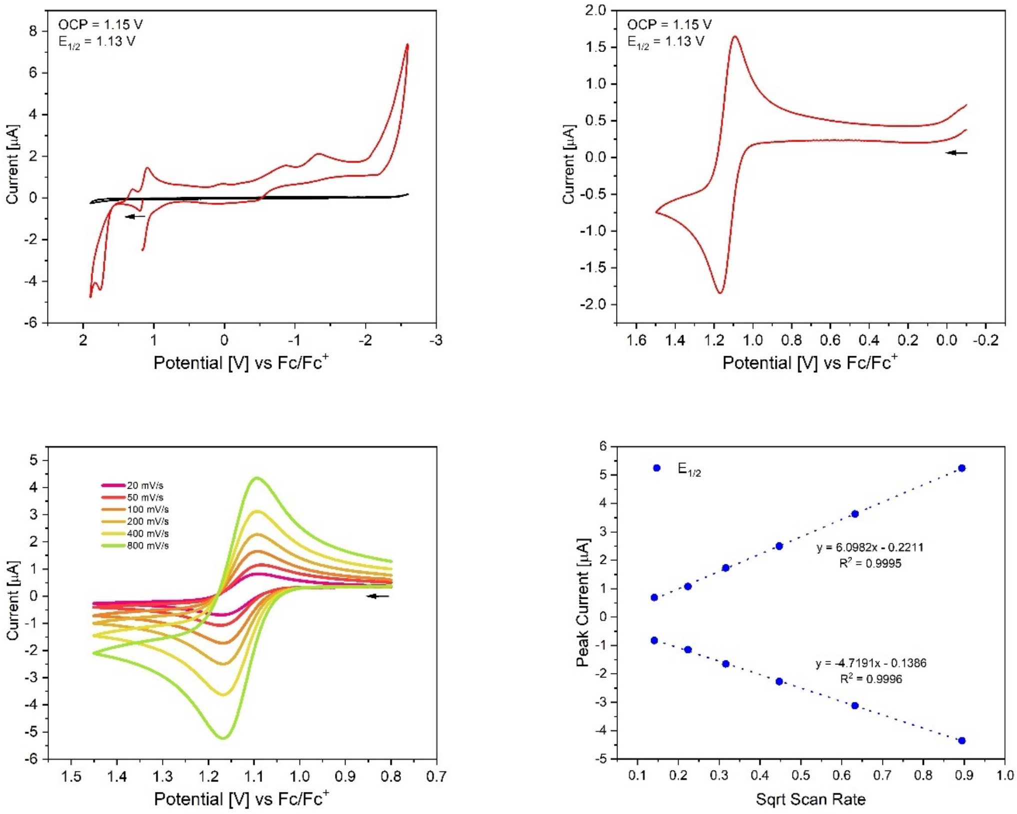

Figure S23. Cyclic voltammogram (top left, right), scan rate study (bottom left) of [**MG**][Al(OR^F)$_4$] (1.0 mM) with 0.10 M [(n-Bu)$_4$N][PF$_6$] as supporting electrolyte in MeCN. Referenced against Fc/Fc$^+$. The full scan (red) was recorded at 100 mV/s and is shown against the background (black) of 0.10 M [(n-Bu)$_4$N][PF$_6$] in MeCN. The oxidation potential of Magic Green AlORF was determined to be 1.13 V vs Fc/Fc$^+$ in MeCN.

Compound 4^+ in MeCN:

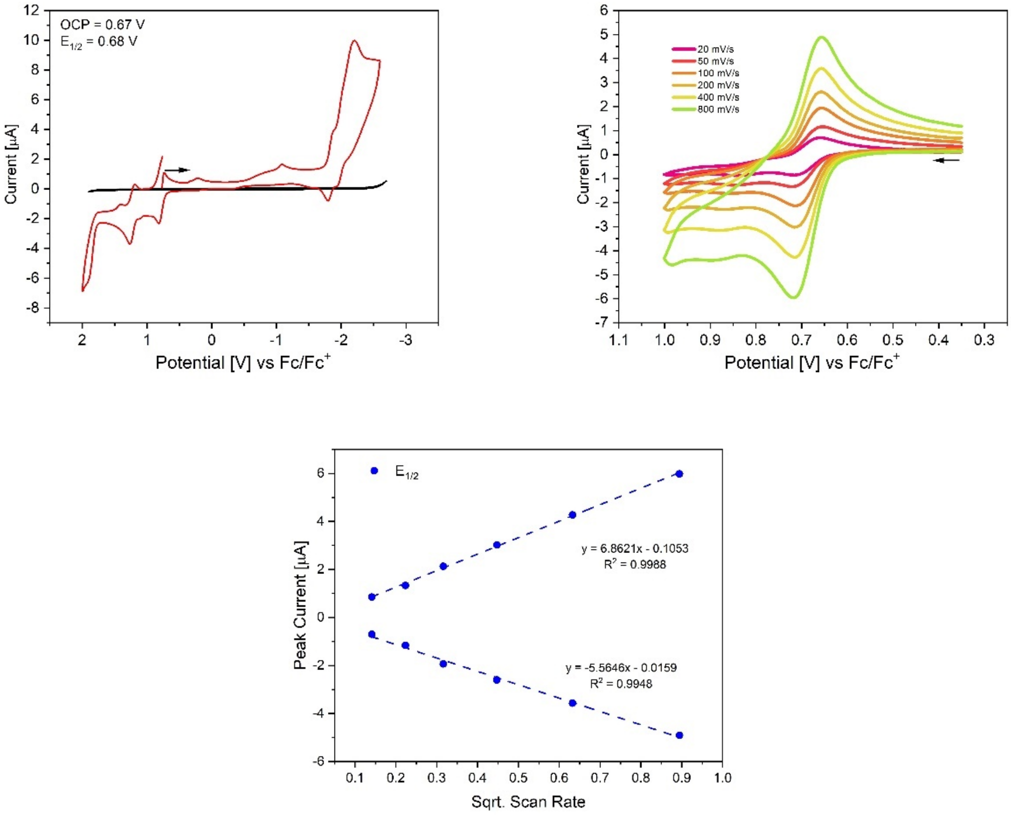

Figure S24. Cyclic voltammogram (top left), and scan rate study (top right, bottom) of *in-situ* generated **4^+** (1.0 mM) with 0.10 M [(*n*-Bu)$_4$N][PF$_6$] as supporting electrolyte in MeCN. Referenced against Fc/Fc$^+$. The full scan (red) was recorded at 100 mV/s and is shown against the background (black) of 0.10 M [(*n*-Bu)$_4$N][PF$_6$] in MeCN. The reversible redox event at 1.13 V corresponds to the by-product (2,4-Br$_2$-Ph)$_3$N.

Compound **4–H⁺** in MeCN

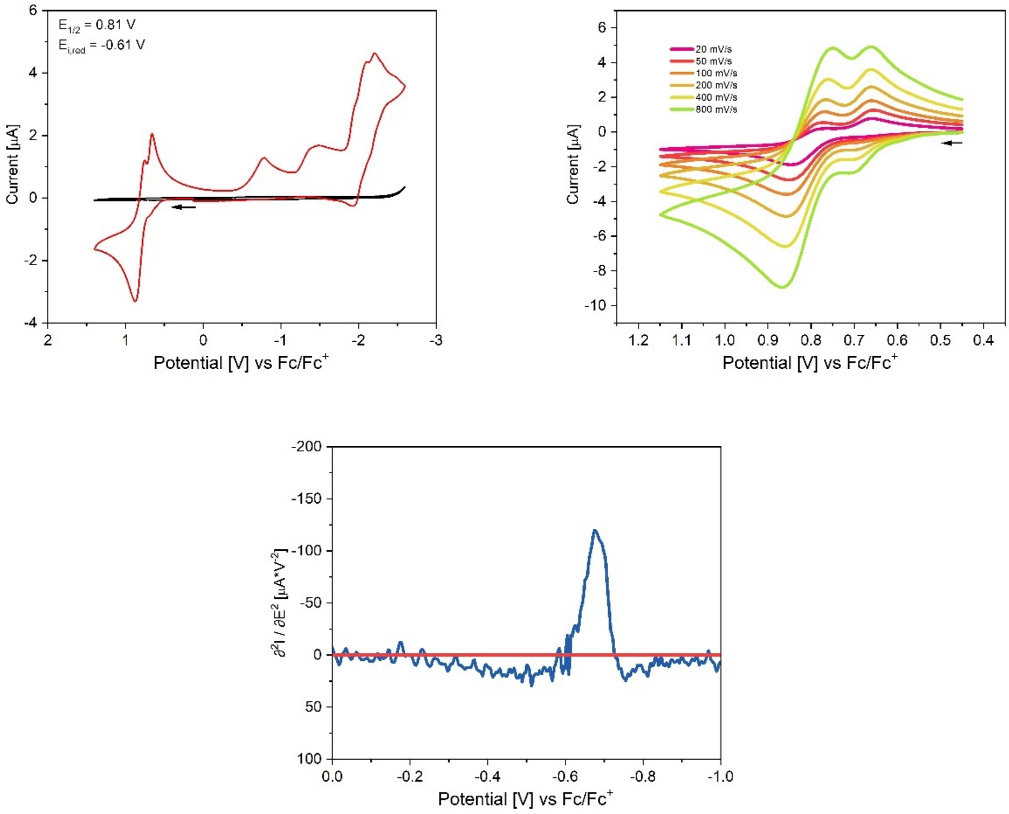

Figure S25. Cyclic voltammogram (top left), scan rate study (top right) of **4–H⁺** (1.0 mM) with 0.10 M [(n-Bu)$_4$N][PF$_6$] as supporting electrolyte in MeCN. Referenced against Fc/Fc⁺. The full scan (red) was recorded at 100 mV/s and is shown against the background (black) of 0.10 M [(n-Bu)$_4$N][PF$_6$] in MeCN. The reduction potential was determined by taking the inflection point of the irreversible reduction (bottom).

5,5''-SMe-TTH (**6**) in DME

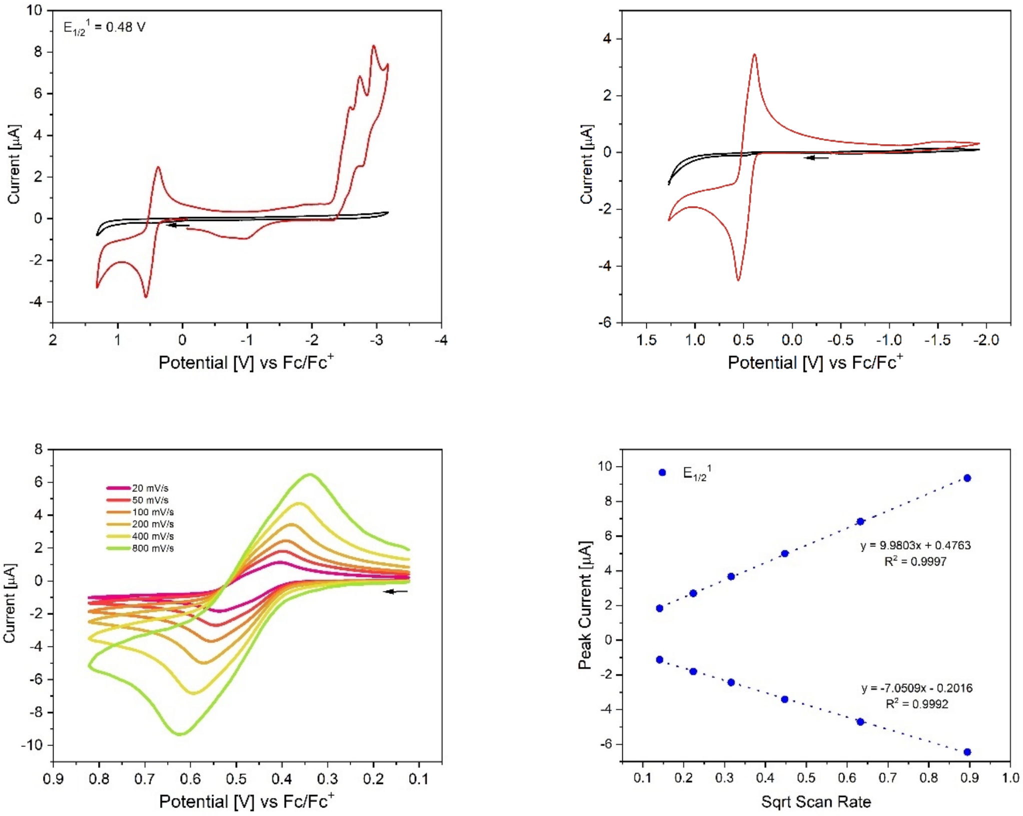

Figure S26. Cyclic voltammogram (top), scan rate study (bottom) of **6** (1.0 mM) with 0.10 M [(*n*-Bu)$_4$N][PF$_6$] as supporting electrolyte in DME. Referenced against Fc/Fc$^+$. The full scan (red) was recorded at 100 mV/s and is shown against the background (black) of 0.10 M [(*n*-Bu)$_4$N][PF$_6$] in DME. The broad oxidation event at ca. –1 V corresponds to unidentified products of the irreversible reduction features observed in the CV of **6** (top left). When the return scan is set at less negative potentials, this feature is no longer observed (top right).

5,5''-SMe-TTH (**6**) in MeCN

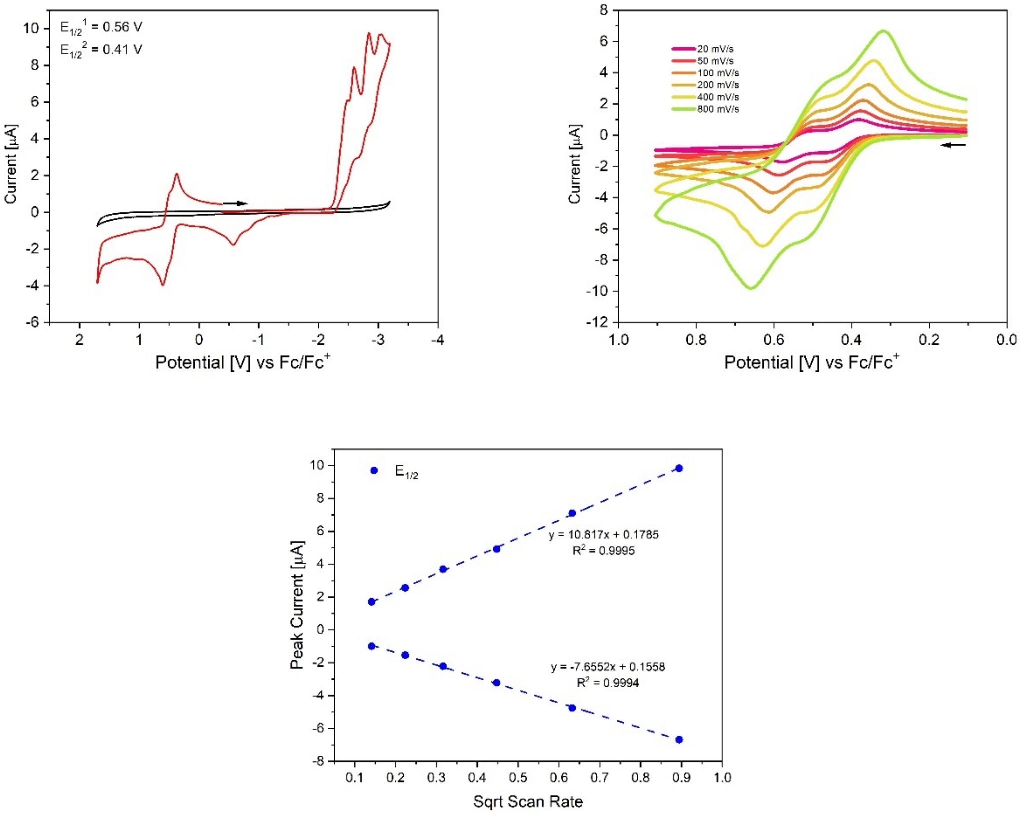

Figure S27. Cyclic voltammogram (top left), and scan rate study (top right, bottom) of **6** (1.0 mM) with 0.10 M [(*n*-Bu)$_4$N][PF$_6$] as supporting electrolyte in MeCN. Referenced against Fc/Fc+. The full scan (red) was recorded at 100 mV/s and is shown against the background (black) of 0.10 M [(*n*-Bu)$_4$N][PF$_6$] in MeCN.

Comparison of **4**, **5** and **6** in MeCN

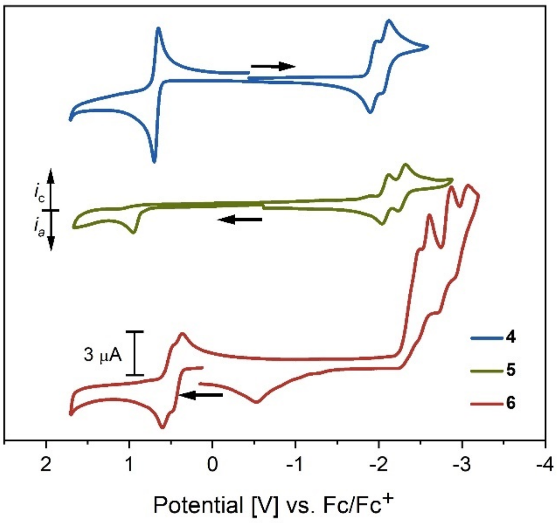

Figure S28. Cyclic Voltammograms of a 1.0 mM solution with 0.10 M [(*n*-Bu)$_4$N][PF$_6$] as supporting electrolyte in MeCN of **4**, **5** and **6**. Compound **5** is very poorly soluble in MeCN (<0.5 mM), thus a comparison in DME is shown in the main text, where **4**, **5**, and **6** are sufficiently soluble.

VII. Additional Electrochemical Data

Terthiophene (TTH) in DME

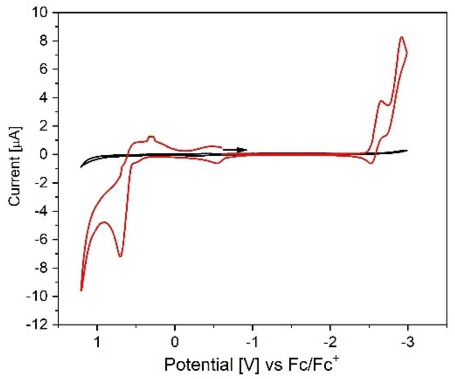

Figure S29. Cyclic voltammogram of **TTH** (1.0 mM) with 0.10 M [(n-Bu)$_4$N][PF$_6$] as supporting electrolyte in DME. Referenced against Fc/Fc+. The full scan (red) was recorded at 100 mV/s and is shown against the background (black) of 0.10 M [(n-Bu)$_4$N][PF$_6$] in DME. TTH polymerizes upon oxidation on the working electrode, thus only one scan was recorded and the electrode was thoroughly polished afterwards.

1,4-Dihydroquinone (**Q–H₂**) in MeCN

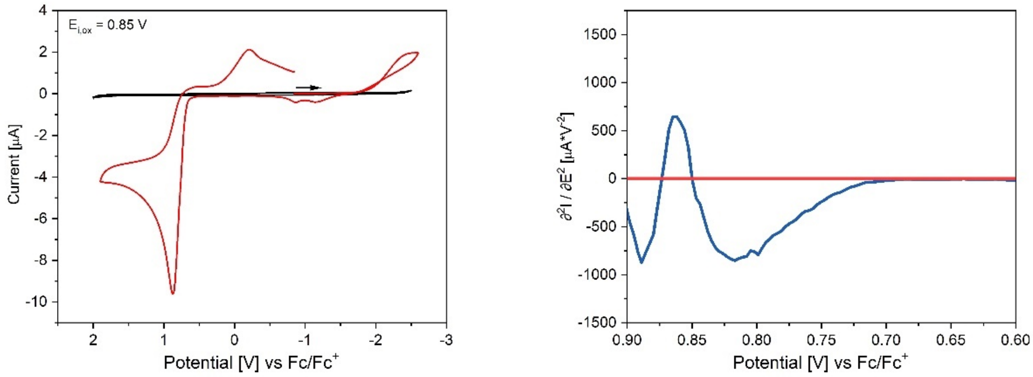

Figure S30. Cyclic voltammogram (left) of **Q–H$_2$** (1.0 mM) with 0.10 M [(n-Bu)$_4$N][PF$_6$] as supporting electrolyte in MeCN. Referenced against Fc/Fc+. The full scan (red) was recorded at 100 mV/s and is shown against the background (black) of 0.10 M [(n-Bu)$_4$N][PF$_6$] in MeCN. The oxidation potential was determined by taking the inflection point of the irreversible oxidation (right).

2,3-Dimethylanthraquinone **(DMAQ)** in MeCN:

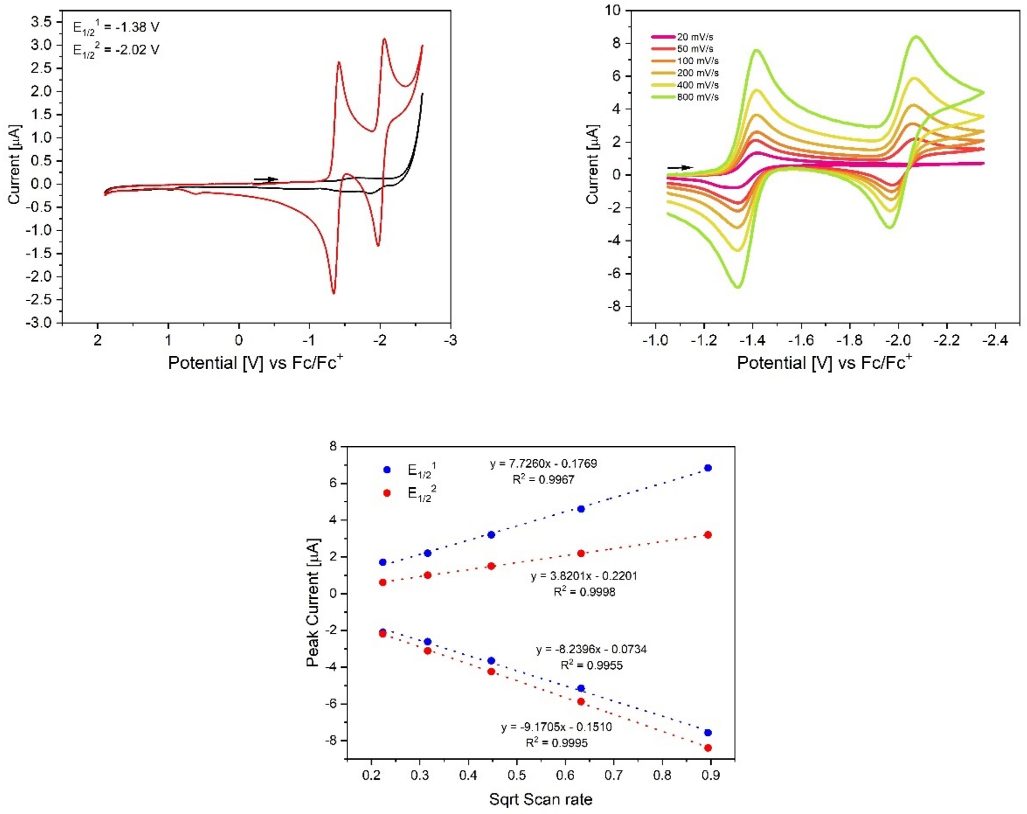

Figure S31. Cyclic voltammogram (top left), and scan rate study (top right) of **DMAQ** (1.0 mM) with 0.10 M [(n-Bu)$_4$N][PF$_6$] as supporting electrolyte in MeCN. Referenced against Fc/Fc+. The full scan (red) was recorded at 100 mV/s and is shown against the background (black) of 0.10 M [(n-Bu)$_4$N][PF$_6$] in MeCN.

[H(OEt$_2$)$_2$][BAr$^F{}_4$] (Ar^F = 3,5-(CF$_3$)$_2$C$_6$H$_3$) in MeCN:

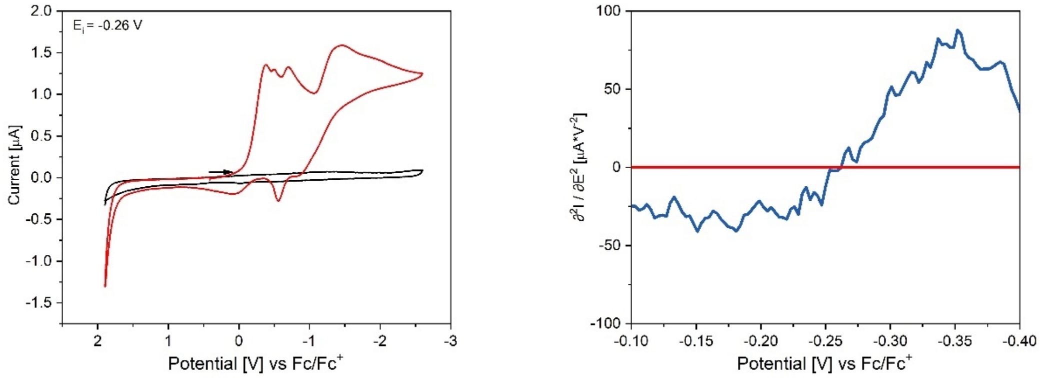

Figure S32. Cyclic voltammogram (left) of [H(OEt$_2$)$_2$][BAr$^F{}_4$] (1.0 mM) with 0.10 M [(n-Bu)$_4$N][PF$_6$] as supporting electrolyte in MeCN. Referenced against Fc/Fc+. The full scan (red) was recorded at 100 mV/s and is shown against the background (black) of 0.10 M [(n-Bu)$_4$N][PF$_6$] in MeCN. The reduction potential was determined by taking the inflection point of the irreversible reduction (right).

VIII. Magnetic Moment Determination

The magnetic moments of **4**$^{+}$ and **4**$^{-}$ were determined by the Evans method[30]. Namely, each compound was carefully weighed into a scintillation vial, followed by weighing in the NMR solvent. Upon dissolution, the resulting solution was transferred into a J-Young NMR tube containing a flame-sealed capillary with ferrocene dissolved in the corresponding NMR solvent. The $\Delta\delta$ measurement for ferrocene was performed in a temperature-controlled NMR probe at 298 K. This procedure was repeated three times and the average of these three measurements is reported.

Table S3. Evans measurements and the corresponding effective magnetic moment of **4**$^{-}$ in THF-d^{8}.

**Trial**	μ_{eff} [μ_B]
1	1.88
2	1.87
3	1.97

Table S4. Evans measurements and the corresponding effective magnetic moment of **4**$^{+}$ in CD_3CN.

**Trial**	μ_{eff} [μ_B]
1	1.82
2	1.89
3	1.82

IX. Photophysical Data

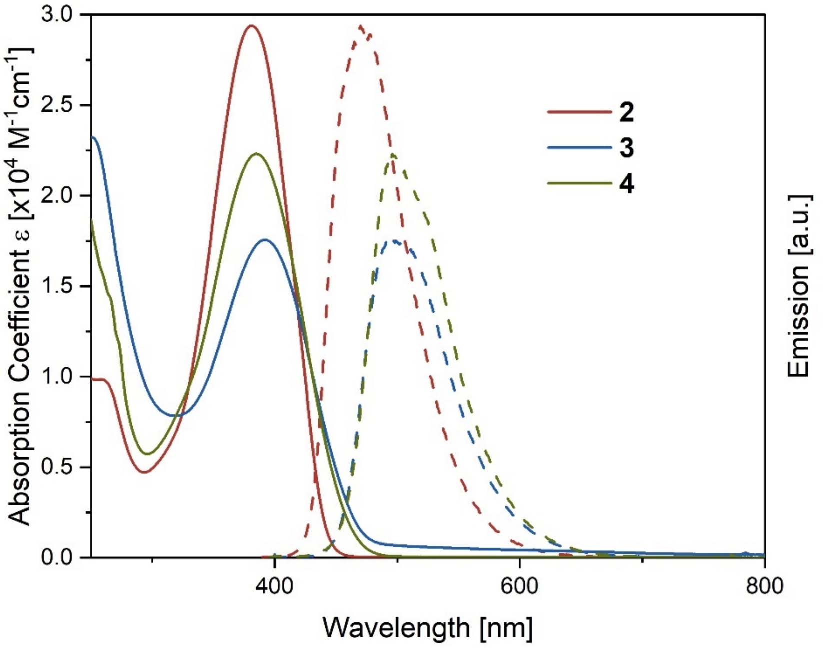

Figure S33. UV-Vis (solid lines) and emission (dashed lines) spectra of compounds **2**, **3** and **4** recorded in MeCN at room temperature.

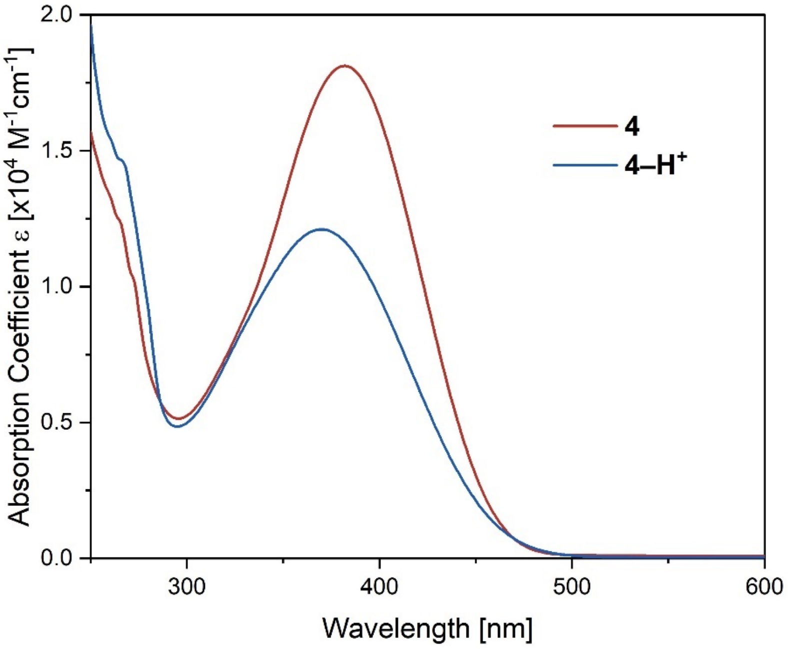

Figure S34. UV-Vis spectra of compounds **4** and **4–H**$^+$ recorded in MeCN at room temperature.

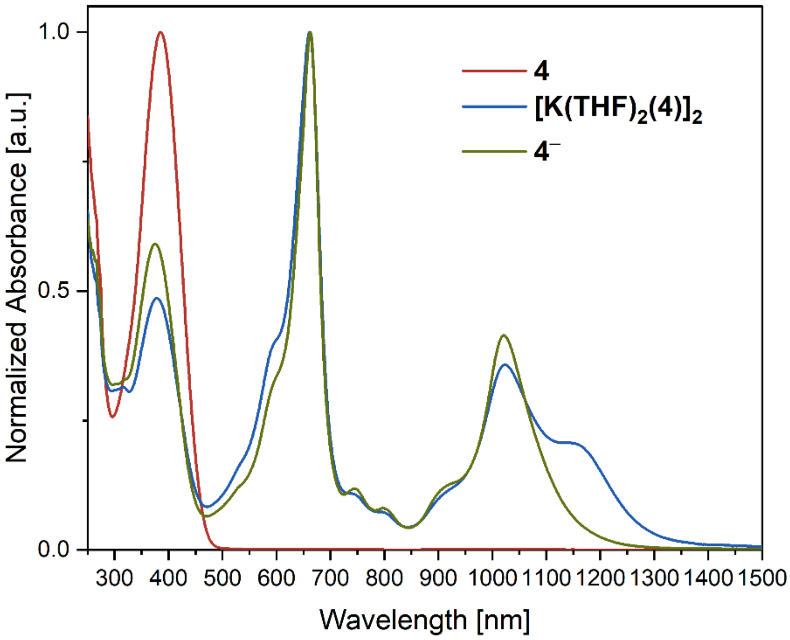

Figure S35. UV-Vis spectra of compounds **4**, **[K(THF)$_2$(4)]$_2$** and **4**$^-$ recorded in DME at room temperature.

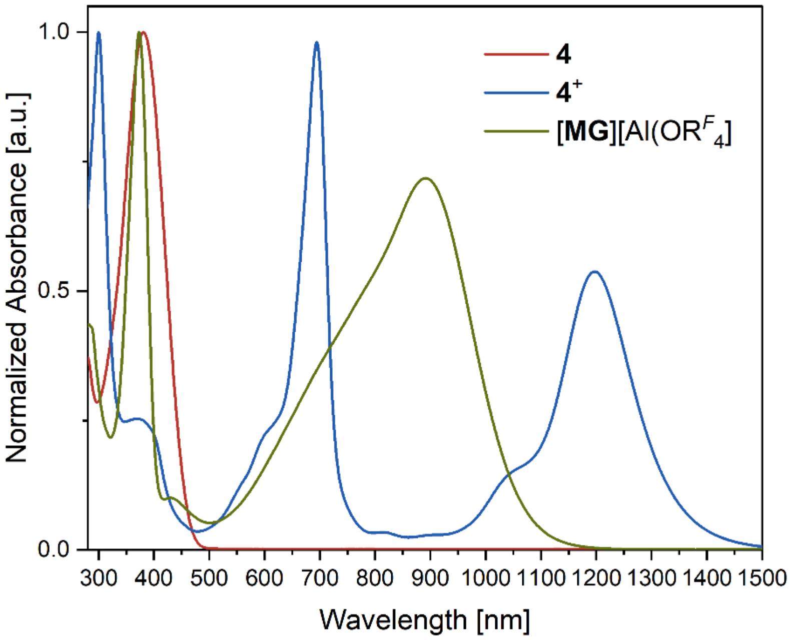

Figure S36. UV-Vis spectra of compounds **4, 4**$^+$ and [**MG**][Al(OR^F)$_4$] recorded in DFB at room temperature. For **4**$^+$ the feature at 300 nm corresponds to (2,4-Br$_2$-Ph)$_3$N generated during its synthesis.

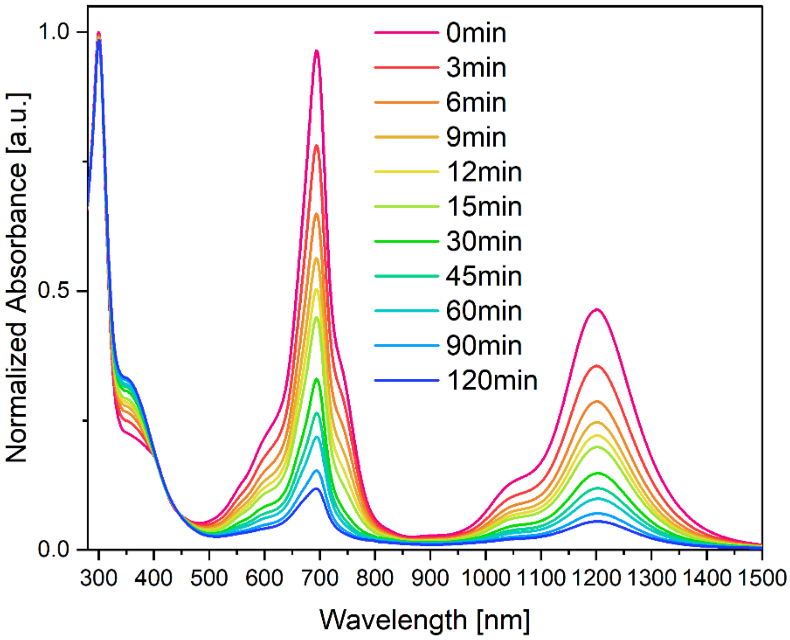

Figure S37. Degradation of **4**$^{+}$ under inert atmosphere in DFB at room temperature. The signal at 300 nm corresponds to (2,4-Br_2-Ph)$_3$N.

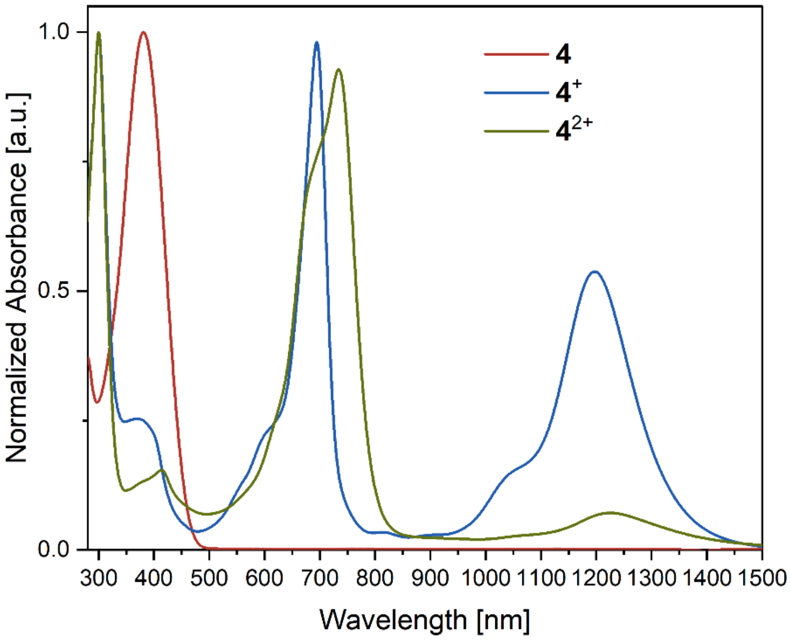

Figure S38. UV-Vis spectra of **4** and *in-situ* generated **4**$^{+}$ and **4**$^{2+}$ in DFB recorded at room temperature.

X. NMR Spectra

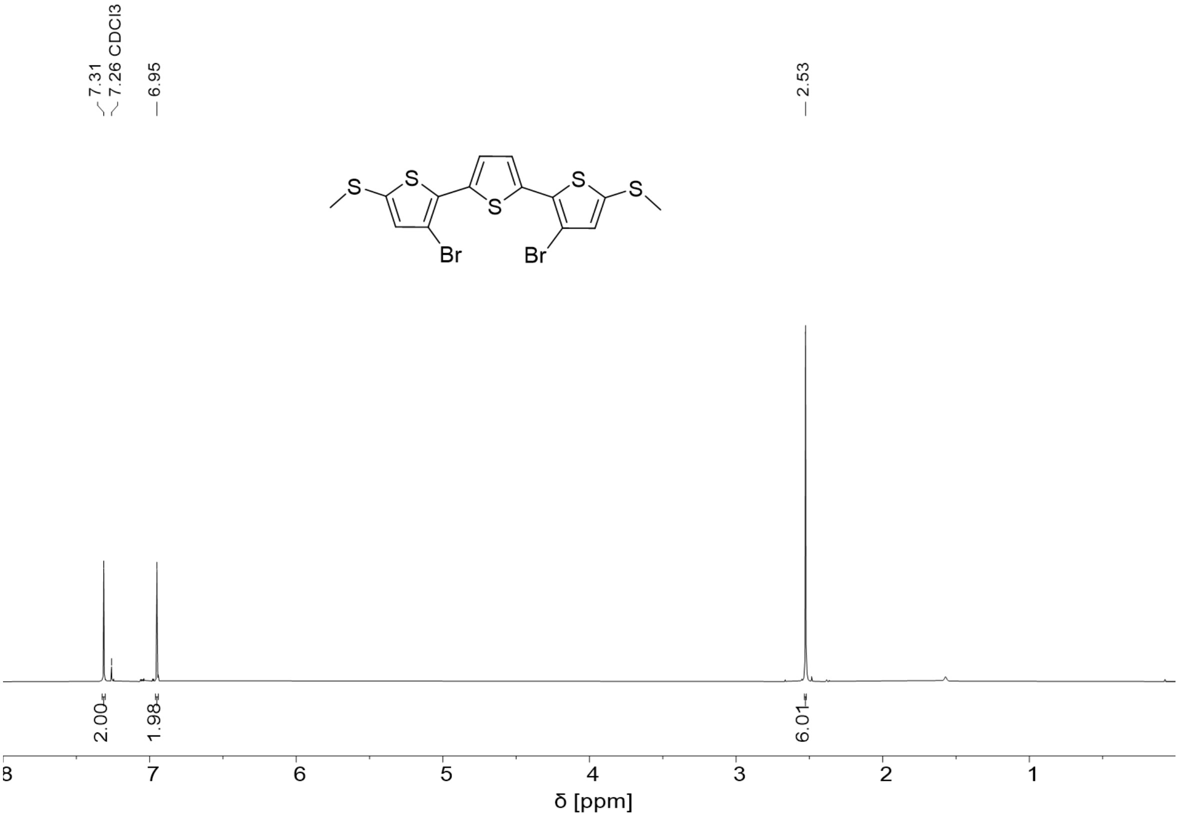

Figure S39. ^{1}H NMR (500 MHz, $CDCl_3$, 298 K) spectrum of **2**.

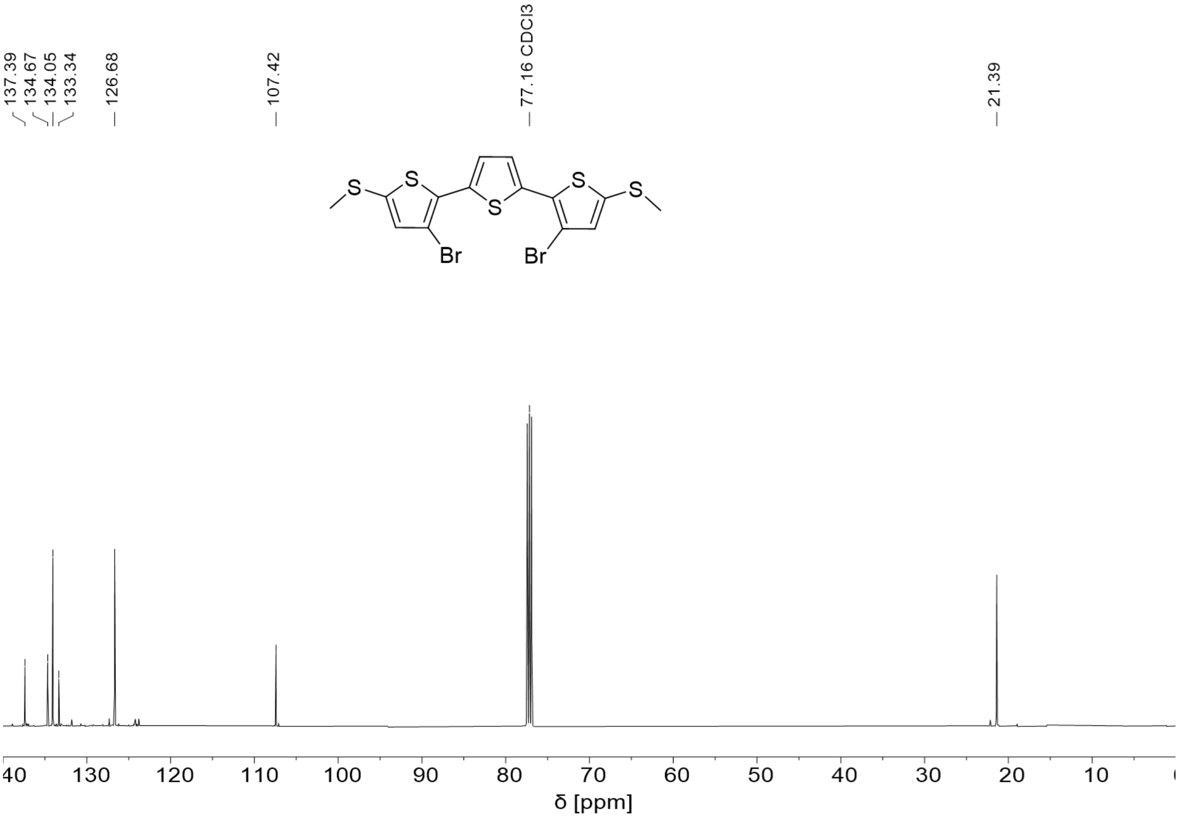

Figure S40. $^{13}C\{^{1}H\}$ NMR (126 MHz, $CDCl_3$, 298 K) spectrum of **2**.

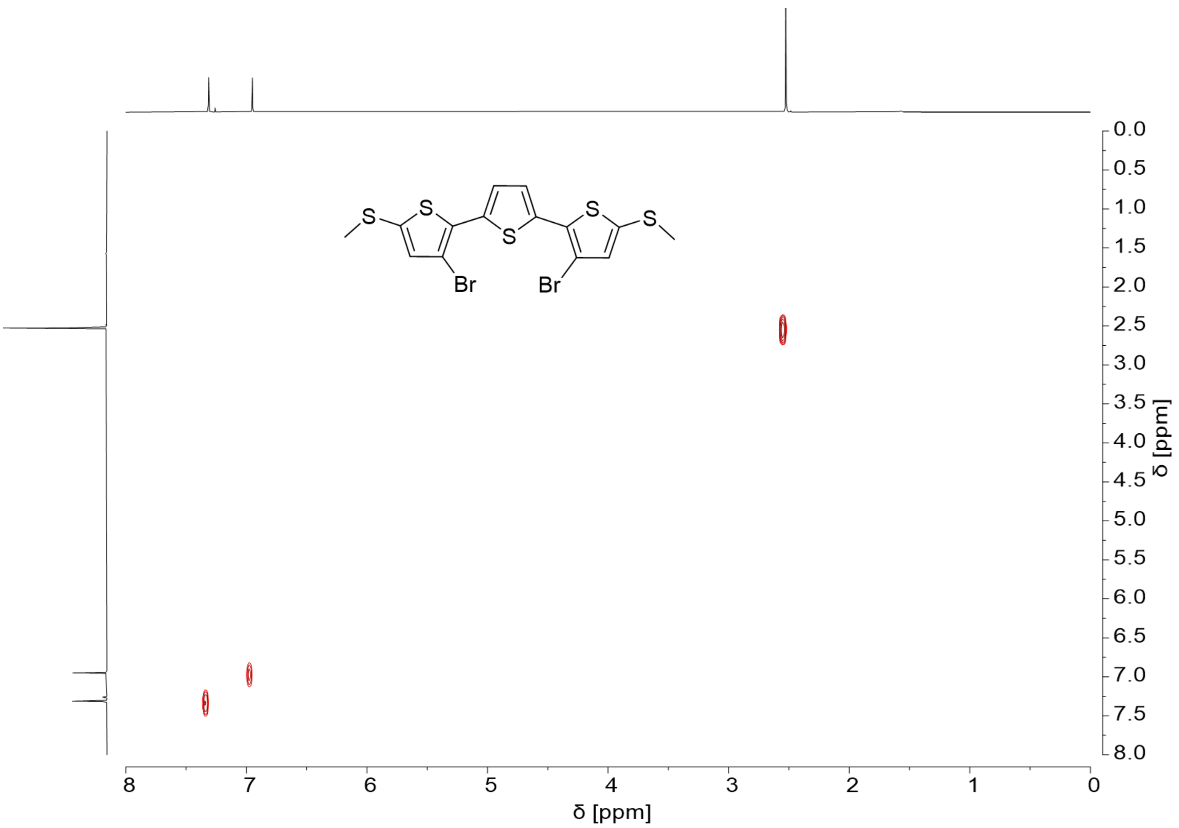

Figure S41. $^{1}H/^{1}H$ COSY NMR (500/500 MHz, $CDCl_3$, 298 K) spectrum of **2**.

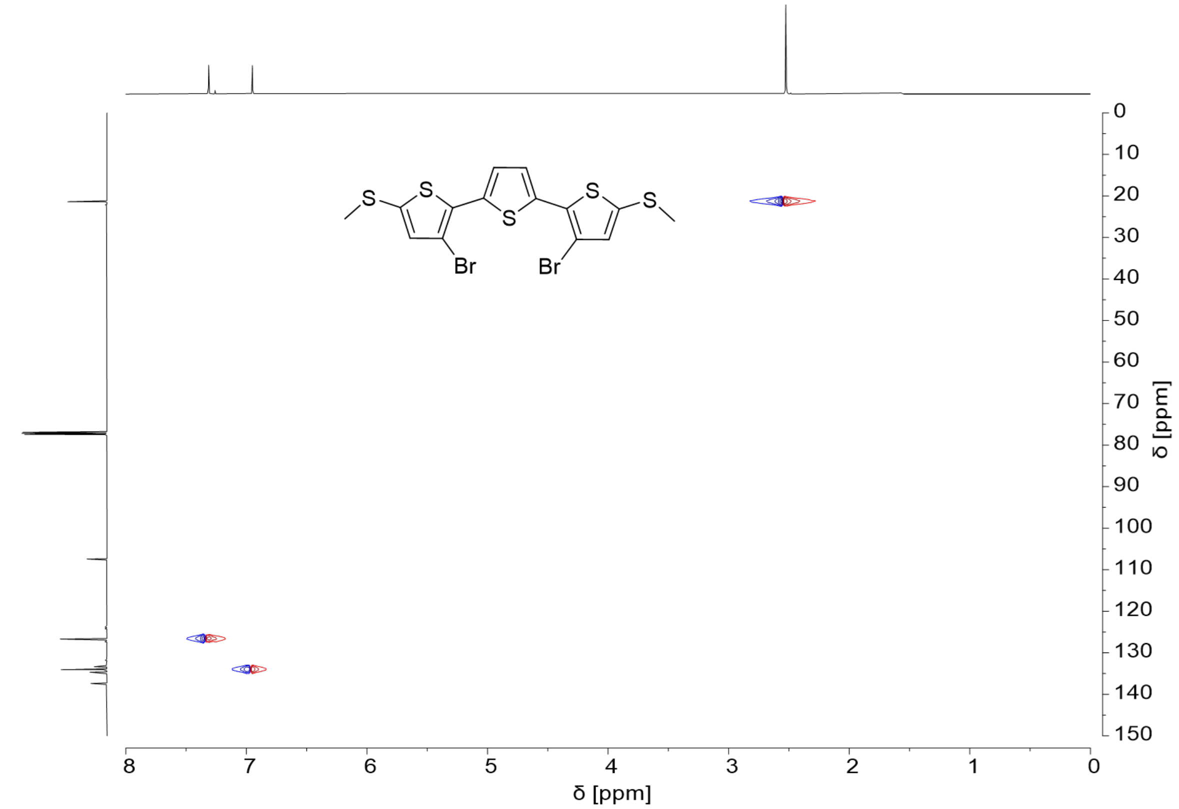

Figure S42. $^{1}H/^{13}C$ HSQC NMR (500/126 MHz, $CDCl_3$, 298 K) spectrum of **2**.

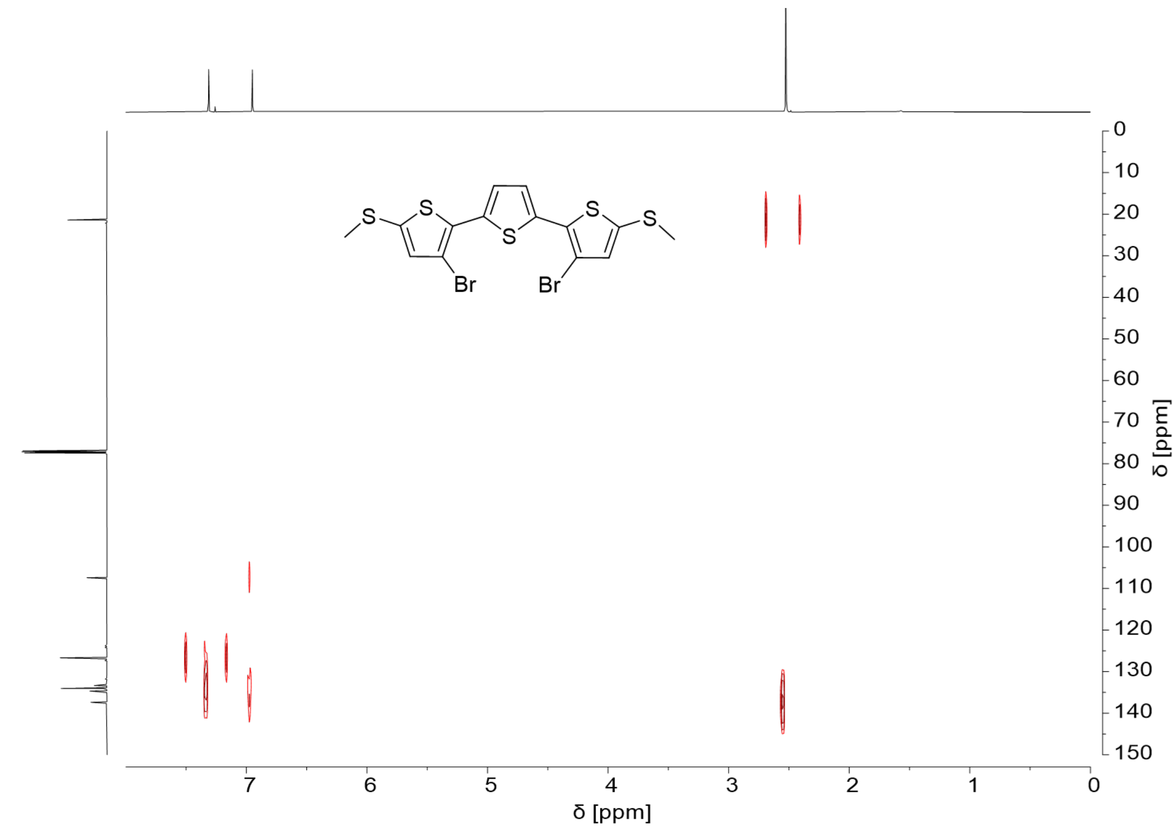

Figure S43. $^{1}H/^{13}C$ HMBC NMR (500/126 MHz, $CDCl_3$, 298 K) spectrum of **2**.

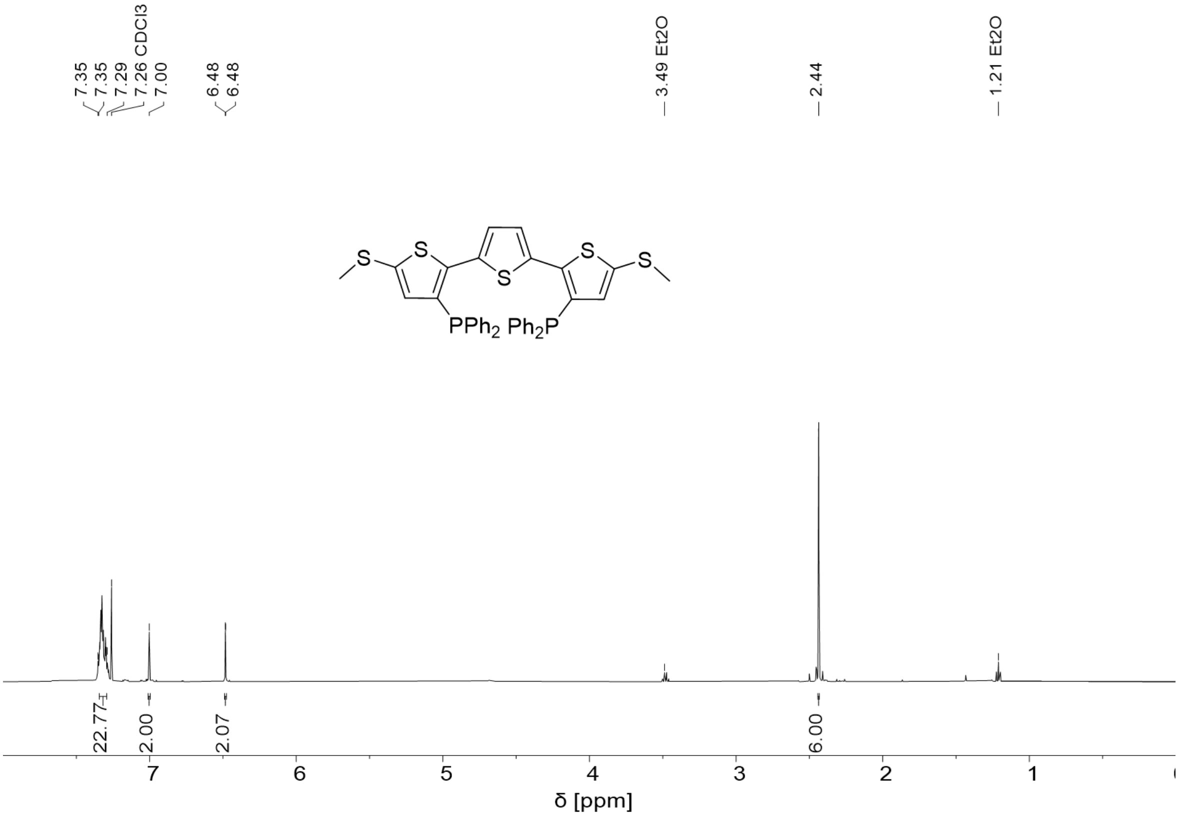

Figure S44. ^{1}H NMR (500 MHz, $CDCl_3$, 298 K) spectrum of **3**.

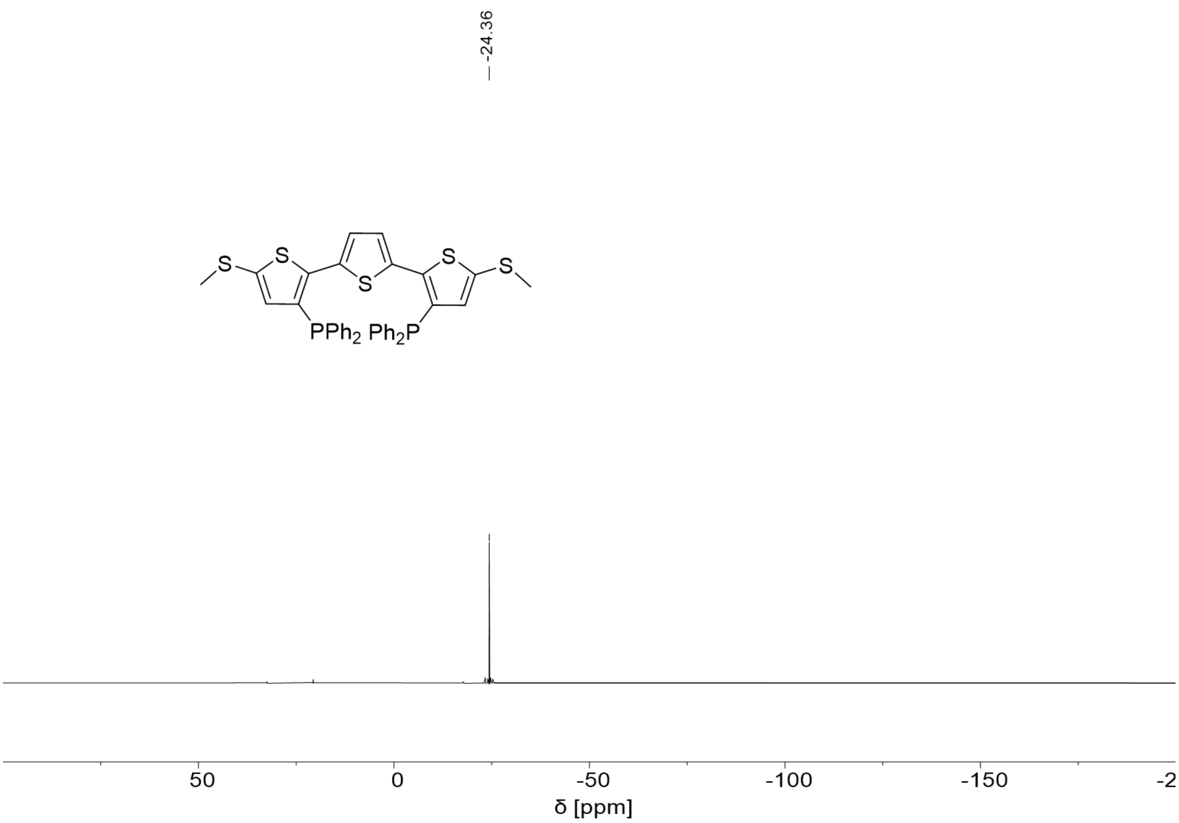

Figure S45. $^{31}P\{^{1}H\}$ NMR (202 MHz, $CDCl_3$, 298 K) spectrum of **3**.

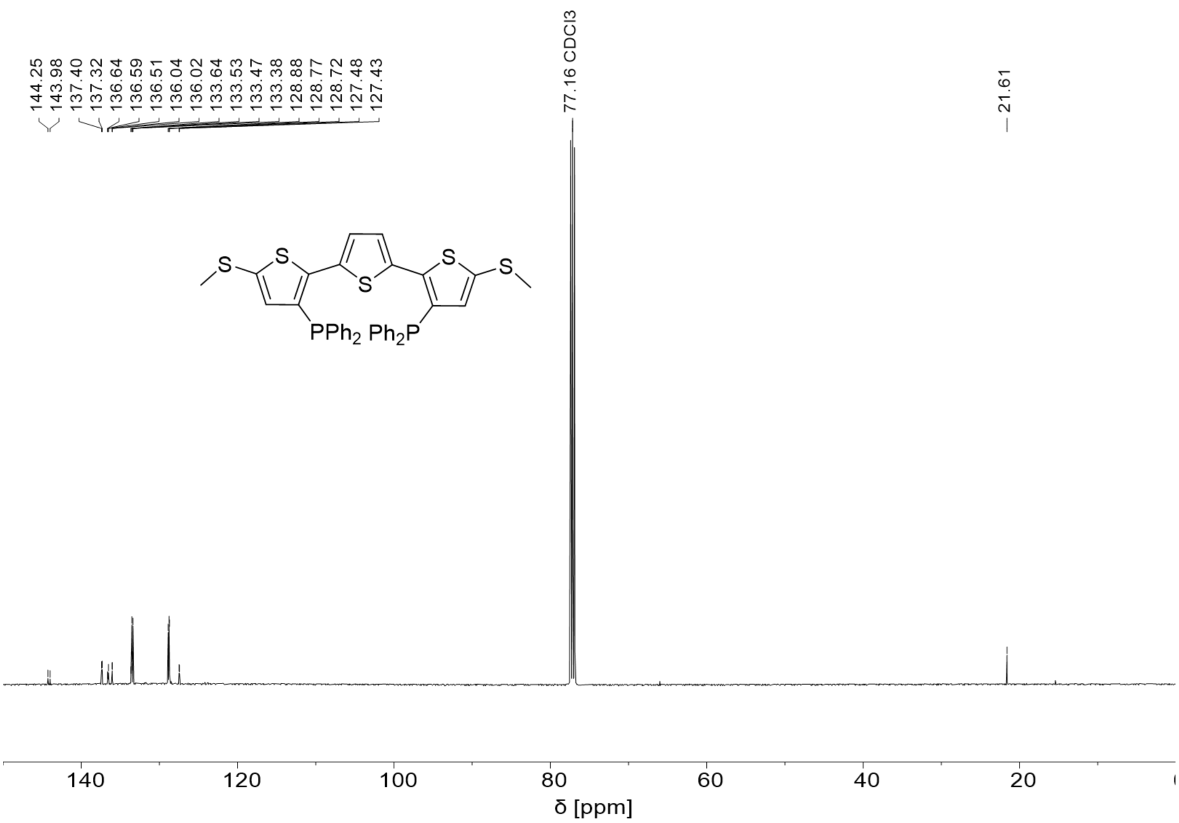

Figure S46. $^{13}C\{^{1}H\}$ NMR (126 MHz, $CDCl_3$, 298 K) spectrum of **3**.

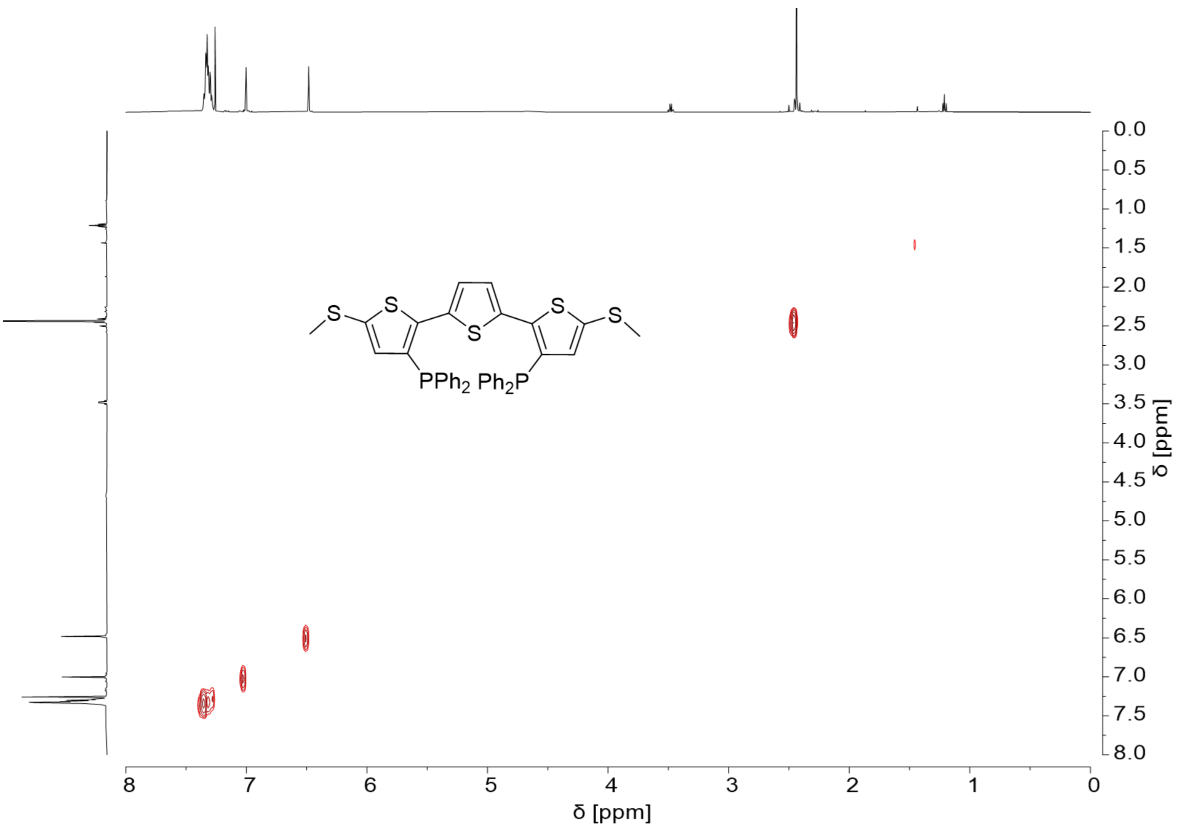

Figure S47. $^{1}H/^{1}H$ COSY NMR (500/500 MHz, $CDCl_3$, 298 K) spectrum of **3**.

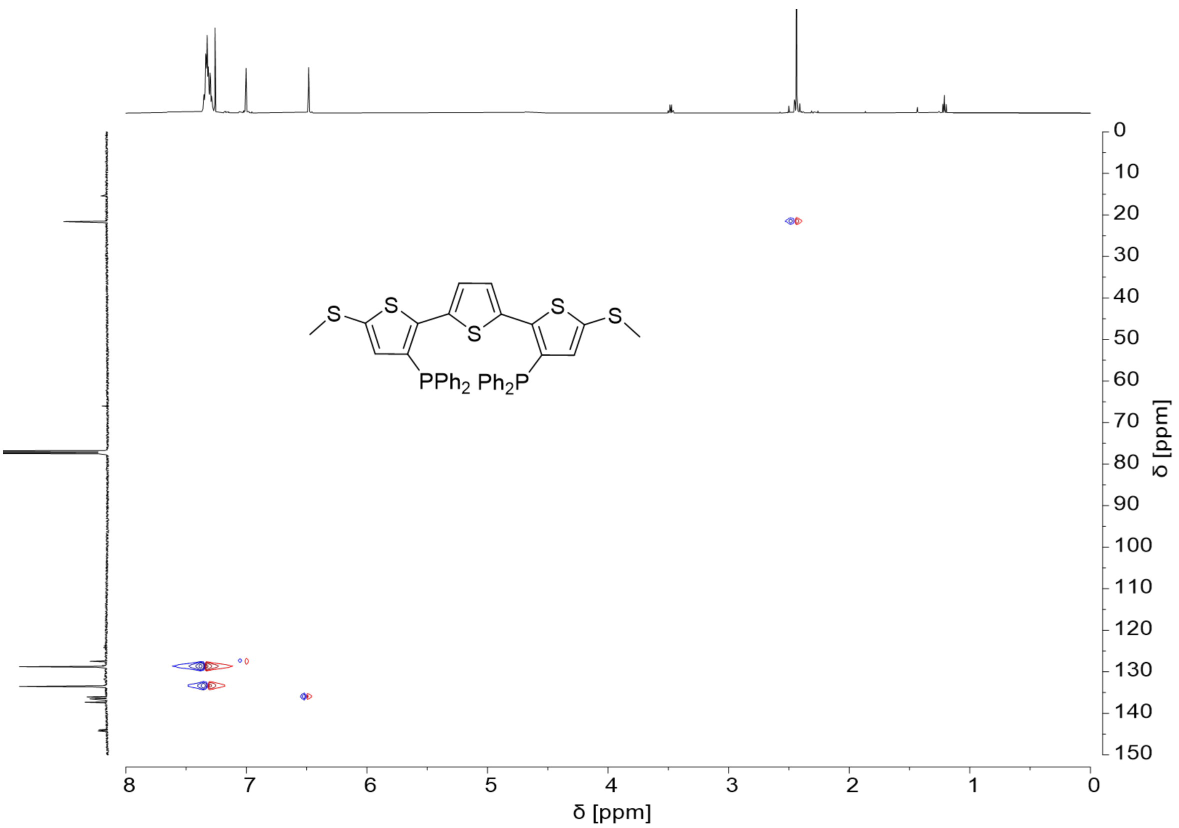

Figure S48. $^{1}H/^{13}C$ HSQC NMR (500/126 MHz, $CDCl_3$, 298 K) spectrum of **3**.

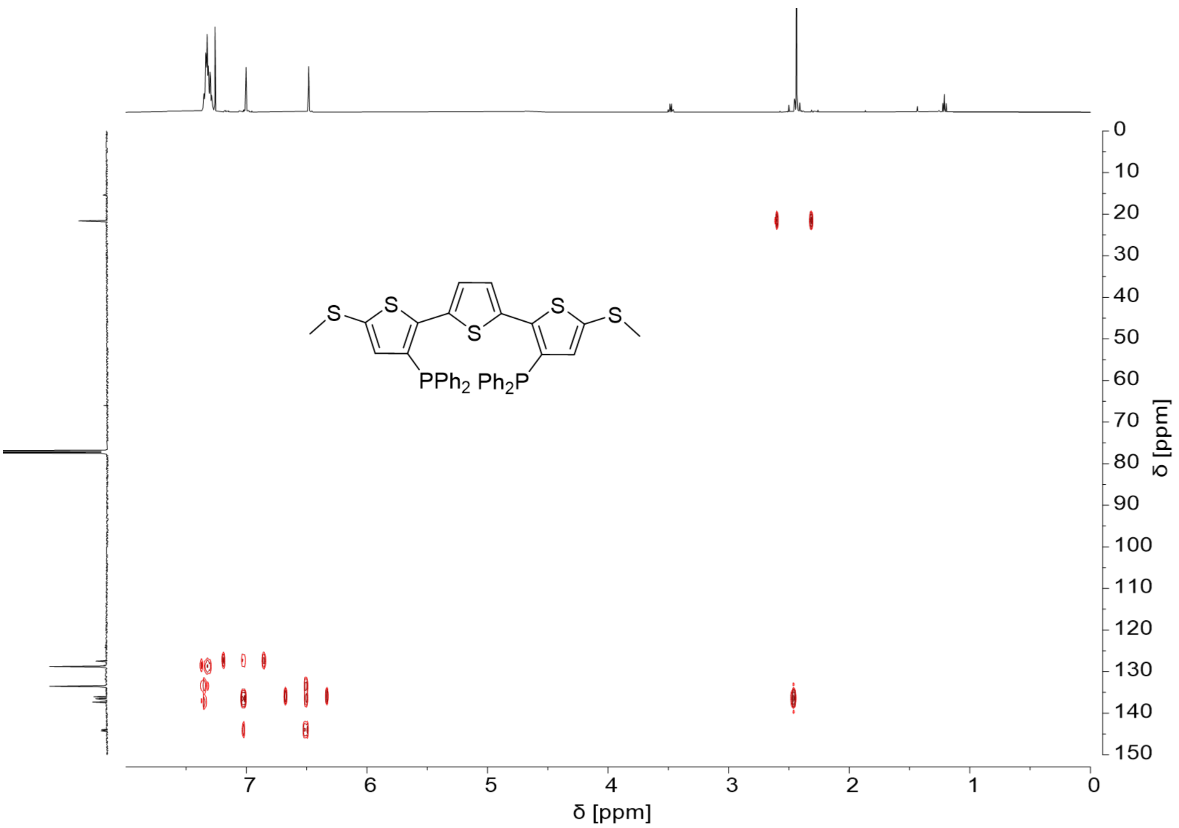

Figure S49. $^1H/^{13}C$ HMBC NMR (500/126 MHz, $CDCl_3$, 298 K) spectrum of **3**.

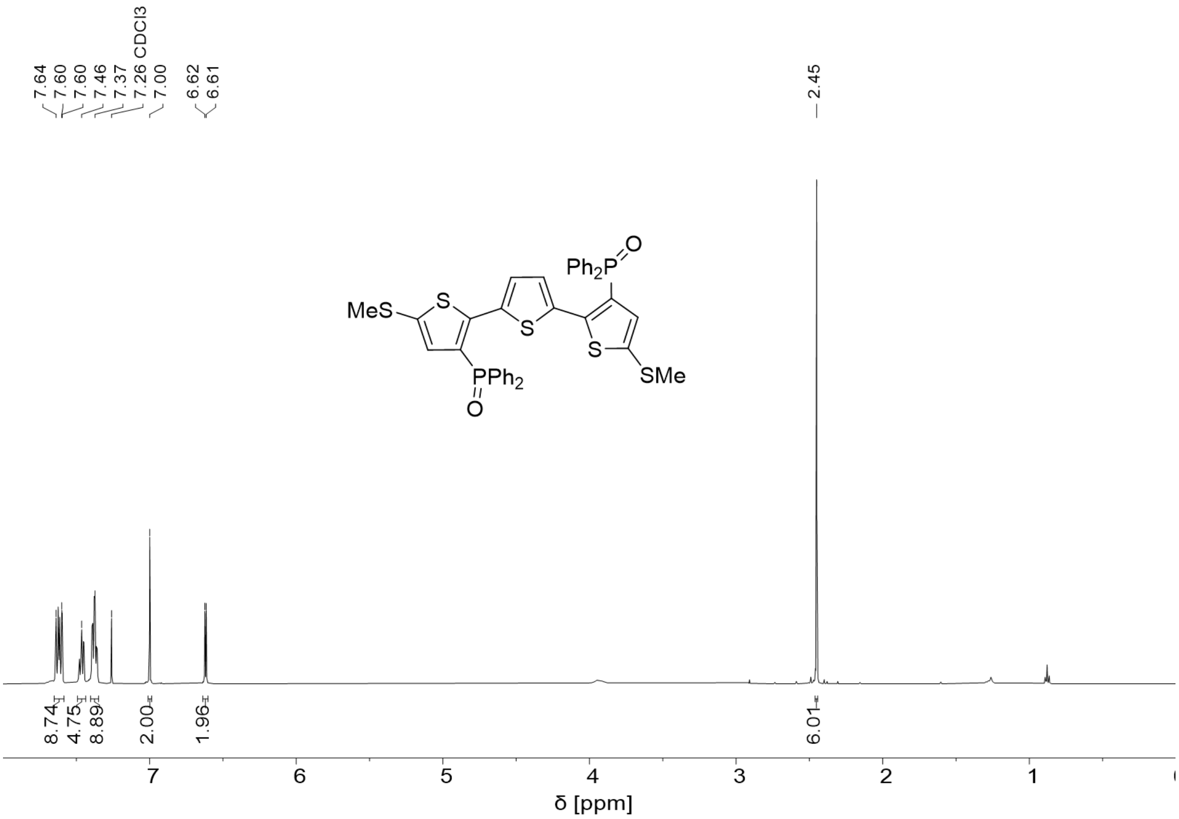

Figure S50. ^1H NMR (500 MHz, $CDCl_3$, 298 K) spectrum of **4**.

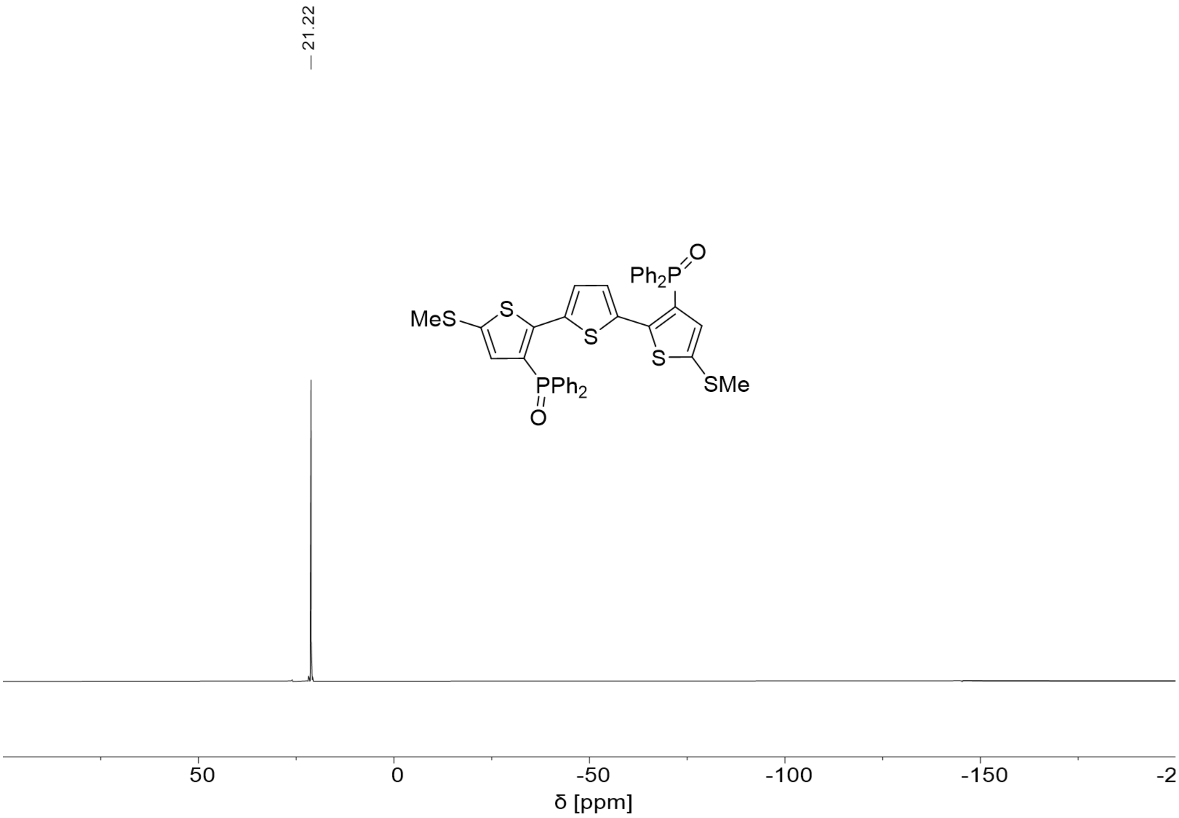

Figure S51. $^{31}P\{^{1}H\}$ NMR (202 MHz, $CDCl_3$, 298 K) spectrum of **4**.

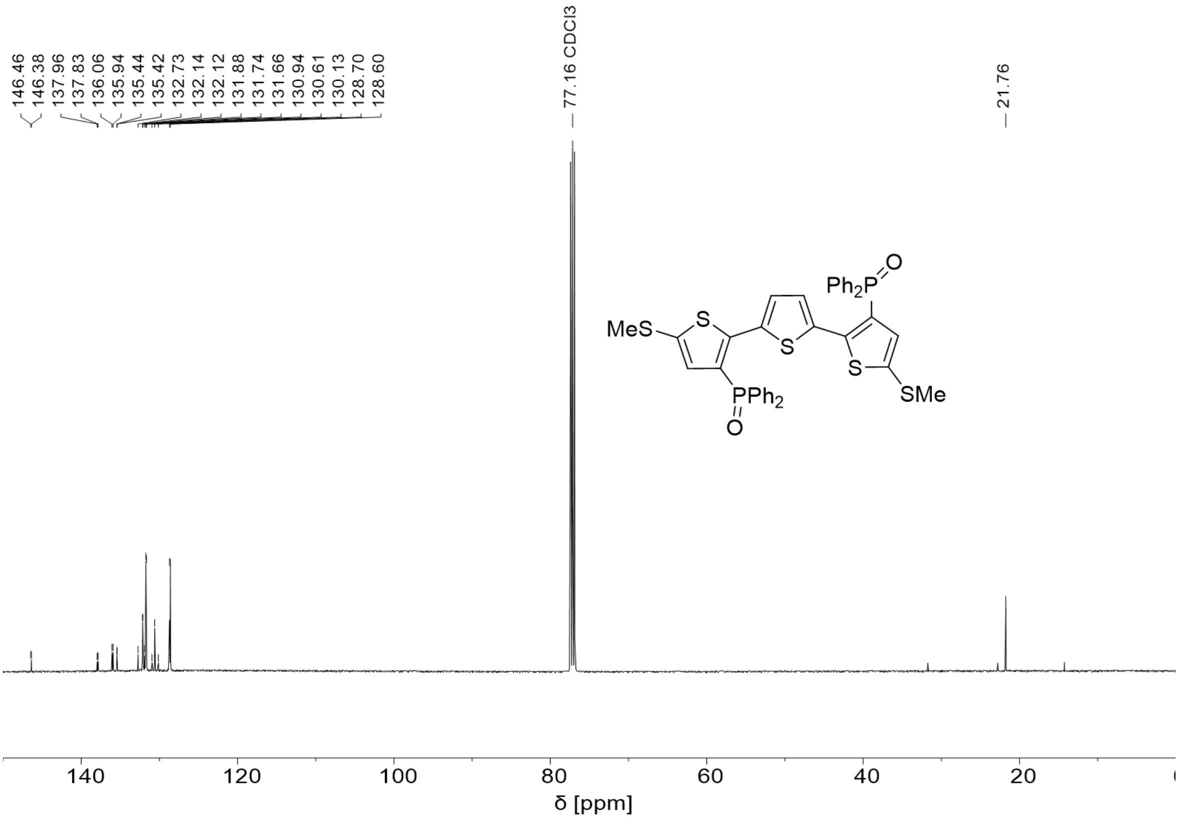

Figure S52. $^{13}C\{^{1}H\}$ NMR (126 MHz, $CDCl_3$, 298 K) spectrum of **4**.

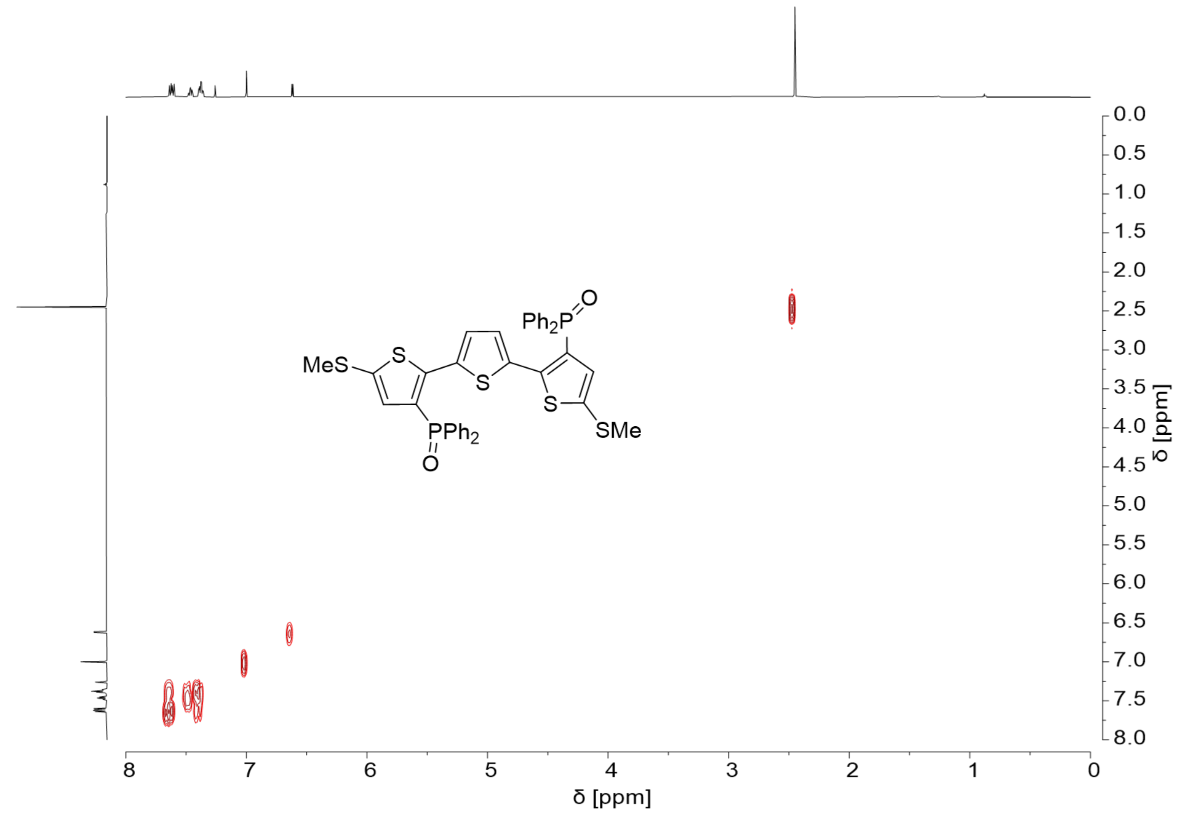

Figure S53. ^{1}H/^{1}H COSY NMR (500/500 MHz, $CDCl_3$, 298 K) spectrum of **4**.

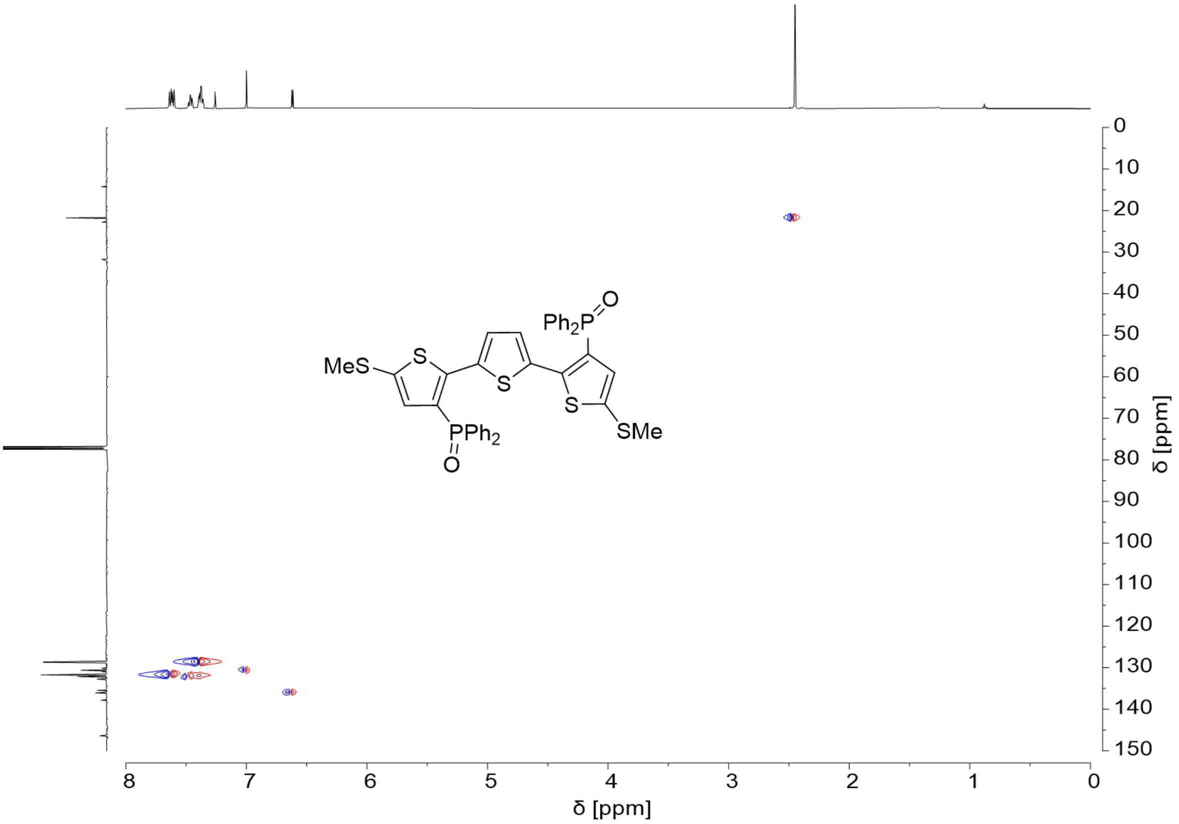

Figure S54. ^{1}H/^{13}C HSQC NMR (500/126 MHz, $CDCl_3$, 298 K) spectrum of **4**.

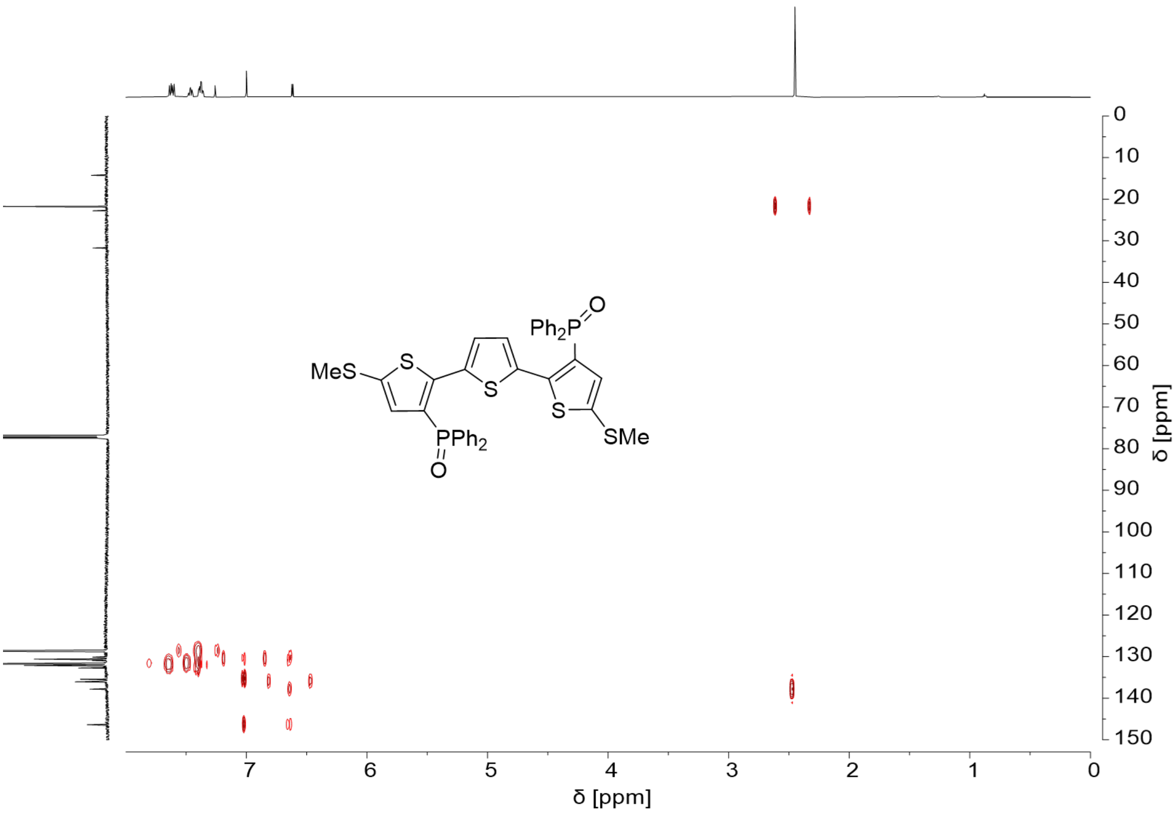

Figure S55. $^{1}H/^{13}C$ HMBC NMR (500/126 MHz, $CDCl_3$, 298 K) spectrum of **4**.

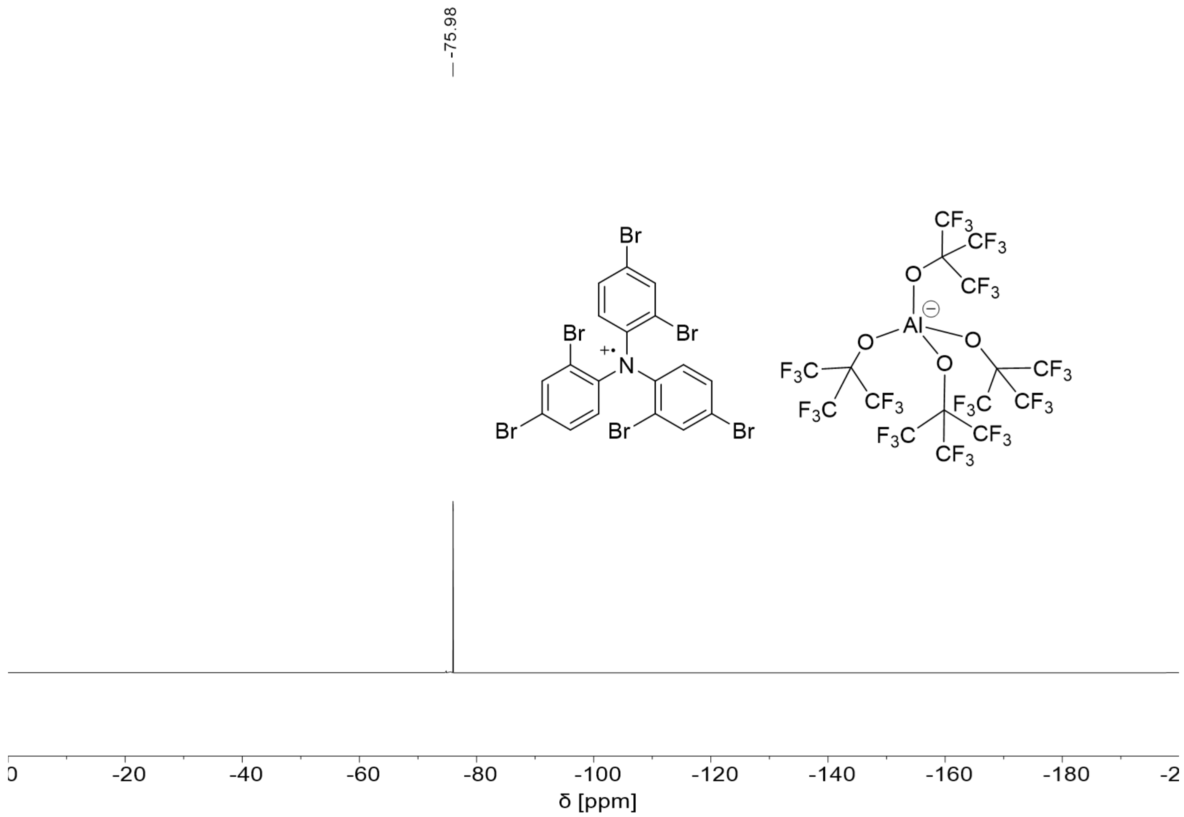

Figure S56. $^{19}F\{^{1}H\}$ NMR (471 MHz, CD_3CN, 298 K) spectrum of [**MG**][Al(OR^F)$_4$].

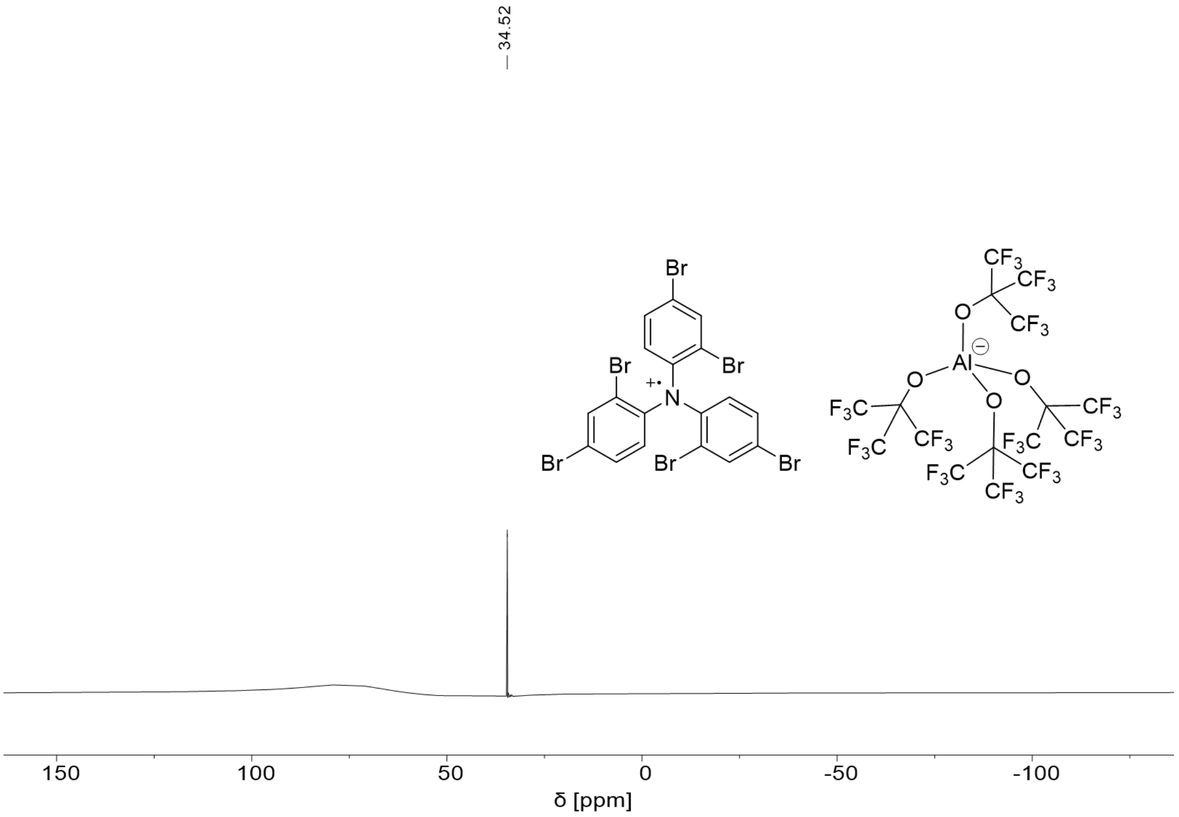

Figure S57. ^{27}Al NMR (130 MHz, CD_3CN, 298 K) spectrum of [**MG**][Al(OR^F)$_4$].

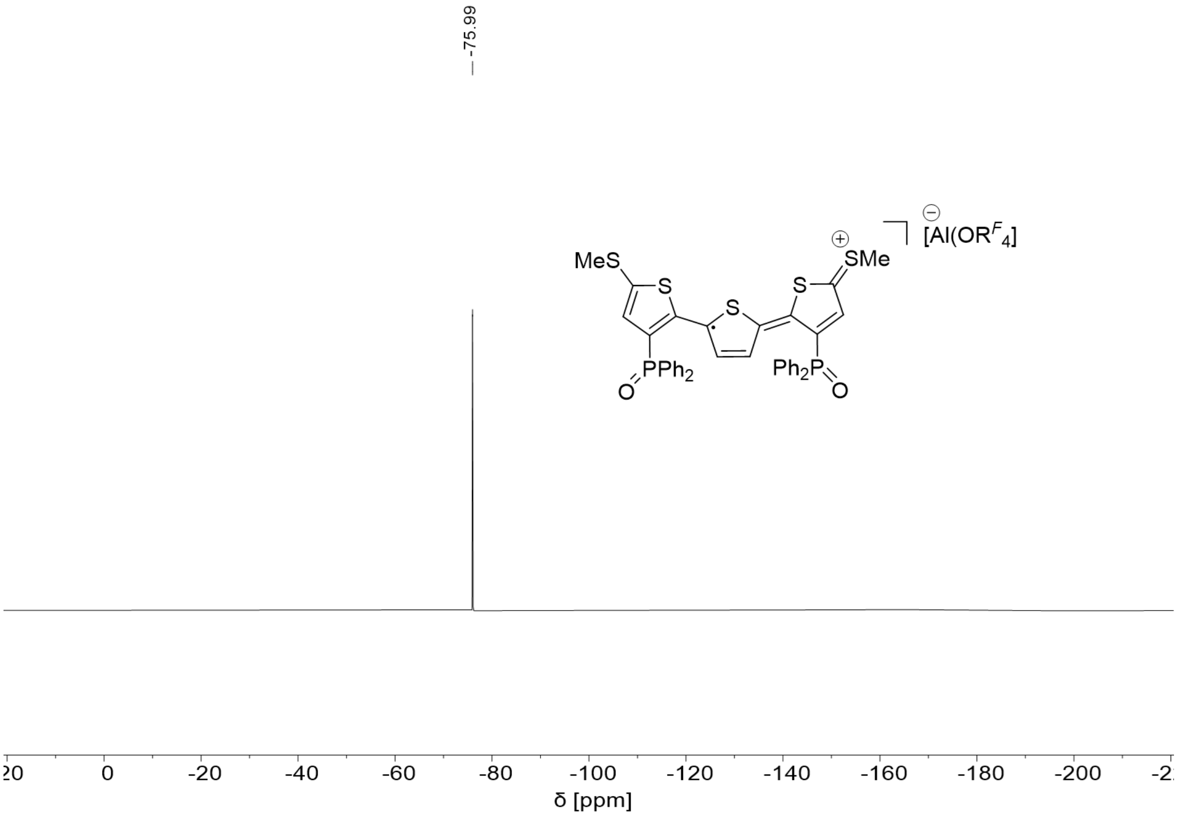

Figure S58. ^{19}F{^{1}H} NMR (471 MHz, CD_3CN, 298 K) spectrum of **4**$^+$.

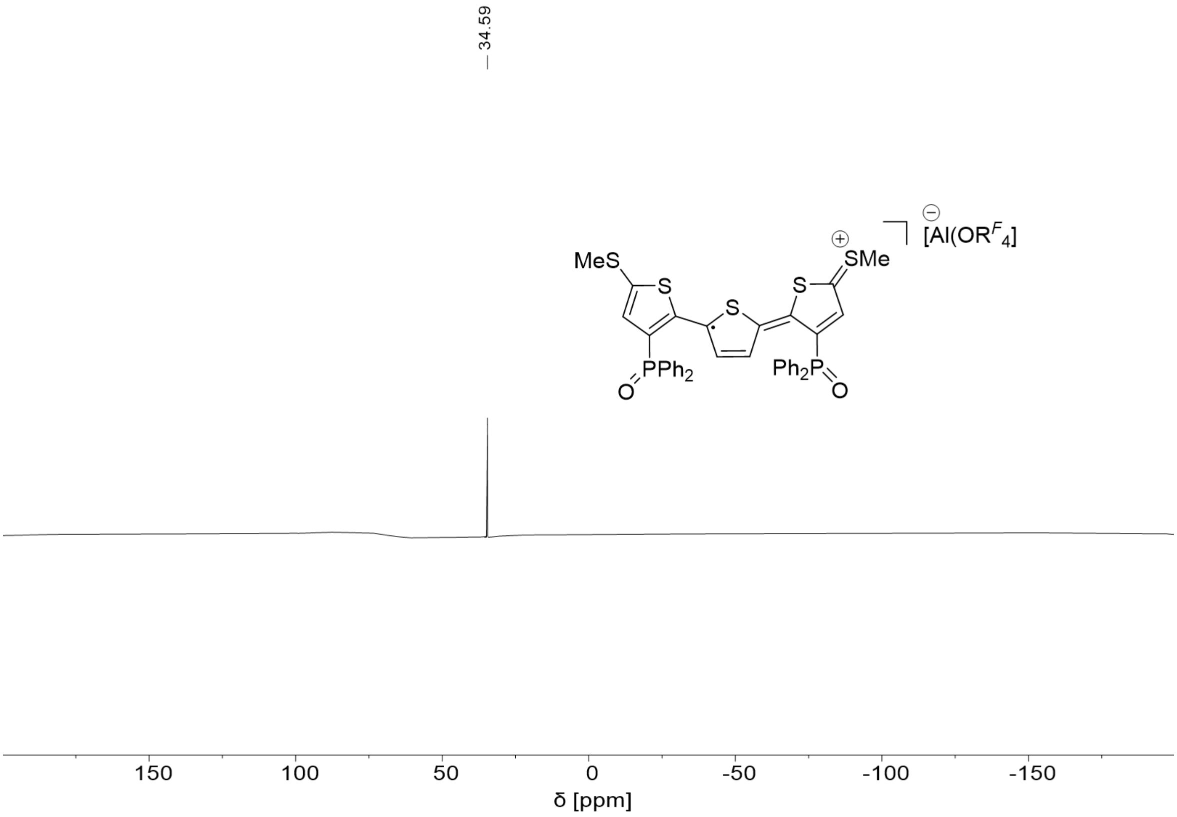

Figure S59. ^{27}Al NMR (130 MHz, CD_3CN, 298 K) spectrum of **4**$^{+}$.

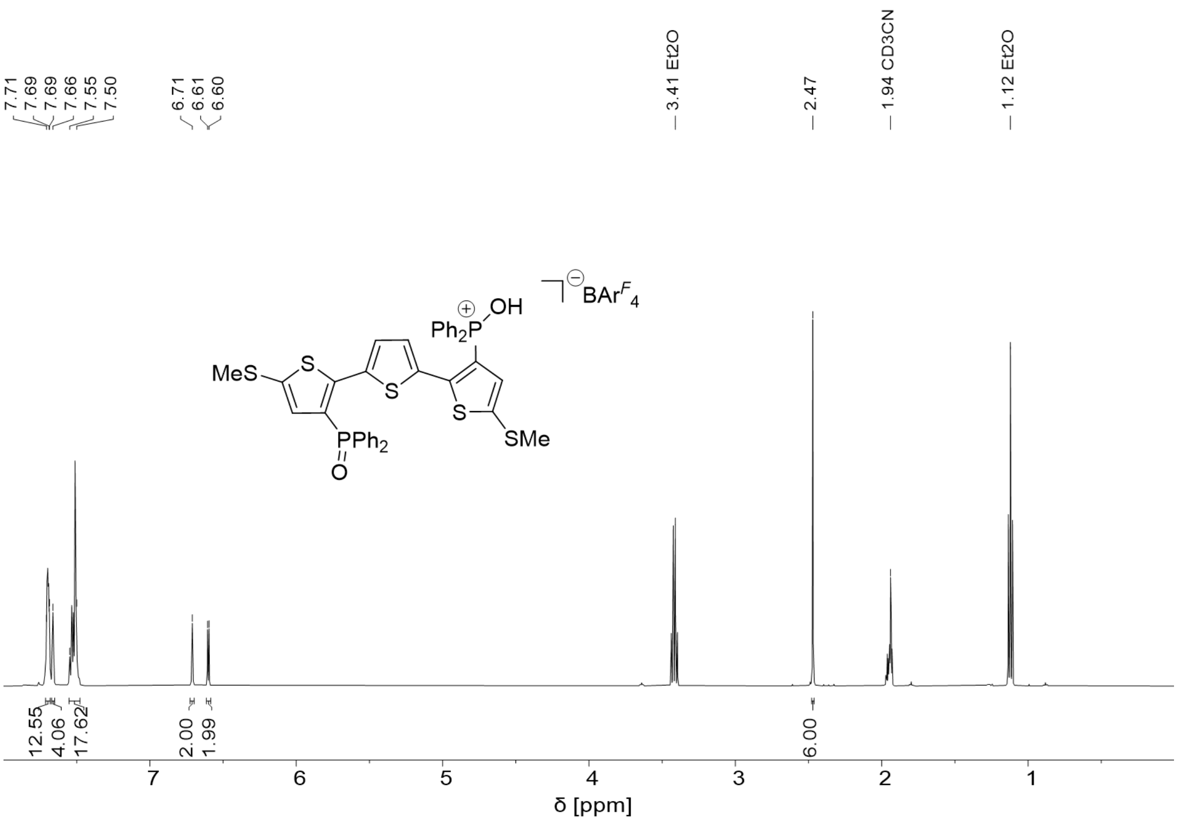

Figure S60. ^{1}H NMR (500 MHz, CD_3CN, 298 K) spectrum of **4–H**$^{+}$.

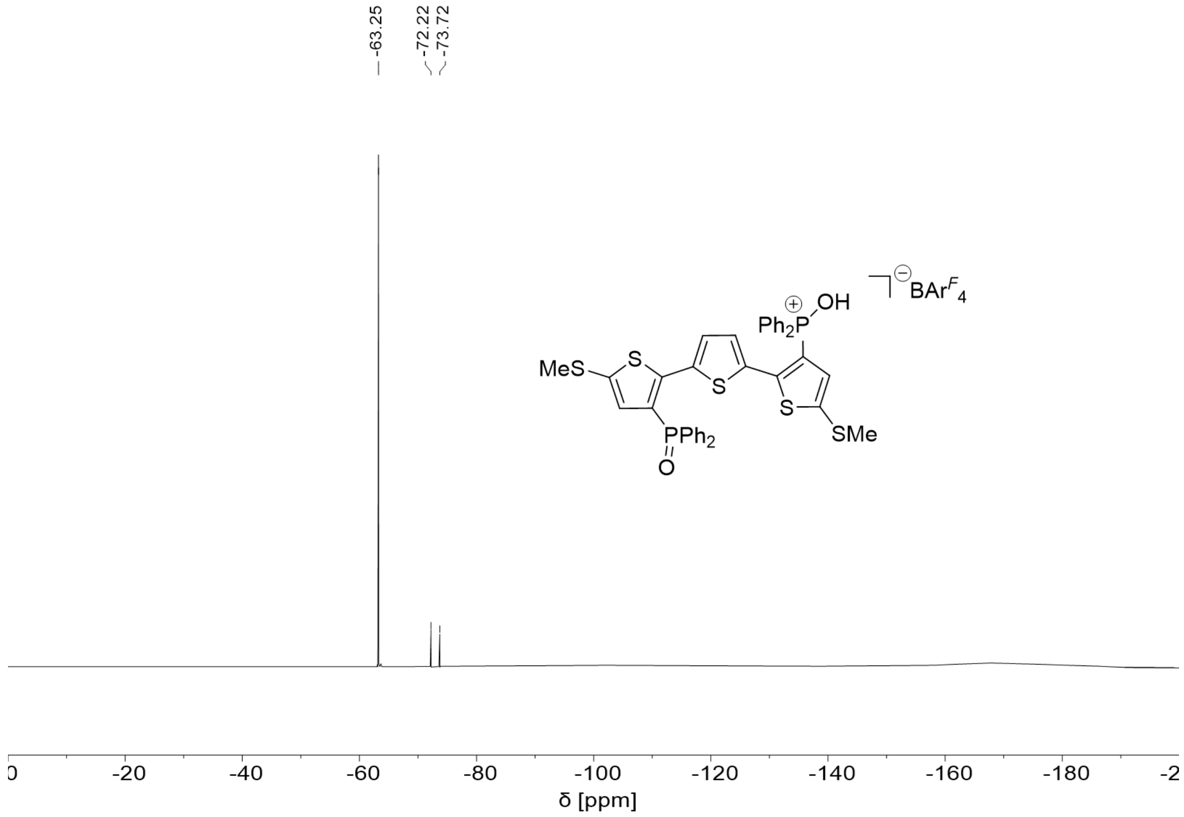

Figure S61. $^{19}F\{^{1}H\}$ NMR (471 MHz, CD_3CN, 298 K) spectrum of **4–H$^+$** with a Li[PF_6] capillary.

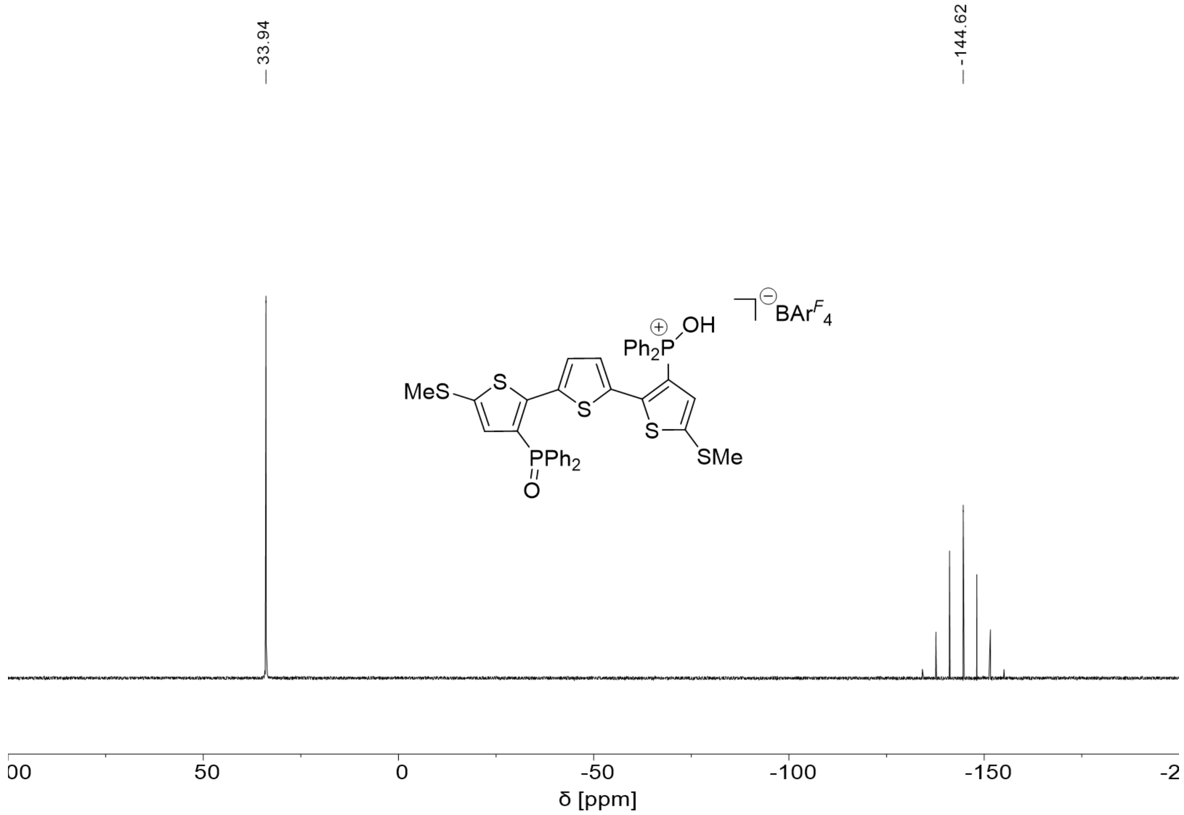

Figure S62. $^{31}P\{^{1}H\}$ NMR (202 MHz, CD_3CN, 298 K) spectrum of **4–H$^+$** with a Li[PF_6] capillary.

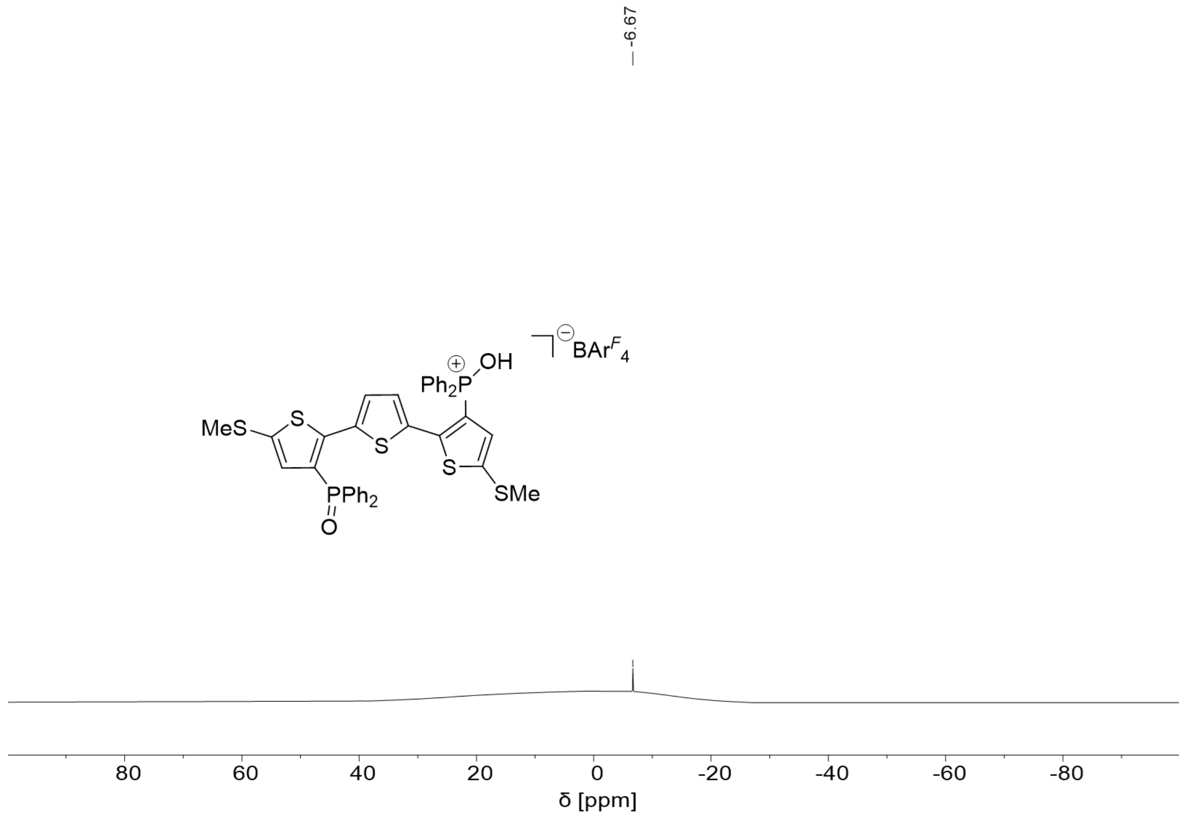

Figure S63. ^{11}B NMR (160 MHz, CD_3CN, 298 K) spectrum of **4–H+**.

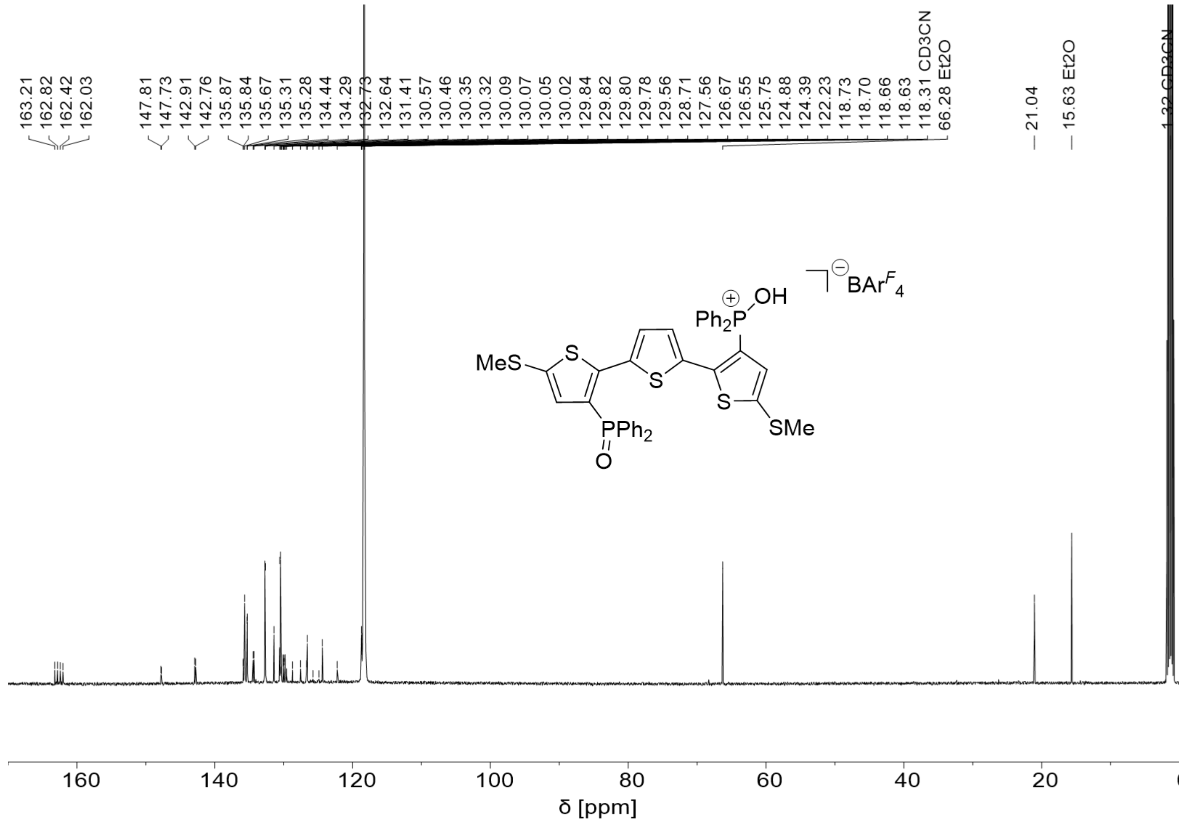

Figure S64. ^{13}C{^{1}H} NMR (126 MHz, CD_3CN, 298 K) spectrum of **4–H+**.

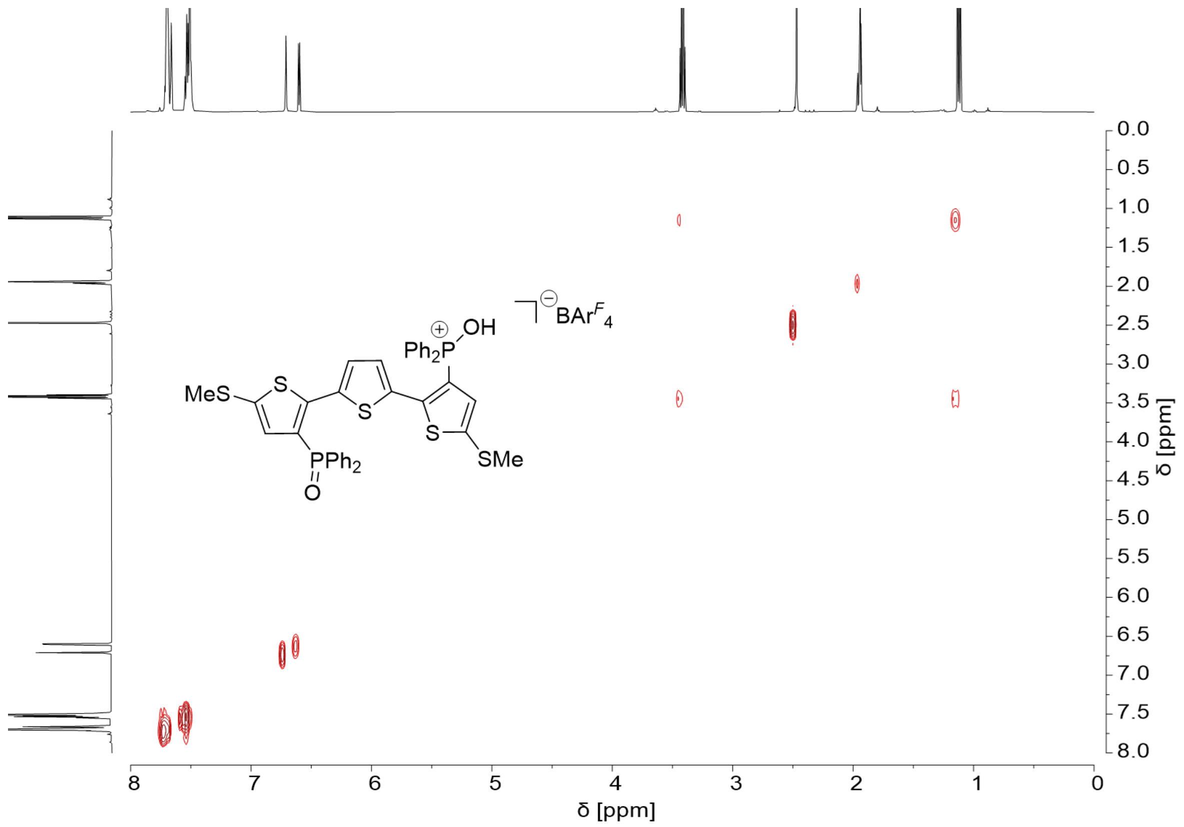

Figure S65. $^{1}H/^{1}H$ COSY NMR (500/500 MHz, CD_3CN, 298 K) spectrum of **4–H+**.

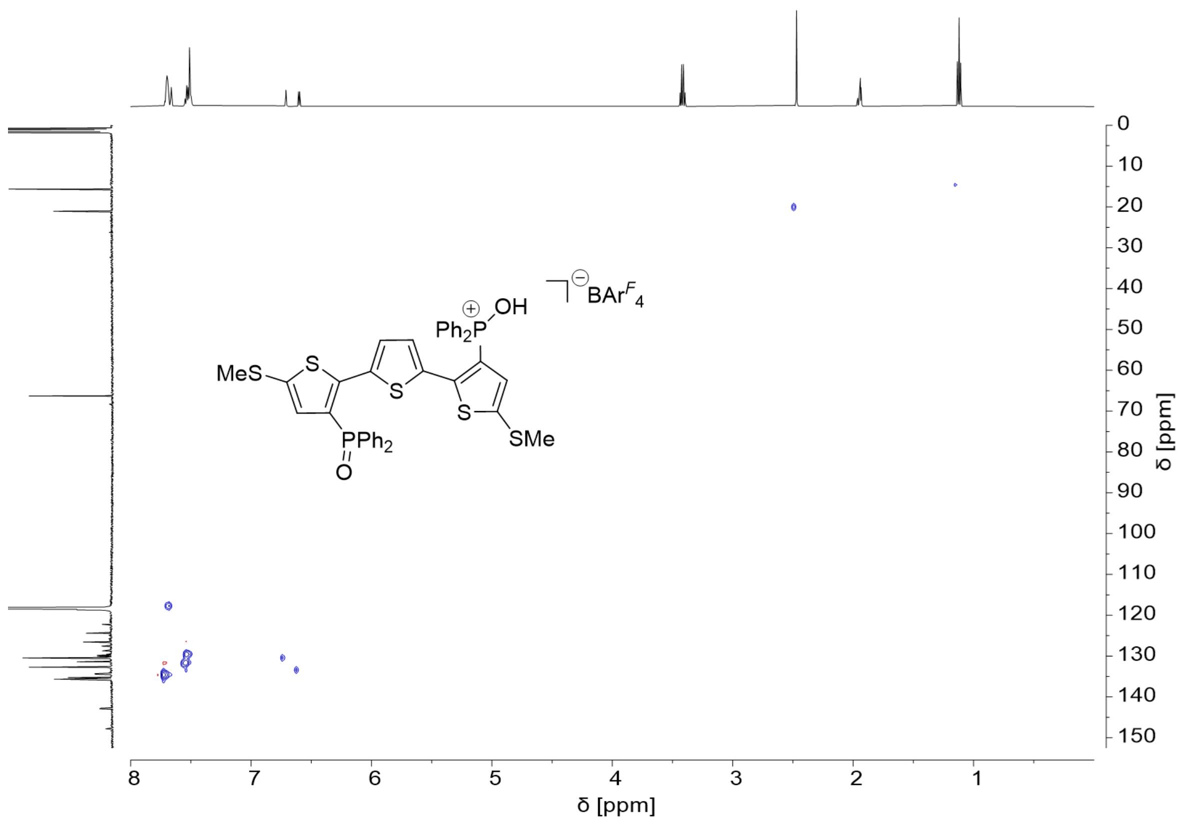

Figure S66. $^{1}H/^{13}C$ HSQC NMR (500/126 MHz, CD_3CN, 298 K) spectrum of **4–H+**.

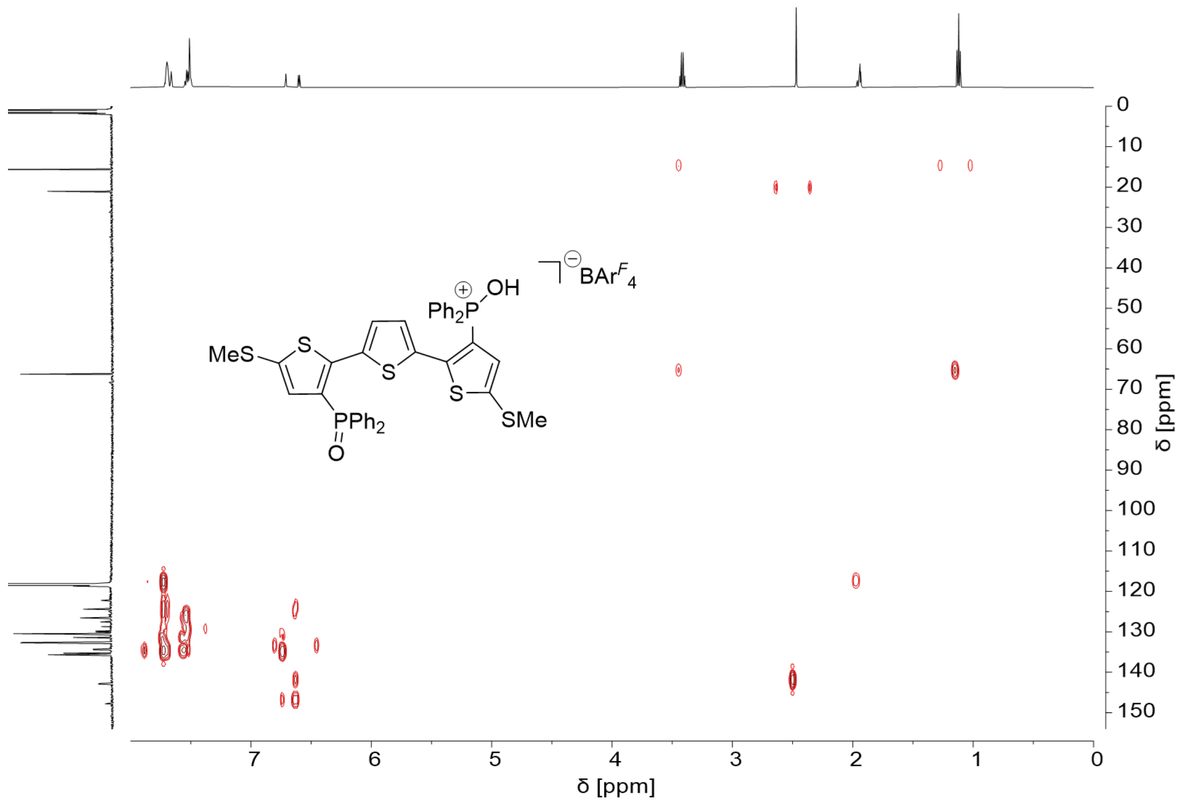

Figure S67. ^{1}H/^{13}C HMBC NMR (500/126 MHz, CD_3CN, 298 K) spectrum of **4–H+**.

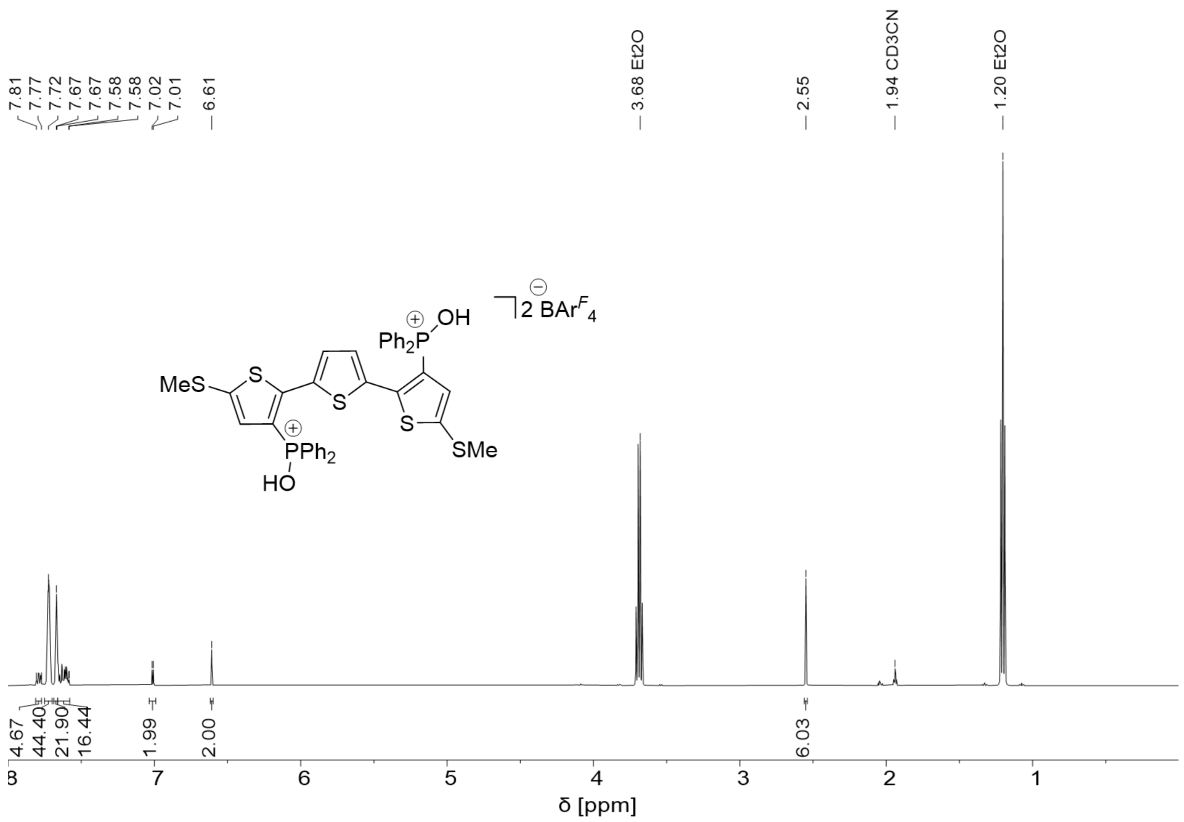

Figure S68. ^{1}H NMR (500 MHz, CD_3CN, 298 K) spectrum of **4–H$_2$$^{2+}$**.

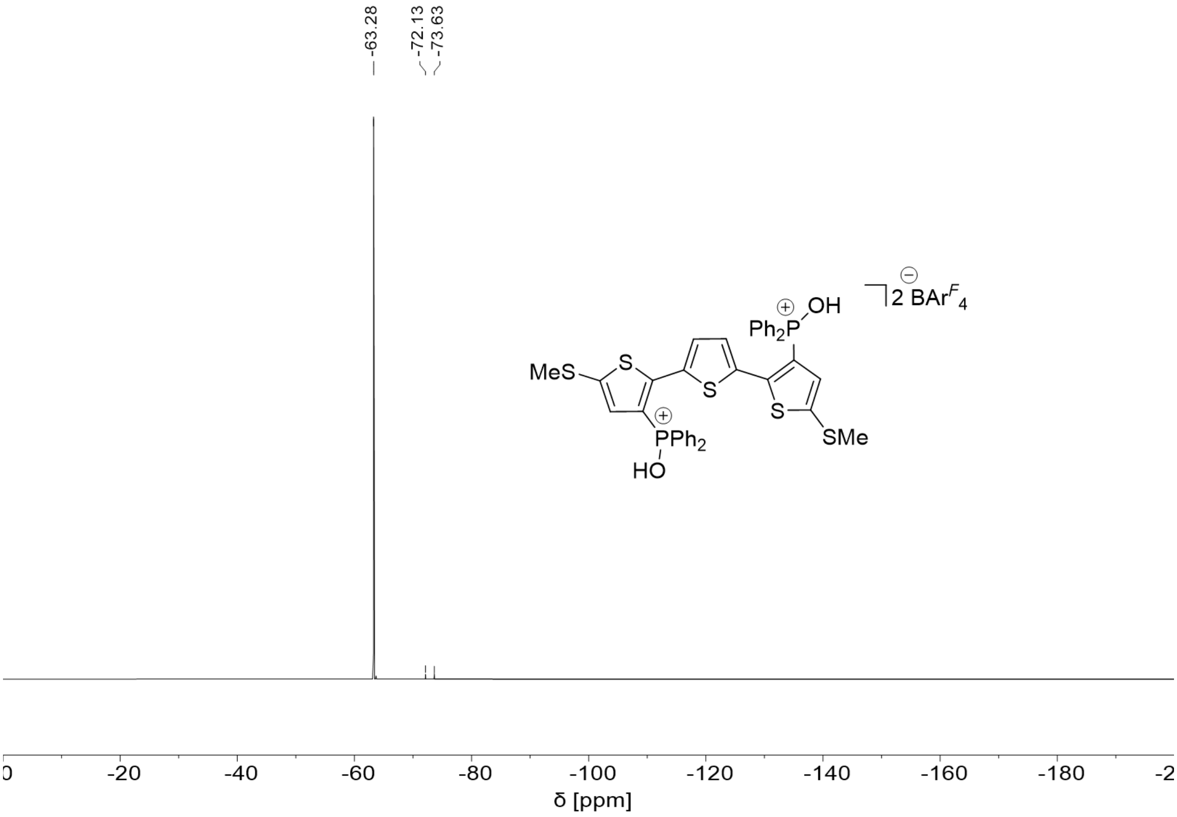

Figure S69. $^{19}F\{^1H\}$ NMR (471 MHz, CD_3CN, 298 K) spectrum of **4–H$_2^{2+}$** with a Li[PF_6] capillary.

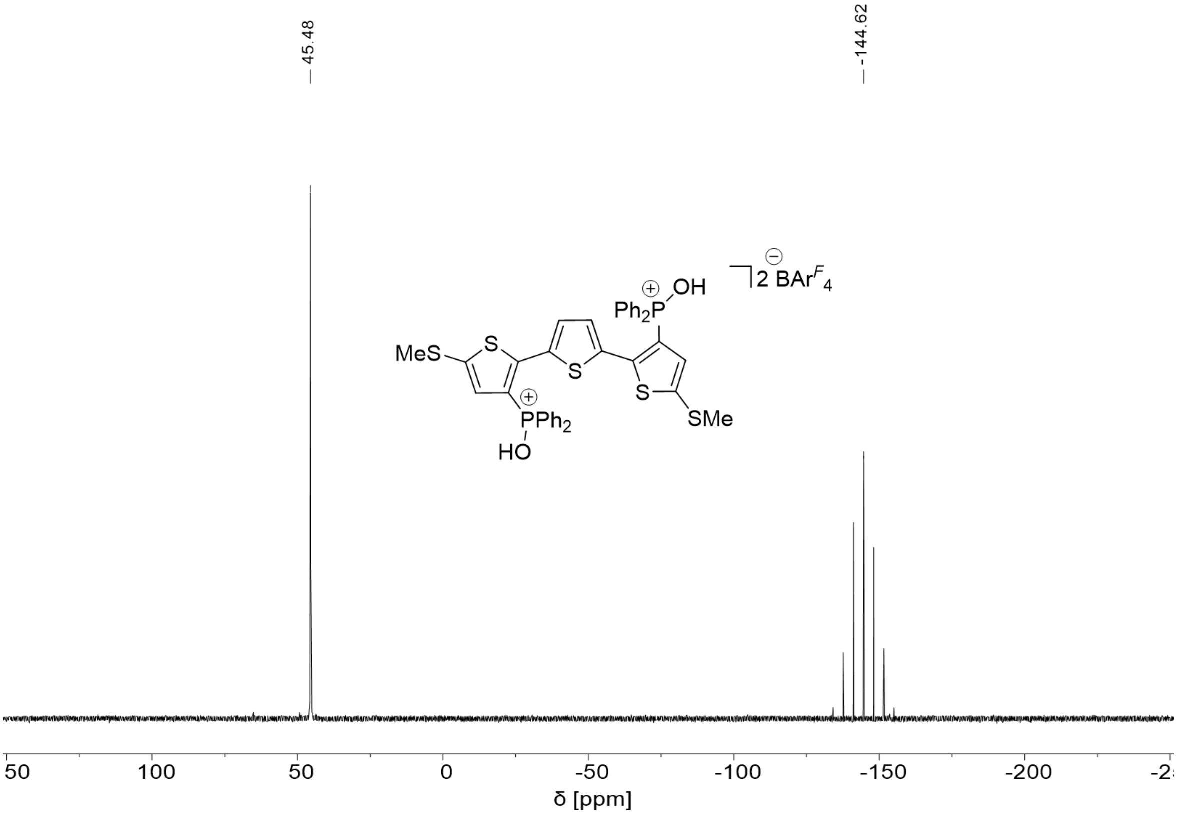

Figure S70. $^{31}P\{^1H\}$ NMR (202 MHz, CD_3CN, 298 K) spectrum of **4–H$_2^{2+}$** with a Li[PF_6] capillary.

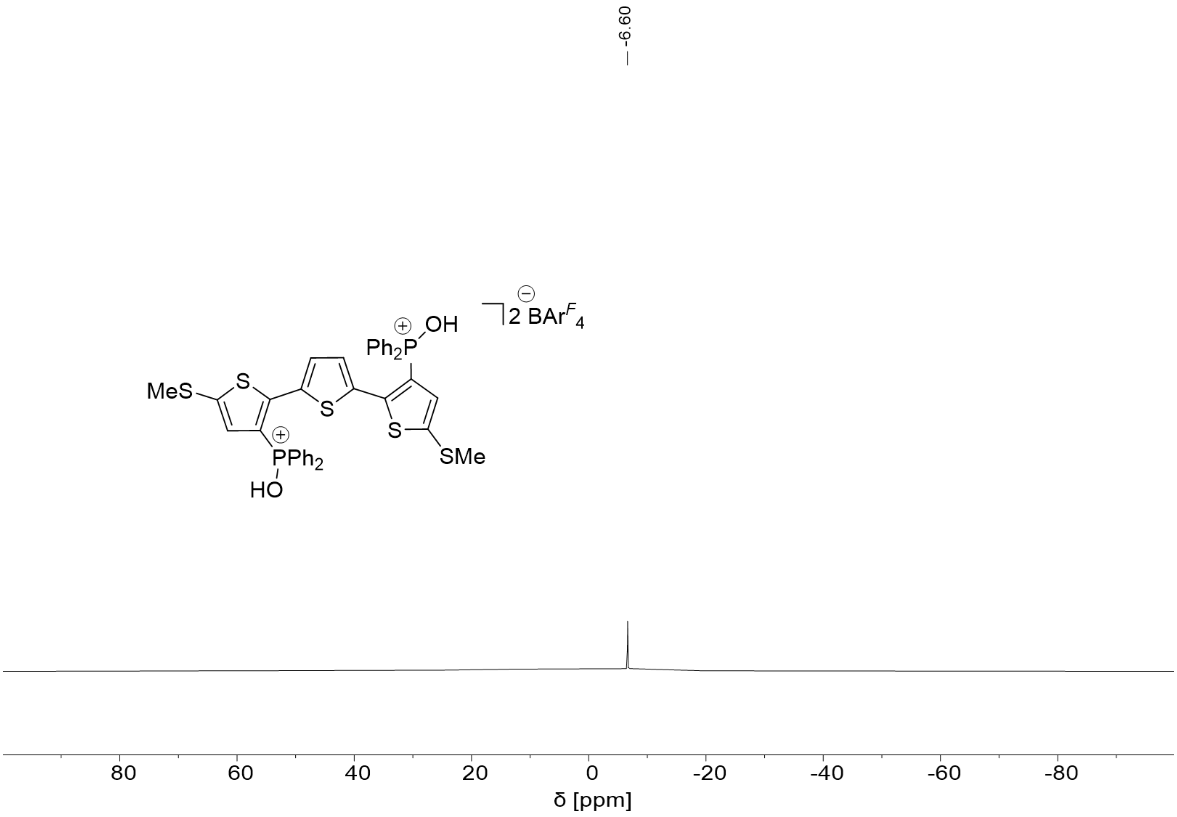

Figure S71. ^{11}B NMR (160 MHz, CD_3CN, 298 K) spectrum of **4–H$_2$$^{2+}$**.

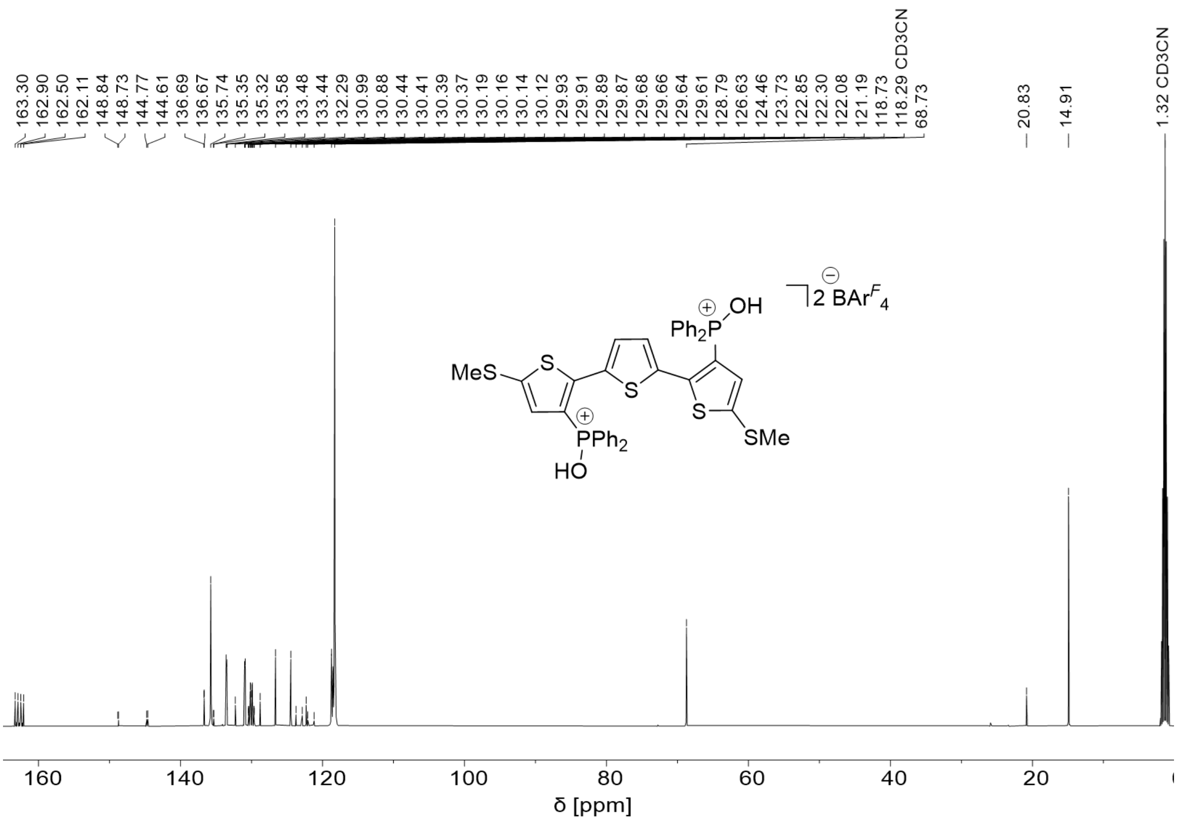

Figure S72. ^{13}C{^{1}H} NMR (126 MHz, CD_3CN, 298 K) spectrum of **4–H$_2$$^{2+}$**.

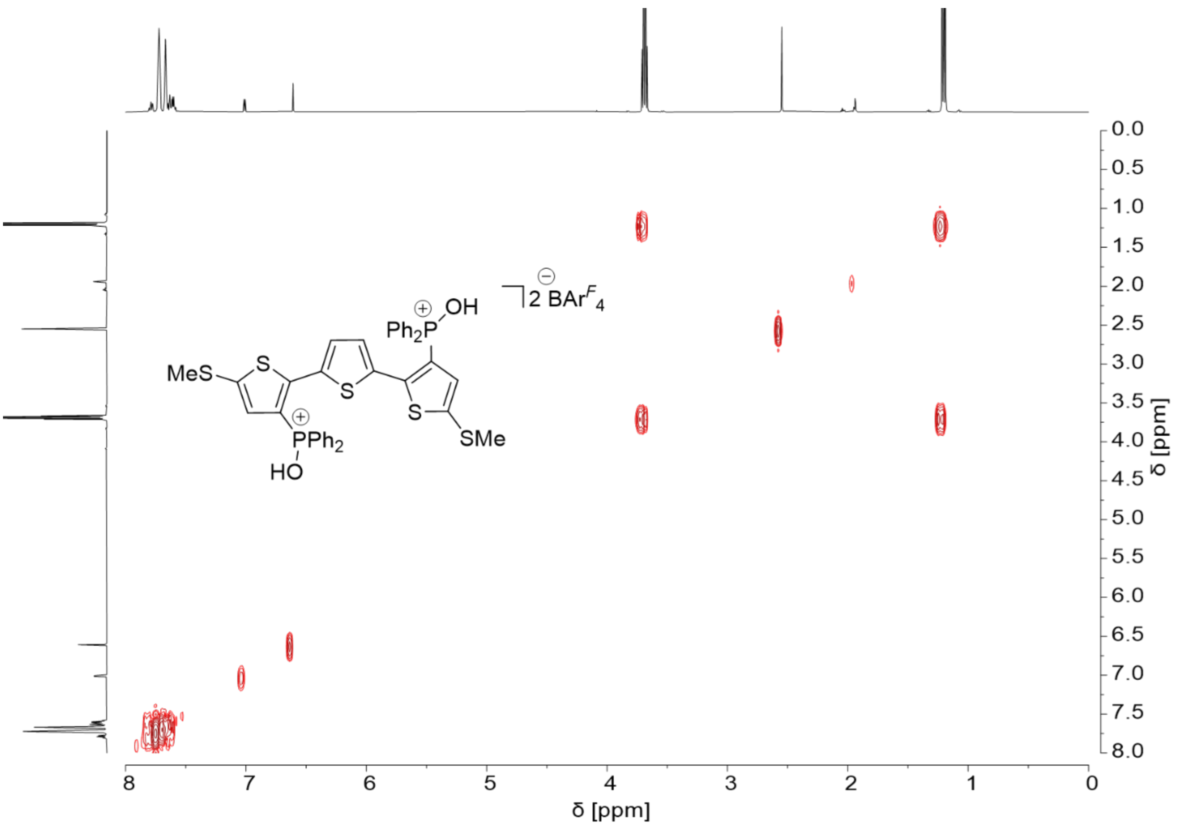

Figure S73. $^{1}H/^{1}H$ COSY NMR (500/500 MHz, CD_3CN, 298 K) spectrum of **4–H$_2^{2+}$**.

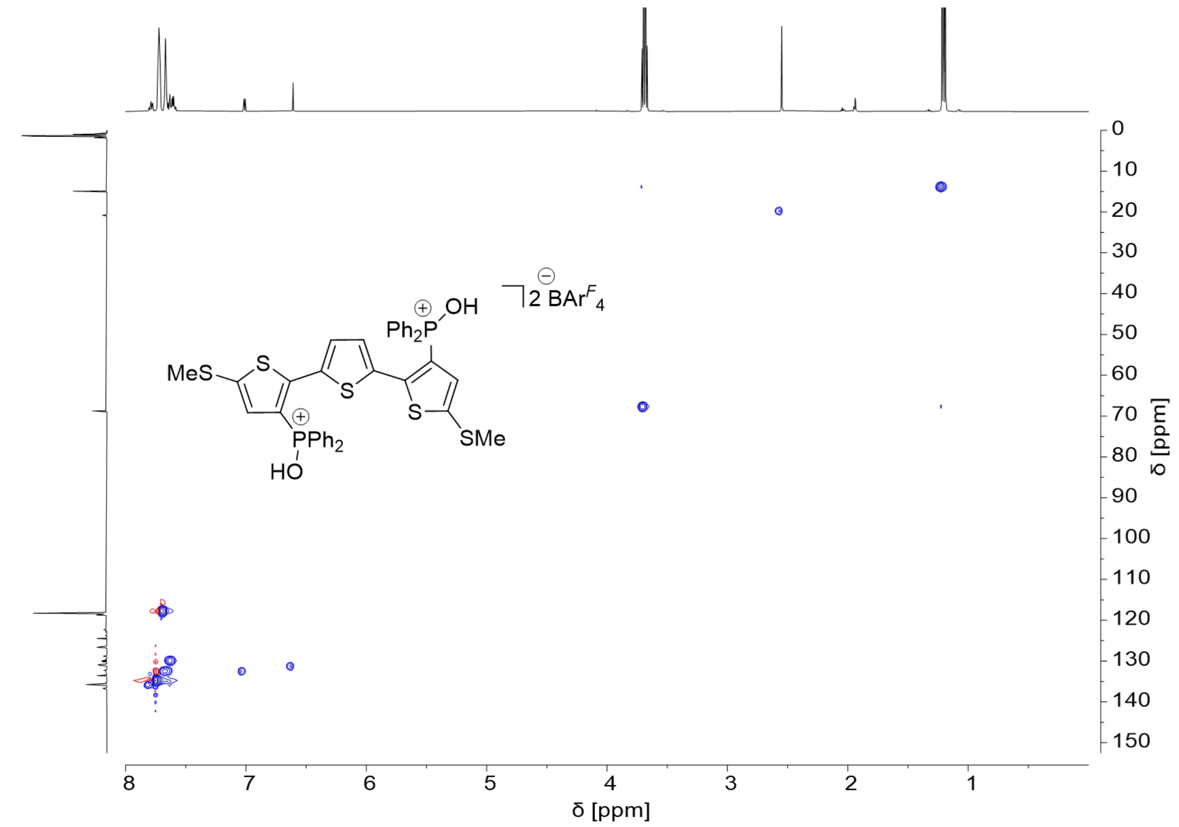

Figure S74. $^{1}H/^{13}C$ HSQC NMR (500/126 MHz, CD_3CN, 298 K) spectrum of **4–H$_2^{2+}$**.

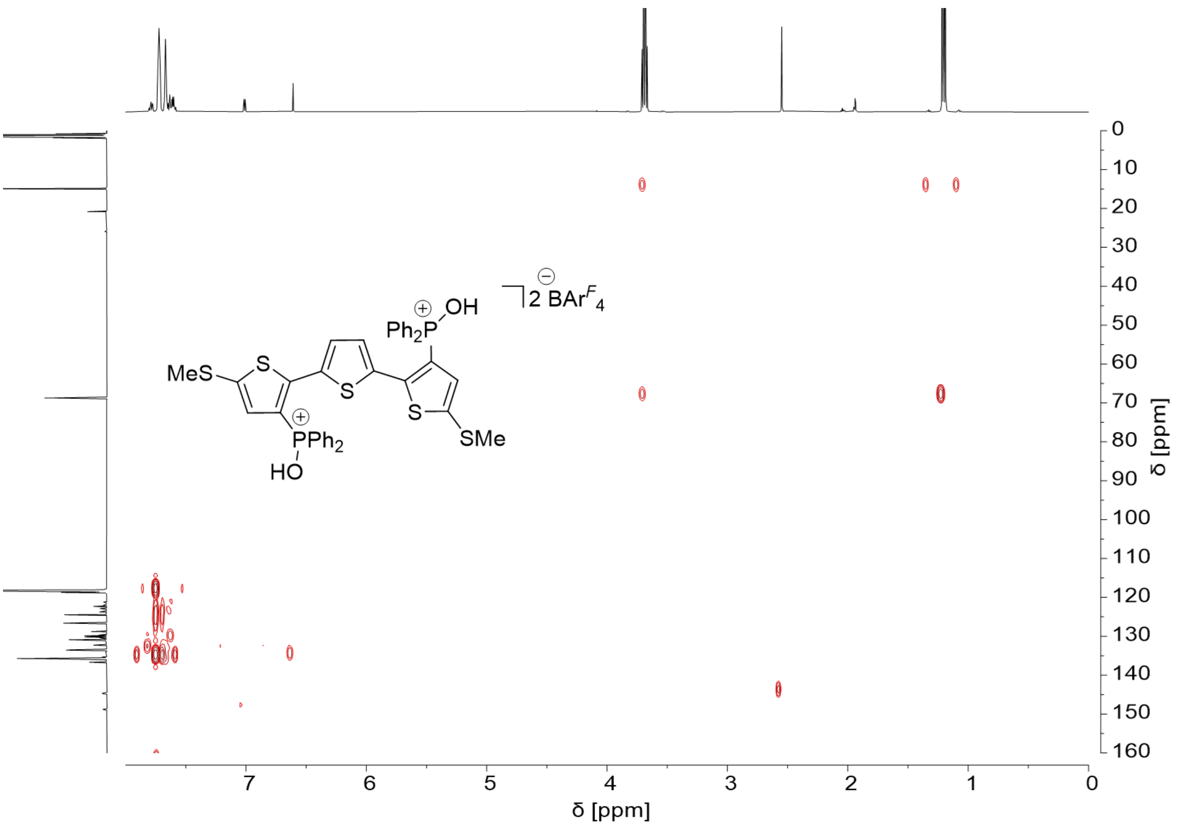

Figure S75. $^{1}H/^{13}C$ HMBC NMR (500/126 MHz, CD_3CN, 298 K) spectrum of **4–H$_2^{2+}$**.

XI. EPR Data

Compound **[K(THF)$_2$(4)]$_2$**

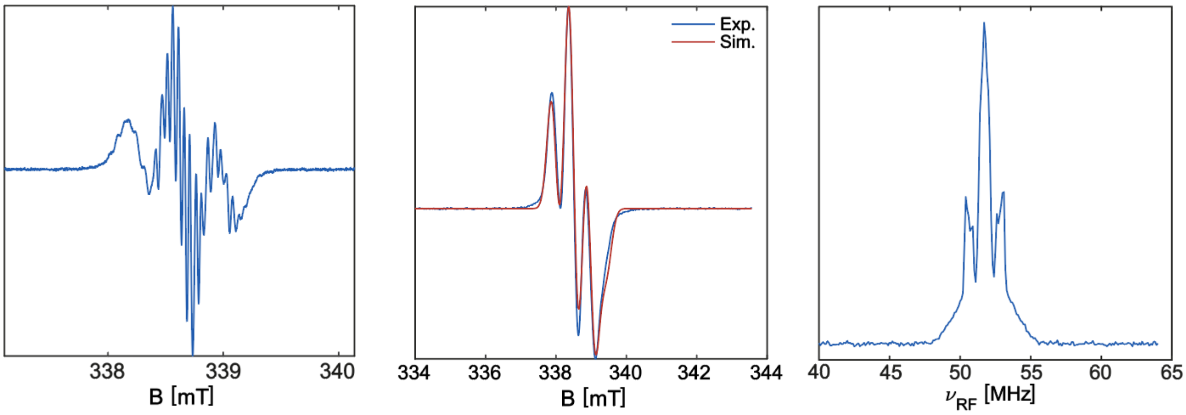

Figure S76. Left: X-band CW EPR spectrum at 298 K. Middle: X-band CW EPR spectrum at 90 K. Right: Q-band ^{1}H ENDOR at 40 K.

The X-band EPR spectrum at 298 K shows slight anisotropy that can be explained by the increased bond order of the *inter*-thiophene bonds, accompanied with the coordination of the phosphine oxide to potassium. While ^{1}H hyperfine splitting can be resolved at 298 K simulation was not feasible due to the observed averaging behaviour that determines the spectral shape, the molecular tumbling/viscosity at 298 K and the 10 ^{1}H atoms of the terthiophene backbone and 1 *ortho*-^{1}H of each phenyl unit with (slightly) distributed hyperfine couplings, yielding the broader ^{1}H ENDOR spectrum. Qualitatively, this is in agreement with the observed spin densities EPR parameter DFT calculations (see chapter XIII: Computational Data). Simulations of the X- and Q-band EPR data result in the anisotropic tensors of g = 2.0017 2.0056 2.0056 and A(^{31}P) = (10.10 13.83 13.83) MHz.

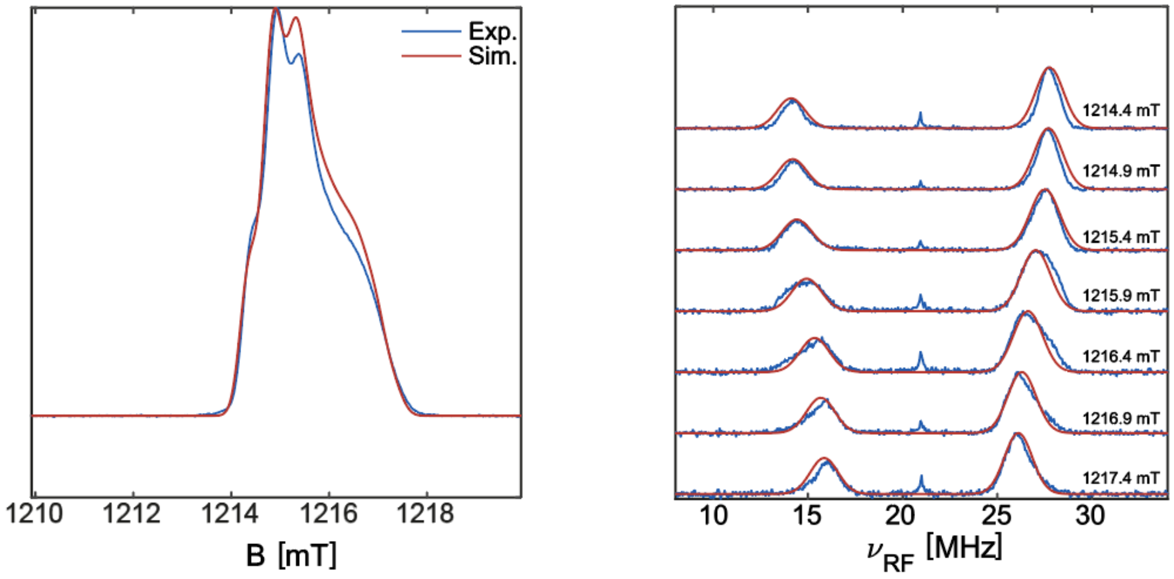

Figure S77. Left: Q-band pulse EPR spectrum at 40 K. Right: Magnetic field dependent Q-band ^{31}P ENDOR at 40 K.

Compound **4**⁻

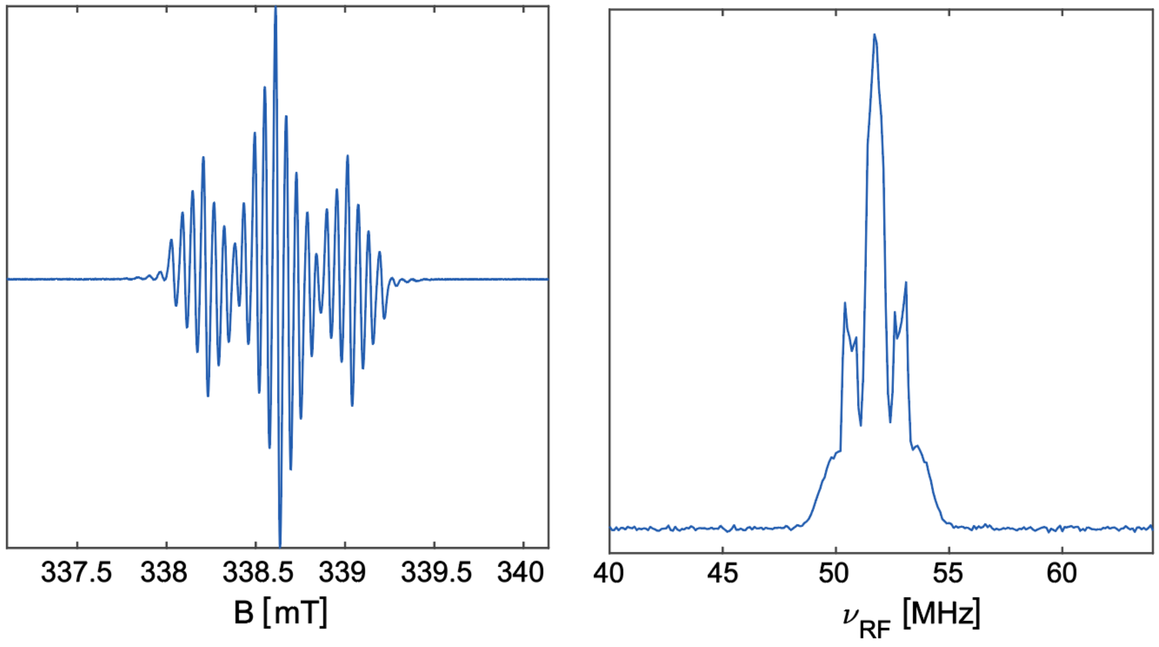

Figure S78. Left: X-band CW EPR spectrum at 298 K. Right: Q-band ^{1}H ENDOR at 40 K.

While ^{1}H hyperfine splitting can be resolved at 298 K, simulation was not attempted due to the molecular tumbling/viscosity at 298 K and the 10 ^{1}H atoms of the terthiophene backbone and 1 *ortho*-^{1}H of each phenyl unit with (slightly) distributed hyperfine couplings, yielding the broader features in the ^{1}H ENDOR spectrum. Qualitatively, this is in agreement with the observed spin densities EPR parameter DFT calculations (see chapter XIII: Computational Data).

Compound [**MG**][Al(OR^F)$_4$]

Simulations of the X- and Q-band EPR data result in the anisotropic g-tensors of g = 2.0009 2.0145 2.0190 and an estimated ^{14}N hyperfine interaction of ca. 20 MHz from the Q-band HYSCORE spectrum.

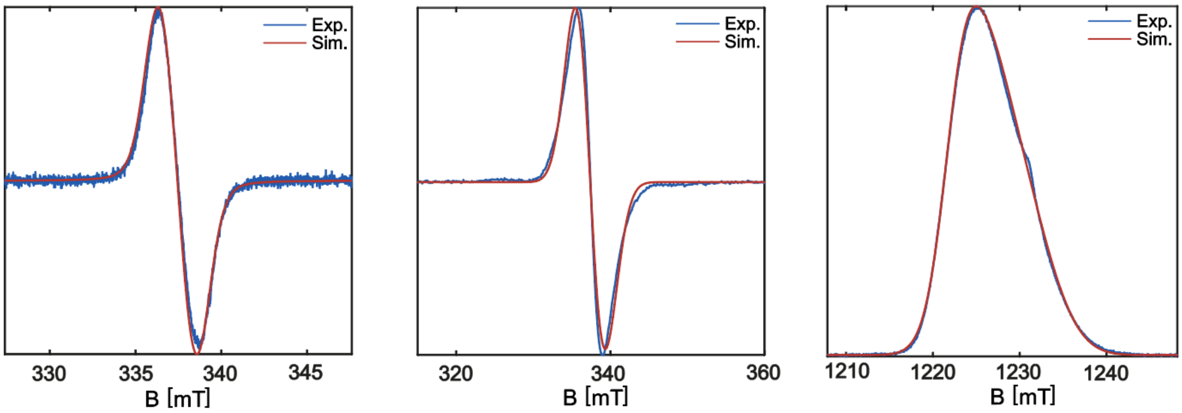

Figure S79. Left: X-band CW EPR spectrum at 298 K. Middle: X-band CW EPR spectrum at 90 K. Right: Q-band pulse EPR spectrum at 90 K.

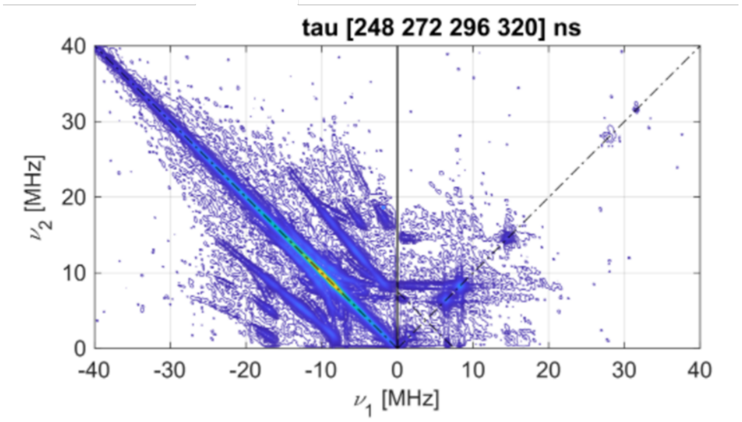

Figure S80. Q-band HYSCORE at 50 K showing strongly coupled anisotropic and distributed ^{14}N.

Compound **4⁺**

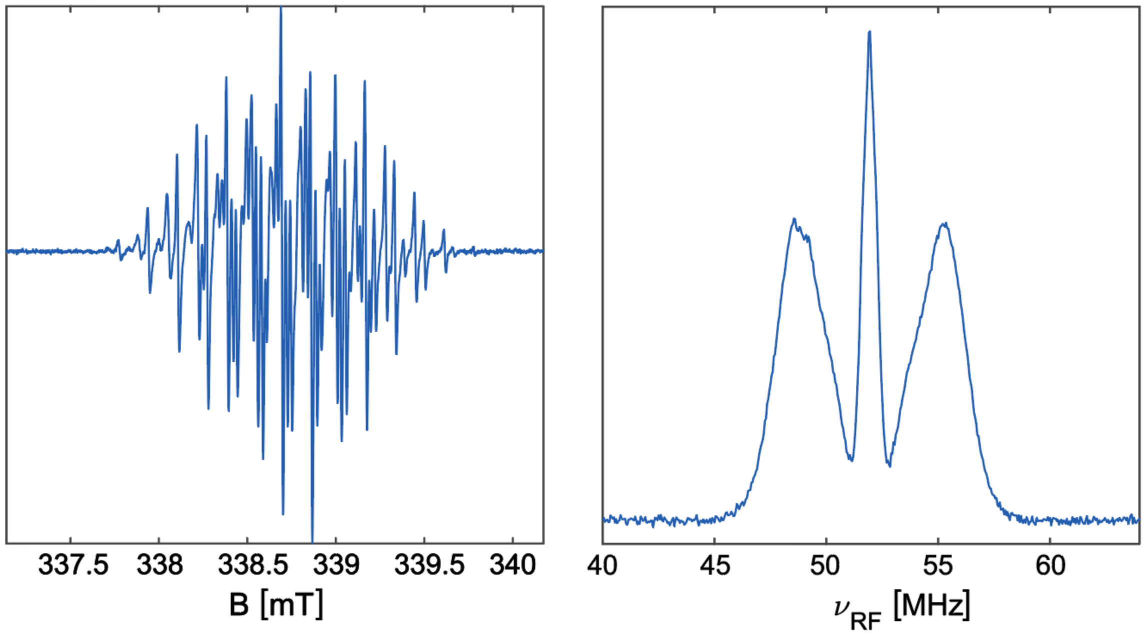

Figure S81. Left: X-band CW EPR spectrum at 298 K. Right: ^{1}H ENDOR at 40 K.

While ^{1}H hyperfine splitting can be resolved at 298 K simulation was not feasible due to the combination of the molecular tumbling/viscosity at 298 K and the 10 ^{1}H atoms of the terthiophene backbone and 1 *ortho*-^{1}H of each phenyl unit with (slightly) distributed hyperfine couplings, yielding the broader ^{1}H ENDOR spectrum. Qualitatively, this is in agreement with the observed spin densities EPR parameter DFT calculations (see chapter XIII: Computational Data).

XII. X-Ray Data

Crystal data and structure refinement of 4

Identification code	2394633
Empirical formula	$C_{38}H_{30}O_2P_2S_5$
Formula weight	740.86
Temperature/K	100.00(10)
Crystal system	triclinic
Space group	P-1
a/Å	9.61400(10)
b/Å	10.14950(10)
c/Å	19.2581(2)
α/°	82.5870(10)
β/°	86.6160(10)
γ/°	65.9560(10)
Volume/Å^3	1701.72(3)
Z	2
ρ_{calc}g/cm^3	1.446
μ/mm^{-1}	4.308
F(000)	768.0
Crystal size/mm^3	0.435 × 0.244 × 0.098
Radiation	Cu Kα (λ = 1.54184)
2Θ range for data collection/°	4.628 to 160.156
Index ranges	$-12 \leq h \leq 12$, $-12 \leq k \leq 12$, $-24 \leq l \leq 23$
Reflections collected	61827
Independent reflections	7312 [R_{int} = 0.0551, R_{sigma} = 0.0253]
Data/restraints/parameters	7312/0/426
Goodness-of-fit on F^2	1.060
Final R indexes [I>=2σ (I)]	R_1 = 0.0330, wR_2 = 0.0881
Final R indexes [all data]	R_1 = 0.0341, wR_2 = 0.0890
Largest diff. peak/hole / e Å^{-3}	0.62/-0.50

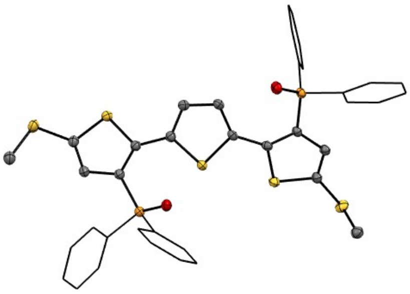

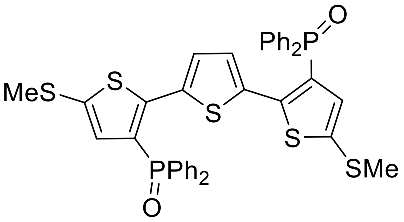

Crystal data and structure refinement of $[K(THF)_2(4)]_2$

Identification code	2394632
Empirical formula	$C_{100}H_{108}K_2O_{10}P_4S_{10}$
Formula weight	1992.54
Temperature/K	99.99(10)
Crystal system	triclinic
Space group	P-1
a/Å	13.2625(2)
b/Å	15.0264(2)
c/Å	16.6619(2)
α/°	113.8460(10)
β/°	97.7510(10)
γ/°	101.3420(10)
Volume/Å^3	2891.41(7)
Z	1
ρ_{calc}g/cm^3	1.144
μ/mm^{-1}	3.324
F(000)	1046.0
Crystal size/mm^3	0.183 × 0.101 × 0.091
Radiation	Cu Kα (λ = 1.54184)
2Θ range for data collection/°	5.972 to 160.38
Index ranges	$-16 \leq h \leq 16$, $-19 \leq k \leq 16$, $-19 \leq l \leq 21$
Reflections collected	45092
Independent reflections	12326 [R_{int} = 0.0462, R_{sigma} = 0.0400]
Data/restraints/parameters	12326/0/574
Goodness-of-fit on F^2	1.067
Final R indexes [I>=2σ (I)]	R_1 = 0.0553, wR_2 = 0.1548
Final R indexes [all data]	R_1 = 0.0604, wR_2 = 0.1593
Largest diff. peak/hole / e Å^{-3}	2.55/-0.75

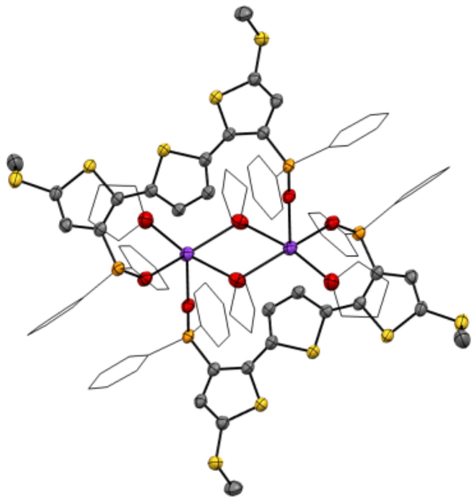

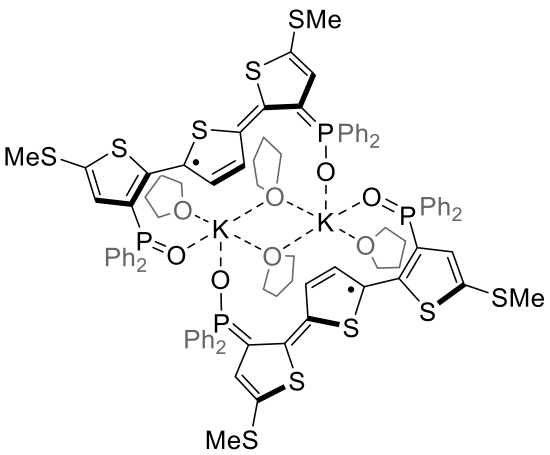

Crystal data and structure refinement of 4⁻

Identification code	2394631
Empirical formula	$C_{54}H_{66}NO_2P_2S_5$
Formula weight	983.31
Temperature/K	100.00(10)
Crystal system	monoclinic
Space group	$P2_1/c$
a/Å	15.71820(10)
b/Å	20.0001(2)
c/Å	16.60120(10)
α/°	90
β/°	100.0110(10)
γ/°	90
Volume/Å^3	5139.38(7)
Z	4
ρ_{calc}g/cm^3	1.271
μ/mm^{-1}	2.980
F(000)	2092.0
Crystal size/mm^3	0.124 × 0.064 × 0.047
Radiation	Cu Kα (λ = 1.54184)
2Θ range for data collection/°	5.71 to 160.38
Index ranges	$-20 \leq h \leq 19$, $-25 \leq k \leq 24$, $-20 \leq l \leq 21$
Reflections collected	48054
Independent reflections	11016 [R_{int} = 0.0345, R_{sigma} = 0.0292]
Data/restraints/parameters	11016/0/583
Goodness-of-fit on F^2	1.034
Final R indexes [I>=2σ (I)]	R_1 = 0.0321, wR_2 = 0.0764
Final R indexes [all data]	R_1 = 0.0381, wR_2 = 0.0791
Largest diff. peak/hole / e Å^{-3}	0.37/-0.33

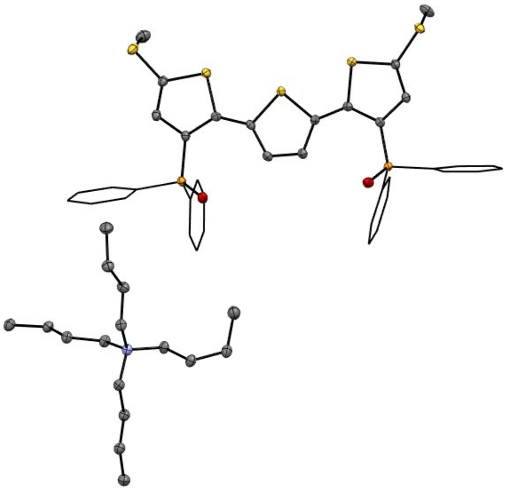

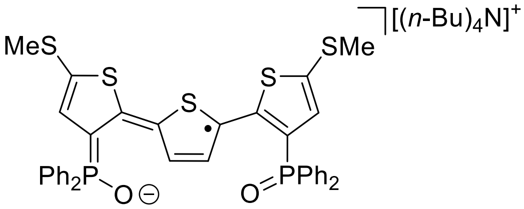

Terthiophene Solid-State Bond Length Comparison

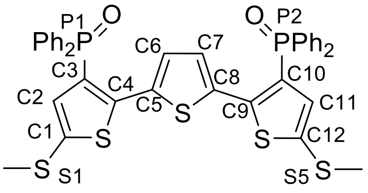

Figure S82. Bond numbering of the terthiophene backbone.

Table S5. Key terthiophene bond length comparisons between **4**, **[K(THF)$_2$(4)]$_2$** and **4$^-$.**

Bond	**4**<br>[exp, Å]	**[K(THF)$_2$(4)]$_2$**<br>[exp, Å]	**4$^-$**<br>[exp, Å]
P1-C3	1.8072(16)	1.786(2)	1.7887(15)
C3-C4	1.390(2)	1.419(3)	1.421(2)
C4-C5	1.461(2)	1.415(3)	1.417(2)
C5-C6	1.375(2)	1.396(3)	1.404(2)
C6-C7	1.412(2)	1.389(3)	1.387(2)
C7-C8	1.376(2)	1.409(3)	1.404(2)
C8-C9	1.459(2)	1.411(3)	1.4162(19)
C9-C10	1.384(2)	1.413(3)	1.4259(19)
C10-P2	1.8076(16)	1.777(2)	1.7766(15)
C1-S1	1.753(2)	1.744(3)	1.745(2)
C12-S5	1.750(2)	1.742(3)	1.741(2)

Packing Diagrams

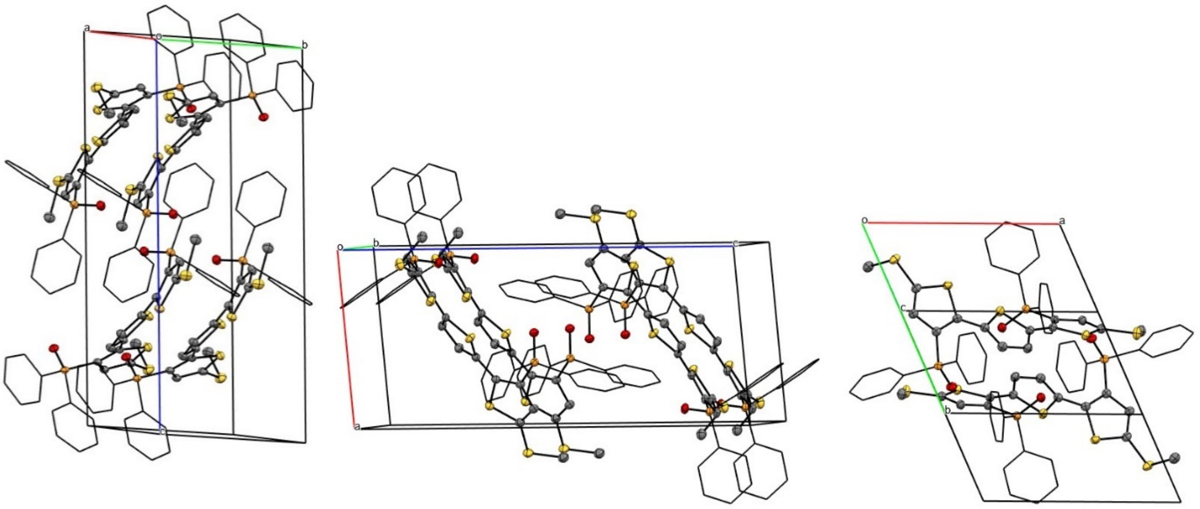

Figure S83. Packing of **4** along the a, b and c axis in the solid-state with 50% probability ellipsoids. Hydrogen atoms are omitted, phenyl substituents are represented without probability ellipsoids for clarity.

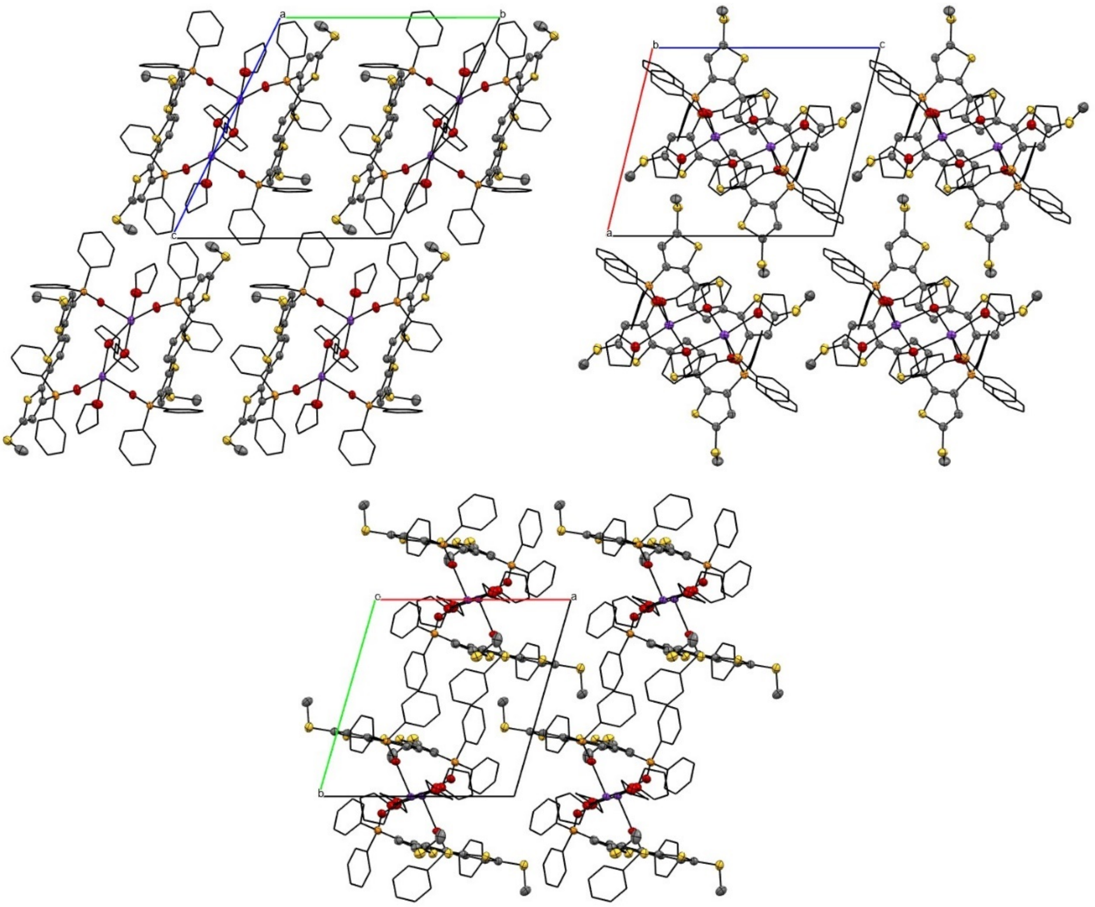

Figure S84. Packing of $[K(THF)_2(\mathbf{4})]_2$ along the a, b and c axis in the solid-state with 50% probability ellipsoids. Hydrogen atoms are omitted, phenyl substituents are represented without probability ellipsoids for clarity.

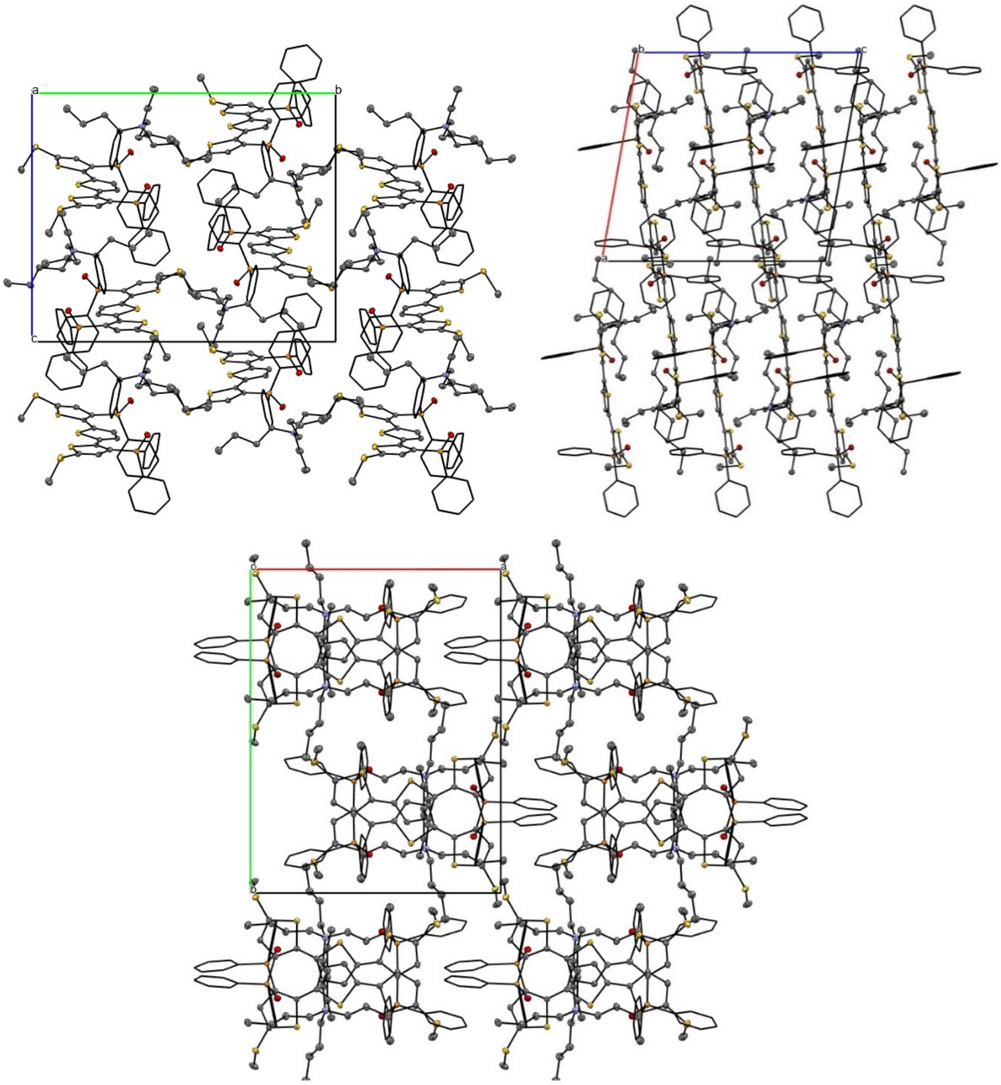

Figure S85. Packing of [(n-Bu)$_4$N][**4**] along the a, b and c axis in the solid-state with 50% probability ellipsoids. Hydrogen atoms are omitted, phenyl substituents are represented without probability ellipsoids for clarity.

XIII. Computational Data

General Considerations

All computations were carried out using the ORCA 5.0[31] program package. Orbitals and spin-density plots were visualized using Chemcraft.

Geometry optimizations and free energy calculations were performed at the BP86/def2-TZVP level of theory in the gas phase.[32–34] Numerical frequency calculations were carried out at the same level of theory to confirm the nature of stationary points located by geometry optimizations and to confirm zero imaginary frequencies. The RI approximation with general def2/J basis set was used to accelerate computations.

TD-DFT calculations were performed on the pre-optimized structures at the ωB97-D3 def2-TZVP level of theory using the CPCM model.[35] The RIJCOSX[36] approximation with general def2/J basis set was used to accelerate the computations.

Hyperfine couplings were calculated based on the optimized structures at the DFT level of theory, using the hybrid functional PBE0[37], using the dedicated EPR-property basis set, EPR-III[38] at the triple zeta level for light atoms, in combination with def2-TZVPP[39] for the elements S and P. The atom-pairwise dispersion correction D3 with Becke-Johnson damping scheme[40,41] was included for consistency. The SCF convergence was set to very tight in ORCA, in combination with fine integration grids (DefGrid3). The solvent, 2-MeTHF, was modelled using the conductor-like polarizable continuum model (CPCM)[35] using a dielectric constant of 6.97 and a refractive index of 1.4059.[42]

Sample ORCA input file for geometry optimization and frequency calculation:

```
! BP86 def2-TZVP

! RI def2/J

! Opt

! Freq

! Normalprint Printbasis PrintMOs


%pal nprocs 8 end

%maxcore 4000


*xyz “charge” “spin multiplicity”

XYZ Coordinates

*
```

Sample ORCA input file for TD-DFT calculation:

```
! wB97X-D3 def2-TZVP
! RIJCOSX def2/J
! CPCM
%cpcm
epsilon 13.8
# Dielectric Constant of 1,2-Difluorobenzene
end



%pal nprocs 16 end
%maxcore 8000

%tddft
nroots 150
maxdim 5
end
```

Sample ORCA input file for EPR hyperfine coupling calculation:

```
! UKS PBE0 EPR-III D3BJ VeryTightSCF NoRI DefGrid3

*xyzfile "charge" "spin multiplicity" filename.xyz

%basis
 newgto S "def2-TZVPP" end
 newgto P "def2-TZVPP" end
end

%cpcm
    epsilon 6.97
    refrac 1.4059
end
```

%eprnmr Nuclei = all P { aiso, adip, fgrad, rho }

Nuclei = all H { aiso, adip, fgrad, rho }

end

Time-Dependent DFT Calculations

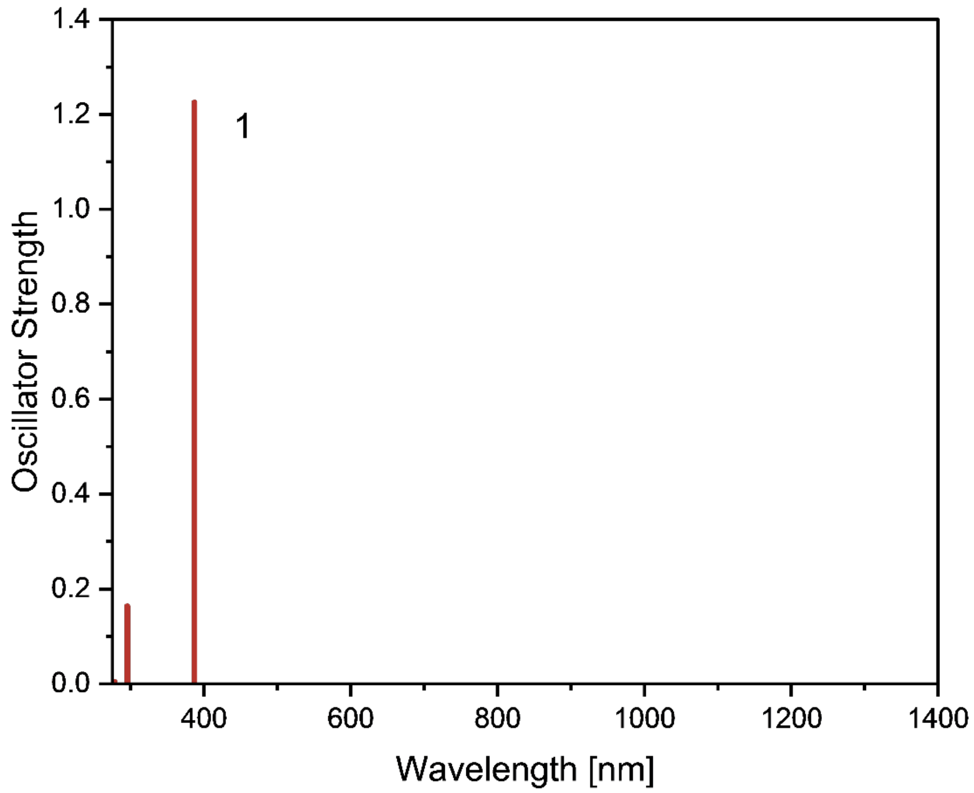

Figure S86. Data from the TDDFT calculations of **4** at the CPCM(1,2-DFB) ωB97-D3/def2-TZVP level of theory.

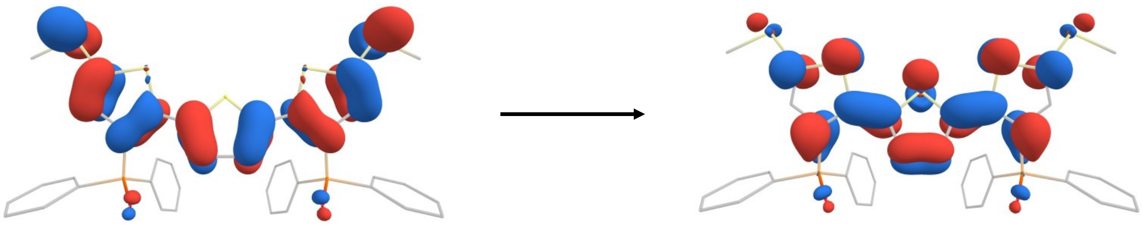

Figure S87. Molecular orbital plots of the dominant transition (94.4%) from transition #1 at 387 nm.

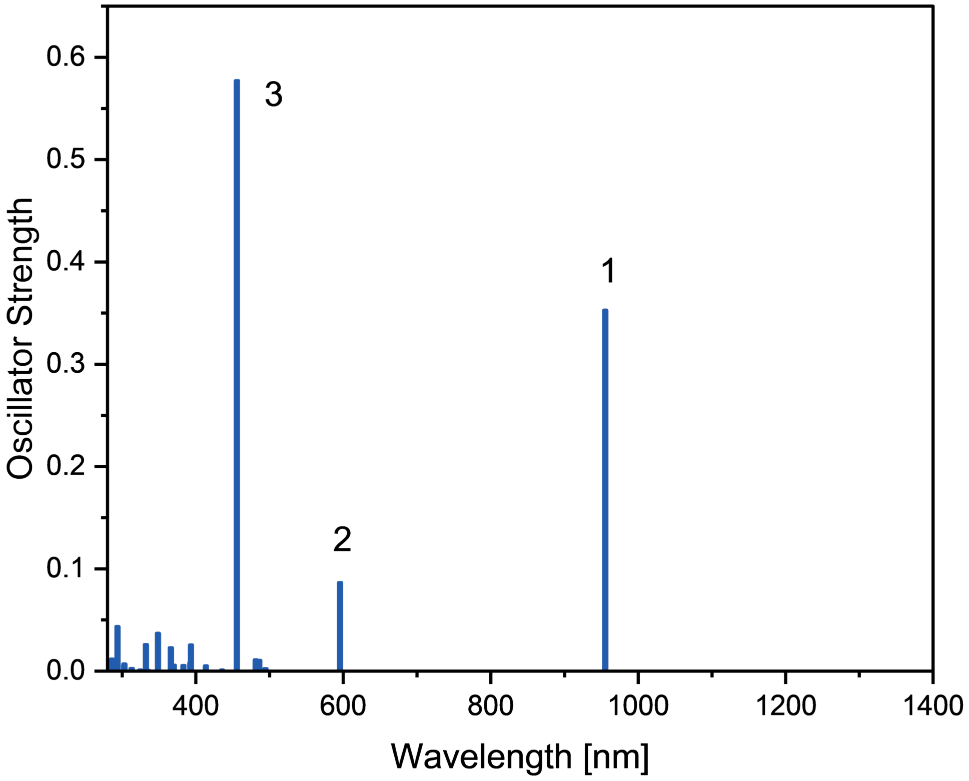

Figure S88. Data from the TDDFT calculations of **4**⁻ at the CPCM(1,2-DFB) ωB97-D3/def2-TZVP level of theory.

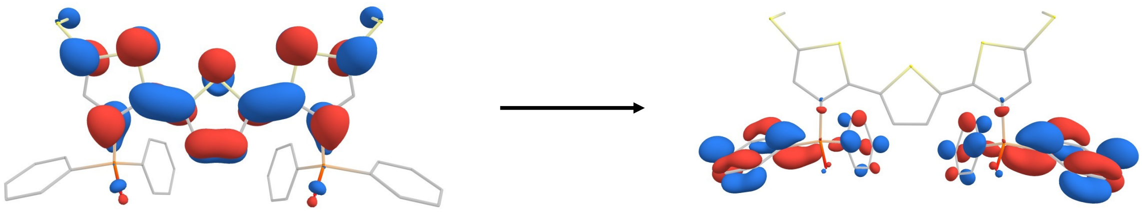

SOMO LUMO

Figure 89. Molecular orbital plots of the dominant transition (86.7%) from transition #1 at 955 nm.

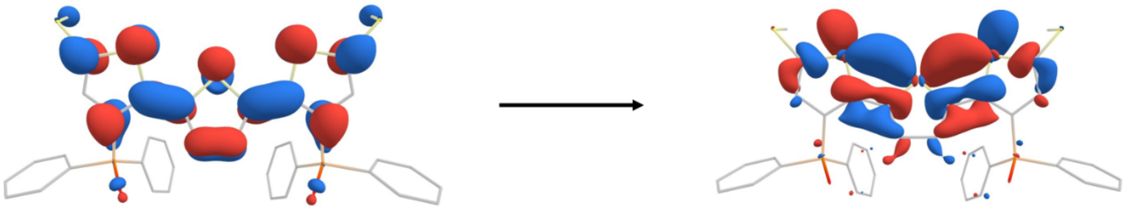

SOMO LUMO+9

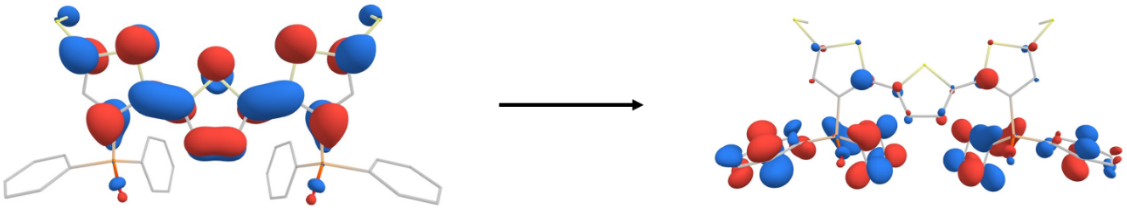

SOMO LUMO+4

Figure S90. Molecular orbital plots of the dominant transitions (71.6% and 8.1%) from transition #2 at 596 nm.

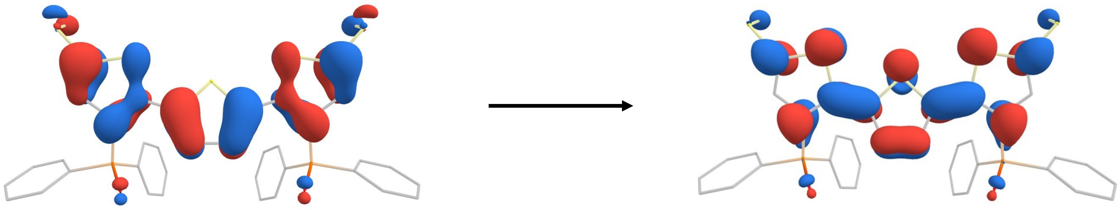

SOMO−1 SOMO

Figure S91. Molecular orbital plots of the dominant transition (79.3%) from transition #3 at 456 nm.

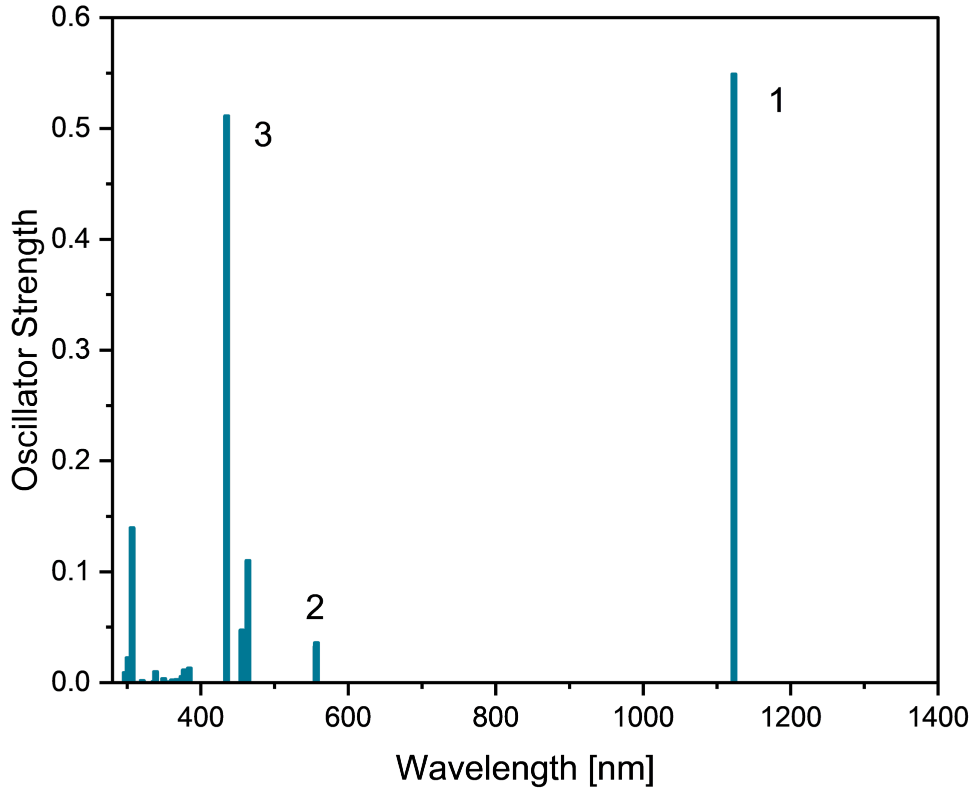

Figure S92. Data from the TDDFT calculations of **4**$^{+}$ at the CPCM(1,2-DFB) ωB97-D3/def2-TZVP level of theory.

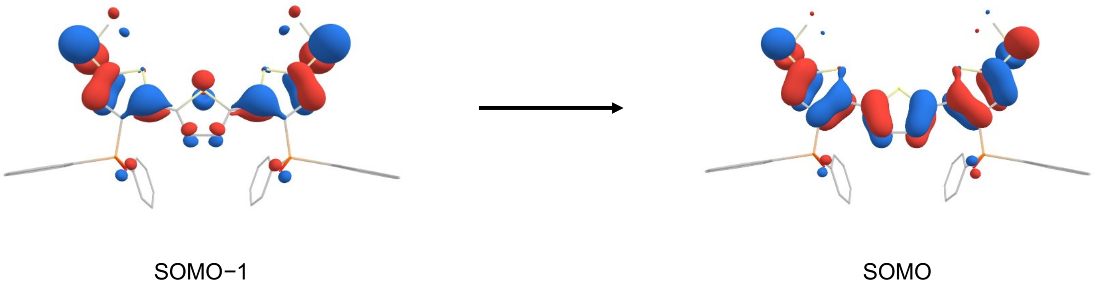

Figure S93. Molecular orbital plots of the dominant transition (92.1%) from transition #1 at 1123 nm.

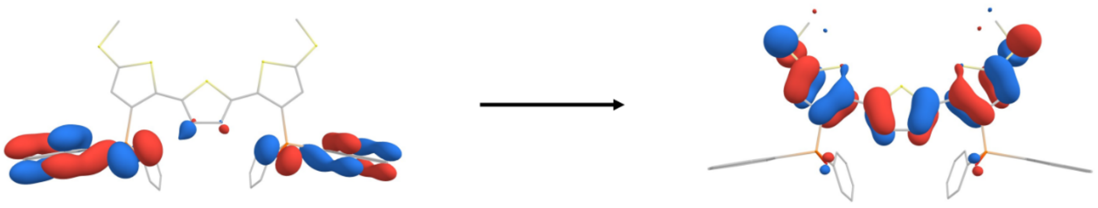

SOMO−2 SOMO

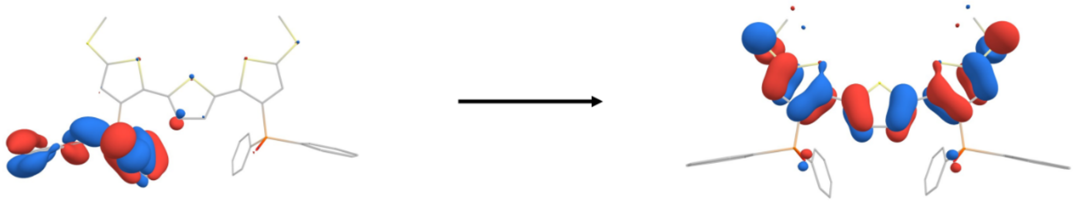

SOMO−13 SOMO

Figure S94. Molecular orbital plots of the dominant transitions (37.3% and 28.5%) from transition #2 at 557 nm.

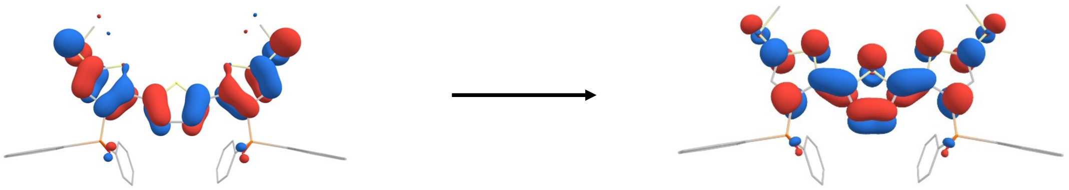

SOMO LUMO

Figure S95. Molecular orbital plots of the dominant transition (68.3%) from transition #3 at 435 nm

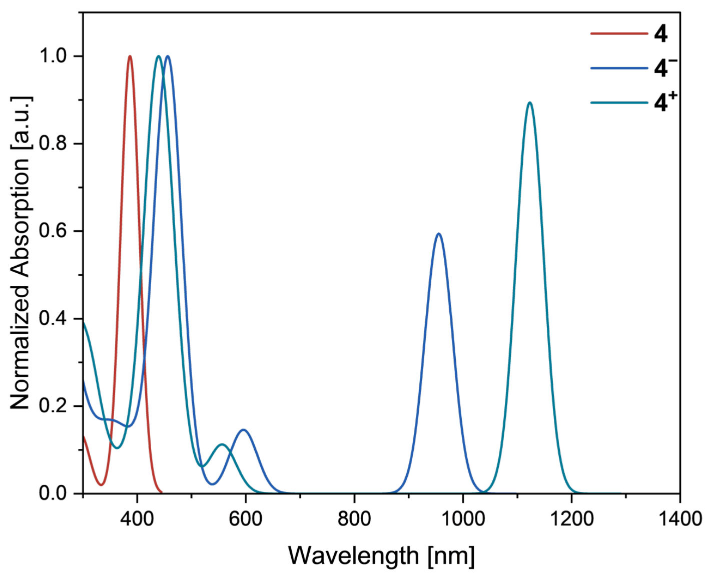

Figure S96. TD-DFT calculated UV-Vis spectra of compounds **4**, **4**⁻ and **4**⁺ using the CPCM solvation model with 1,2-difluorobenzene (ε=13.8).

Q–H BDFE Calculations of Quinone-Based HAT Reagents

BDFEs of Q–H bonds in the quinone-based hydrogen atom transfer (HAT) reagents were evaluated according to a method presented by Chirik and coworkers.[43] First, the obtained BDEs were converted to gas-phase BDFEs using the assumption that the entropic contributions of Q· and Q–H are approximately identical due to their similar size and geometry ($S°_{gas}$(Q·) ≈ $S°_{gas}$(Q–H)).[44] A literature value for the entropy term for the hydrogen atom $S°_{gas}$(H·) of 114.72 J mol^{-1} K^{-1} was used, which allows calculation of the gas-phase BDFEs at 289.15 K.[45]

$$BDFE_{gas}(Q\text{–}H) = BDE_{gas}(Q\text{–}H) - TS°_{gas}(H\cdot) - T\{S°_{gas}(Q\cdot) - S°_{gas}(Q\text{–}H)\} \quad (S13)$$

$$BDFE_{gas}(Q\text{–}H) \approx BDE_{gas}(Q\text{–}H) - TS°_{gas}(H\cdot) \quad (S14)$$

$$BDFE_{gas}(Q\text{–}H) \approx BDE_{gas}(Q\text{–}H) - 8.17\ kcal\ mol^{-1} \quad (S15)$$

The gas-phase BDFEs were then used to calculate the solution-phase BDFEs in MeCN. In this step, it was assumed that the free energies of solvation of Q· and Q–H are approximately identical (ΔG_{solv}°(Q·) ≈ ΔG_{solv}°(Q–H). Further, the free energy of solvation of H· (ΔG_{solv}°(H·) was approximated as that of H_2 in the same solvent (ΔG_{solv}°(H_2)).[46] For MeCN, the free energy of solvation of H_2 is reported as 5.12 kcal mol^{-1}.[46]

$$BDFE_{solv}(Q\text{–}H) = BDFE_{gas}(Q\text{–}H) + \Delta G_{solv}°(H\cdot) + \Delta G_{solv}°(Q\cdot) - \Delta G_{solv}°(Q\text{–}H) \quad (S16)$$

$$BDFE_{solv}(Q\text{–}H) \approx BDFE_{gas}(Q\text{–}H) + \Delta G_{solv}°(H_2) \quad (S17)$$

$$BDFE_{solv}(Q\text{–}H) \approx BDFE_{gas}(Q\text{–}H) + 5.12\ kcal\ mol^{-1} \quad (S18)$$

Table S6. Calculated BDFEs of quinone-based HAT reagents used in this study and corresponding average BDFE values simulated in MeCN.

Entry	Process	BDE_{gas} (kcal mol^{-1})	$BDFE_{gas}$ (kcal mol^{-1})	$BDFE_{solv}$ (kcal mol^{-1})
1	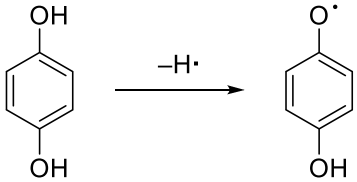	76.91	68.74	73.86
2	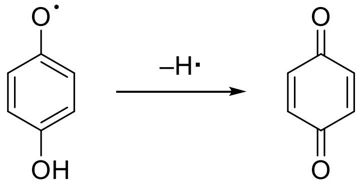	64.08	55.91	61.03
3	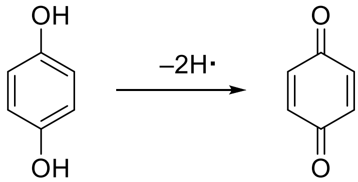	average $BDFE_{solv}$ 67 kcal mol^{-1}		
4	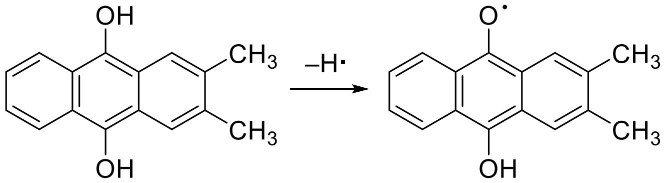	62.67	54.50	59.62
5	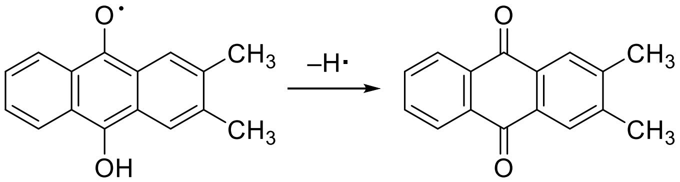	49.69	41.52	46.64
6	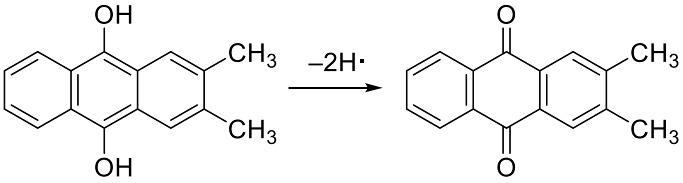	average $BDFE_{solv}$ 53 kcal mol^{-1}		

EPR Coupling Constants

Table S7. Comparison of ^{31}P hyperfine couplings experimentally obtained and calculated by DFT.

Molecule	Experimental ^{31}P hyperfine coupling constants [MHz]	DFT calculated ^{31}P hyperfine coupling constants [MHz]
**4⁻**	9.90 13.50 13.50	–10.71 –14.77 –16.90<br>–10.76 –14.94 –17.06
**4⁺**	7.99 7.68 8.84	–7.62 –8.04 –9.48<br>–7.62 –8.05 –9.49

Consistent with the experimental ^{31}P hyperfine coupling constants, DFT suggests that both ^{31}P centers are nearly equivalent. The calculated ^{31}P coupling constants of **4⁻** and **4⁺** match well with the experimentally obtained numbers.

Coordinates

4

S = 0

Final Gibbs free Energy: –4290.77913019 Eh

S	2,77244	8,97711	6,93553
S	4,86001	6,56019	6,58920
P	0,45018	12,89787	9,25974
S	1,96077	11,64154	5,35103
P	4,33869	6,18037	10,91488
O	1,57959	13,02672	10,24059
O	4,92051	7,37502	11,61416
C	3,63135	8,31394	8,30738
C	4,31039	7,04500	8,18416
C	2,40009	10,45940	7,78718
C	0,94187	12,59981	7,53062
C	-0,73118	11,57115	9,68382
C	4,67897	6,08073	9,12793
C	5,38454	4,98010	8,54902
H	5,75132	4,14088	9,13453
C	1,73840	11,53797	7,08957
C	5,00510	4,62498	11,61964
C	0,52561	13,45978	6,46658
H	-0,09489	14,33770	6,62696
C	3,57516	9,19969	9,37847
H	4,07343	8,98843	10,32578
C	5,54560	5,07773	7,18190
C	2,88731	10,39640	9,08855
H	2,77800	11,23046	9,78298
C	0,97589	13,06705	5,22262
C	4,93372	2,24239	12,07987
H	4,43279	1,27757	11,98327
C	2,52658	6,04577	11,09633
C	0,08491	15,60976	9,65180
H	1,12063	15,55596	9,99143
C	-0,61478	16,81883	9,66978
H	-0,11715	17,72780	10,01202
C	-2,58960	15,69262	8,83725
H	-3,63715	15,72038	8,53227
C	-1,89221	14,48227	8,81658
H	-2,40203	13,56965	8,50204
C	-1,47737	10,87322	8,72245
H	-1,31983	11,07232	7,66084
C	4,37257	3,37846	11,49220
H	3,43178	3,29597	10,94471
C	6,19655	4,72324	12,35452

H	6,66287	5,70295	12,47175
C	6,12704	2,34407	12,80241
H	6,56099	1,45647	13,26611
C	1,70401	5,42434	10,14479
H	2,13388	5,05398	9,21222
C	-0,91559	11,28470	11,04607
H	-0,31384	11,81334	11,78741
C	1,95970	6,55308	12,27645
H	2,60106	7,05866	13,00049
C	6,75560	3,58470	12,94096
H	7,68083	3,66886	13,51357
C	0,33208	5,29867	10,37808
H	-0,30369	4,82048	9,63106
C	-2,40748	9,90918	9,12026
H	-2,98026	9,36490	8,36751
C	-0,22471	5,79357	11,56035
H	-1,29648	5,69549	11,74083
C	-2,59593	9,63709	10,47782
H	-3,32269	8,88381	10,78675
C	-1,84624	10,32186	11,43936
H	-1,98369	10,10201	12,49930
C	0,58902	6,42510	12,50603
H	0,15383	6,82413	13,42368
C	-1,95075	16,86236	9,26071
H	-2,49857	17,80607	9,28153
C	-0,54787	14,43466	9,21695
S	0,71809	13,76362	3,63580
S	6,30039	3,98969	6,03462

4^{+}

S = 1/2
Final Gibbs free Energy: –4290.56402072 Eh

S	11,02100	18,38755	10,82901
S	8,39532	16,99330	9,75986
P	12,52231	14,25629	11,38452
S	5,36073	17,53409	9,11981
P	4,90774	13,12742	9,26515
S	13,69119	19,73655	11,51013
S	2,49631	18,06009	8,15483
O	11,54805	13,53700	12,27053
O	5,44828	12,66654	10,58684
C	5,89832	15,87888	9,40541
C	9,13988	14,75962	10,86555
H	9,74792	14,03431	11,41298
C	10,89545	16,62916	10,86513
C	13,12804	17,03281	11,45868

H	14,14860	16,79522	11,75451
C	9,63202	16,04780	10,56717
C	3,19342	12,57891	9,00004
C	4,84532	14,96964	9,12207
C	5,87982	12,57034	7,83190
C	3,67523	15,62281	8,70246
H	2,75486	15,09944	8,44867
C	12,14445	16,05865	11,22385
C	12,57340	13,62961	9,67729
C	7,22889	15,68727	9,86963
C	7,81901	14,56183	10,48304
H	7,23663	13,66023	10,69092
C	14,21954	14,18725	12,03554
C	3,78355	17,01710	8,63730
C	12,28859	12,26524	9,49970
H	12,00679	11,65976	10,36280
C	12,68892	18,35044	11,28216
C	6,52757	11,32954	7,95589
H	6,46649	10,78462	8,89942
C	12,91699	14,41843	8,56783
H	13,12763	15,48257	8,69116
C	5,97207	13,28650	6,62792
H	5,48412	14,25771	6,52524
C	2,42411	12,32422	10,14801
H	2,87458	12,45267	11,13339
C	7,24982	10,80976	6,88055
H	7,74873	9,84480	6,97984
C	2,63778	12,37237	7,72702
H	3,23862	12,53612	6,83071
C	0,55175	11,69016	8,74949
H	-0,47646	11,33897	8,65147
C	7,33242	11,52260	5,68062
H	7,89507	11,11280	4,84054
C	14,36060	13,98935	13,41948
H	13,46845	13,87819	14,03763
C	6,69775	12,76173	5,55577
H	6,76806	13,32166	4,62212
C	1,31806	11,93141	7,60511
H	0,89188	11,76337	6,61512
C	12,70795	12,48573	7,12534
H	12,76394	12,03984	6,13124
C	12,35816	11,69810	8,22623
H	12,13939	10,63771	8,09359
C	1,10588	11,88280	10,01859
H	0,51300	11,67967	10,91139
C	15,36159	14,28572	11,22420
H	15,26152	14,40588	10,14401

C	12,98334	13,84550	7,29549
H	13,24992	14,46196	6,43579
C	12,57351	21,12227	11,16688
H	13,18368	22,02527	11,28881
H	11,74593	21,14379	11,88698
H	12,19758	21,07564	10,13682
C	15,63476	13,90751	13,98460
H	15,74119	13,74765	15,05838

4^{-}

S = 1/2

Final Gibbs free Energy: –4290.84697089 Eh

S	2.86478	9.07320	7.03025
S	4.95935	6.64621	6.70391
P	0.47897	12.92828	9.38825
S	2.08788	11.78945	5.48492
P	4.30126	6.19826	11.00309
O	1.58937	13.04395	10.39247
O	4.84834	7.39046	11.73416
C	3.68806	8.38439	8.40937
C	4.36691	7.11176	8.28070
C	2.47597	10.53931	7.89863
C	1.00998	12.67789	7.66332
C	–0.69380	11.57793	9.75769
C	4.69627	6.12891	9.22586
C	5.41372	5.03858	8.64256
H	5.76520	4.18134	9.21309
C	1.82772	11.63385	7.20618
C	4.96449	4.64077	11.70280
C	0.62324	13.56180	6.60825
H	–0.00506	14.43672	6.76197
C	3.61156	9.25047	9.49356
H	4.08310	9.01801	10.44974
C	5.61388	5.17042	7.29153
C	2.93342	10.45501	9.20816
H	2.80663	11.27667	9.91461
C	1.11369	13.20042	5.37873
C	4.90488	2.25172	12.12806
H	4.41542	1.28391	12.00659
C	2.48595	6.04321	11.12818
C	0.07009	15.62217	9.84851
H	1.09482	15.56982	10.22010
C	–0.64360	16.82281	9.87627
H	–0.16784	17.72711	10.25944
C	–2.57611	15.70024	8.94524
H	–3.61208	15.72641	8.60310

C	–1.86520	14.49827	8.91532
H	–2.35388	13.59016	8.55727
C	–1.41891	10.89806	8.76758
H	–1.24860	11.12629	7.71380
C	4.34499	3.39116	11.54604
H	3.41569	3.30797	10.97943
C	6.14104	4.73887	12.46142
H	6.59758	5.72024	12.60083
C	6.08342	2.35320	12.87422
H	6.51710	1.46253	13.33214
C	1.69758	5.43071	10.14258
H	2.15830	5.07927	9.21741
C	–0.89576	11.25431	11.10908
H	–0.31062	11.76946	11.87286
C	1.87986	6.52444	12.29978
H	2.49498	7.02278	13.05107
C	6.69844	3.59690	13.04236
H	7.61242	3.68046	13.63279
C	0.32072	5.28775	10.33339
H	–0.28808	4.81576	9.56040
C	–2.34549	9.91547	9.12614
H	–2.90211	9.38607	8.35099
C	–0.27504	5.75644	11.50728
H	–1.35042	5.64418	11.65505
C	–2.55157	9.60655	10.47319
H	–3.27588	8.83913	10.75146
C	–1.82285	10.27308	11.46322
H	–1.97416	10.02477	12.51494
C	0.50452	6.37916	12.48702
H	0.03885	6.75701	13.39865
C	–1.96509	16.86396	9.42280
H	–2.52335	17.80134	9.44951
C	–0.53475	14.45359	9.35997
H	0.96375	13.69362	4.42274
H	6.11888	4.48624	6.61586

Q–H_2

S = 0

Final Gibbs free Energy: –382.79030401 Eh

C	–4,73013	2,63054	–0,00559
C	–5,91518	1,88592	0,04186
C	–5,86594	0,49057	0,09487
C	–4,63213	–0,17425	0,10039
C	–3,44708	0,57037	0,05294

C	−3,49632	1,96572	−0,00007
H	−2,48637	0,05290	0,05743
H	−2,56848	2,54208	−0,03723
O	−4,83627	4,00418	−0,05618
O	−4,52599	−1,54789	0,15097
H	−3,94108	4,39134	−0,08600
H	−5,42118	−1,93505	0,18080
H	−6,79378	−0,08579	0,13203
H	−6,87589	2,40339	0,03737

Q–H

S = 1/2
Final Gibbs free Energy: –382.17020150 Eh

C	−4,72408	2,63303	0,00000
C	−5,94352	1,90762	0,00000
C	−5,92129	0,53415	0,00000
C	−4,66994	−0,20714	0,00000
C	−3,44763	0,58033	0,00000
C	−3,47842	1,95499	0,00000
H	−6,88253	2,46378	0,00000
H	−6,84896	−0,04086	0,00000
H	−2,49839	0,04172	0,00000
H	−2,55500	2,54003	0,00000
O	−4,81281	3,97972	0,00000
O	−4,64544	−1,47486	0,00000
H	−3,91880	4,37957	0,00000

Q

S = 0
Final Gibbs free Energy: –381.56931481 Eh

C	−4,83928	2,66811	−0,00080
C	−6,06349	1,83391	−0,00006
C	−5,98823	0,48852	−0,00006
C	−4,67887	−0,20418	−0,00079
C	−3,45466	0,63002	−0,00006

C	−3,52992	1,97541	−0,00007
H	−7,01679	2,36621	0,00035
H	−6,87652	−0,14643	0,00035
H	−2,50135	0,09772	0,00035
H	−2,64163	2,61036	0,00035
O	−4,90811	3,90313	0,00022
O	−4,61004	−1,43920	0,00022

DMAQ–H_2

S = 0

Final Gibbs free Energy: −768.69781212 Eh

O	0,97321	−0,01612	0,00750
C	2,34753	0,03921	0,00035
C	3,02724	−1,19713	−0,00906
C	4,47334	−1,21504	−0,03014
C	5,14050	−2,47778	−0,05286
C	4,43070	−3,65750	−0,04919
C	3,00846	−3,63991	−0,02088
C	2,32852	−2,44290	−0,00189
C	3,05670	1,25905	−0,00918
C	4,50587	1,24104	−0,00261
C	5,18668	2,49561	0,02431
C	4,53212	3,70932	0,02211
C	5,30748	4,99942	0,05235
C	3,09180	3,72735	−0,01147
C	2,34873	5,03645	−0,03116
C	2,40730	2,53034	−0,02513
C	5,18412	0,00380	−0,02369
O	6,55641	−0,08682	−0,02885
H	6,38817	4,80894	0,07832
H	5,04356	5,60712	0,93261
H	5,08839	5,61957	−0,83144
H	2,62806	5,64483	−0,90619

H	2,58215	5,64395	0,85779
H	1,26359	4,87322	–0,05923
H	1,31483	2,58429	–0,05879
H	0,60877	0,87735	0,14717
H	1,23896	–2,43125	0,01886
H	4,96015	–4,61173	–0,06733
H	2,45524	–4,58072	–0,01503
H	6,23003	–2,49349	–0,07340
H	6,28016	2,52237	0,05618
H	6,94465	0,80019	–0,14324

DMAQ–H

S = 1/2
Final Gibbs free Energy: –768.10144220 Eh

O	1,01422	0,19973	–0,02304
C	2,36441	0,09762	–0,02056
C	3,03499	–1,16558	–0,01782
C	4,46831	–1,19326	–0,01536
C	5,13171	–2,43338	–0,01265
C	4,42556	–3,62958	–0,01232
C	3,01656	–3,60873	–0,01475
C	2,33476	–2,40237	–0,01744
C	3,08597	1,32833	–0,02083
C	4,51250	1,31449	–0,01843
C	5,19843	2,54142	–0,01873
C	4,54031	3,76892	–0,02131
C	5,32111	5,05447	–0,02179
C	3,11143	3,78477	–0,02364
C	2,36173	5,08954	–0,02691
C	2,41953	2,58022	–0,02334
C	5,26161	0,05030	–0,01563
O	6,51680	0,02453	–0,01340
H	6,40094	4,85967	–0,02078
H	5,07942	5,67013	0,85953

H	5,08068	5,66860	−0,90451
H	2,62199	5,70199	0,85096
H	1,27730	4,92229	−0,02194
H	2,61459	5,69288	−0,91330
H	1,32911	2,59649	−0,02496
H	0,60001	−0,68496	−0,02262
H	1,24223	−2,43386	−0,01921
H	4,95742	−4,58204	−0,01020
H	2,45604	−4,54468	−0,01450
H	6,22244	−2,42821	−0,01079
H	6,28990	2,51370	−0,01685

DMAQ

S = 0

Final Gibbs free Energy: −767.52240288 Eh

O	1,04927	0,10915	−0,02932
C	2,28651	0,09534	−0,02789
C	3,03951	−1,19190	−0,02587
C	4,45288	−1,21191	−0,02355
C	5,12716	−2,44163	−0,02182
C	4,41230	−3,63911	−0,02234
C	3,01102	−3,61928	−0,02463
C	2,33050	−2,40197	−0,02637
C	3,07659	1,35255	−0,02655
C	4,48852	1,33262	−0,02427
C	5,18658	2,54749	−0,02341
C	4,52757	3,78065	−0,02452
C	5,31416	5,06151	−0,02277
C	3,10687	3,80068	−0,02688
C	2,35622	5,10277	−0,02568
C	2,41324	2,58662	−0,02798
C	5,24237	0,05335	−0,02291
O	6,47950	0,03164	−0,02116
H	6,21758	−2,44173	−0,02005

H	4,94523	−4,59114	−0,02096
H	2,45138	−4,55586	−0,02507
H	1,24052	−2,37093	−0,02822
H	6,27771	2,51826	−0,02165
H	6,39301	4,86355	−0,01607
H	5,06693	5,67754	0,85635
H	5,07761	5,67341	−0,90775
H	2,63557	5,72450	−0,89086
H	2,59399	5,69520	0,87213
H	1,27244	4,93549	−0,05253
H	1,32175	2,58848	−0,03047

XIV. References

(1) Facchetti, A.; Yoon, M. H.; Stern, C. L.; Hutchison, G. R.; Ratner, M. A.; Marks, T. J. Building Blocks for N-Type Molecular and Polymeric Electronics. Perfluoroalkyl- versus Alkyl-Functionalized Oligothiophenes (NTs; n = 2-6). Systematic Synthesis, Spectroscopy, Electrochemistry, and Solid-State Organization. *J. Am. Chem. Soc.* **2004**, *126* (41), 13480–13501. https://doi.org/10.1021/ja048988a.

(2) Brookhart, M.; Grant, B.; Volpe, A. F. Synthesis of HBArF x 2Et2O. *Organometallics* **1992**, *11* (11), 3920–3922.

(3) Schmidt, W.; Steckhan, E. Über Organische Elektronenüberträgersysteme, I. Elektrochemische Und Spektroskopische Untersuchung Bromsubstituierter Triarylamin-Redoxsysteme. *Chem. Ber.* **1980**, *113* (2), 577–585. https://doi.org/10.1002/cber.19801130215.

(4) Krossing, I. The Facile Preparation of Weakly Coordinating Anions: Structure and Characterisation of Silverpolyfluoroalkoxyaluminates AgAl(ORF)4, Calculation of the Alkoxide Ion Affinity. *Chem. Eur. J.* **2001**, *7* (2), 490–502. https://doi.org/10.1002/1521-3765(20010119)7:2<490::AID-CHEM490>3.0.CO;2-I.

(5) Käch, D.; Gasser, A. C.; Wettstein, L.; Schweinzer, C.; Bezdek, M. J. Phosphine Oxide-Functionalized Terthiophene Redox Systems. *Angew. Chem. Int. Ed.* **2023**, *62* (29). https://doi.org/10.1002/anie.202304600.

(6) Capozzi, B.; Dell, E. J.; Berkelbach, T. C.; Reichman, D. R.; Venkataraman, L.; Campos, L. M. Length-Dependent Conductance of Oligothiophenes. *J. Am. Chem. Soc.* **2014**, *136* (29), 10486–10492. https://doi.org/10.1021/ja505277z.

(7) Raabe, I.; Wagner, K.; Guttsche, K.; Wang, M.; Grätzel, M.; Santiso-Quiñones, G.; Krossing, I. Tetraalkylammonium Salts of Weakly Coordinating Aluminates: Ionic Liquids, Materials for Electrochemical Applications and Useful Compounds for Anion Investigation. *Chem. Eur. J.* **2009**, *15* (8), 1966–1976. https://doi.org/10.1002/chem.200800417.

(8) Suffert, J. Simple Direct Titration of Organolithium Reagents Using N-Pivaloyl-o-Toluidine and/or N-Pivaloyl-o-Benzylaniline. *J. Org. Chem* **1989**, *54*, 509–510.

(9) Eaton, G. R.; Eaton, S. S.; Barr, D. P.; Weber, R. T. *Quantitative EPR*; Springer: Vienna, 2010.

(10) Gromov, I.; Shane, J.; Forrer, J.; Rakhmatoullin, R.; Rozentzwaig, Y.; Schweiger, A. A Q-Band Pulse EPR/ENDOR Spectrometer and the Implementation of Advanced One- and Two-Dimensional Pulse EPR Methodology. *J. Magn. Reson.* **2001**, *149* (2), 196–203. https://doi.org/10.1006/jmre.2001.2298.

(11) Tschaggelar, R.; Kasumaj, B.; Santangelo, M. G.; Forrer, J.; Leger, P.; Dube, H.; Diederich, F.; Harmer, J.; Schuhmann, R.; García-Rubio, I.; Jeschke, G. Cryogenic 35 GHz Pulse ENDOR Probehead Accommodating Large Sample Sizes: Performance and Applications. *J. Magn. Reson.* **2009**, *200* (1), 81–87. https://doi.org/10.1016/j.jmr.2009.06.007.

(12) Davies, E. R. A New Pulse ENDOR Technique. *Phys. Lett.* **1974**, *47A* (1), 1–2.

(13) Epel, B.; Arieli, D.; Baute, D.; Goldfarb, D. Improving W-Band Pulsed ENDOR Sensitivity - Random Acquisition and Pulsed Special TRIPLE. *J. Magn. Reson.* **2003**, *164* (1), 78–83. https://doi.org/10.1016/S1090-7807(03)00191-5.

(14) Höfer, P.; Grupp, A.; Nebenführ, H.; Mehring, M. Hyperfine Sublevel Correlation (HYSCORE) Spectroscopy: A 2D ESR Investigation of the Squaric Acid Radical. *Chem. Phys. Lett.* **1986**, *132* (3).

(15) Stoll, S.; Schweiger, A. EasySpin, a Comprehensive Software Package for Spectral Simulation and Analysis in EPR. *J. Magn. Reson.* **2006**, *178* (1), 42–55. https://doi.org/10.1016/j.jmr.2005.08.013.

(16) Fábregas Ibáñez, L.; Soetbeer, J.; Klose, D.; Tinzl, M.; Hilvert, D.; Jeschke, G. Non-Uniform HYSCORE: Measurement, Processing and Analysis with Hyscorean. *J. Magn. Reson.* **2019**, *307*. https://doi.org/10.1016/j.jmr.2019.106576.

(17) Dolomanov, O. V.; Bourhis, L. J.; Gildea, R. J.; Howard, J. A. K.; Puschmann, H. OLEX2: A Complete Structure Solution, Refinement and Analysis Program. *J. Appl. Crystallogr.* **2009**, *42* (2), 339–341. https://doi.org/10.1107/S0021889808042726.

(18) Sheldrick, G. M. SHELXT - Integrated Space-Group and Crystal-Structure Determination. *Acta Crystallogr A* **2015**, *71* (1), 3–8. https://doi.org/10.1107/S2053273314026370.

(19) Espinoza, E. M.; Clark, J. A.; Soliman, J.; Derr, J. B.; Morales, M.; Vullev, V. I. Practical Aspects of Cyclic Voltammetry: How to Estimate Reduction Potentials When Irreversibility Prevails. *J. Electrochem. Soc.* **2019**, *166* (5), H3175–H3187. https://doi.org/10.1149/2.0241905jes.

(20) Sevov, C. S.; Hickey, D. P.; Cook, M. E.; Robinson, S. G.; Barnett, S.; Minteer, S. D.; Sigman, M. S.; Sanford, M. S. Physical Organic Approach to Persistent, Cyclable, Low-Potential Electrolytes for Flow Battery Applications. *J. Am. Chem. Soc.* **2017**, *139* (8), 2924–2927. https://doi.org/10.1021/jacs.7b00147.

(21) Schorpp, M.; Heizmann, T.; Schmucker, M.; Rein, S.; Weber, S.; Krossing, I. Synthesis and Application of a Perfluorinated Ammoniumyl Radical Cation as a Very Strong Deelectronator. *Angew. Chem. Int. Ed.* **2020**, *59* (24), 9453–9459. https://doi.org/10.1002/anie.202002768.

(22) Brunner, E. Solubility of Hydrogen in 10 Organic Solvents at 298.15, 323.15, and 373.15 K. *J. Eng. Chem. Data* **1985**, *30*, 269–273.

(23) Perrin, C. L.; Fabian, M. A. Multicomponent NMR Titration for Simultaneous Measurement of Relative PKa's. *Anal. Chem.* **1996**, *68* (13), 2127–2134.

(24) Coetzee, J. F. Ionic Reactions in Acetonitrile. In *Progress in Physical Organic Chemistry*; wiley, 2007; Vol. 4, pp 45–92. https://doi.org/10.1002/9780470171837.ch2.

(25) Tshepelevitsh, S.; Kütt, A.; Lõkov, M.; Kaljurand, I.; Saame, J.; Heering, A.; Plieger, P. G.; Vianello, R.; Leito, I. On the Basicity of Organic Bases in Different Media. *Eur. J. Org. Chem.* **2019**, *2019* (40), 6735–6748. https://doi.org/10.1002/ejoc.201900956.

(26) Kütt, A.; Tshepelevitsh, S.; Saame, J.; Lõkov, M.; Kaljurand, I.; Selberg, S.; Leito, I. Strengths of Acids in Acetonitrile. *Eur. J. Org. Chem.* **2021**, *2021* (9), 1407–1419. https://doi.org/10.1002/ejoc.202001649.

(27) Warren, J. J.; Tronic, T. A.; Mayer, J. M. Thermochemistry of Proton-Coupled Electron Transfer Reagents and Its Implications. *Chem. Rev.* **2010**, *110* (12), 6961–7001. https://doi.org/10.1021/cr100085k.

(28) Bordwell, F. G.; Cheng, J.-P.; Harrelson, J. A. Homolytic Bond Dissociation Energies in Solution from Equilibrium Acidity and Electrochemical Data. *J. Am. Chem. Soc* **1988**, *110*, 1229–1231.

(29) Agarwal, R. G.; Coste, S. C.; Groff, B. D.; Heuer, A. M.; Noh, H.; Parada, G. A.; Wise, C. F.; Nichols, E. M.; Warren, J. J.; Mayer, J. M. Free Energies of Proton-Coupled Electron Transfer Reagents and Their Applications. *Chem. Rev.* American Chemical Society January 12, 2022, pp 1–49. https://doi.org/10.1021/acs.chemrev.1c00521.

(30) Evans, D. F. The Determination of the Paramagnetic Susceptibility of Substances in Solution by Nuclear Magnetic Resonance. *J. Chem. Soc.* **1959**, *81* (1), 2003–2005.

(31) Neese, F.; Wennmohs, F.; Becker, U.; Riplinger, C. The ORCA Quantum Chemistry Program Package. *J. Chem. Phys.* **2020**, *152* (22). https://doi.org/10.1063/5.0004608.

(32) Becke, A. D. Density-Functional Exchange-Energy Approximation with Correct Asymptotic Behavior. *Phys. Rev. A* **1988**, *38* (6), 3098–3100.

(33) Perdew, J. P. Density-Functional Approximation for the Correlation Energy of the Inhomogeneous Electron Gas. *Phys. Rev. B* **1986**, *38*, 8822–8824.

(34) Bursch, M.; Mewes, J.-M.; Hansen, A.; Grimme, S. Best-Practice DFT Protocols for Basic Molecular Computational Chemistry**. *Angew. Chem. Int. Ed.* **2022**, *61* (42), e202205735. https://doi.org/10.26434/chemrxiv-2022-n304h.

(35) Barone, V.; Cossi, M. Quantum Calculation of Molecular Energies and Energy Gradients in Solution by a Conductor Solvent Model. *J. Phys. Chem. A* **1998**, No. 102, 1995–2001.

(36) Kossmann, S.; Neese, F. Efficient Structure Optimization with Second-Order Many-Body Perturbation Theory: The RIJCOSX-MP2 Method. *J. Chem. Theory Comp.* **2010**, *6* (8), 2325–2338. https://doi.org/10.1021/ct100199k.

(37) Adamo, C.; Barone, V. Toward Reliable Density Functional Methods without Adjustable Parameters: The PBE0 Model. *J. Chem. Phys.* **1999**, *110* (13), 6158–6170. https://doi.org/10.1063/1.478522.

(38) Rega, N.; Cossi, M.; Barone, V. Development and Validation of Reliable Quantum Mechanical Approaches for the Study of Free Radicals in Solution. *J. Chem. Phys.* **1996**, *105* (24), 11060–11067. https://doi.org/10.1063/1.472906.

(39) Weigend, F.; Ahlrichs, R. Balanced Basis Sets of Split Valence, Triple Zeta Valence and Quadruple Zeta Valence Quality for H to Rn: Design and Assessment of Accuracy. *Phys. Chem. Chem. Phys.* **2005**, *7* (18), 3297–3305. https://doi.org/10.1039/b508541a.

(40) Grimme, S.; Antony, J.; Ehrlich, S.; Krieg, H. A Consistent and Accurate Ab Initio Parametrization of Density Functional Dispersion Correction (DFT-D) for the 94 Elements H-Pu. *J. Chem. Phys.* **2010**, *132* (15). https://doi.org/10.1063/1.3382344.

(41) Grimme, S.; Ehrlich, S.; Goerigk, L. Effect of the Damping Function in Dispersion Corrected Density Functional Theory. *J. Comput. Chem.* **2011**, *32* (7), 1456–1465. https://doi.org/10.1002/jcc.21759.

(42) *CRC Handbook of Chemistry and Physics*, 105th ed.; Rumble, J., Ed.; Taylor&Francis, 2023.

(43) Park, Y.; Kim, S.; Tian, L.; Zhong, H.; Scholes, G. D.; Chirik, P. J. Visible Light Enables Catalytic Formation of Weak Chemical Bonds with Molecular Hydrogen. *Nat. Chem.* **2021**, *13* (10), 969–976. https://doi.org/10.1038/s41557-021-00732-z.

(44) Agarwal, R. G.; Coste, S. C.; Groff, B. D.; Heuer, A. M.; Noh, H.; Parada, G. A.; Wise, C. F.; Nichols, E. M.; Warren, J. J.; Mayer, J. M. Free Energies of Proton-Coupled Electron Transfer Reagents and Their Applications. *Chem. Rev.* **2022**, *122* (1), 1–49. https://doi.org/10.1021/acs.chemrev.1c00521.

(45) Afeefy, H. Y.; Liebman, J. F.; Stein, S. E. *Neutral Thermochemical Data. In NIST Chemistry WebBook, NIST Standard Reference Database Number 69*.

(46) Wise, C. F.; Agarwal, R. G.; Mayer, J. M. Determining Proton-Coupled Standard Potentials and X-H Bond Dissociation Free Energies in Nonaqueous Solvents Using Open-Circuit Potential Measurements. *J. Am. Chem. Soc.* **2020**, *142* (24), 10681–10691. https://doi.org/10.1021/jacs.0c01032.
